# How Sure Can We Be about ML Methods-Based Evaluation of Compound Activity: Incorporation of Information about Prediction Uncertainty Using Deep Learning Techniques

**DOI:** 10.3390/molecules25061452

**Published:** 2020-03-23

**Authors:** Igor Sieradzki, Damian Leśniak, Sabina Podlewska

**Affiliations:** 1 Faculty of Mathematics and Computer Science, Jagiellonian University, 6 S. Łojasiewicza Street, 30-348 Cracow, Poland; igor.sieradzki@doctoral.uj.edu.pl (I.S.); damian.lesniak@doctoral.uj.edu.pl (D.L.); 2 Department of Technology and Biotechnology of Drugs, Jagiellonian University, Medical College, 9 Medyczna Street, 30-688 Cracow, Poland; 3 Maj Institute of Pharmacology, 12 Smętna Street, 31-343 Cracow, Poland

**Keywords:** machine learning, prediction uncertainty, deep learning, ligands, ChEMBL database

## Abstract

A great variety of computational approaches support drug design processes, helping in selection of new potentially active compounds, and optimization of their physicochemical and ADMET properties. Machine learning is a group of methods that are able to evaluate in relatively short time enormous amounts of data. However, the quality of machine-learning-based prediction depends on the data supplied for model training. In this study, we used deep neural networks for the task of compound activity prediction and developed dropout-based approaches for estimating prediction uncertainty. Several types of analyses were performed: the relationships between the prediction error, similarity to the training set, prediction uncertainty, number and standard deviation of activity values were examined. It was tested whether incorporation of information about prediction uncertainty influences compounds ranking based on predicted activity and prediction uncertainty was used to search for the potential errors in the ChEMBL database. The obtained outcome indicates that incorporation of information about uncertainty of compound activity prediction can be of great help during virtual screening experiments.

## 1. Introduction

Computational methods have now become indispensable part of drug design process, supporting its every stage, from proposing new drug candidates, via optimization of their activity, to tuning their physicochemical and pharmacokinetic properties and minimizing adverse effects (computer-aided drug design, CADD) [1,2,3,4,5,6,7,8]. Great desire for new medications for various diseases is an impulse for conduction of experiments in the field, which causes the exponential growth of the amount of pharmaceutical-related data that can then be used for modeling of compounds bioactivity and properties.

There is a number of databases storing information about various aspects of biologically active compounds—from data on compounds activity towards particular target, such as the ChEMBL database [9] or PDSP [10], through the information on 3-dimensional structure of proteins (PDB) [11], data on existing drugs (DrugBank) [12] or compounds toxicity (TOXNET) [13]. The information stored in such databases can be very useful during the design of new compounds with desired biological activity; however, the great amount of information stored there makes it impossible to be carried out by simple statistical tools. Therefore, more sophisticated tools need to be use in order to derive relationships that can facilitate the process of finding new drug candidates. This is the reason why machine learning (ML) methods have recently gained such great popularity in the field of drug design. They are used both to select potential drug candidates from large compounds databases, but also to generate the structures of new chemical compounds de novo—or to optimize their physicochemical and pharmacokinetic properties [14,15,16,17,18,19,20,21,22,23,24,25,26,27].

Despite a wide range of possibilities offered by ML methods, there are also problems which leads to inaccurate predictions of compounds activity or other evaluated properties. First of all, in order to apply ML methods for dealing with cheminformatic problems, the structures of chemical compounds need to be properly represented. One of the most popular approaches to this task is fingerprinting that is translating a compound into the form of a bit string coding information about its structure [28,29,30,31,32]. There are two main types of this way of compound transformation: hashed fingerprints and key-based fingerprints and each type of compounds translation is connected with losing some pieces of information about compound structure. For example, in the key-based fingerprints, subsequent positions provide information about the presence or absence of particular chemical moieties in the molecule; however, after representing compound in such a form, information about the connections between these moieties is lost.

Another problem with application of ML methods in the process of selecting new drug candidates is related to the fact that the already known ligands of a given receptor usually cover relatively narrow chemical space [33]. It then causes, that if they are used for training a ML model, we obtain correct predictions on the activity of compounds that are structurally close to the previously examined ones, but there are difficulties in evaluation of structurally novel compounds. Generalization issues are difficult to be solved and various approaches have already been tested to improve the prediction accuracy on the molecules with dissimilar structures to known ligands [34,35].

Each computational model, before application in CADD tasks, needs to be evaluated in terms of its prediction accuracy. Such retrospective studies are also challenging, as the proper testing approach needs to be selected. It was already reported in several studies, that cross-validation (CV) with random splitting leads to overoptimistic results as we rather obtain information on a model that works via memorizing the training set than generalizing it on new data. However, other splitting approaches (such as cluster-based or scaffold-based splitting) are also not perfect; they also do not fully solve the problem of providing information about the ability of a model to evaluate structurally novel compounds [33,34].

Another problem related to application of computational tools in CADD is the fact that most of them (not only ML-based, but also pharmacophore modeling or homology modeling when model evaluation is considered) need first to be trained, which is usually performed on experimental data stored in various databases. However, as it was already indicated and what is also a subject of this study, experimental data are not always reproducible and the provided compound activity values are not always reliable [36].

There are different types of uncertainty that can be considered. Two most important categories of this problem are epistemic and aleatoric uncertainty. The latter type of uncertainty is sometimes called also a systematic uncertainty and its source is lack of knowledge of various types. It can be related to misunderstanding of the analyzed process or missing data of a particular type. Epistemic uncertainty influences evaluations of events of ‘accident’ types, such as probability of failure of particular machine and evaluation of probability of human error (when the analyst does not possess enough data to make proper decision). On the other hand, aleatoric uncertainty is also known as statistical uncertainty and is related to randomness occurring during experiment (causing differences in the obtained outcome when experiment is run several times with the same settings) [37].

Out of a wide range of ML models applied in CADD, great popularity is now gained by deep learning (DL) approaches. DL methods are known for their ability to model complicated dependencies in data, much more efficiently than their shallow counterparts. In CADD, they are both used for evaluation of compounds activity and other properties (physicochemical, ADMET), as well as for the generation of molecules with properties falling in specific range of values (e.g., with defined solubility, stability, etc.) [38,39,40,41,42,43,44,45].

There are different approaches to estimate prediction uncertainty. At first, we would like to remark on the use of the soft-max probabilities in uncertainty estimation. It should be pointed out that measures calculated solely on the output of soft-max probability distribution are not actually modeling uncertainty. As shown by Gal et al. [46], the model can be *certain* (meaning high probability of class assignment) for a data point that was never seen by the model during training. Given a *perfect* classifier, for a sample out of the training distribution, but with some features resembling an specific subgroup of the training set (e.g., active compounds), we would like to predict the ligand active; however, with a measurable margin of uncertainty (as the model has not observed such an exact sample before). The certainty of such activity prediction is the expected outcome in the soft-max distribution, as it does not provide any additional information about its decision.

In this study, we used a method for uncertainty estimation proposed by Gal et al.—dropout-based uncertainty. It uses an indeterministic model both during training and evaluation. The stochasticity is expressed by the dropout mechanic [47], which was originally developed to combat overfitting of neural networks. In the original formulation, some of the network weights (i.e., neurons) are dropped out, zeroing their weighs, which in turn means that they do not contribute to the prediction. The set of neurons that are dropped out is different in each iteration (for each data batch, different neurons are dropped). In the typical dropout setting, none of the weights are dropped during evaluation, as we typically want the prediction to be deterministic.

Nevertheless, for the dropout-based uncertainty, the dropout *on* during inference is kept. Moreover, each testing sample is passed through the network multiple times, each with different dropout mask (i.e., different set of neurons dropped) and prediction statistics are calculated based on those outputs. Measuring the variance of each run for a given data point yields the model uncertainty.

We would like also to mention two other approaches for estimating model uncertainty. Bayesian neural networks are a popular framework for models with built-in uncertainty weights, with Probabilistic Backpropagation [48] as an example have already been used to estimate model uncertainty. Other approach, related to Bayesian models belongs to the group of Variational Inference methods, which provide an approximation to Bayesian inference over network’s weights [49]. The drawback of those methods is computational complexity, whereas the approach used in the study requires only few additional forwards passes through the model.

In the study, several types of experiments have been performed:

- the relationships between the prediction error, similarity to the training set and prediction uncertainty for the data from the test set were examined, together with analysis of correlation between uncertainty and the number of activity values provided—and also between uncertainty and standard deviation of activity values
- we tested whether incorporation of information about prediction uncertainty improves the compounds ranking on the basis of predicted activity
- uncertainty of predictions was used to search for the potential errors in the ChEMBL database.

The study was carried out for two sets of targets: 10 targets from previous benchmark experiments [35] and additional 15 targets from various G protein coupled receptors (GPCRs) families. The predictions (numerical regression of bioactivity of ligands) were carried out in two settings: random CV and balanced agglomerative clustering (BAC) for two compounds representations.

## 2. Results and Discussion

### 2.1. General Observations

Table 1 and Table 2 gather values of mean squared error (MSE) for CV and BAC splitting, together with the estimation of uncertainty.

The results gathered in Table 1 and Table 2 show that in general, MSE values were much higher for BAC splitting than random CV, which is related to increased task simplicity when compounds are divided into folds randomly [35]. In BAC splitting, the compounds from the test set are supposed to be structurally dissimilar to those that are present in the training set; therefore, via this approach we can evaluate the true ability of ML models to assess compounds covering broad chemical space. Nevertheless, using such an approach for ML methods evaluation is always related to worse performance, as CV-based output provides overoptimistic results (the compounds from the test set resemble examples from the training set, so the evaluation task is relatively simple) that are not reflected in real application of such models.

Interestingly, MSE values obtained for MACCSFP are higher in both CV and BAC splitting in comparison to hashed Morgan FP representation. The difference is higher for BAC splitting, but it is related to higher MSE values for these experiments.

Another consistent observation is that MSE is higher than dropout MSE, which means that the non-deterministic MSE is lower than its deterministic equivalent. It can be explained in such a way, that multiple drawing of dropout’s masks is identical to the prediction of the network committee, which usually is characterized by slightly better results.

Our last observation is connected with uncertainty evaluation. It also reaches higher values for BAC experiments in comparison to CV, while on the other hand, when different compound representations are compared, uncertainty values are higher for MACCSFP in comparison to Morgan FP.

When the results are examined from the target point of view, the highest error rate is observed for ACM_1_, mGluR_5_, ACM_1_ and ACM_2_ for both representations and splitting approaches and the lowest for D_2_ and MC_3_. In general, smaller datasets led to worse prediction efficiency.

### 2.2. Analysis of Uncertainties, Errors and Compound Similarities

**Figure 1:**
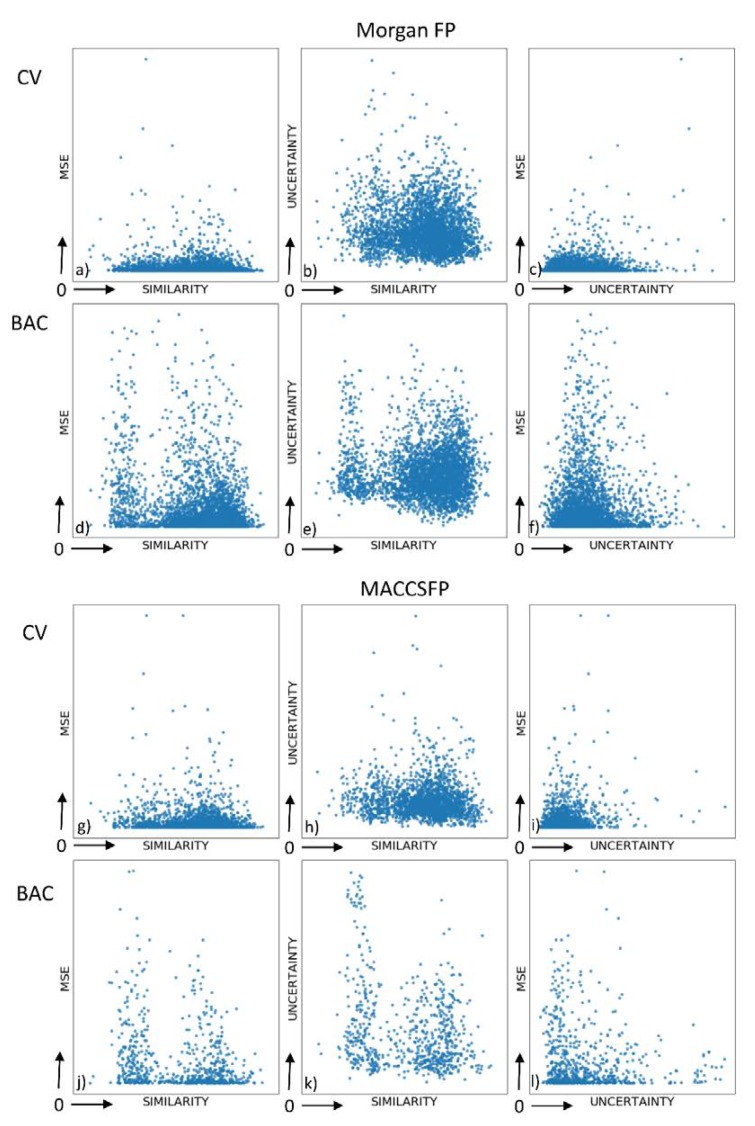
Visual analysis of dependencies between mean squared error (MSE), prediction uncertainty and compound similarity for adenosine A_1_ receptor. (**a**) MSE vs. similarity for Morgan FP in CV; (**b**) uncertainty vs. similarity for Morgan FP in CV (**c**) MSE vs. uncertainty for Morgan FP in CV; (**d**) MSE vs. similarity for Morgan FP in BAC; (**e**) uncertainty vs. similarity for Morgan FP in BAC; (**f**) MSE vs. uncertainty for Morgan FP in BAC; (**g**) MSE vs. similarity for MACCSFP in CV; (**h**) uncertainty vs. similarity for MACCSFP in CV (**i**) MSE vs. uncertainty for MACCSFP in CV; (**j**) MSE vs. similarity for MACCSFP in BAC; (**k**) uncertainty vs. similarity for MACCSFP in BAC; (**l**) MSE vs. uncertainty for MACCSFP in BAC.

The visualization of dependencies between the regression error, similarity to the training set (calculated for Morgan FP and with the use of Tanimoto coefficient) and uncertainty was performed (Figure 1, Supporting Information File S1).

The first observation coming from Figure 1 is that there is no significant difference between results obtained for two representation used—the only qualitative and strong indicated differentiation is the uncertainty vs. similarity dependence for random CV, which is spread over greater area for Morgan FP than MACCSFP. For this dependency it is also visible that the data points are placed differently when random CV vs. BAC splitting is considered—for random CV, the datapoints are concentrated closer to higher values of similarity coefficients, whereas for BAC they are spreading from the corner with lower similarity and uncertainty values. The highest concentration of datapoints in lower values of both parameters considered (MSE and uncertainty) is also observed for MACCSFP and random CV; for other combinations of dataset splitting approach and representation the datapoints cover much broader space of the respective chart although and MSE is more concentrated parameter than uncertainty, which adopts quite broad range of values. MSE vs. similarity charts also much more depended on the splitting approach than the compound representation and the highest concentration of points is shifted towards higher similarity values for random CV than for BAC.

**Figure 2:**
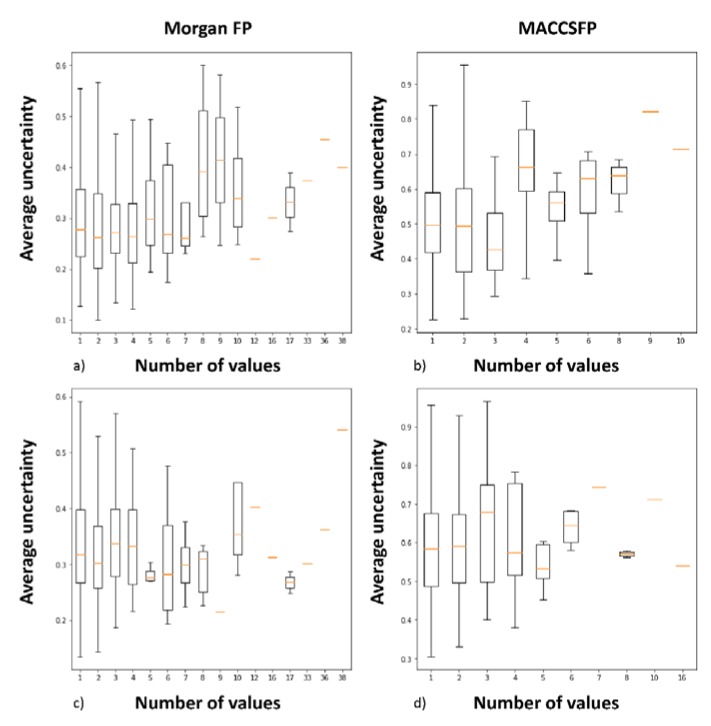
Analysis of dependency between prediction uncertainty and number of activity values provided for particular compound in the ChEMBL database for dopamine D_2_ ligands. (**a**) for Morgan FP in CV experiments; (**b**) for MACCFP in CV experiments; (**c**) for Morgan FP in BAC experiments; (**d**) for Morgan FP in BAC experiments.

Another type of analysis involved the examination of relationships between the number of different activity values reported and average prediction uncertainty (Figure 2, Supporting Information File S2). In the case, no ‘box’ is presented on the chart, only one compound was reported for particular number of activity values. Activity range is shown by lines, box size refers to first and last quartile and orange line to median activity value. The results show that there is no direct relationship between the number of activity values provided for particular compound and uncertainty of predictions obtained for them, for both random CV and BAC. No direct conclusion that increasing number of activity values reported led to higher uncertainty can be drawn.

**Figure 3:**
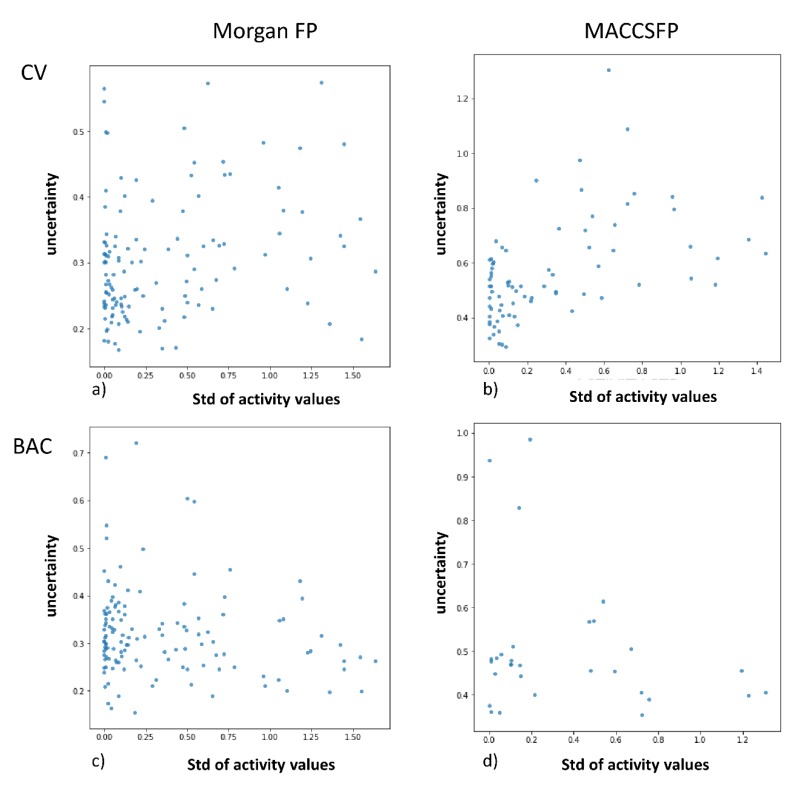
Analysis of dependency between prediction uncertainty and standard deviation of activity values provided for particular compound in the ChEMBL database for adenosine A_1_ ligands. (**a**) for Morgan FP in CV experiments; (**b**) for MACCFP in CV experiments; (**c**) for Morgan FP in BAC experiments; (**d**) for Morgan FP in BAC experiments.

Last type of analysis involved the examination of correlation between the standard deviation of activity values reported for given compound and prediction uncertainty (Figure 3, Supporting Information File S3).

**Figure 4:**
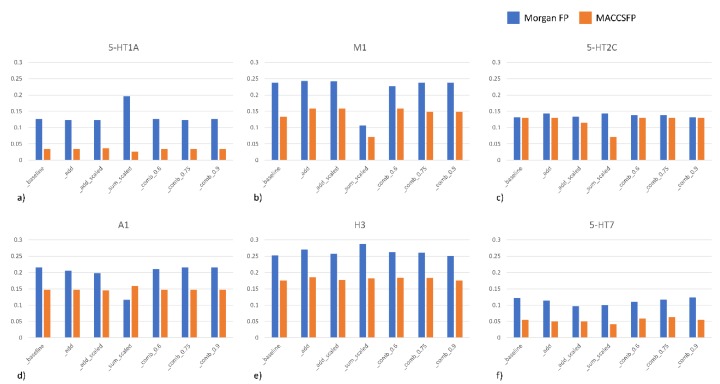
Precision at top 10% for various ranking strategies for selected targets. (**a**) 5-HT_1A_; (**b**) M_1_; (**c**) 5-HT_2C_; (**d**) A_1_; (**e**) H_3_; (**f**) 5-HT_7_.

The first observation coming from Figure 4 is that the consistency in data in training set (standard deviation of activity values equal to zero) is not related to clear and certain predictions for given compound and wide range of uncertainty is assigned to compounds with standard deviation of activity values equal to zero. Further, there is no correlation between standard deviation and uncertainty of predictions and even for compounds with wide range of activity values reported, predictions with low uncertainty can be obtained.

### 2.3. Compounds Ranking

In this experiment, we sorted compounds using particular strategy and the results were compared with the sorting based on true activities. The baseline model performs sorting only on the basis of prediction of a model, remaining strategies use also information about model uncertainty in various ways. It was indicated that using information about uncertainty in general improves sorting efficiency.

For the ranking strategies, we will assume that ${\hat{y}}_{i}$ is the predicted bioactivity (in terms of affinity values—K_i_) and u_i_ is the prediction uncertainty. We will denote $R\left({\hat{y}}_{i},u_{i}\right)$ as output of ranking function, meaning the lower the R value, the higher in our ranking the compound is.

The following ranking strategies were used:

- Baseline—only prediction of a model is taken into account
  (1) $$R\left({\hat{y}}_{i},u_{i}\right)={\hat{y}}_{i}$$
- Add—to model prediction, information about uncertainty is added directly; the less uncertain the model is about the sample, the better
  (2) $$R\left({\hat{y}}_{i},u_{i}\right)={\hat{y}}_{i}+u_{i}$$
- Scale—the uncertainty estimation is normalized to fit the range of [0,1]) and used to scale the prediction
  (3) $$R\left({\hat{y}}_{i},u_{i}\right)={\tilde{u}}_{i}*{\hat{\;y}}_{i}$$
  (4) $${\tilde{u}}_{i}=\frac{u_{i}-\underset{j}{min}u_{j}}{\underset{j}{max}u_{j}},$$
  where ${\tilde{u}}_{i}$ is a normalized uncertainty based on the measures for the whole test set.
- Add scaled—the uncertainty estimation is normalized to fit into [0,1] and added directly to the prediction
  (5) $$R\left({\hat{y}}_{i},u_{i}\right)={\tilde{u}}_{i}+{\hat{\;y}}_{i}$$
- Sum scaled—both prediction of a model and its uncertainty are normalized and then summed up
  $$R\left({\hat{y}}_{i},u_{i}\right)={\tilde{u}}_{i}+{\tilde{y}}_{i}\;{\tilde{y}}_{i}=\frac{y_{i}-\underset{j}{min}y_{j}}{\underset{j}{max}y_{j}}$$
  where ${\tilde{y}}_{i}$ is a normalized prediction based on the predictions over the whole dataset.
- Comb λ—linear combination of prediction and uncertainty with the λ coefficient (various λ values were tested).
  (6) $$R\left({\hat{y}}_{i},u_{i}\right)=\lambda {\hat{y}}_{i}+\left(1-\lambda \right)u_{i}$$

The results are presented in the form of the so-called “precision at top 10%”, which means that the compounds are sorted on the basis of their activity (two lists are prepared: based on true activity and based on the predicted values of activity parameters). Then, the 10% of top scored compounds from the list of the most active compounds is picked up and it is cross-checked with the list of the 10% of top scored compounds on the basis of predicted activity. The percentage of overlapping compounds between these two lists for different strategies for BAC splitting for example targets is gathered in Figure 4.

We can notice that the results and prediction effectiveness strongly depend both on the target, as well as compound representation. For all targets presented in Figure 4 Morgan FP appeared to be more effective representation. The differences in precision between different compound representations also vary for various targets—from slight differences for 5-HT_2C_, via a ~0.1 difference for M_1_, A_1_, H_3_ and 5-HT_7,_ to up to 0.15 difference for 5-HT_1A_. On the other hand, the differences related to various compound representations were higher than differences related to application of different approaches. When information on uncertainty is added, the precision values averaged over all targets considered revealed that for both compound representations _add approach was the best. Nevertheless, in both cases, the improvement in comparison to baseline was relatively low—0.004 and 0.007 for MorganFP and MACCSFP, respectively when averaged values for all targets are taken into account.

However, considering particular cases separately, there was an approach that improved the accuracy of ML predictions in comparison to baseline; there were examples where these improvement was quite significant. For example, for D_2_ and H_3_ ligands, precision at top 10% was higher by ~0.03 when _sum_scaled approach is compared to baseline (for Morgan FP). _sum_scaled approach gave the highest improvement over baseline for A_2A_, for MACCSFP representation (~0.04).

**Figure 5:**
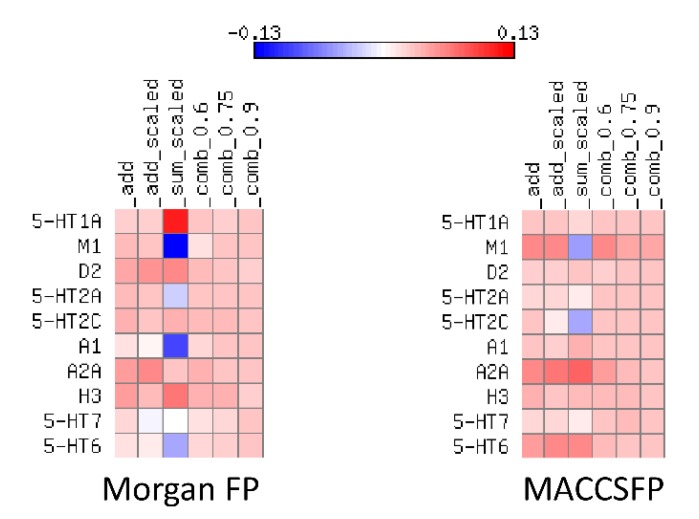
Heat maps presenting differences in precision at top 10% between approaches that take into account uncertainty and baseline for BAC splitting for 10 targets from benchmark studies.

In order to examine the influence of incorporation of uncertainty into ML models, the heat maps were prepared comparing the precision at top 10% for baseline and other approaches (Figure 5).

Figure 5 clearly show that in the majority of cases incorporation of information about prediction uncertainty improved efficiency of predictions of ML models (indicated by pink and red cells on heat maps). Nevertheless, the results strongly depend on target and compound representation. For M_1_, A_2A_ and 5-HT_6_, strong improvement is observed for MACCSFP; for Morgan FP, it was D_2_, A_2A_ and H_3_ that led to the highest improvement. This effect (for both representations is observed mainly for _add, _add_scaled and _sum_scaled approaches. On the other hand, the _sum_scaled approach was the only one for which the worsening of the results was observed for some targets (M_1_, A_1_ and 5-HT_6_ for Morgan FP and M_1_ and 5-HT_2C_ for MACCSFP).

### 2.4. Detection of Potential Errors in Bioactivity Databases

**Figure 6:**
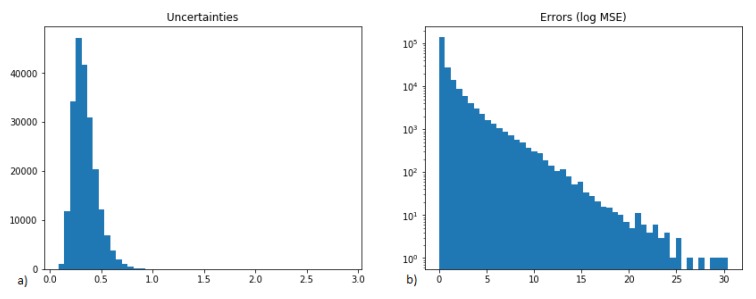
Analysis of uncertainty and MSE of activity prediction (calculated for all targets considered). (**a**) uncertainty; (**b**) prediction error expressed as log(MSE).

The developed methodology was used for detection of potential errors in the ChEMBL database. First of all, for all targets considered, the MSE of activity prediction together with uncertainties were calculated for the test set (Figure 6).

On the basis of these data, the ‘suspected’ data points were indicated; they were defined as those for which the MSE was in the 95 percentile (and higher) and uncertainty on the 5^th^ percentile (and higher).

**Figure 7:**
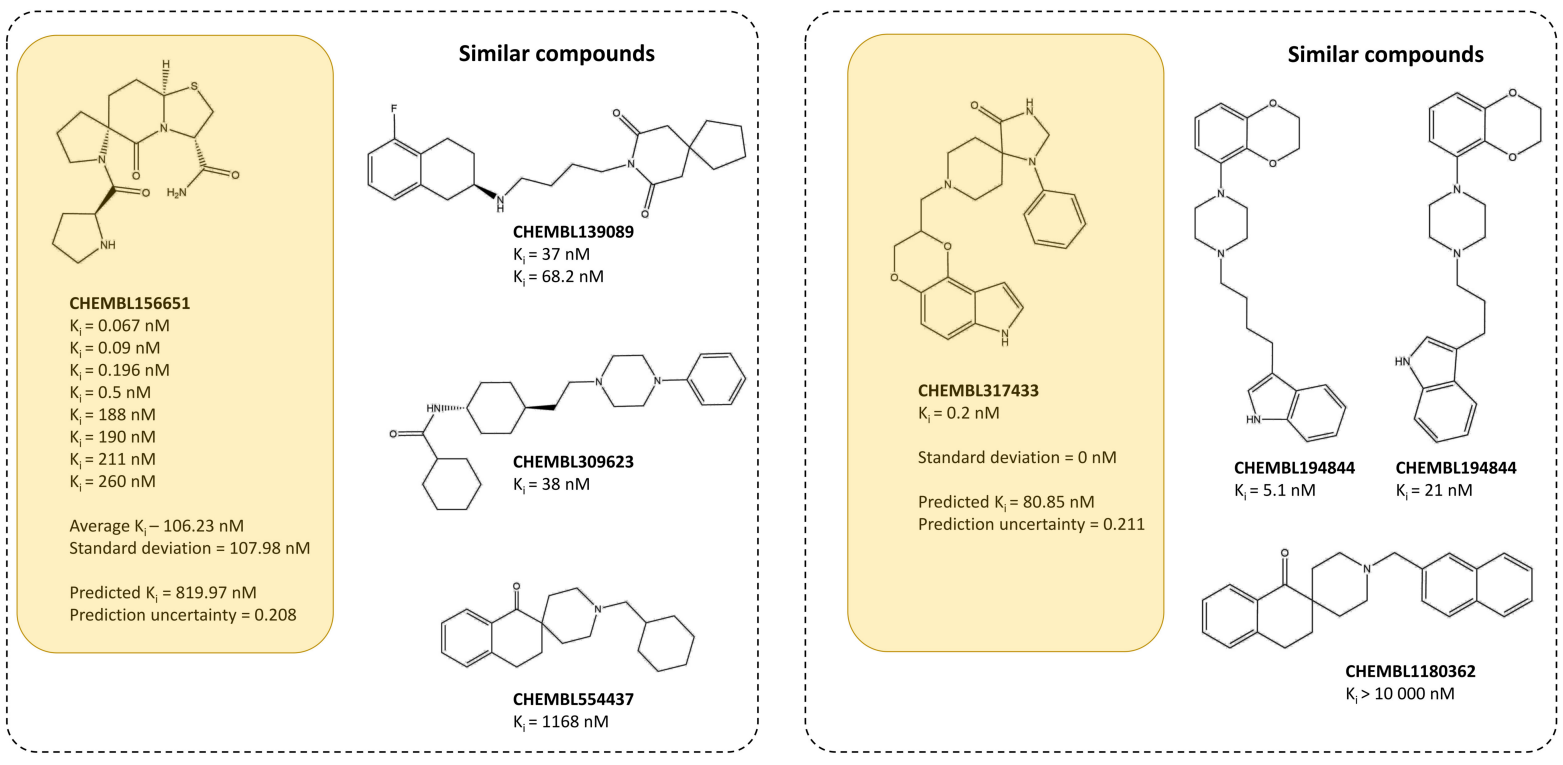
Analysis of selected dopamine D_2_ ligands with terms of affinity values provided in ChEMBL, predicted activity, prediction uncertainty and activity of structurally similar ligands (for Morgan FP).

Examples of such compounds—together with the true activities reported and activities of 10 most similar compounds—are presented below (Figure 7).

The provided examples of dopamine D_2_ ligands prove that the prediction uncertainty is not correlated with the standard deviation of activity values provided in activity databases. For ligand CHEMBL156651, there were 8 K_i_ values towards D_2_ receptor reported, ranging from 0.067 nM to 260 nM (with the average K_i_ equal to 106.23 nM) and standard deviation of 108 nM. The predicted K_i_ for this compound is however much lower than the actual affinity values and is equal to 11.11 nM which is expressed also by high uncertainty factor (0.708). The relatively low value of predicted K_i_ is a result of activities of similar compounds present in the dataset. Figure 7 presents only selected examples, but even among them can be found compounds with much lower K_i_ than actual average K_i_, such as CHEMBL139089, for which two K_i_ values are reported (37 and 68.2 nM). On the other hand, there is compound CHEMBL317433, for which only one K_i_ value is available (0.2 nM). Although for this compound, the standard deviation of K_i_ values is equal to zero, the prediction uncertainty is similar to the uncertainty determined for CHEMBL156651 (0.211) and the predicted K_i_ for this compound is equal to 80.85 nM. In this case, the activity of similar compounds is in narrower range, as the most active CHEMBL194844 has affinity of 5.1 nM, but there is also CHEMBL196171, whose K_i_ is equal to 21 nM.

## 3. Materials and Methods

The ChEMBL database [9] was used as a data source. Experiments were performed on 10 target proteins that were previously subject to detailed study in terms of dataset preparation for ML experiments [35]: serotonin receptors 5-HT_1A_, 5-HT_2A_, 5-HT_2C_, 5-HT_6_, 5-HT_7_ [50,51,52,53], muscarinic receptor ACM_1_ [54], adenosine receptors A_1_ [55] and A_2A_ [56,57], histamine receptor H_3_ [58] and dopamine receptor D_2_ [59,60]. The targets are mostly representatives of aminergic GPCRs and were selected due to the knowledge of ligands of these receptors and the datasets themselves due to previous studies performed on these targets [35]. In addition, 15 proteins covering also other GPCRs’ families were selected to minimize results bias related to target selection: bradykinin B1 receptor [61], melanocortin (MC) receptors subtype 3, 4 and 5 [62], kappa opioid receptor (KOR) [63,64], mu opioid receptor (MOR) [64], delta opioid receptor (DOR) [64] orexin receptors 1 and 2 (OX1R, OX2R) [65], cannabinoid CB_1_ receptor [66], cannabinoid CB_2_ receptor [66], melatonin receptors MT_1A_ and MT_1B_ [67], metabotropic glutamate receptor mGluR5 [68] and C-C chemokine receptor type 1 (CCR1) [69,70]. The respective datasets were extracted using the following protocol: all records referring to human-related data were gathered and all cases that were not describing binding data (activity parameter included in the list: K_i_, log(K_i_), pK_i_, IC_50_, log(IC_50_), pIC_50_) were filtered out. Then, only ‘equal to’ relation between activity parameter and its value were kept and units of activity parameters values should belong to the set {M, mM, µM, nM, pM, fM}; additional results were produced for extended set of relations between activity parameter and its value (“=”, “<”, “>”, “≤”, “≥”, “~”)—the results are presented in the Supporting Information and were obtained for the set of 10 targets from benchmark studies. Recalculation of IC_50_ into K_i_ was performed using the following formula: K_i_ = IC_50_/2 [35] Finally, the K_i_ values were converted to the logarithmic form and such datasets were used in the study.

The predictions were carried out in two settings: random CV and BAC [35]. The compounds structures were represented by Morgan Fingerprint (radius equal to 2) [71] and MACCSFP [72] calculated by RDKit [73]. The dataset sizes in these two splitting types are presented in Table 3.

**Scheme 1:**
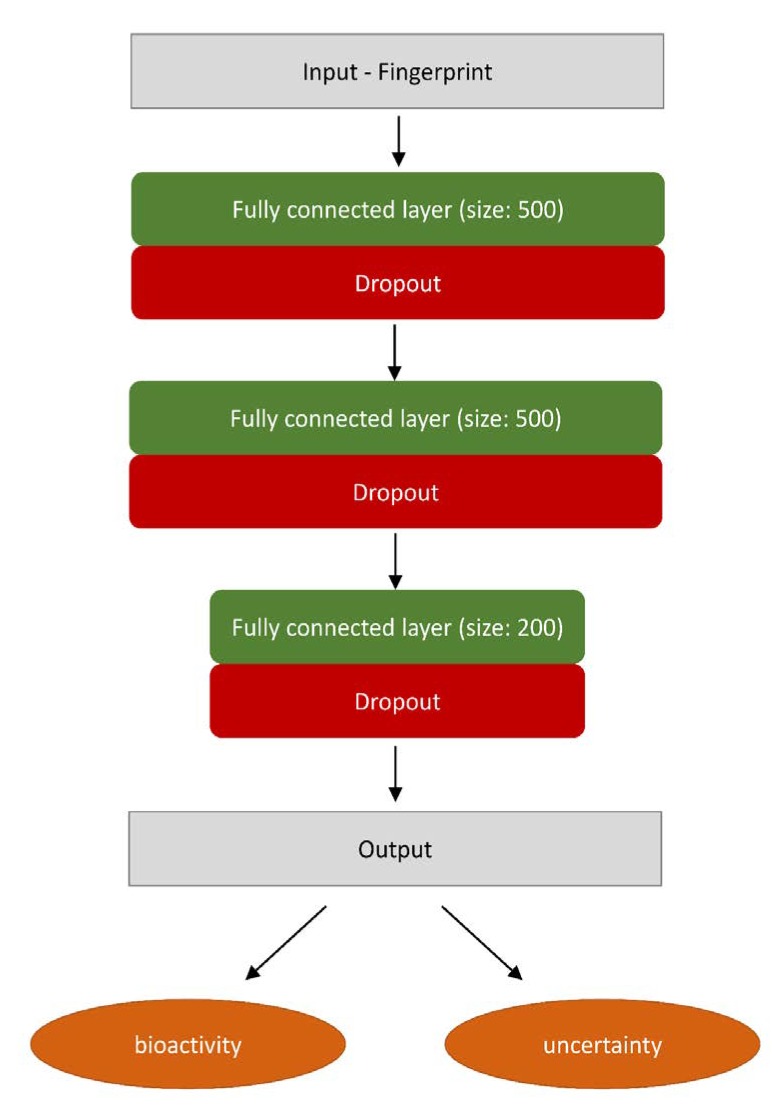
Neural network used in the study.

The problem considered in the study is numerical regression of bioactivity of ligands toward a particular target. For all of the experiments we used the same model architecture, a 3-hidden layer Multi-layer Perceptron with following hidden layer sizes: 500, 500, 200 and a single-neuron regression output layer. After each fully connected layer, except the final output layer, there was an ReLU nonlinearity activation function. Additionally, to enable uncertainty estimation, after each hidden layer, there is a *dropout* layer with probability of a neuron to be dropped set to 0.5 (Scheme 1), according to Gal et al. [46]. No additional regularization penalty were used throughout the training procedure. For the learning process, data points were supplied in mini batches of 100 examples, Adam method [74] was used for the optimizer with learning rate set to 0.001, each model was trained for 200 epochs. For each protein-representation pair a 5-fold cross-validation scheme was performed using two different splitting strategies mentioned in the earlier (random CV, BAC). The model, the training procedure as well as the uncertainty estimation algorithm was implemented using DeepChem package [75]. If a specific hyperparameter value is not mentioned, the default value provided by DeepChem was used.

## 4. Conclusions

In the study, we presented a DL-based approach for examination of uncertainty of compounds activity prediction. Several approaches were considered and their influence on activity prediction by ML methods was examined. Extended examination of the relationships between prediction uncertainty and compound similarity, number of activity values provided, and their standard deviation was carried out. We developed several dropout-based approaches for estimation of prediction uncertainty and applied uncertainty analysis for detection of potential errors in the ChEMBL database. The developed methodology can be of great help during virtual screening experiments, as information about prediction uncertainty for compounds indicated as potentially active might have crucial impact on making decision about their purchase.

## Figures and Tables

**Table 1:** Regression results obtained for random CV.

Target	Morgan			MACCSFP		
	MSE	Dropout MSE	Uncertainty	MSE	Dropout MSE	Uncertainty
5-HT_1A_	0.416 ± 0.02	0.406 ± 0.02	0.318 ± 0.01	0.568 ± 0.04	0.552 ± 0.04	0.580 ± 0.00
ACM_1_	0.665 ± 0.18	0.660 ± 0.17	0.352 ± 0.01	0.781 ± 0.16	0.763 ± 0.15	0.567 ± 0.01
D_2_	0.352 ± 0.03	0.344 ± 0.03	0.298 ± 0.01	0.436 ± 0.00	0.420 ± 0.00	0.510 ± 0.00
5-HT_2A_	0.467 ± 0.03	0.459 ± 0.03	0.319 ± 0.01	0.559 ± 0.06	0.551 ± 0.05	0.523 ± 0.01
5-HT_2C_	0.494 ± 0.02	0.488 ± 0.02	0.304 ± 0.01	0.532 ± 0.03	0.518 ± 0.02	0.535 ± 0.04
A_1_	0.429 ± 0.03	0.421 ± 0.03	0.308 ± 0.00	0.479 ± 0.02	0.474 ± 0.02	0.547 ± 0.03
A_2A_	0.412 ± 0.03	0.406 ± 0.03	0.326 ± 0.01	0.528 ± 0.03	0.515 ± 0.02	0.561 ± 0.02
H_3_	0.407 ± 0.04	0.399 ± 0.04	0.300 ± 0.01	0.504 ± 0.02	0.488 ± 0.002	0.518 ± 0.02
5-HT_7_	0.455 ± 0.04	0.450 ± 0.03	0.302 ± 0.02	0.899 ± 0.04	0.902 ± 0.03	0.523 ± 0.02
5-HT_6_	0.461 ± 0.03	0.455 ± 0.02	0.309 ± 0.00	0.520 ± 0.00	0.507 ± 0.00	0.544 ± 0.01
MT_1A_	0.630 ± 0.12	0.622 ± 0.13	0.365 ± 0.02	0.769 ± 0.10	0.761 ± 0.10	0.596 ± 0.03
MT_1B_	0.630 ± 0.06	0.623 ± 0.05	0.351 ± 0.02	0.856 ± 0.13	0.839 ± 0.12	0.586 ± 0.02
CB_1_	0.494 ± 0.05	0.486 ± 0.05	0.343 ± 0.01	0.584 ± 0.03	0.578 ± 0.03	0.565 ± 0.03
MOR	0.538 ± 0.06	0.528 ± 0.06	0.380 ± 0.01	0.663 ± 0.06	0.643 ± 0.06	0.645 ± 0.02
DOR	0.473 ± 0.01	0.466 ± 0.01	0.380 ± 0.00	0.614 ± 0.03	0.605 ± 0.03	0.636 ± 0.01
KOR	0.513 ± 0.04	0.500 ± 0.04	0.375 ± 0.01	0.651 ± 0.04	0.636 ± 0.04	0.629 ± 0.03
CB_2_	0.542 ± 0.03	0.525 ± 0.03	0.343 ± 0.01	0.641 ± 0.03	0.627 ± 0.03	0.601 ± 0.02
MC_4_	0.407 ± 0.04	0.396 ± 0.04	0.361 ± 0.00	0.540 ± 0.03	0.526 ± 0.04	0.589 ± 0.01
mGluR5	0.628 ± 0.11	0.627 ± 0.11	0.332 ± 0.02	0.720 ± 0.09	0.713 ± 0.09	0.524 ± 0.03
CCR2	0.417 ± 0.08	0.409 ± 0.08	0.285 ± 0.02	0.546 ± 0.20	0.539 ± 0.20	0.534 ± 0.03
B1	0.578 ± 0.15	0.576 ± 0.16	0.320 ± 0.03	0.722 ± 0.18	0.722 ± 0.17	0.618 ± 0.04
MC_5_	0.484 ± 0.11	0.471 ± 0.10	0.433 ± 0.03	0.428 ± 0.11	0.423 ± 0.10	0.578 ± 0.04
MC_3_	0.339 ± 0.06	0.343 ± 0.06	0.413 ± 0.02	0.422 ± 0.09	0.418 ± 0.08	0.542 ± 0.02
OX_2_R	0.445 ± 0.04	0.439 ± 0.04	0.307 ± 0.01	0.562 ± 0.06	0.550 ± 0.06	0.599 ± 0.01
OX_1_R	0.340 ± 0.04	0.336 ± 0.03	0.325 ± 0.01	0.510 ± 0.05	0.500 ± 0.05	0.570 ± 0.01

**Table 2:** Regression results obtained for BAC.

Target	Morgan FP			MACCSFP		
	MSE	Dropout MSE	Uncertainty	MSE	Dropout MSE	Uncertainty
5-HT_1A_	1.323 ± 0.21	1.284 ± 0.18	0.347 ± 0.04	1.879 ± 0.10	1.746 ± 0.03	0.563 ± 0.07
ACM_1_	1.788 ± 0.43	1.766 ± 0.41	0.389 ± 0.07	2.792 ± 0.80	2.706 ± 0.73	0.594 ± 0.09
D_2_	0.967 ± 0.16	0.954 ± 0.16	0.342 ± 0.02	1.424 ± 0.26	1.304 ± 0.15	0.588 ± 0.04
5-HT_2A_	1.667 ± 0.56	1.609 ± 0.57	0.395 ± 0.04	1.496 ± 0.00	1.449 ± 0.00	0.568 ± 0.00
5-HT_2C_	1.534 ± 0.75	1.502 ± 0.72	0.338 ± 0.04	1.714 ± 0.44	1.671 ± 0.44	0.517 ± 0.01
A_1_	1.378 ± 0.56	1.359 ± 0.56	0.337 ± 0.03	1.602 ± 0.00	1.537 ± 0.00	0.476 ± 0.00
A_2A_	1.445 ± 0.37	1.430 ± 0.36	0.367 ± 0.03	1.264 ± 0.19	1.207 ± 0.19	0.567 ± 0.02
H_3_	1.001 ± 0.13	0.961 ± 0.13	0.354 ± 0.02	1.284 ± 0.46	1.205 ± 0.43	0.547 ± 0.05
5-HT_7_	1.267 ± 0.53	1.252 ± 0.54	0.316 ± 0.03	2.027 ± 0.65	1.956 ± 0.63	0.534 ± 0.04
5-HT_6_	1.337 ± 0.39	1.305 ± 0.38	0.323 ± 0.03	1.597 ± 0.41	1.453 ± 0.44	0.564 ± 0.03
MT_1A_	1.989 ± 0.54	1.989 ± 0.55	0.367 ± 0.06	2.101 ± 0.60	2.071 ± 0.59	0.532 ± 0.04
MT_1B_	1.921 ± 0.27	1.914 ± 0.28	0.340 ± 0.06	1.925 ± 0.42	1.899 ± 0.43	0.565 ± 0.06
CB_1_	1.413 ± 0.44	1.399 ± 0.41	0.351 ± 0.03	1.595 ± 0.49	1.511 ± 0.43	0.563 ± 0.02
MOR	1.602 ± 0.30	1.546 ± 0.30	0.405 ± 0.02	1.994 ± 0.38	1.878 ± 0.38	0.653 ± 0.05
DOR	1.921 ± 1.01	1.895 ± 0.99	0.360 ± 0.02	2.393 ± 1.07	2.251 ± 0.94	0.607 ± 0.05
KOR	1.653 ± 0.43	1.613 ± 0.42	0.401 ± 0.03	2.159 ± 0.86	2.052 ± 0.79	0.651 ± 0.05
CB_2_	1.601 ± 0.41	1.577 ± 0.43	0.350 ± 0.02	1.552 ± 0.41	1.508 ± 0.38	0.619 ± 0.08
MC_4_	1.652 ± 0.87	1.561 ± 0.78	0.392 ± 0.07	1.406 ± 0.49	1.371 ± 0.48	0.577 ± 0.07
mGluR5	2.289 ± 1.08	2.279 ± 1.08	0.336 ± 0.05	1.844 ± 0.71	1.839 ± 0.73	0.488 ± 0.07
CCR2	0.792 ± 0.43	0.789 ± 0.43	0.288 ± 0.05	1.480 ± 0.60	1.428 ± 0.57	0.411 ± 0.04
B1	1.903 ± 0.65	1.894 ± 0.66	0.353 ± 0.05	1.503 ± 0.42	1.478 ± 0.42	0.597 ± 0.16
MC_5_	1.221 ± 1.12	1.237 ± 1.13	0.441 ± 0.04	1.230 ± 1.35	1.207 ± 1.31	0.504 ± 0.02
MC_3_	0.947 ± 0.41	0.913 ± 0.39	0.482 ± 0.05	0.917 ± 0.43	0.895 ± 0.42	0.533 ± 0.05
OX_2_R	1.192 ± 0.64	1.183 ± 0.63	0.291 ± 0.02	1.573 ± 0.84	1.582 ± 0.87	0.547 ± 0.05
OX_1_R	1.178 ± 0.57	1.164 ± 0.56	0.345 ± 0.05	1.833 ± 0.14	1.778 ± 0.13	0.569 ± 0.08

**Table 3:** Size of datasets used in the study.

Target CHEMBLID	Target Name	Training Set Size CV	Test Set Size CV	Training Set Size BAC	Test Set Size BAC
CHEMBL214	5-HT_1A_	2599	649	2495	753
CHEMBL216	ACM_1_	676	168	652	192
CHEMBL217	D_2_	4496	1124	4243	1379
CHEMBL224	5-HT_2A_	2385	596	2025	956
CHEMBL225	5-HT_2C_	1559	389	1590	358
CHEMBL226	Adenosine A_1_	2782	695	2758	719
CHEMBL251	Adenosine A_2A_	3165	791	2915	1041
CHEMBL264	Histamine H_3_	2546	636	2689	493
CHEMBL3155	5-HT_7_	1209	302	1070	441
CHEMBL3371	5-HT_6_	2074	518	1979	613
CHEMBL1945	MT _1A_	573	143	622	94
CHEMBL1946	MT _1B_	572	142	524	190
CHEMBL218	CB_1_	1945	486	1950	481
CHEMBL233	MOR	2815	703	2997	521
CHEMBL236	DOR	2457	614	2070	1001
CHEMBL237	KOR	2381	595	2147	829
CHEMBL253	CB_2_	2611	652	2687	576
CHEMBL259	MC_4_	1450	362	1364	448
CHEMBL3227	mGluR5	272	67	283	56
CHEMBL4015	CCR2	168	42	180	30
CHEMBL4308	B1	444	110	431	123
CHEMBL4608	MC_5_	314	78	334	58
CHEMBL4644	MC_3_	400	99	410	89
CHEMBL4792	OX_2_R	1269	317	1175	411
CHEMBL5113	OX_1_R	1087	271	1182	176

## Supplementary Materials

The following are available online. File S1. Visual analysis of dependencies between MSE, prediction uncertainty and compound similarity for all targets considered in the study for only ‘=’ relation between activity parameter and its value; File S2. Analysis of dependency between prediction uncertainty and number of activity values provided for particular compound in the ChEMBL database for all targets considered in the study for only ‘=’ relation between activity parameter and its value; File S3. Analysis of dependency between prediction uncertainty and standard deviation of activity values provided for particular compound in the ChEMBL database for all targets considered in the study for only ‘=’ relation between activity parameter and its value. File S4. Visual analysis of dependencies between MSE, prediction uncertainty and compound similarity for benchmark targets for extended set of relations between activity parameter and its value. File S5. Analysis of dependency between prediction uncertainty and number of activity values provided for particular compound in the ChEMBL database for benchmark targets for extended set of relations between activity parameter and its value; File S6. Analysis of dependency between prediction uncertainty and standard deviation of activity values provided for particular compound in the ChEMBL database for benchmark targets for extended set of relations between activity parameter and its value.
