# Supplementary material for: How Sure Can We Be about ML Methods-Based Evaluation of Compound Activity: Incorporation of Information about Prediction Uncertainty Using Deep Learning Techniques
Source: Molecules. 2020 Mar 23;25(6):1452. doi: 10.3390/molecules25061452 (PMC7144469; doi:10.3390/molecules25061452)
Supplement: Supplementary file 1 [file molecules-25-01452-s001.zip › Supp_Info_for_submission/FileS1.pdf]

Morgan FP
CHEMBL214

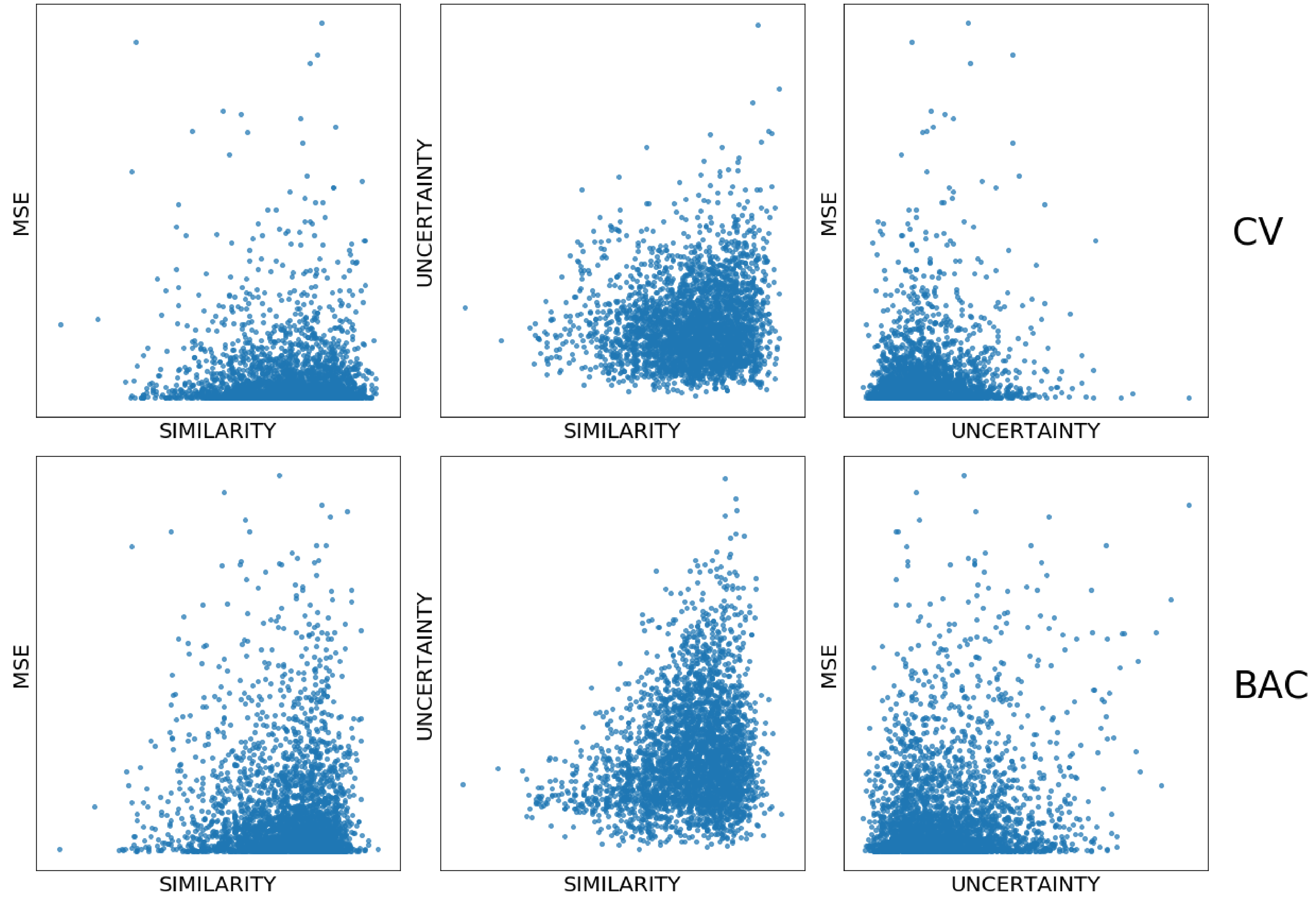

Morgan FP
CHEMBL216

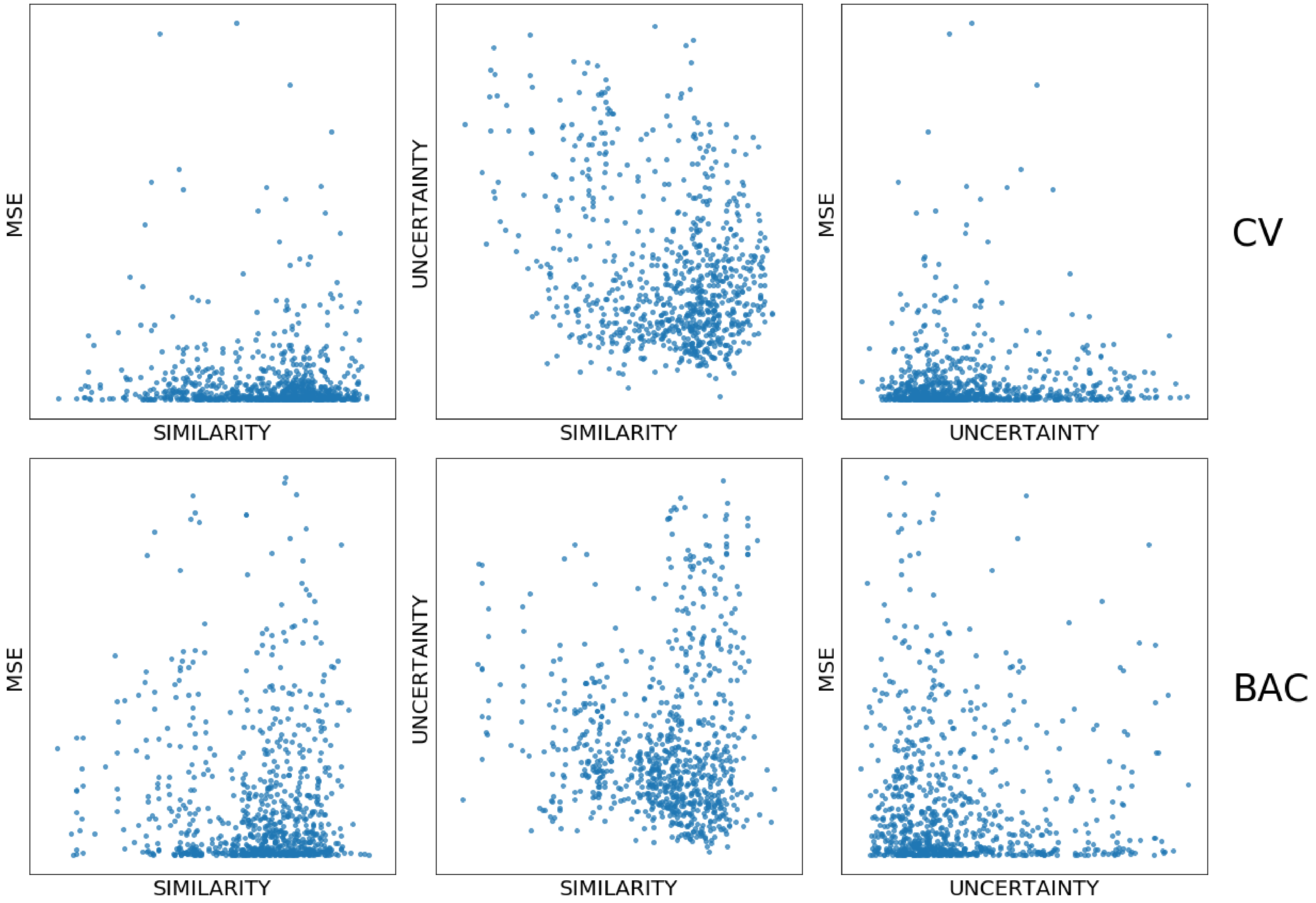

Morgan FP
CHEMBL217

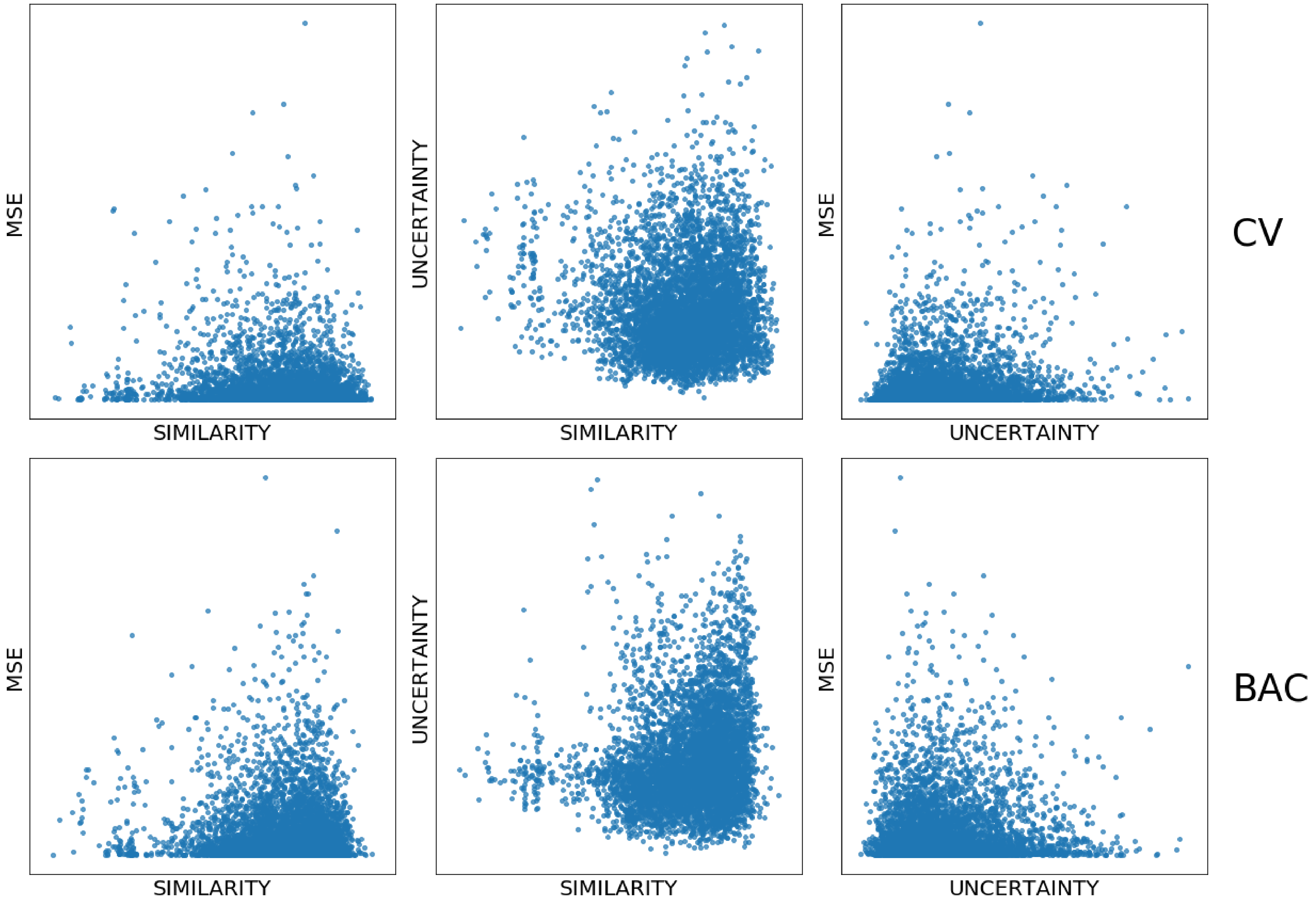

Morgan FP
CHEMBL224

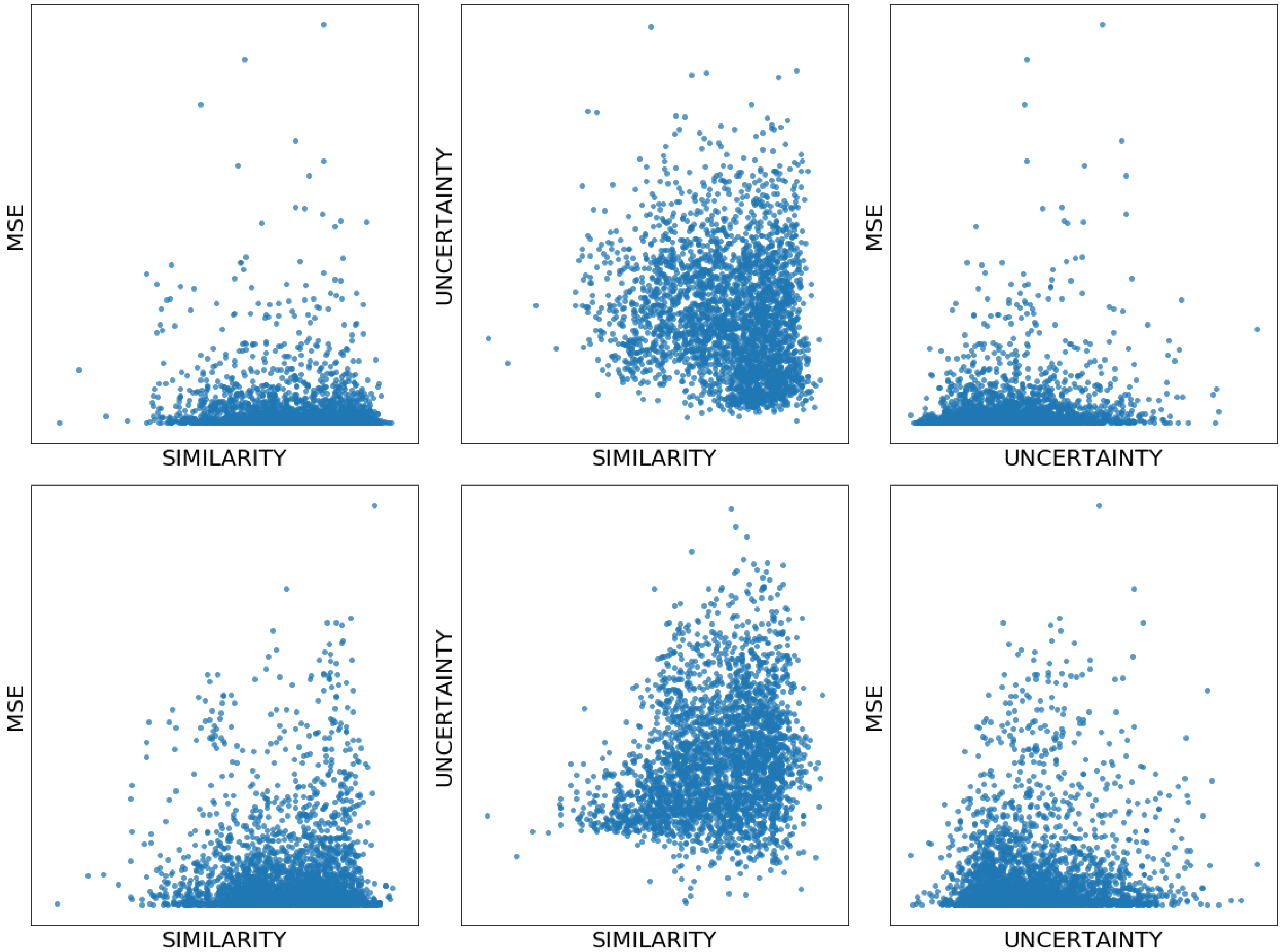

CV

BAC

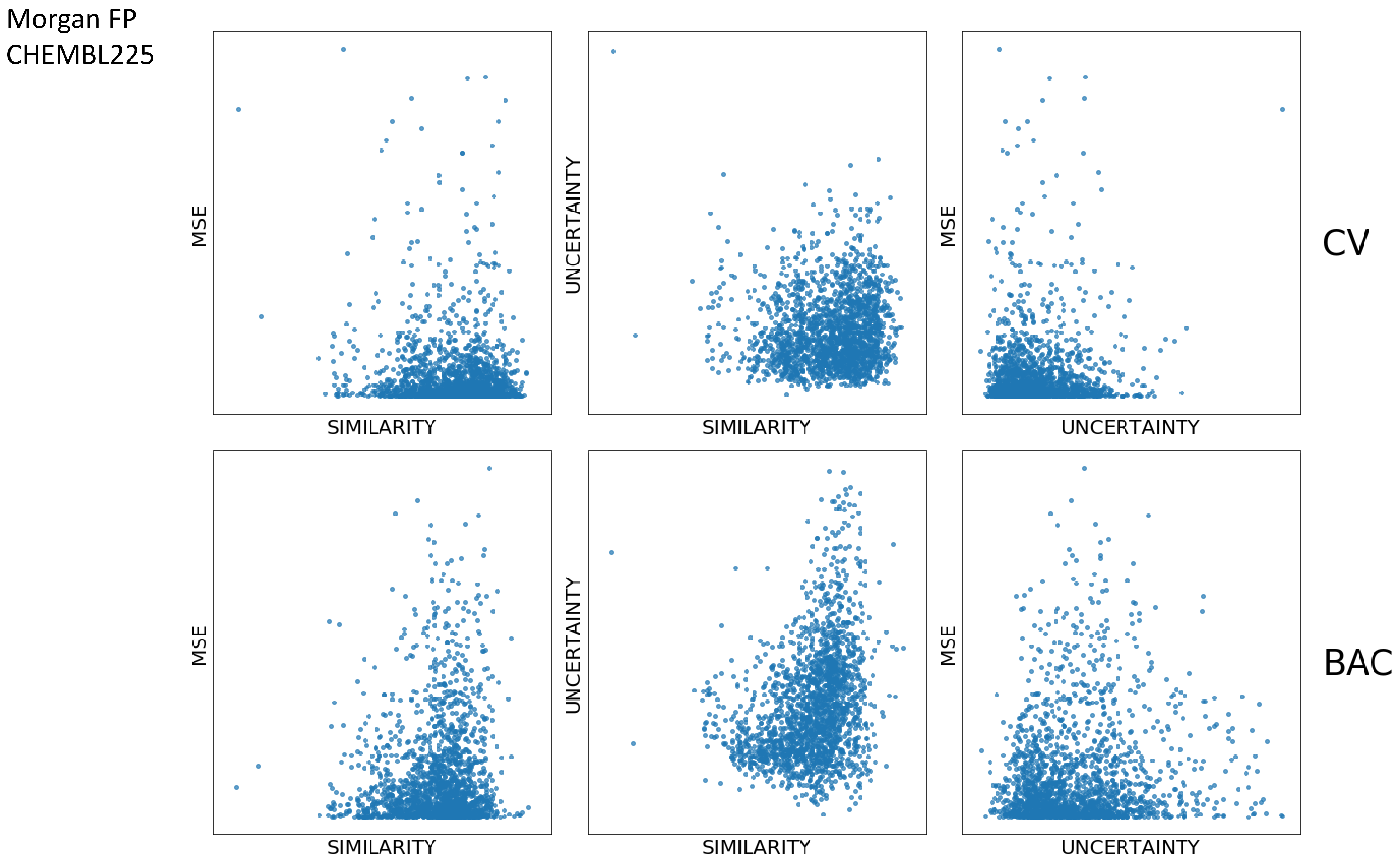
Morgan FP
CHEMBL225
MSE
SIMILARITY
UNCERTAINTY
SIMILARITY
MSE
UNCERTAINTY
CV
MSE
SIMILARITY
UNCERTAINTY
SIMILARITY
MSE
UNCERTAINTY
BAC

Morgan FP
CHEMBL226

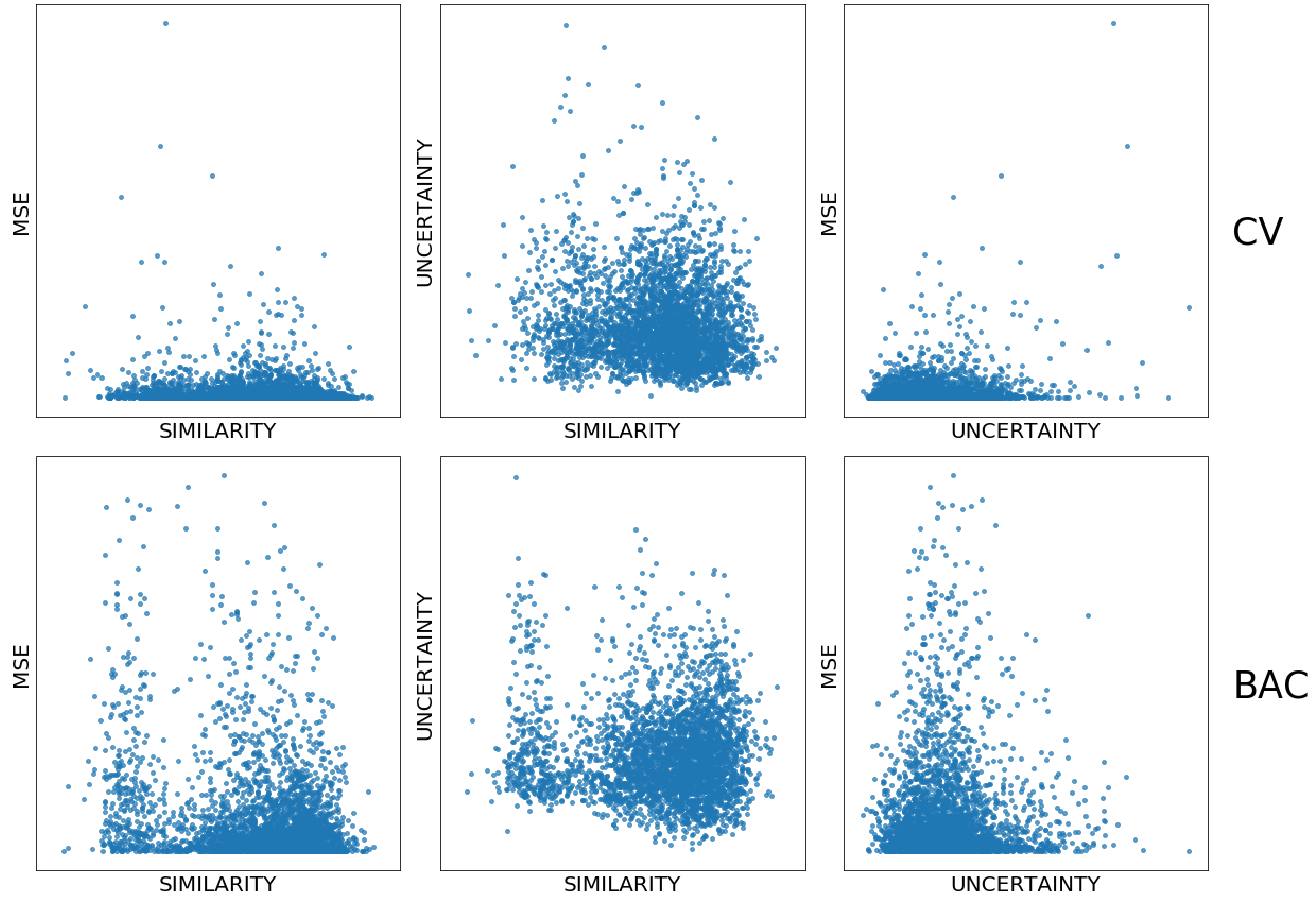

Morgan FP
CHEMBL251

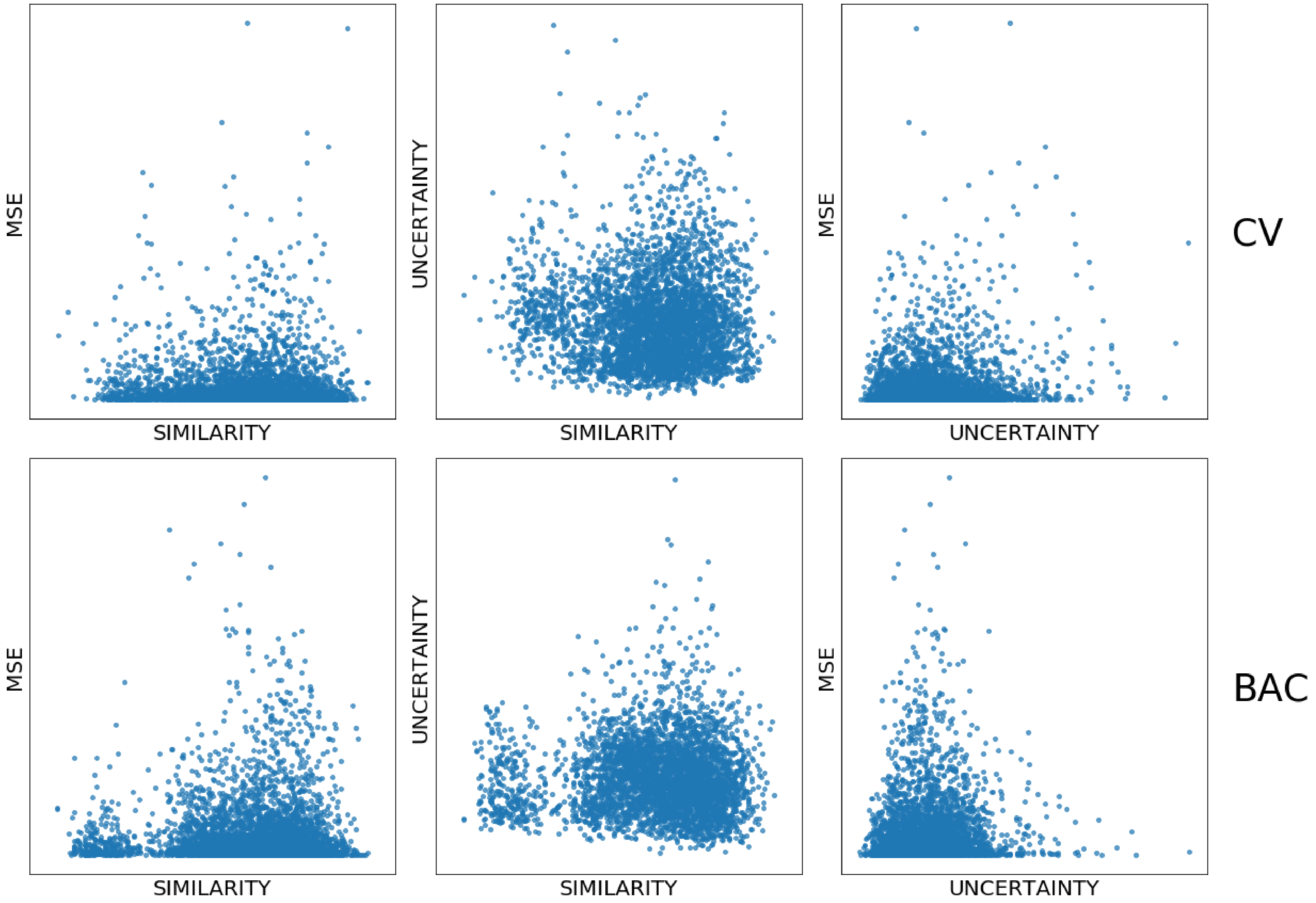

Morgan FP
CHEMBL264

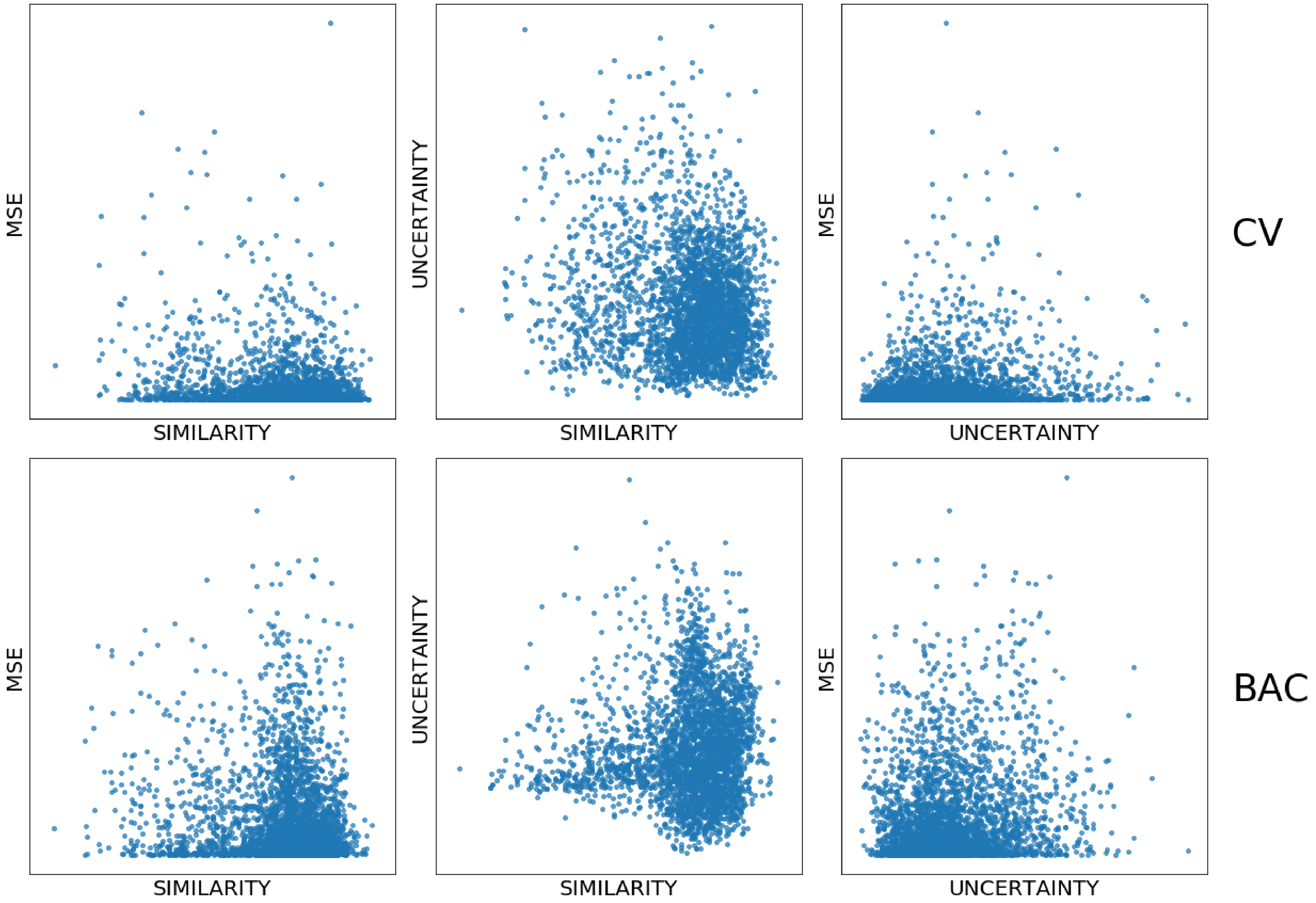

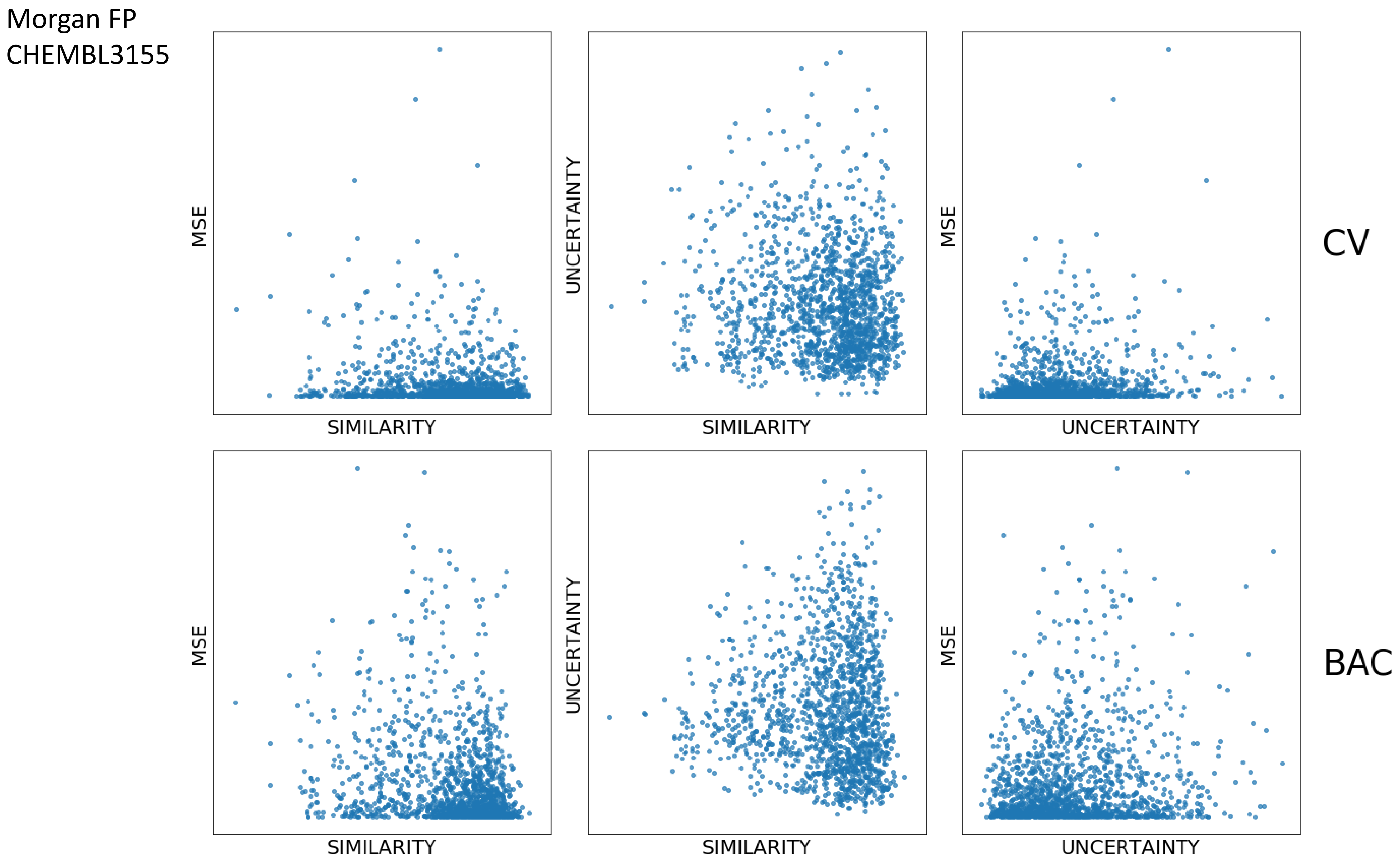
Morgan FP
CHEMBL3155
MSE
SIMILARITY
UNCERTAINTY
SIMILARITY
MSE
UNCERTAINTY
CV
MSE
SIMILARITY
UNCERTAINTY
SIMILARITY
MSE
UNCERTAINTY
BAC

Morgan FP
CHEMBL3371

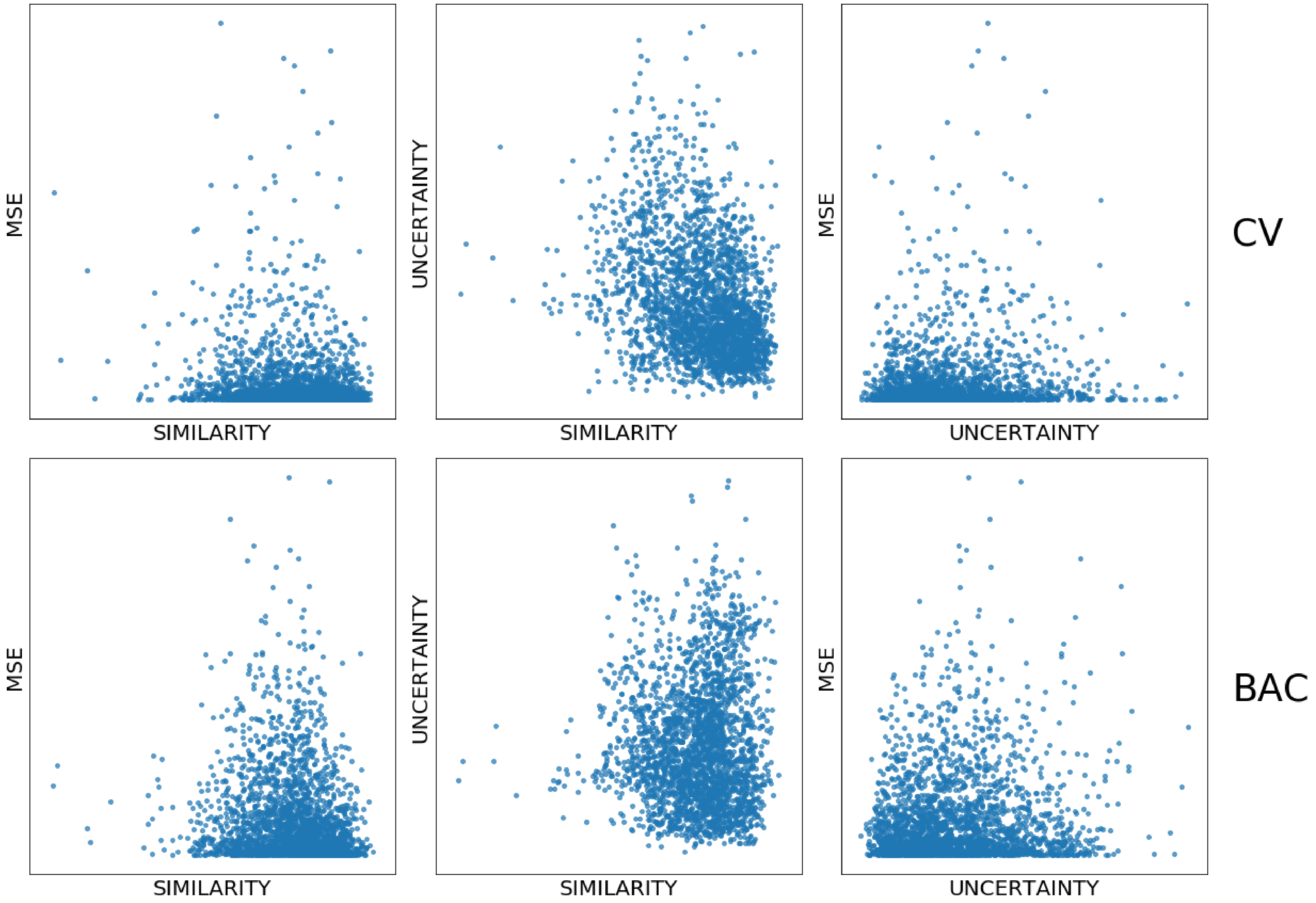

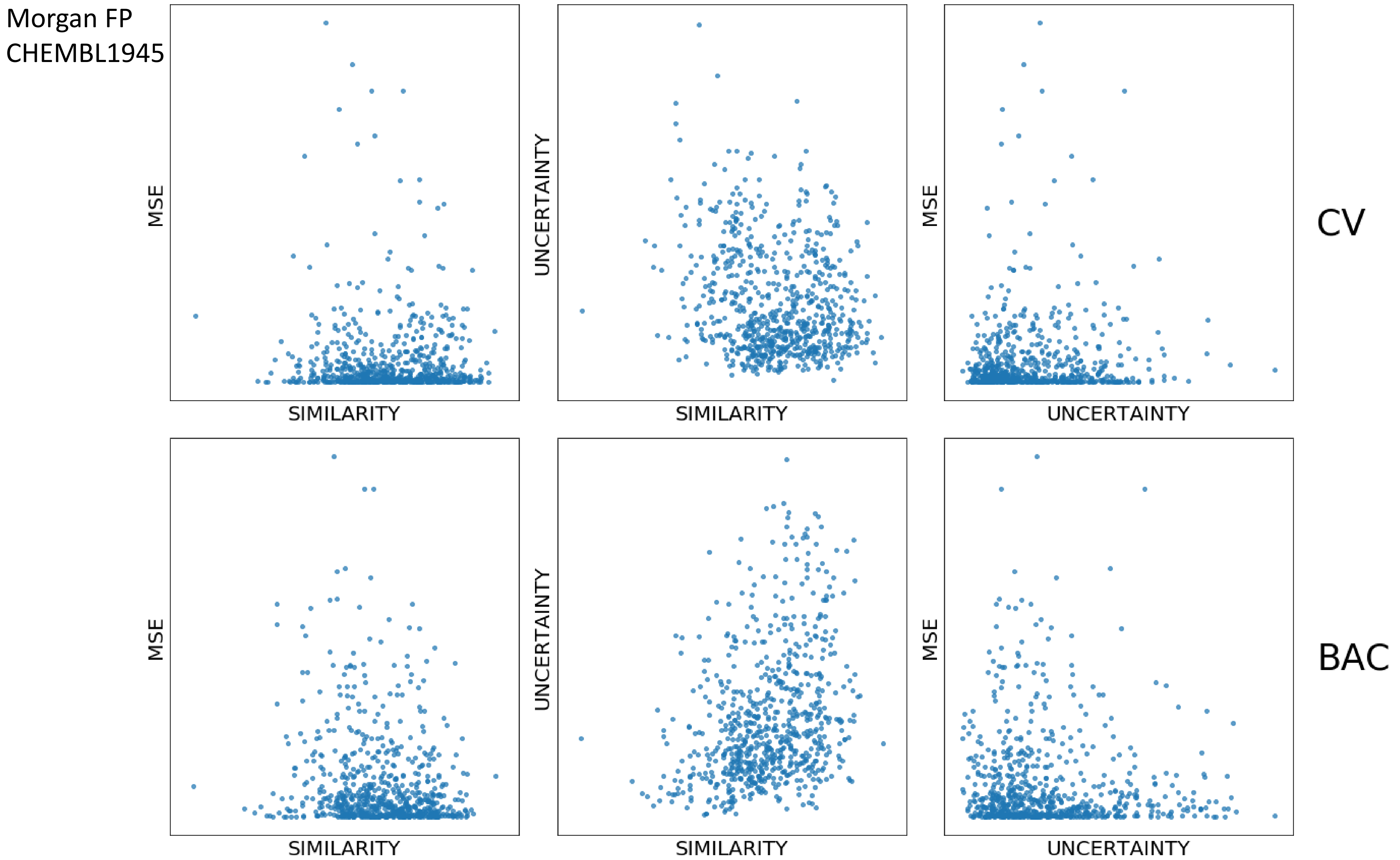
Morgan FP
CHEMBL1945
MSE
SIMILARITY
UNCERTAINTY
SIMILARITY
MSE
UNCERTAINTY
CV
MSE
SIMILARITY
UNCERTAINTY
SIMILARITY
MSE
UNCERTAINTY
BAC

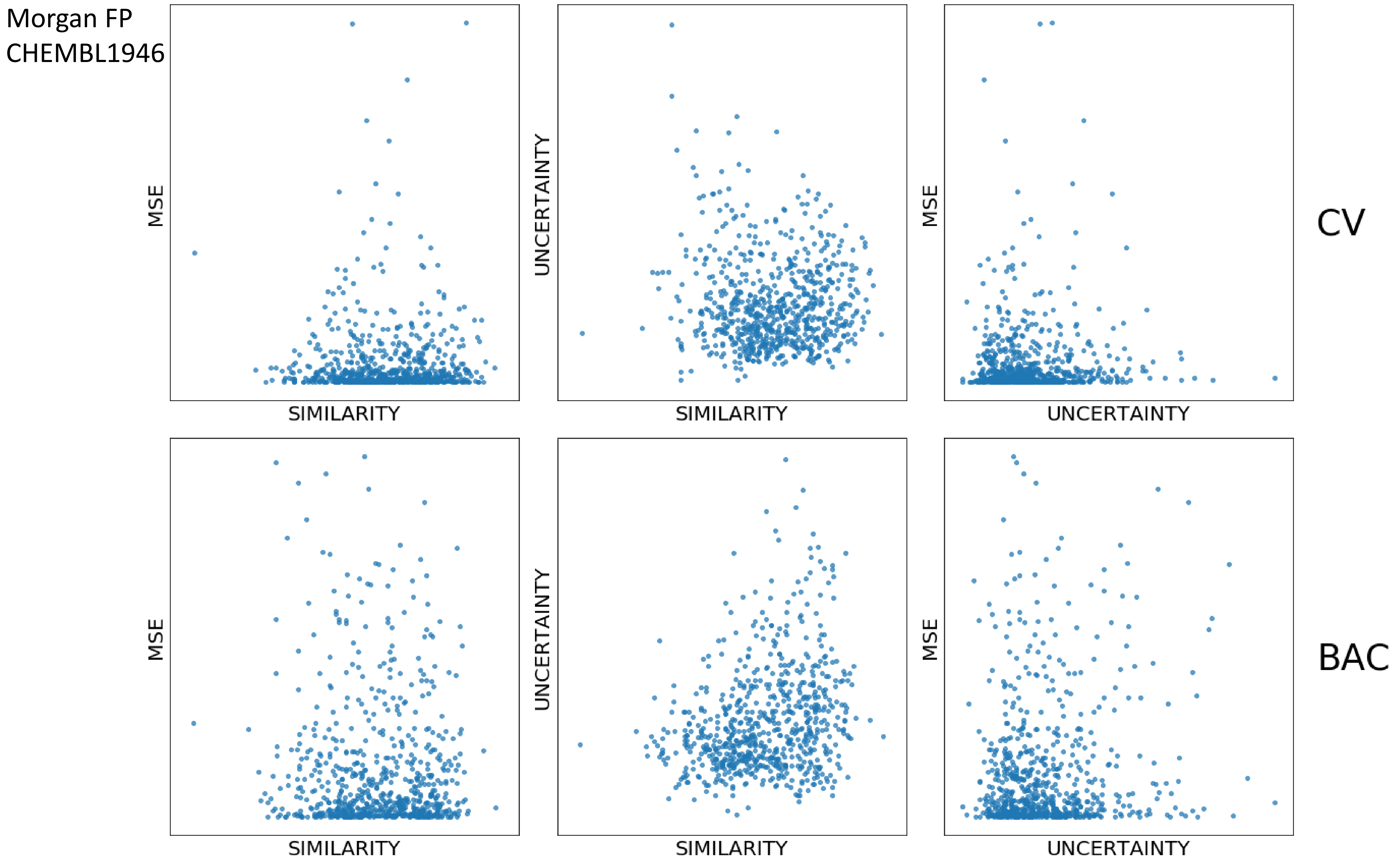
Morgan FP
CHEMBL1946
MSE
SIMILARITY
UNCERTAINTY
SIMILARITY
MSE
UNCERTAINTY
CV
MSE
SIMILARITY
UNCERTAINTY
SIMILARITY
MSE
UNCERTAINTY
BAC

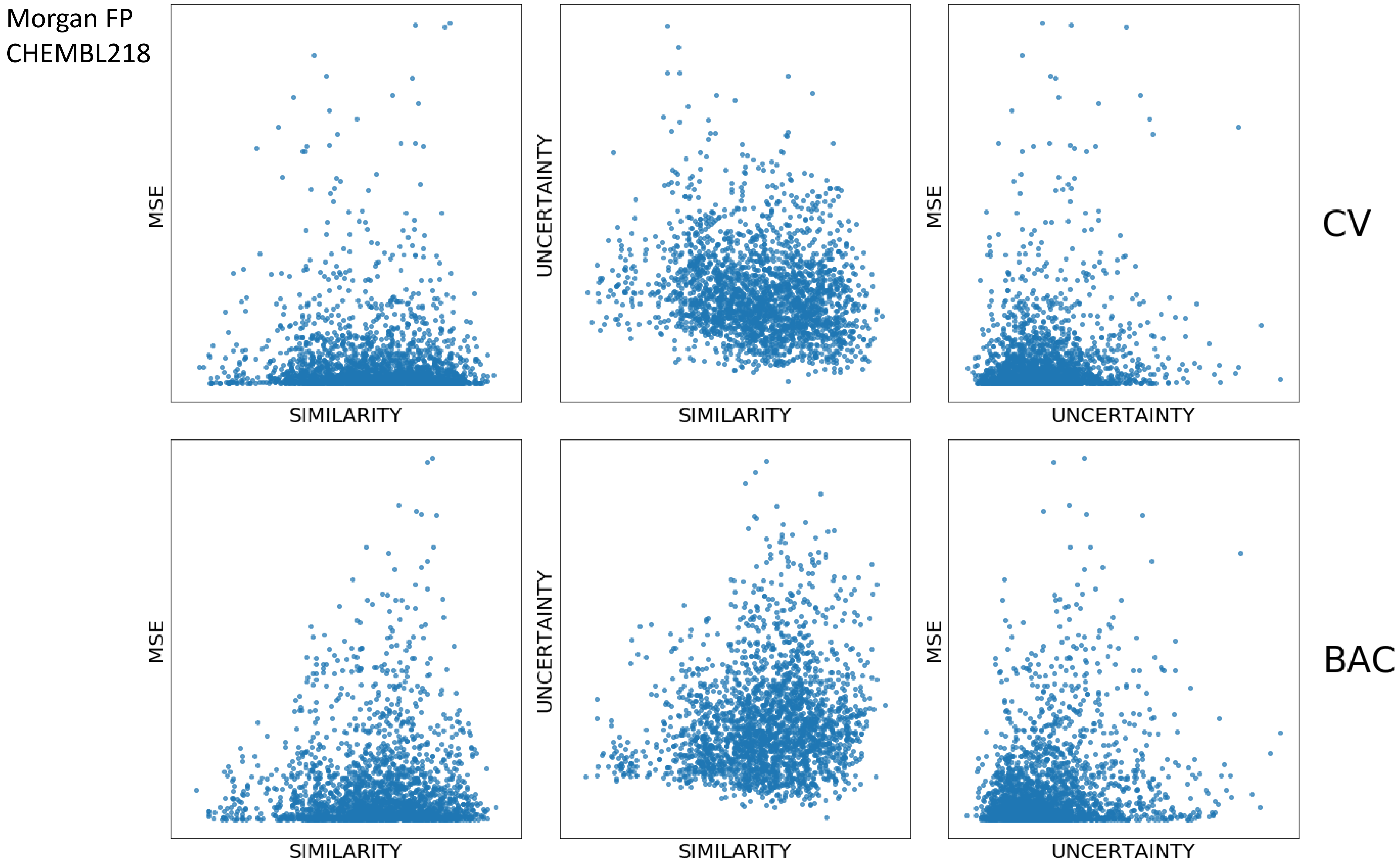
Morgan FP
CHEMBL218
MSE
SIMILARITY
UNCERTAINTY
SIMILARITY
MSE
UNCERTAINTY
CV
MSE
SIMILARITY
UNCERTAINTY
SIMILARITY
MSE
UNCERTAINTY
BAC

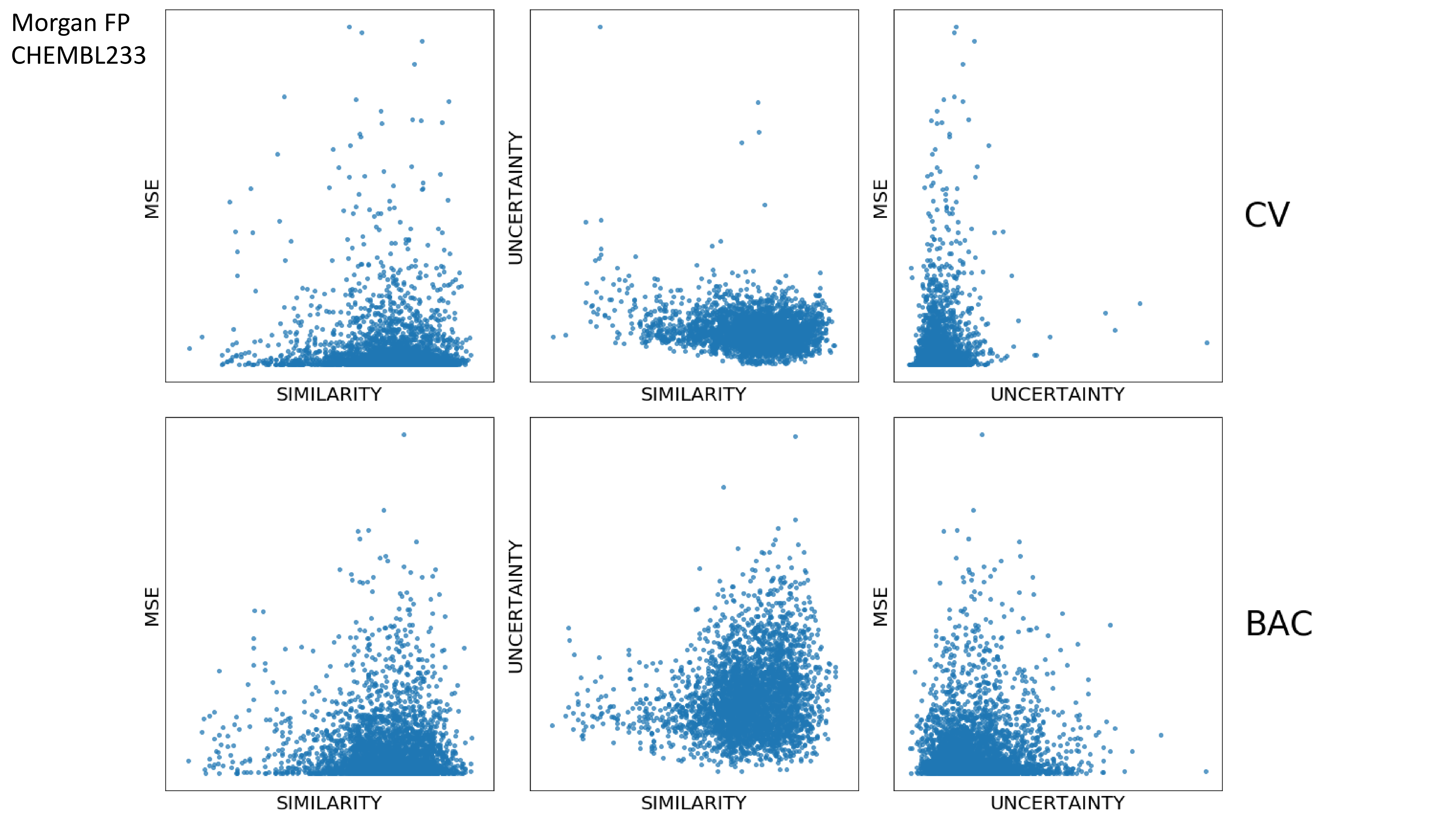
Morgan FP
CHEMBL233
MSE
SIMILARITY
UNCERTAINTY
SIMILARITY
MSE
UNCERTAINTY
CV
MSE
SIMILARITY
UNCERTAINTY
SIMILARITY
MSE
UNCERTAINTY
BAC

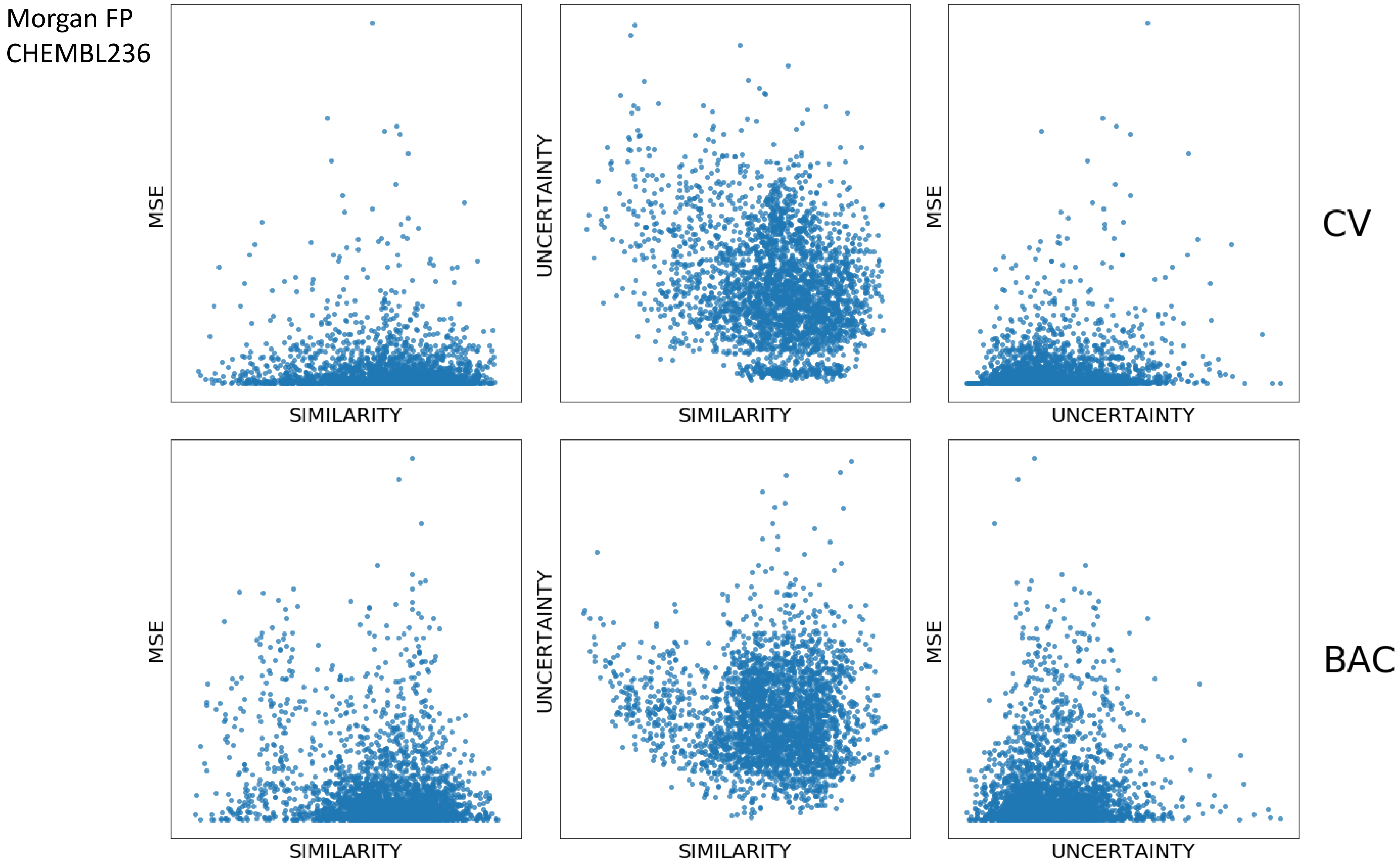
Morgan FP
CHEMBL236
MSE
SIMILARITY
UNCERTAINTY
SIMILARITY
MSE
UNCERTAINTY
CV
MSE
SIMILARITY
UNCERTAINTY
SIMILARITY
MSE
UNCERTAINTY
BAC

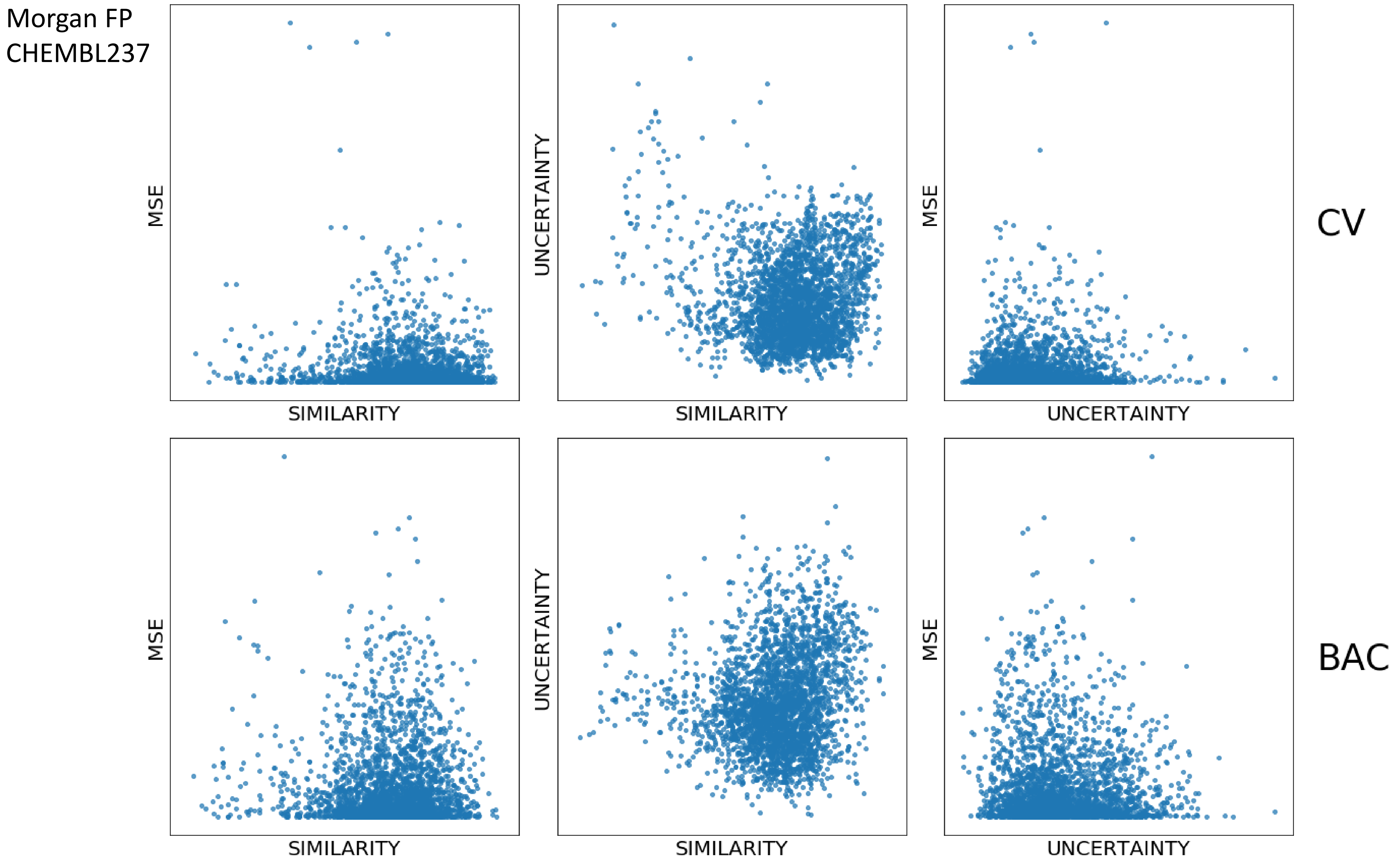
Morgan FP
CHEMBL237
MSE
SIMILARITY
UNCERTAINTY
SIMILARITY
MSE
UNCERTAINTY
CV
MSE
SIMILARITY
UNCERTAINTY
SIMILARITY
MSE
UNCERTAINTY
BAC

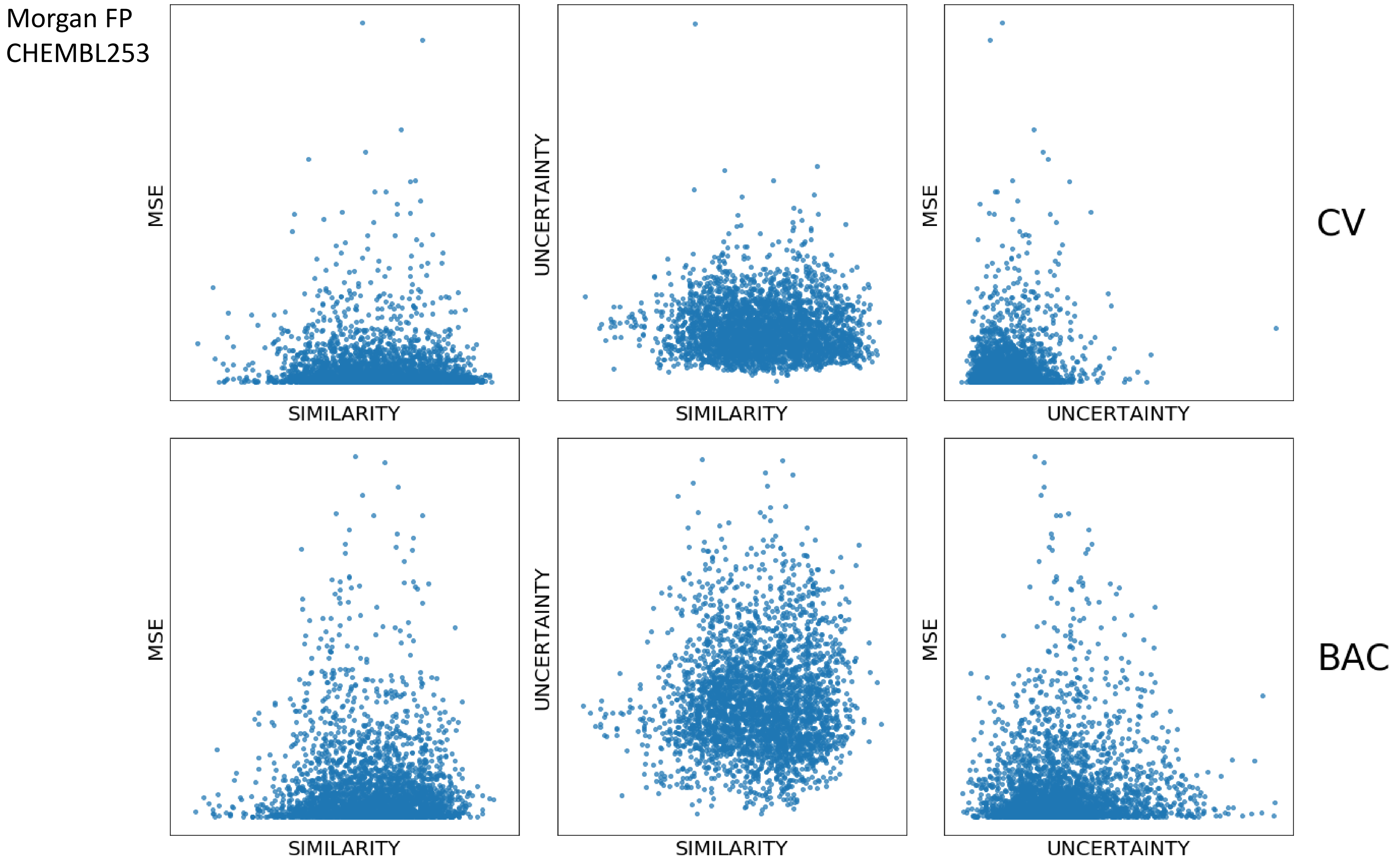
Morgan FP
CHEMBL253
MSE
SIMILARITY
UNCERTAINTY
SIMILARITY
MSE
UNCERTAINTY
CV
MSE
SIMILARITY
UNCERTAINTY
SIMILARITY
MSE
UNCERTAINTY
BAC

Morgan FP
CHEMBL259

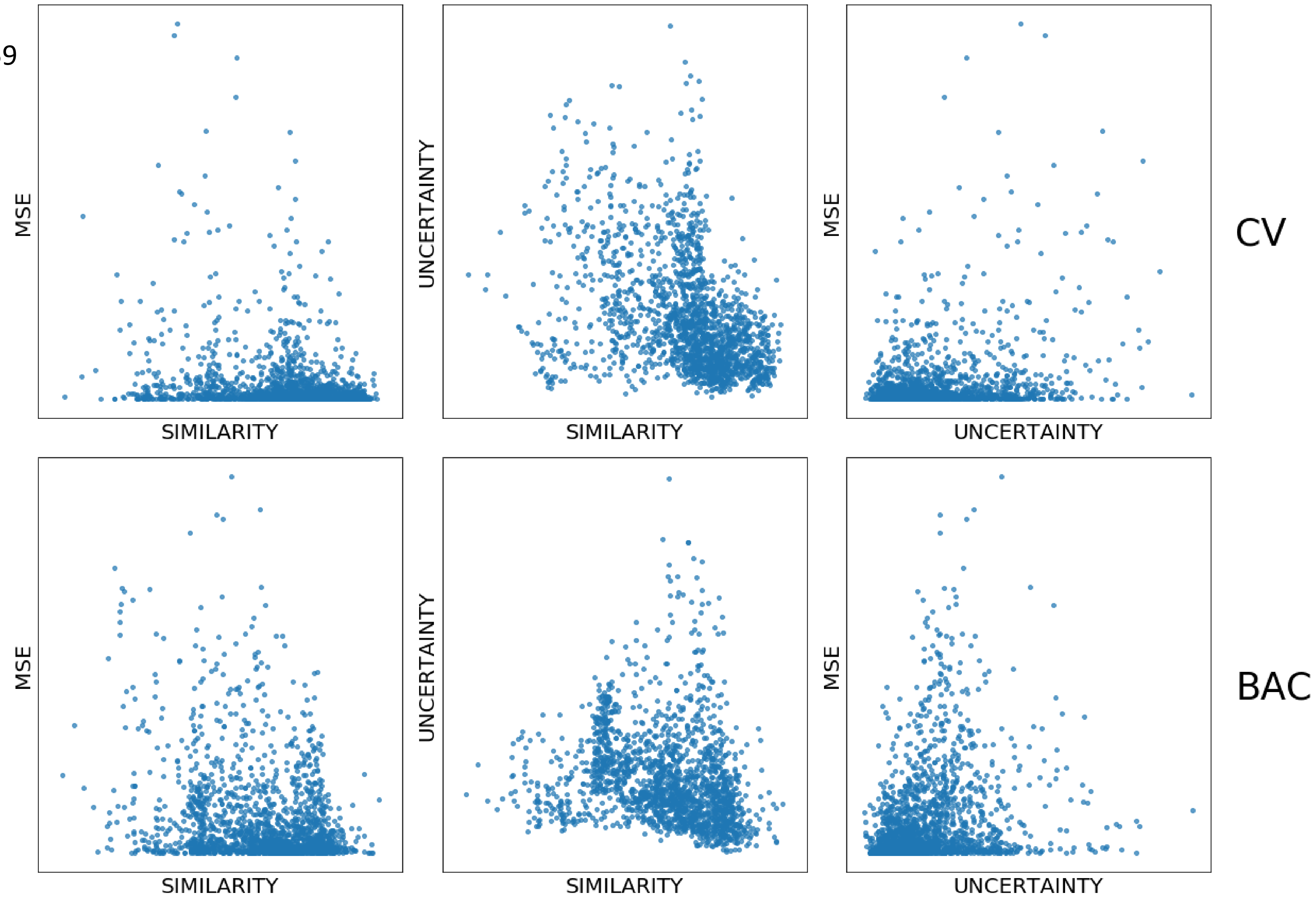

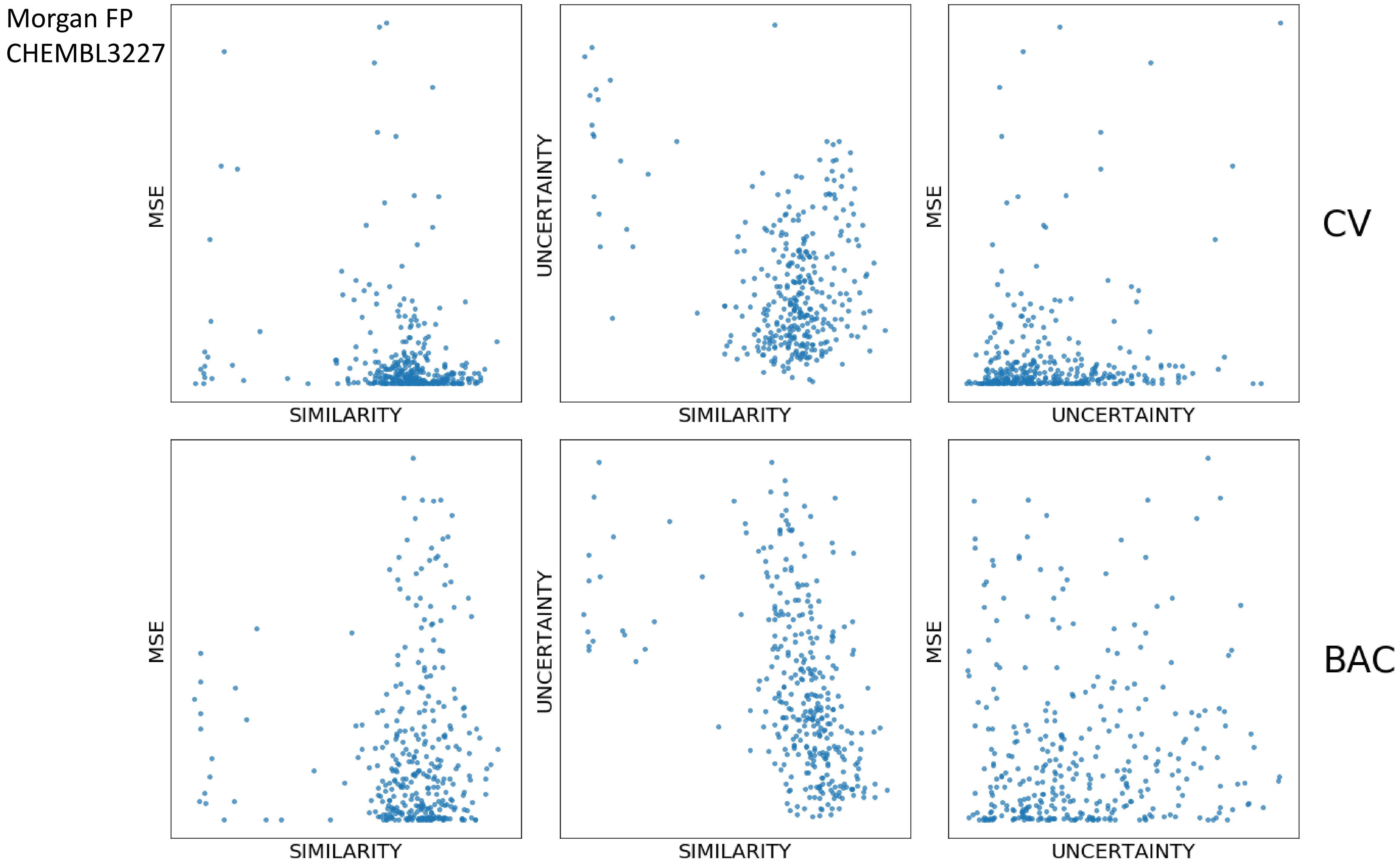
Morgan FP
CHEMBL3227
MSE
SIMILARITY
UNCERTAINTY
SIMILARITY
MSE
UNCERTAINTY
CV
MSE
SIMILARITY
UNCERTAINTY
SIMILARITY
MSE
UNCERTAINTY
BAC

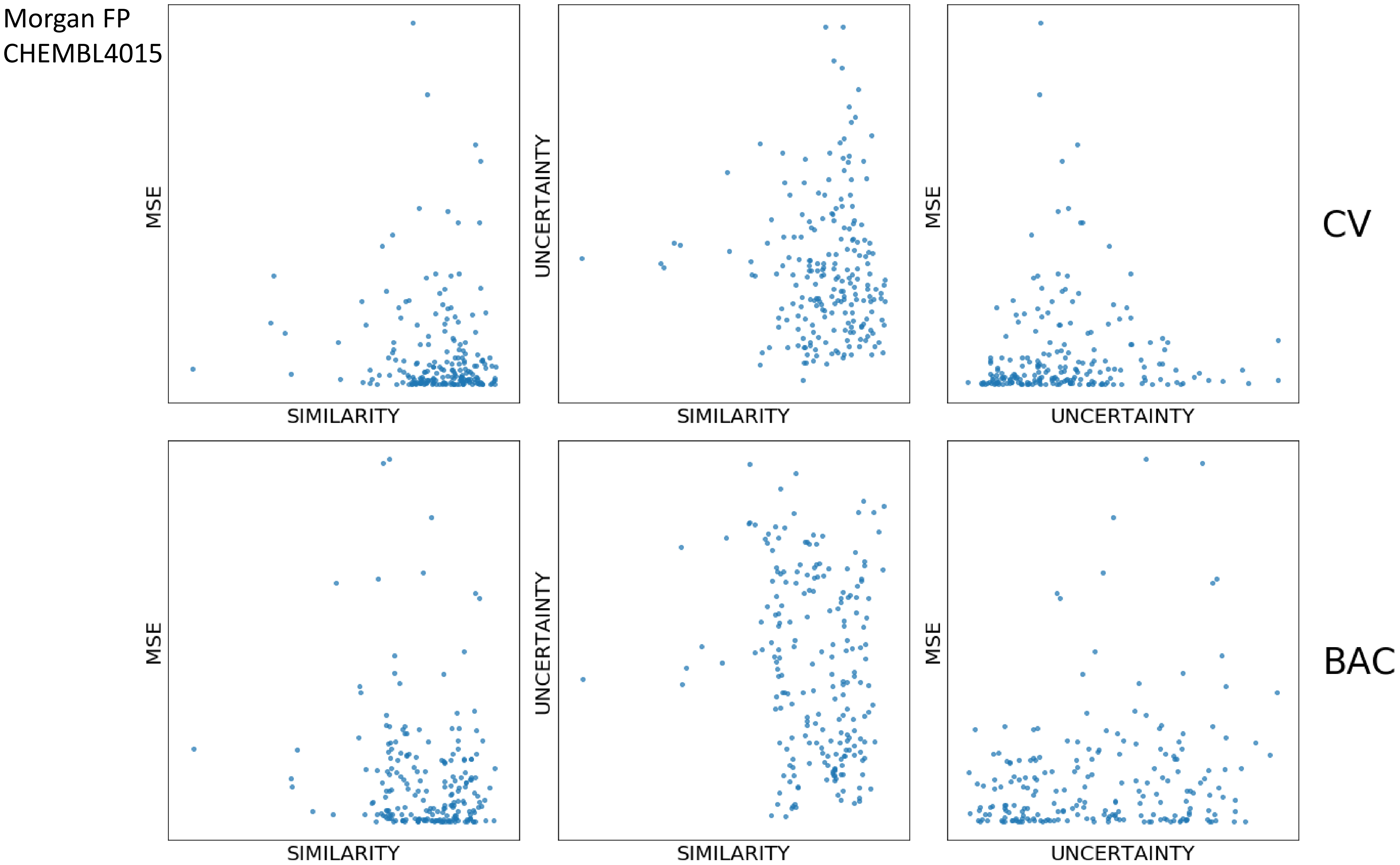

Morgan FP
CHEMBL4015
MSE
SIMILARITY
UNCERTAINTY
SIMILARITY
MSE
UNCERTAINTY
CV
MSE
SIMILARITY
UNCERTAINTY
SIMILARITY
MSE
UNCERTAINTY
BAC

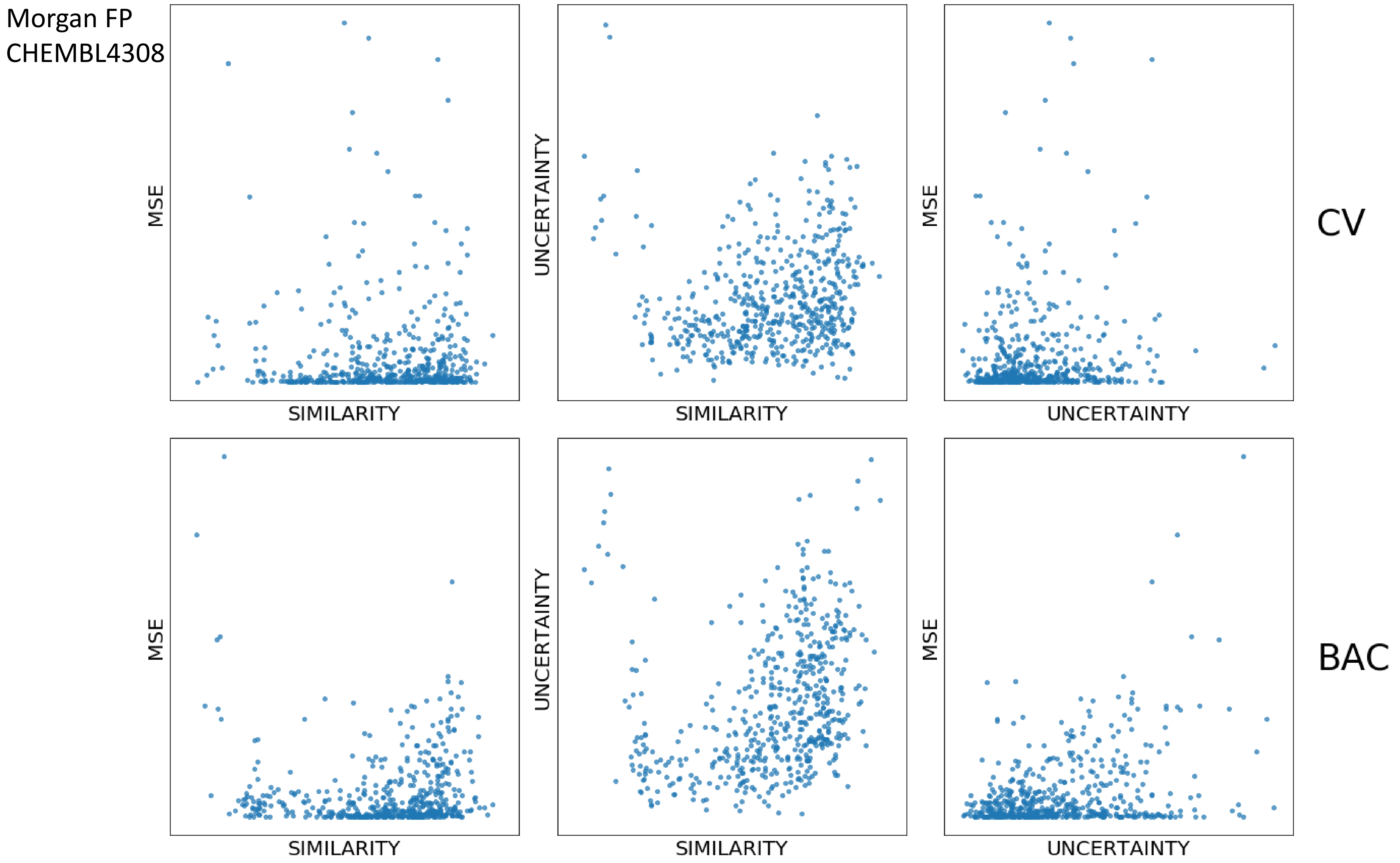
Morgan FP
CHEMBL4308
MSE
SIMILARITY
UNCERTAINTY
SIMILARITY
MSE
UNCERTAINTY
CV
MSE
SIMILARITY
UNCERTAINTY
SIMILARITY
MSE
UNCERTAINTY
BAC

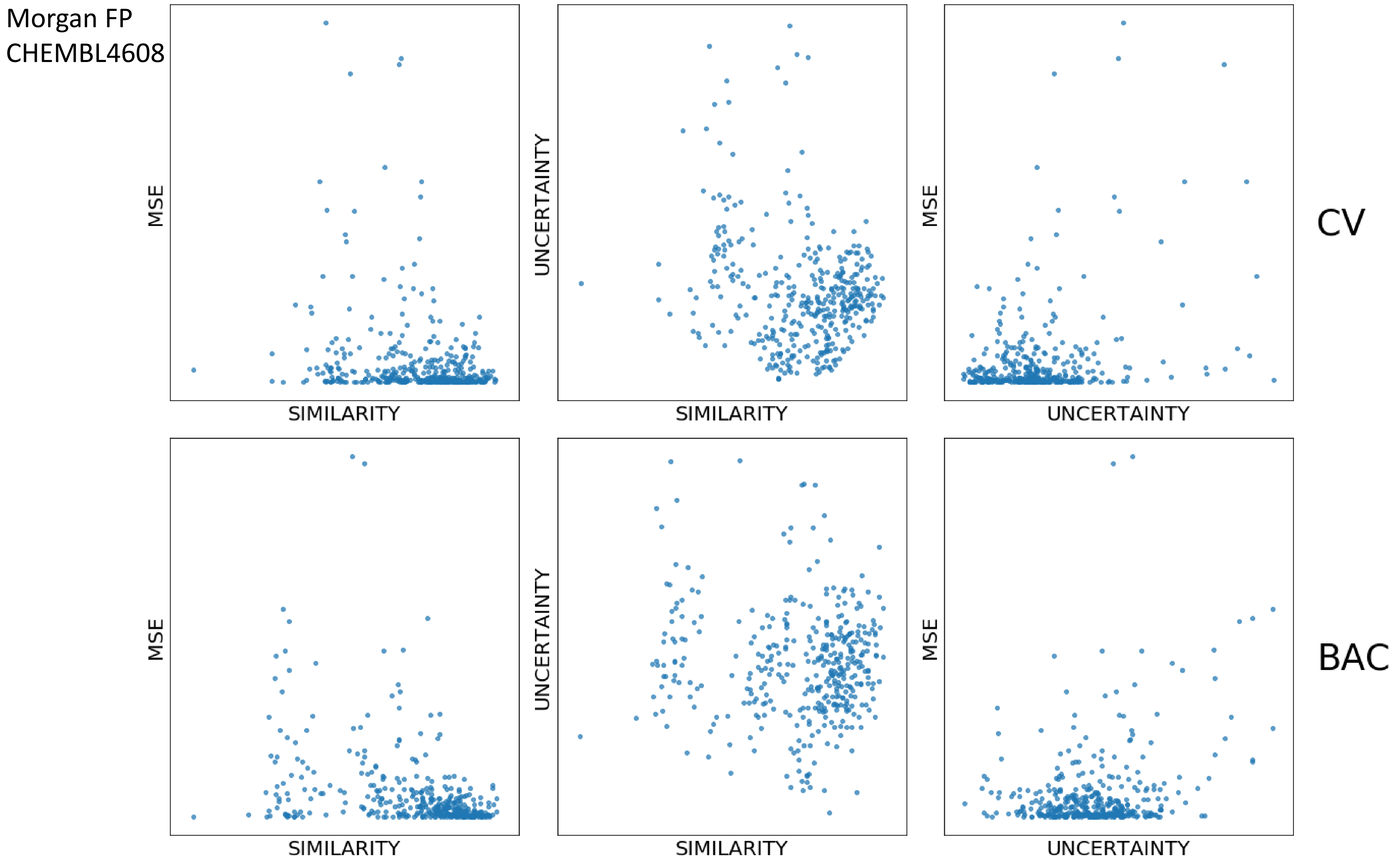
Morgan FP
CHEMBL4608
MSE
SIMILARITY
UNCERTAINTY
SIMILARITY
MSE
UNCERTAINTY
CV
MSE
SIMILARITY
UNCERTAINTY
SIMILARITY
MSE
UNCERTAINTY
BAC

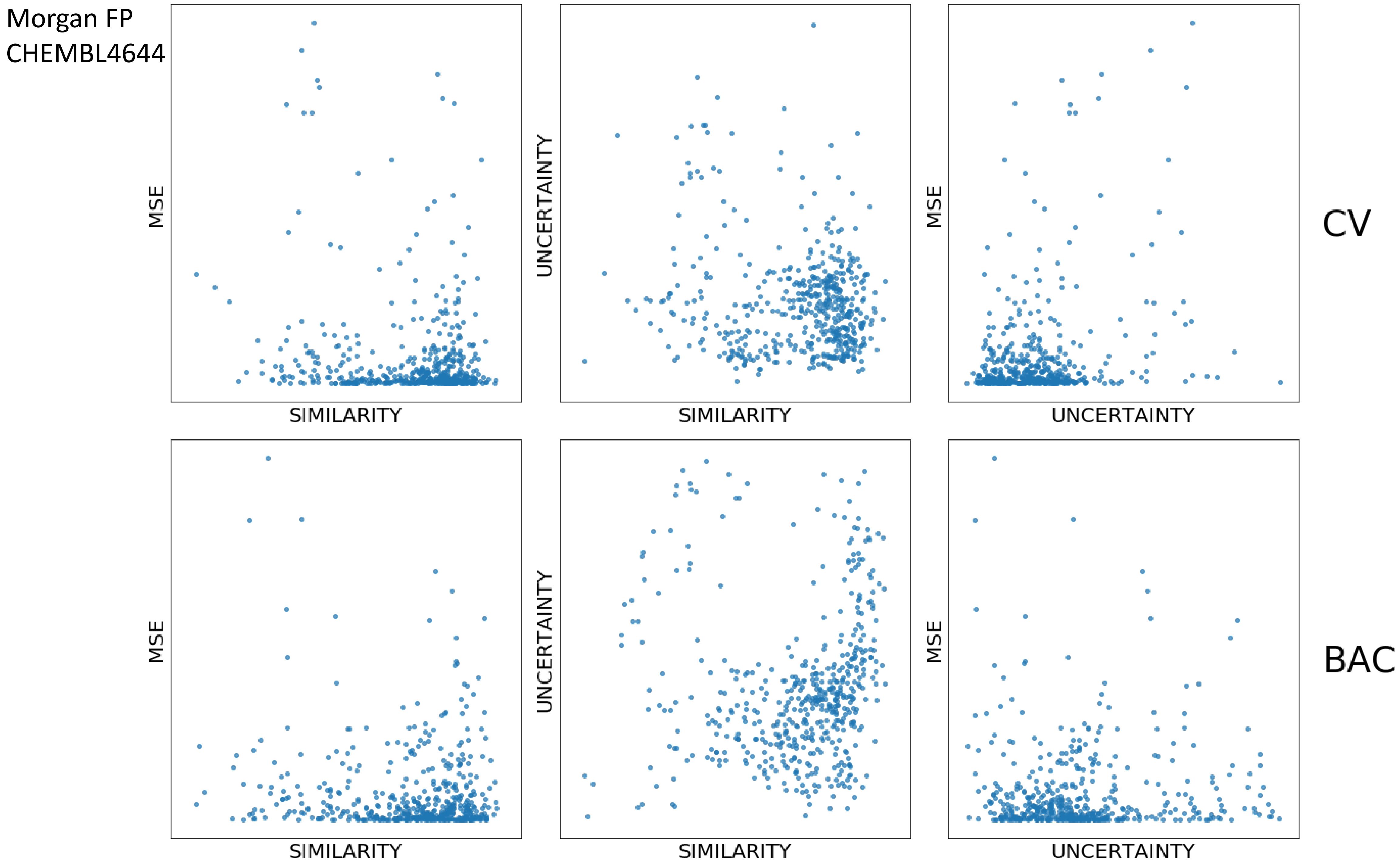
Morgan FP
CHEMBL4644
MSE
SIMILARITY
UNCERTAINTY
SIMILARITY
MSE
UNCERTAINTY
CV
MSE
SIMILARITY
UNCERTAINTY
SIMILARITY
MSE
UNCERTAINTY
BAC

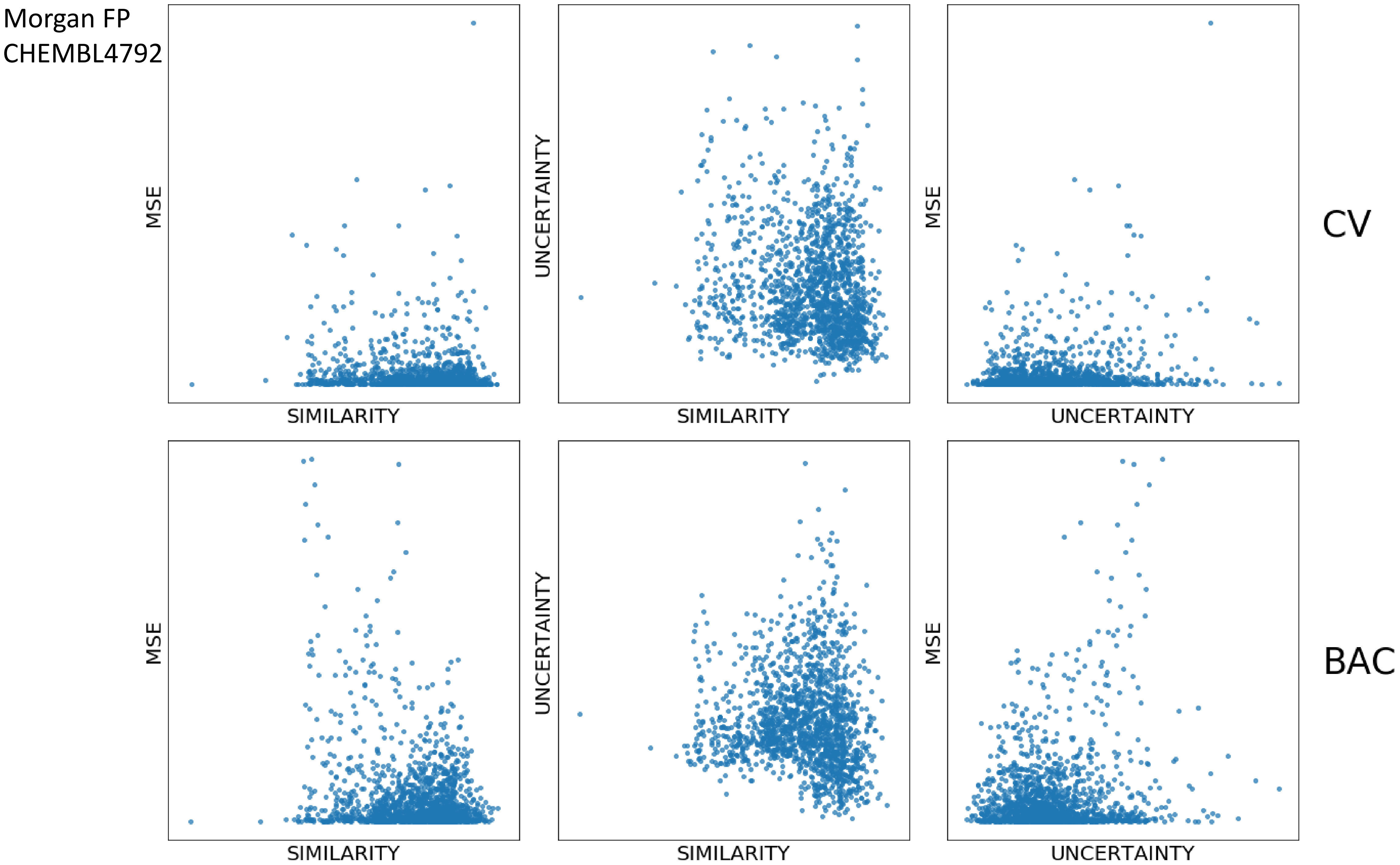
Morgan FP
CHEMBL4792
MSE
SIMILARITY
UNCERTAINTY
SIMILARITY
MSE
UNCERTAINTY
CV
MSE
SIMILARITY
UNCERTAINTY
SIMILARITY
MSE
UNCERTAINTY
BAC

Morgan FP
CHEMBL5113

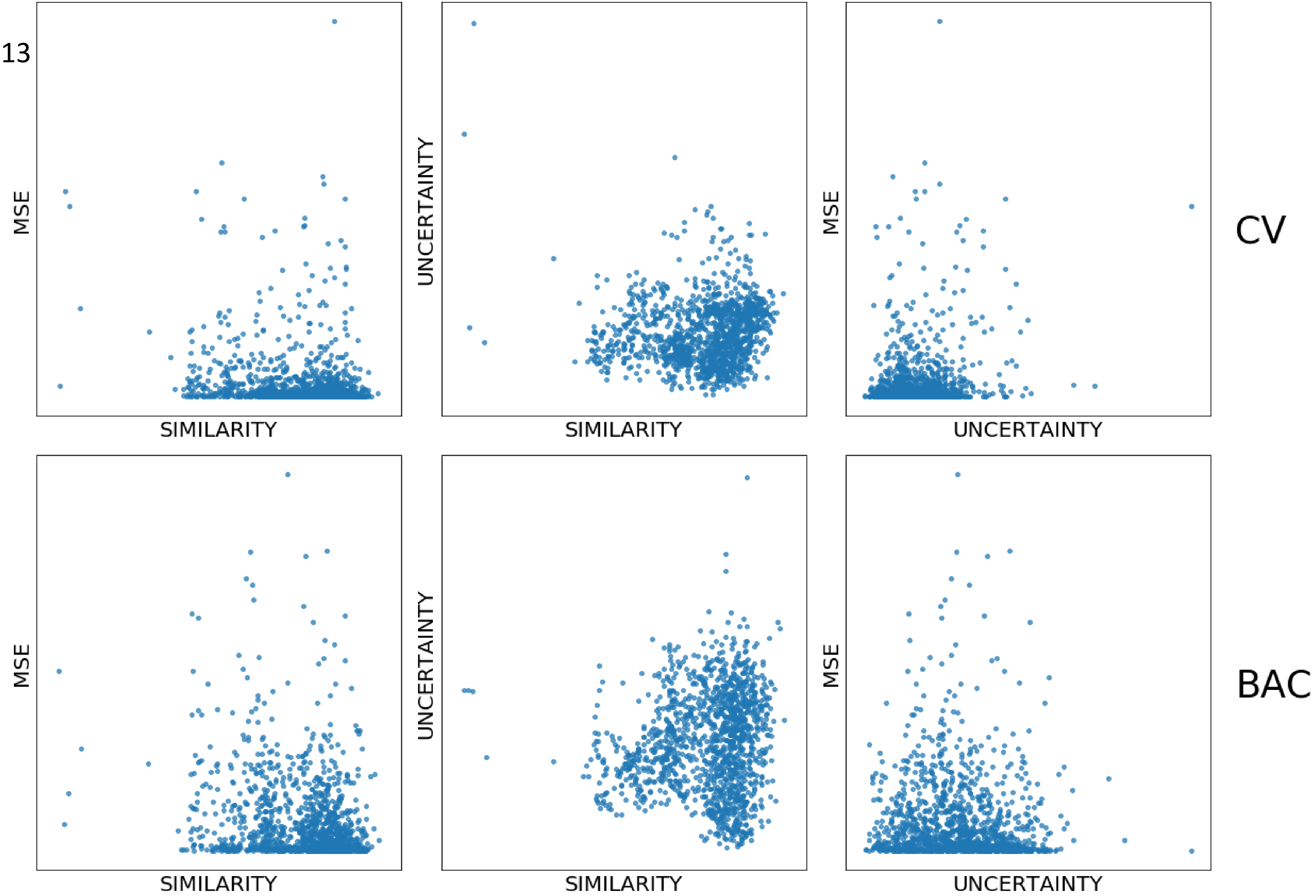

MACCSFP
CHEMBL214

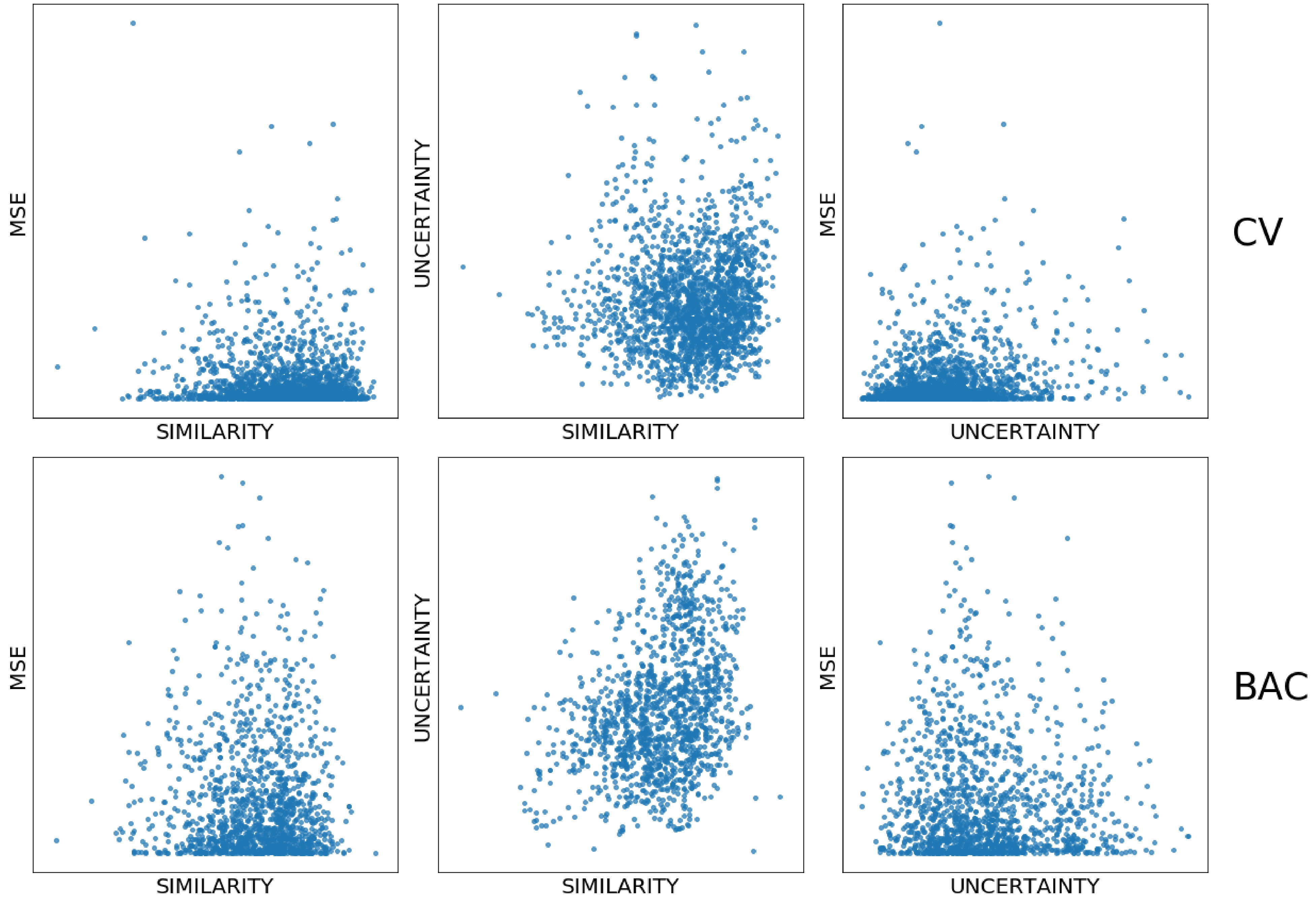

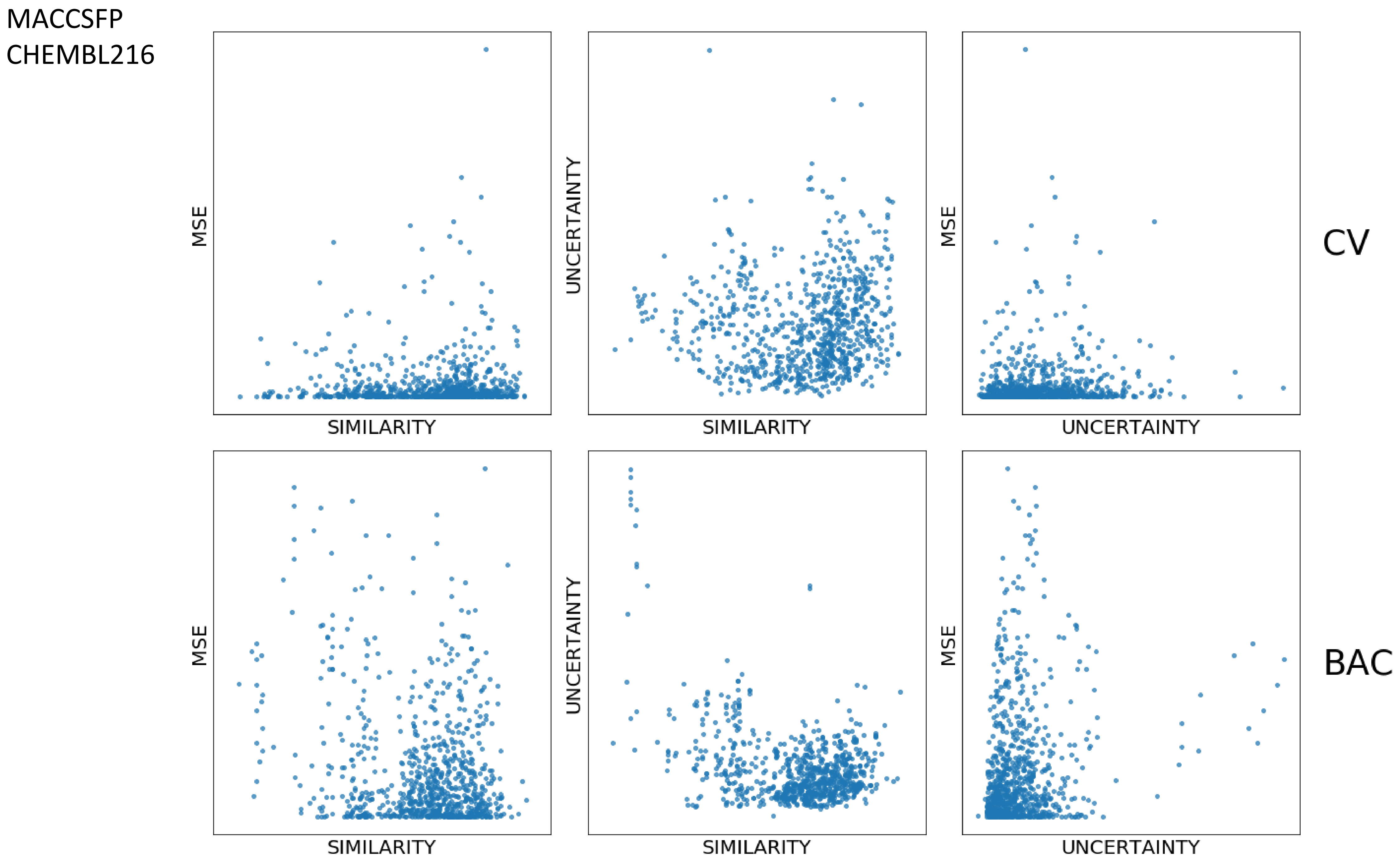
MACCSFP
CHEMBL216
MSE
SIMILARITY
UNCERTAINTY
SIMILARITY
MSE
UNCERTAINTY
CV
MSE
SIMILARITY
UNCERTAINTY
SIMILARITY
MSE
UNCERTAINTY
BAC

MACCSFP
CHEMBL217

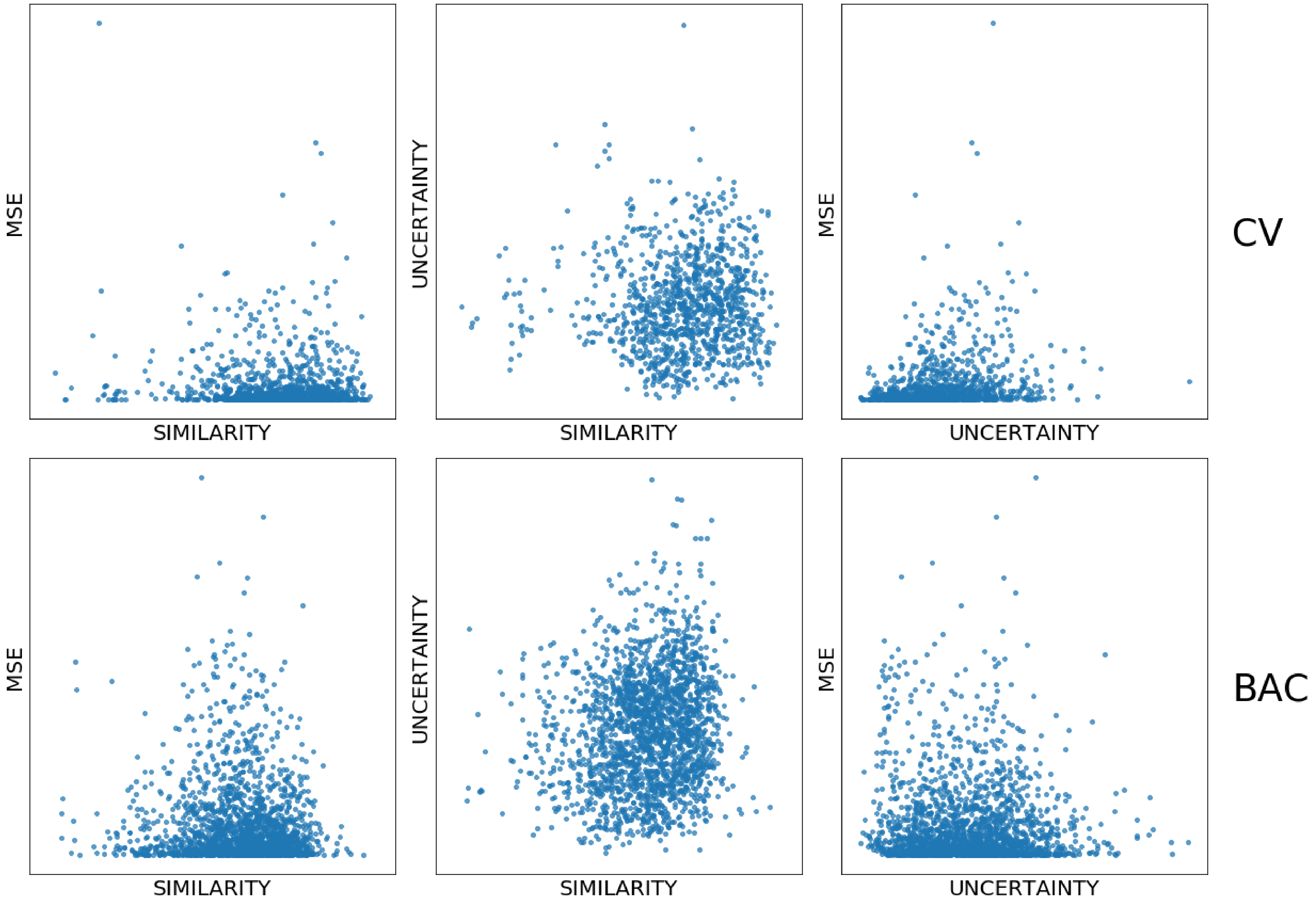

MACCSFP
CHEMBL224

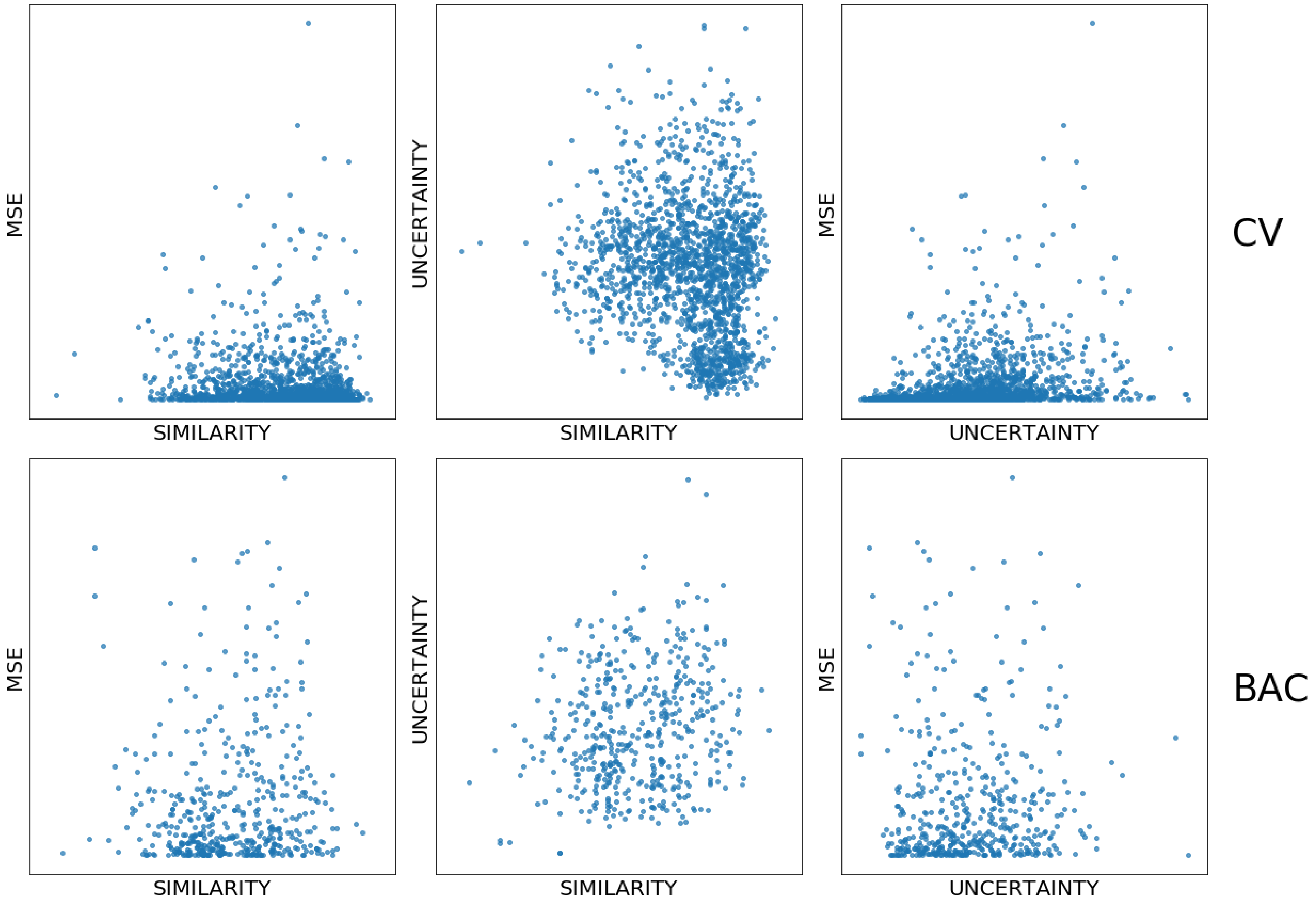

MACCSFP
CHEMBL225

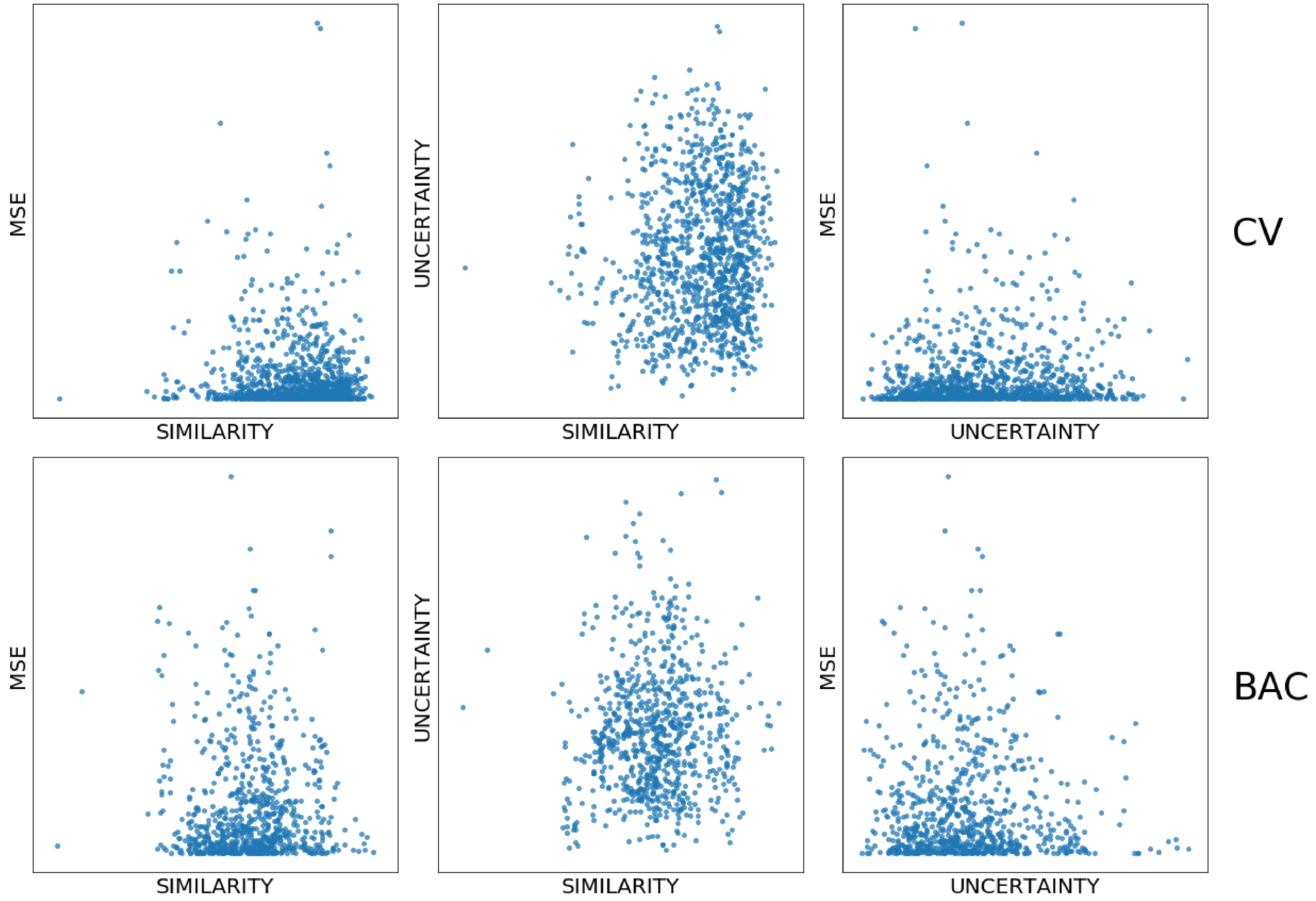

MACCSFP
CHEMBL226

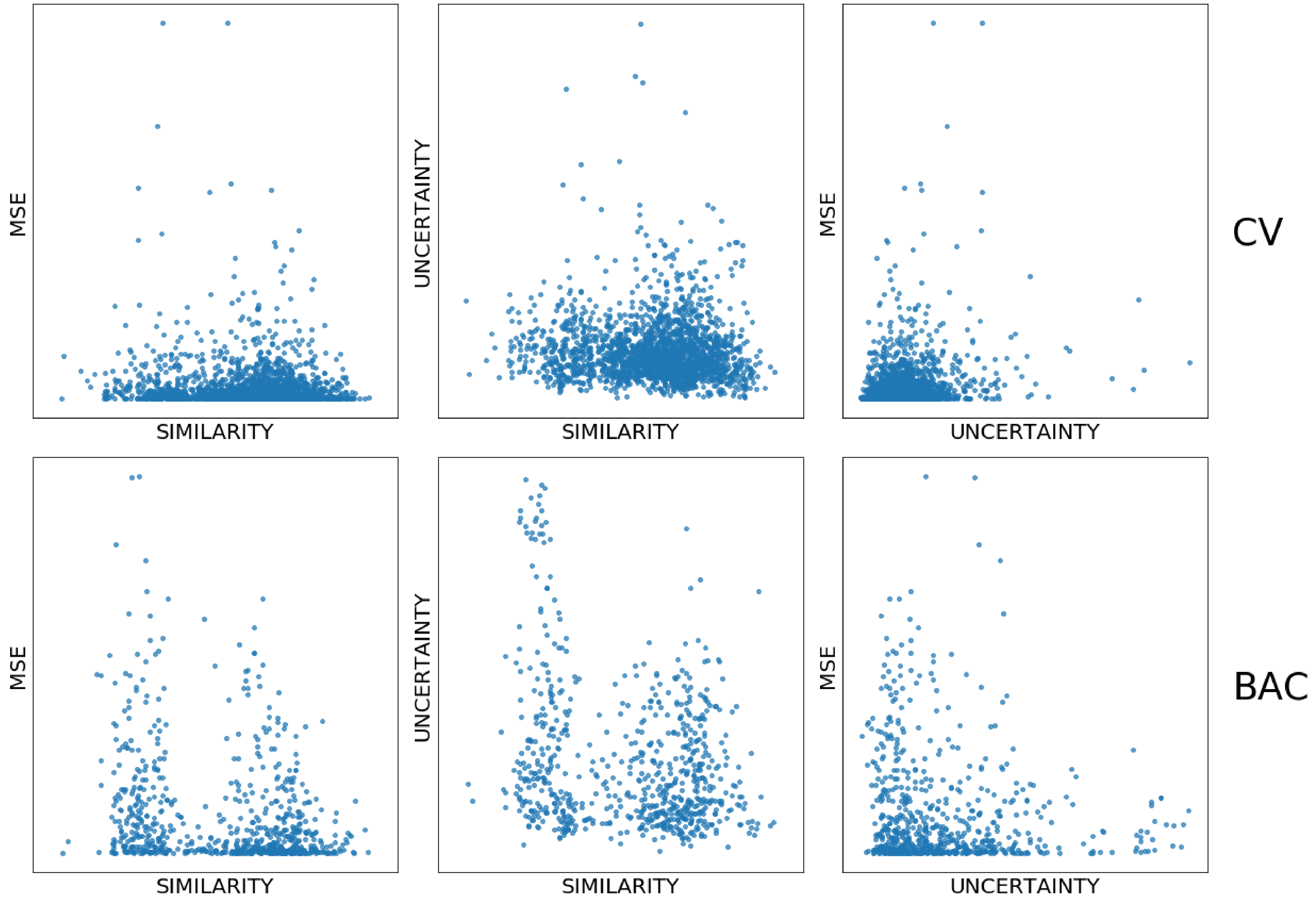

MACCSFP
CHEMBL251

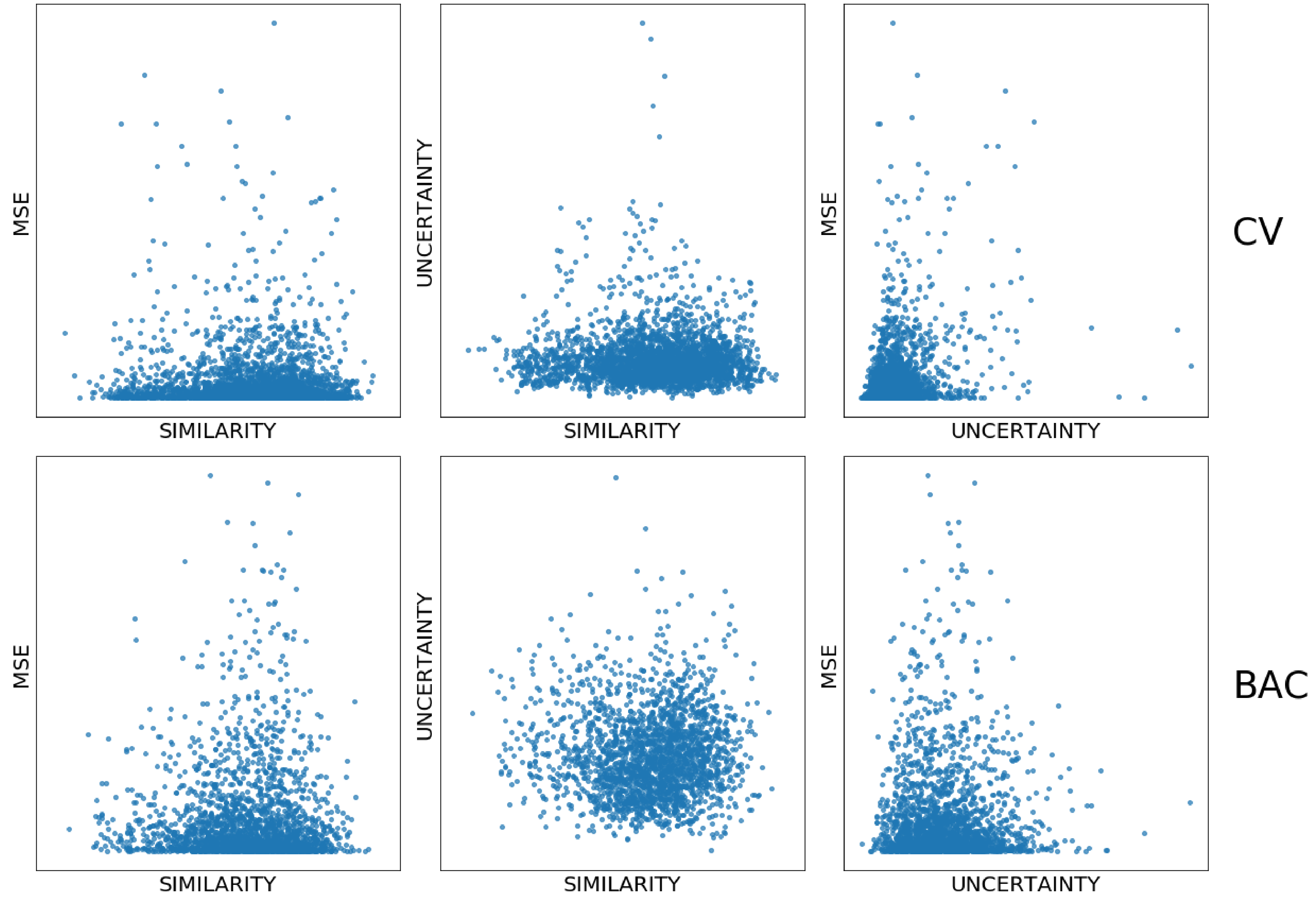

MACCSFP
CHEMBL264

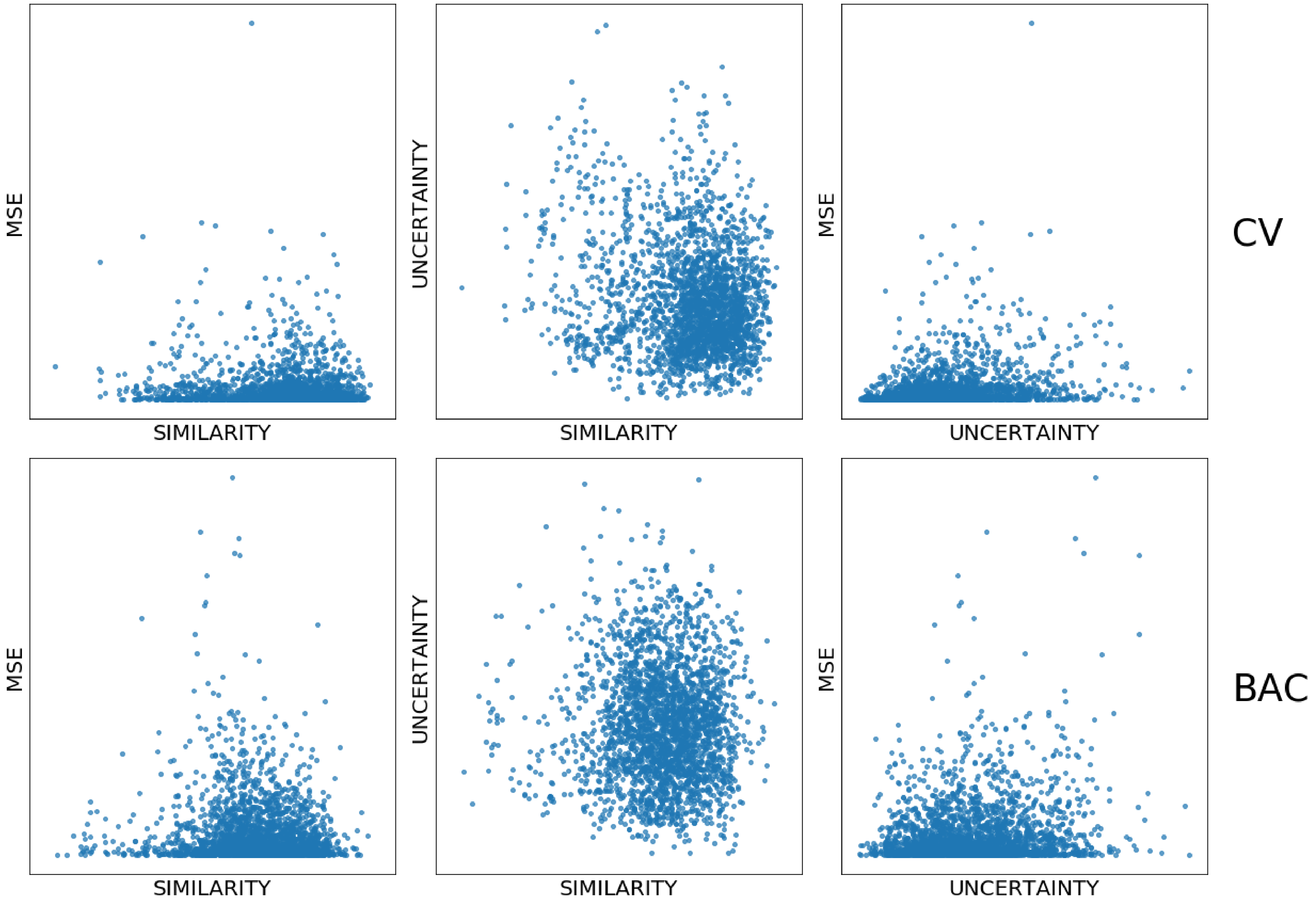

MACCSFP
CHEMBL3155

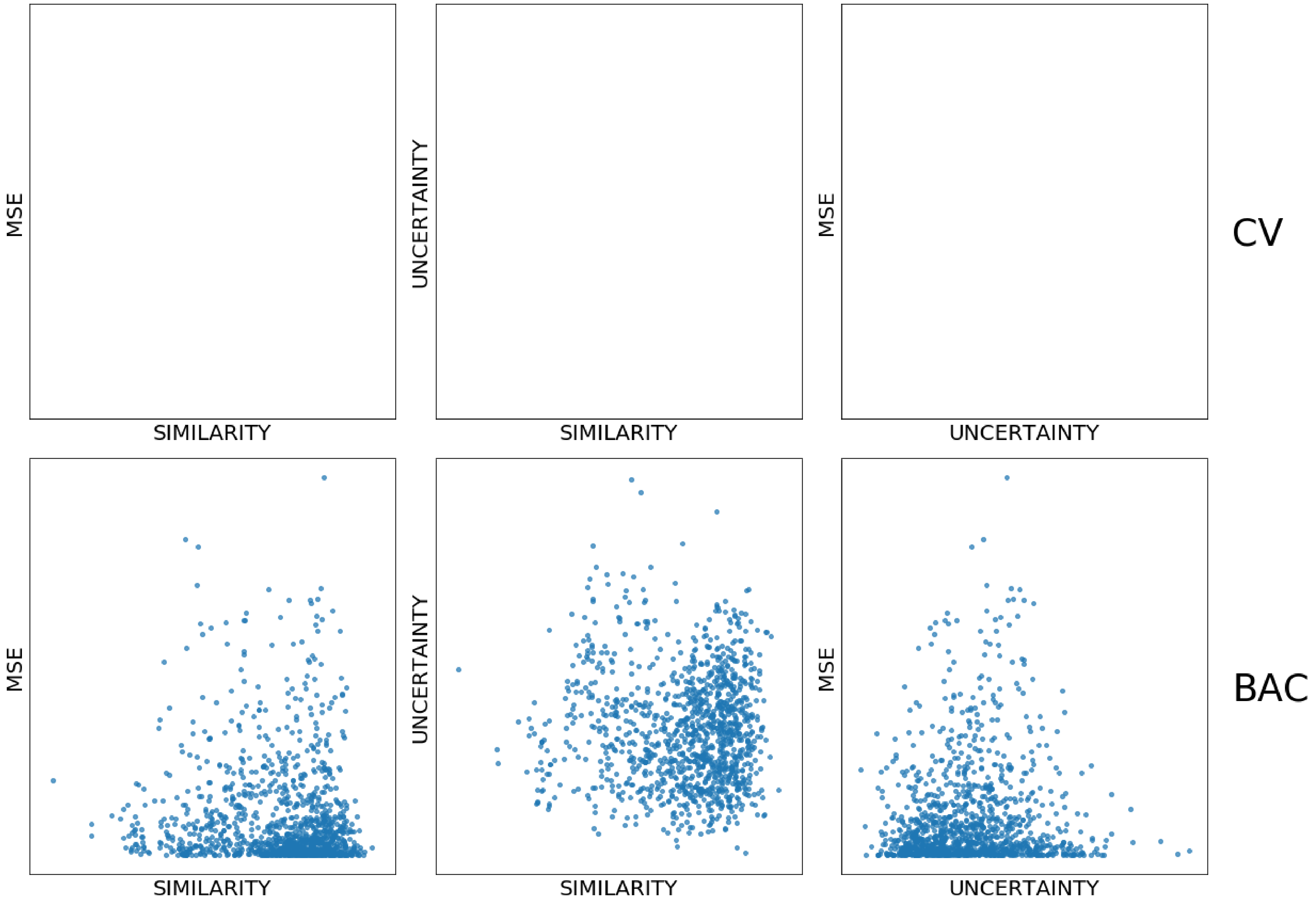

MACCSFP
CHEMBL3371

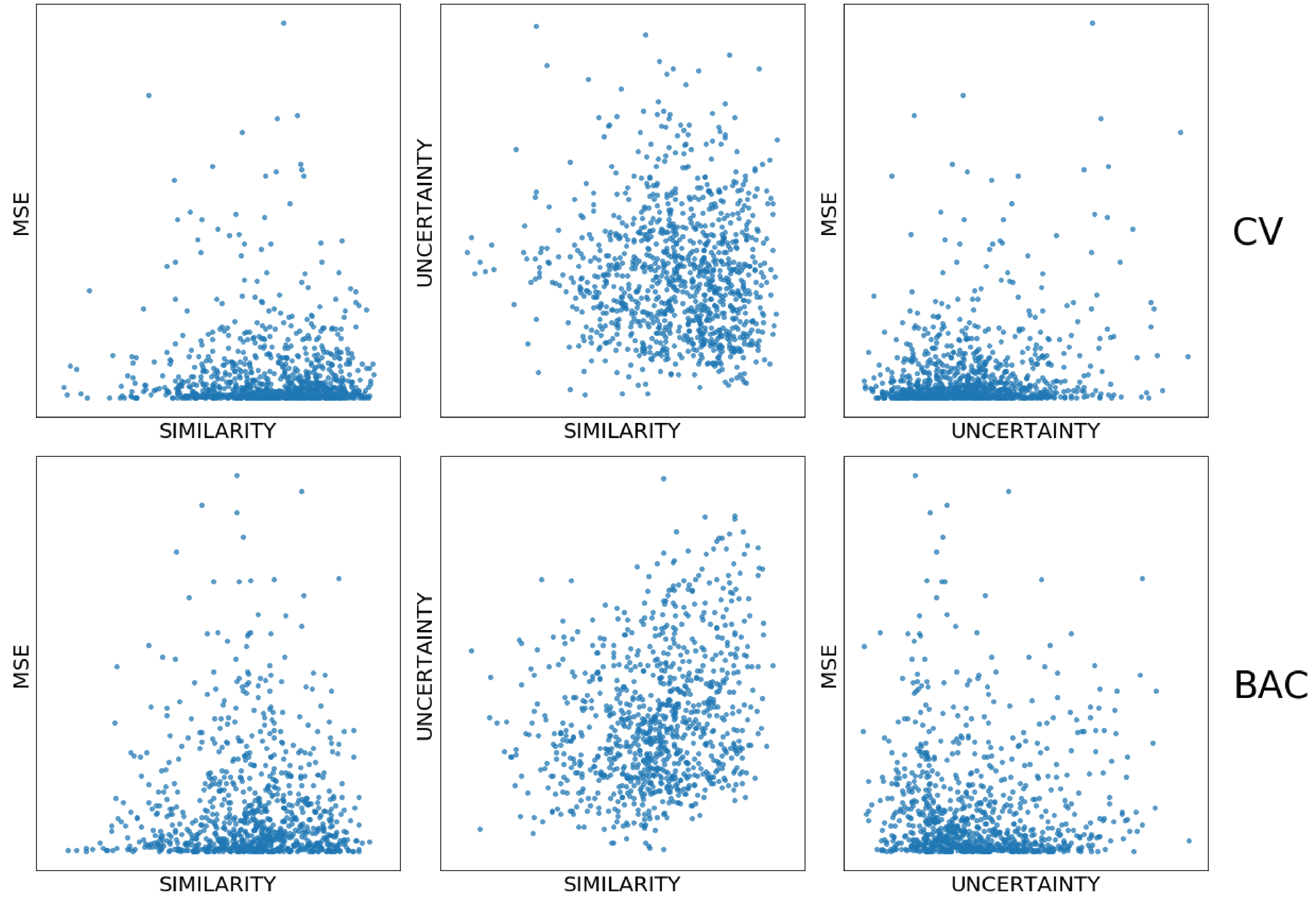

MACCSFP
CHEMBL1945

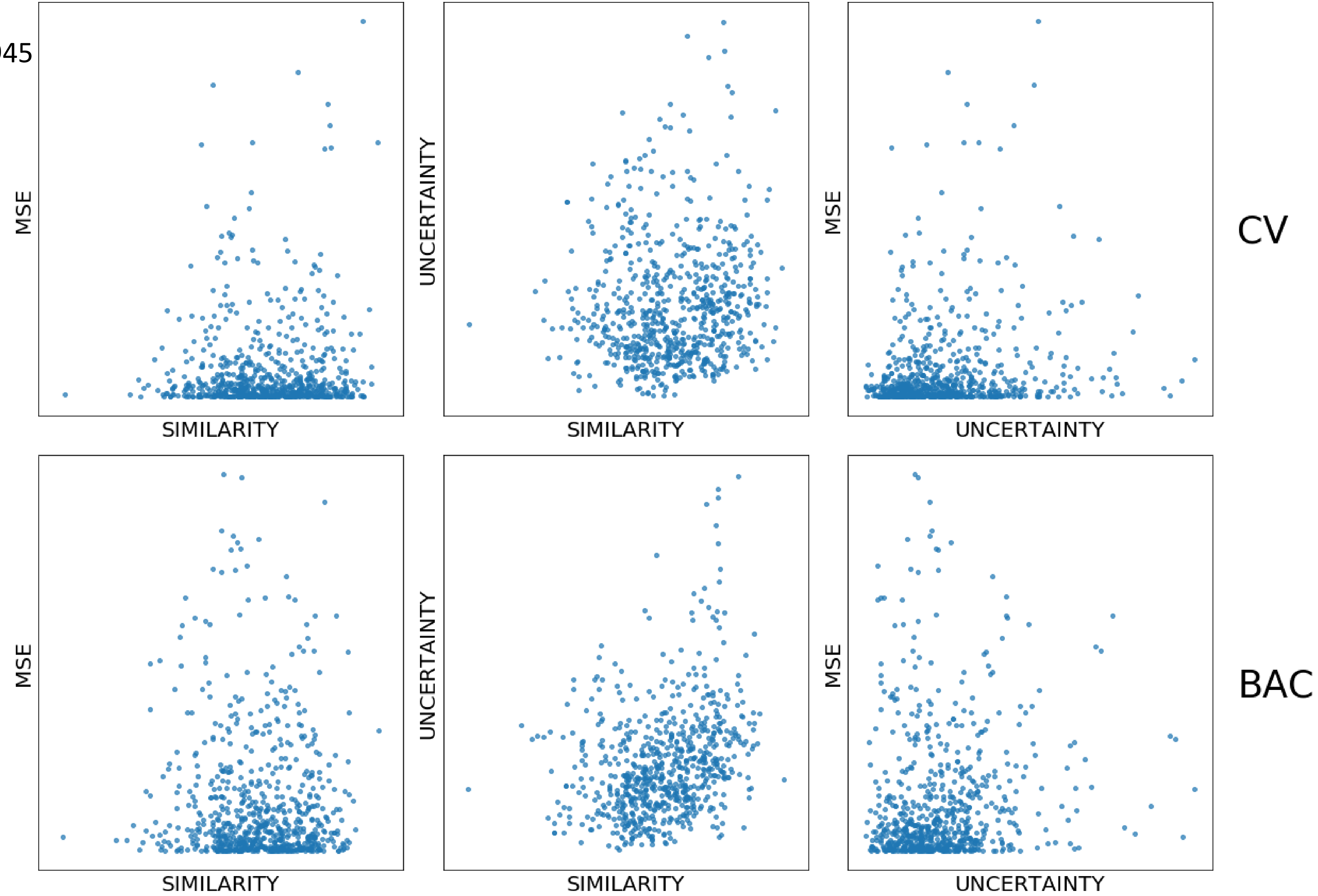

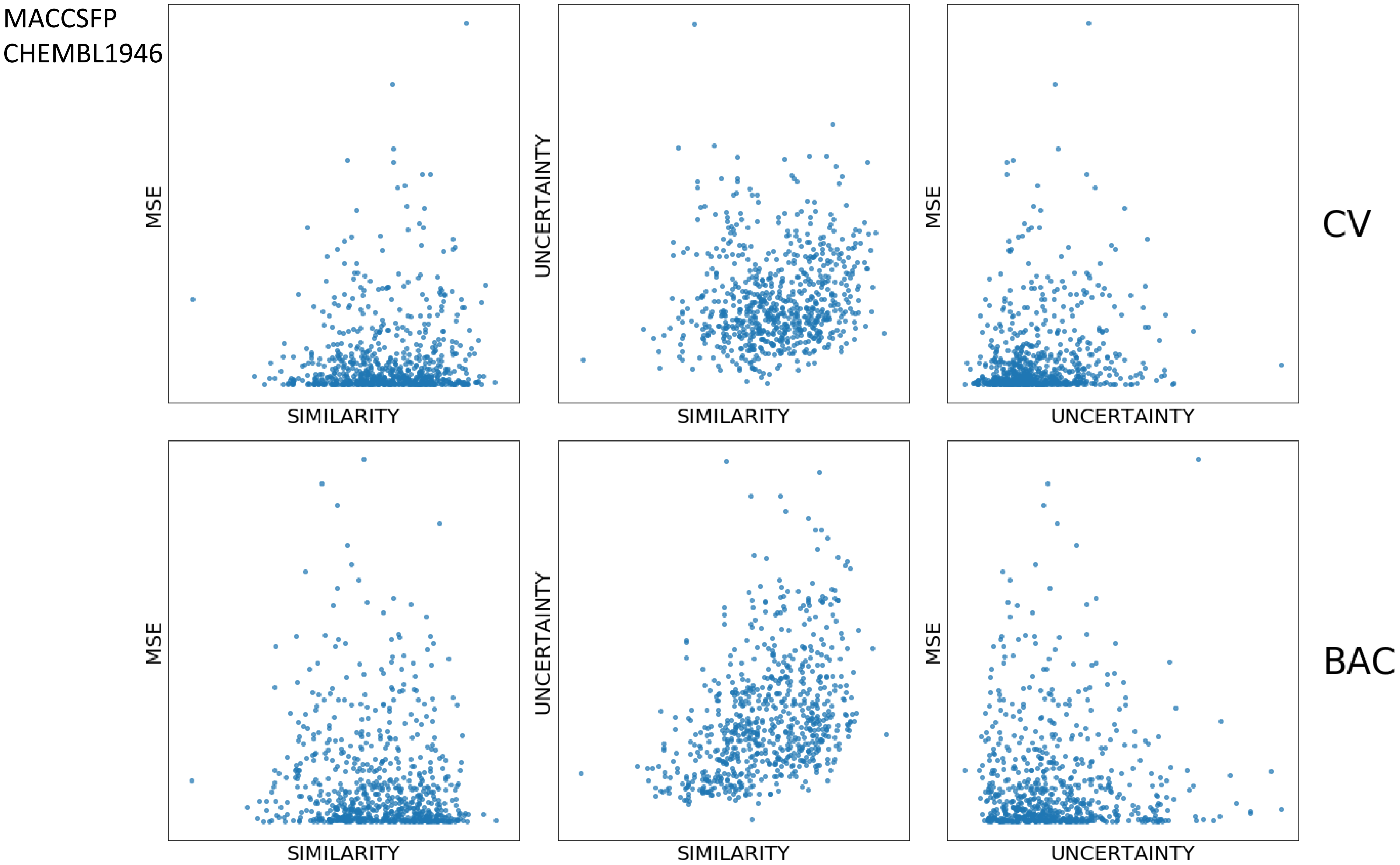
MACCSFP
CHEMBL1946
MSE
SIMILARITY
UNCERTAINTY
SIMILARITY
MSE
UNCERTAINTY
CV
MSE
SIMILARITY
UNCERTAINTY
SIMILARITY
MSE
UNCERTAINTY
BAC

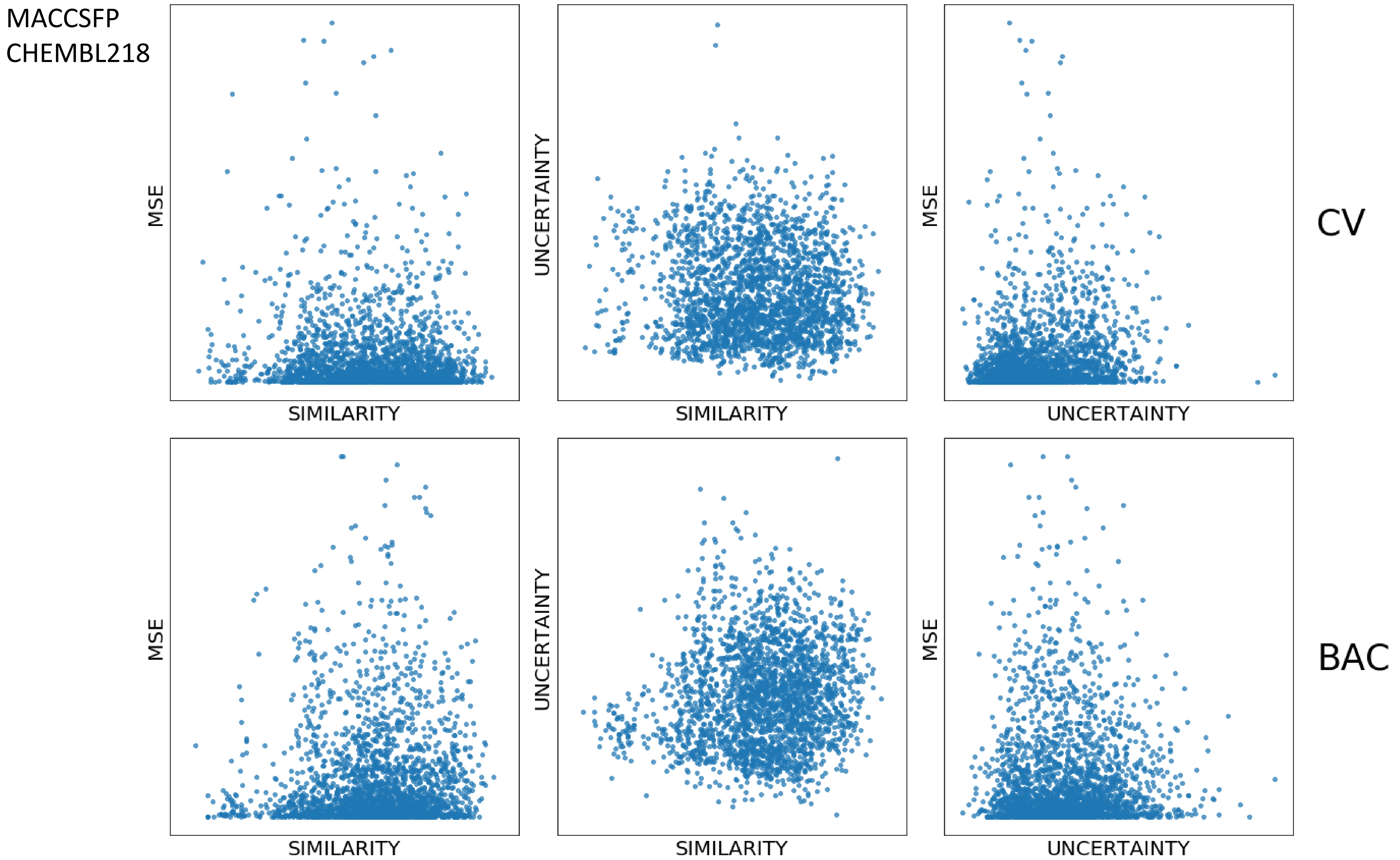
MACCSFP
CHEMBL218
MSE
SIMILARITY
UNCERTAINTY
SIMILARITY
MSE
UNCERTAINTY
CV
MSE
SIMILARITY
UNCERTAINTY
SIMILARITY
MSE
UNCERTAINTY
BAC

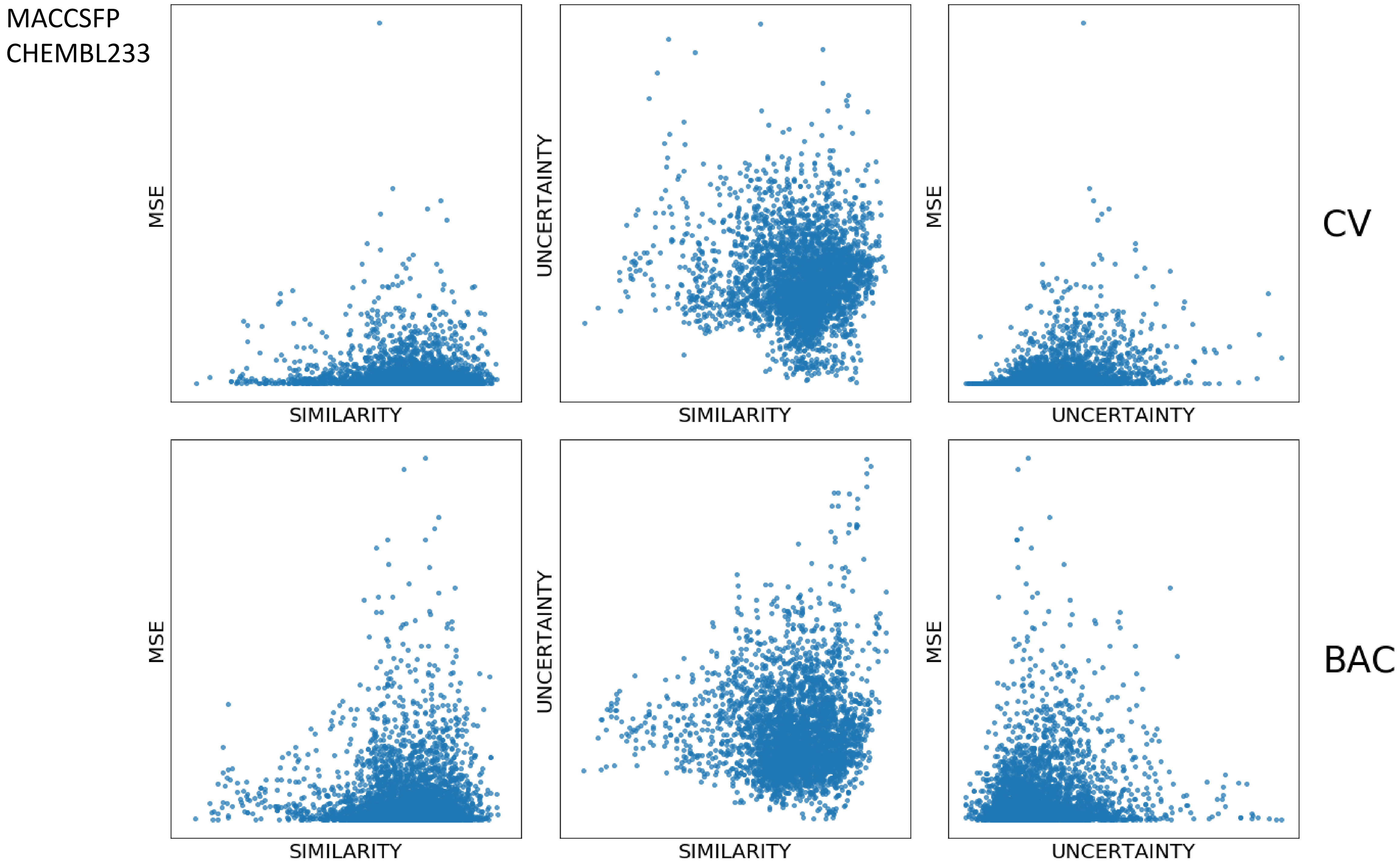
MACCSFP
CHEMBL233
MSE
SIMILARITY
UNCERTAINTY
SIMILARITY
MSE
UNCERTAINTY
CV
MSE
SIMILARITY
UNCERTAINTY
SIMILARITY
MSE
UNCERTAINTY
BAC

MACCSFP
CHEMBL236

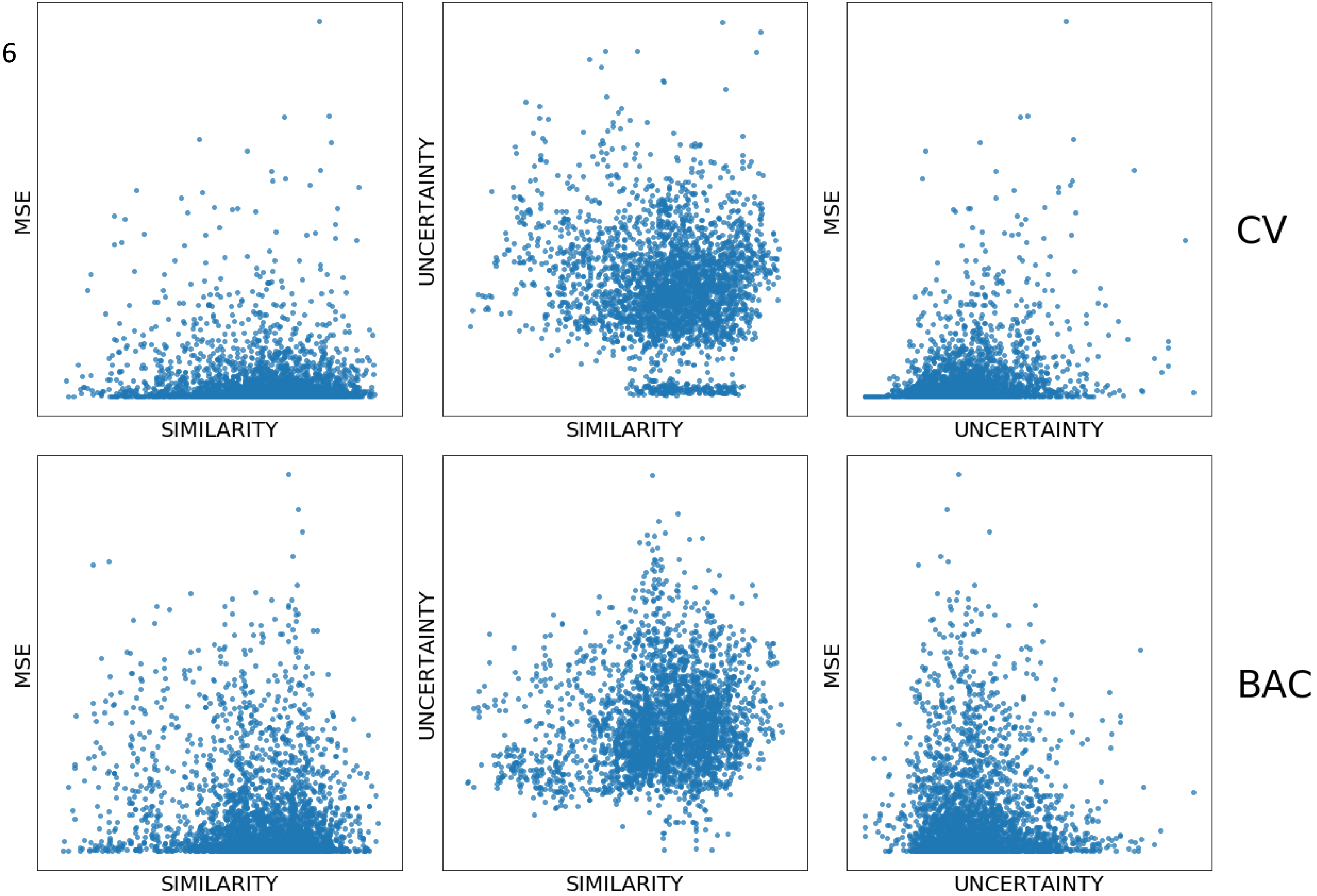

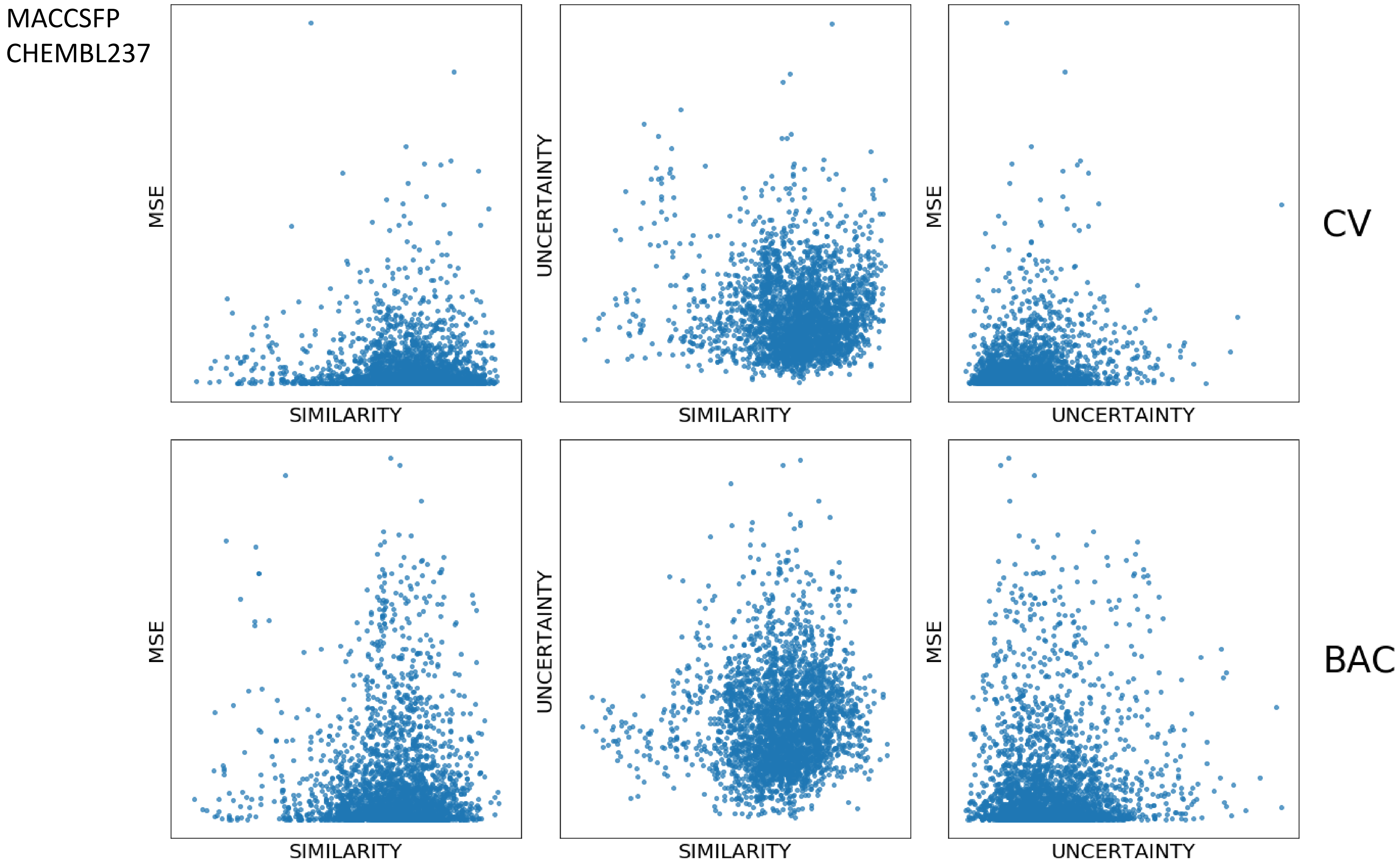
MACCSFP
CHEMBL237
MSE
SIMILARITY
UNCERTAINTY
SIMILARITY
MSE
UNCERTAINTY
CV
MSE
SIMILARITY
UNCERTAINTY
SIMILARITY
MSE
UNCERTAINTY
BAC

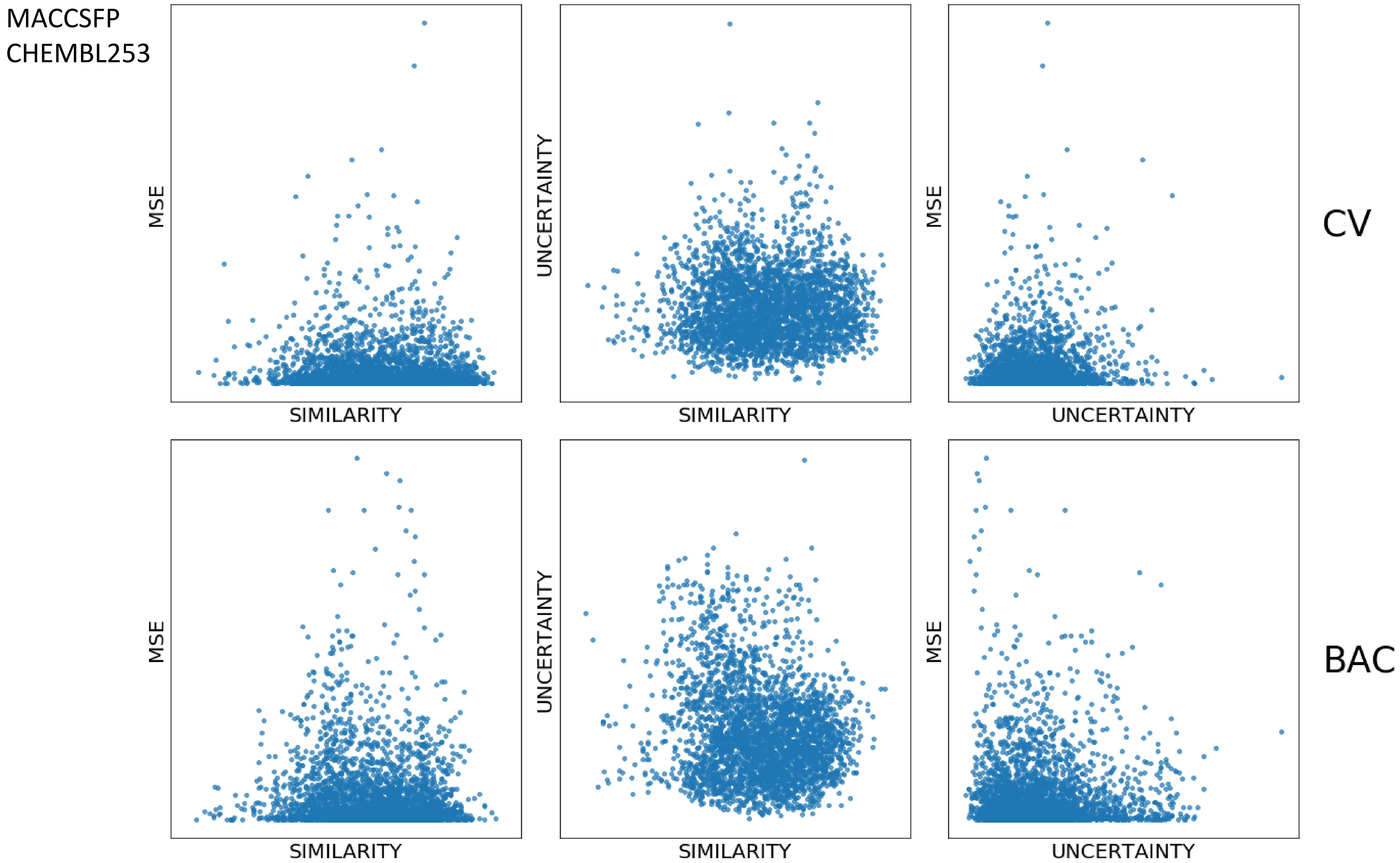
MACCSFP
CHEMBL253
MSE
SIMILARITY
UNCERTAINTY
SIMILARITY
MSE
UNCERTAINTY
CV
MSE
SIMILARITY
UNCERTAINTY
SIMILARITY
MSE
UNCERTAINTY
BAC

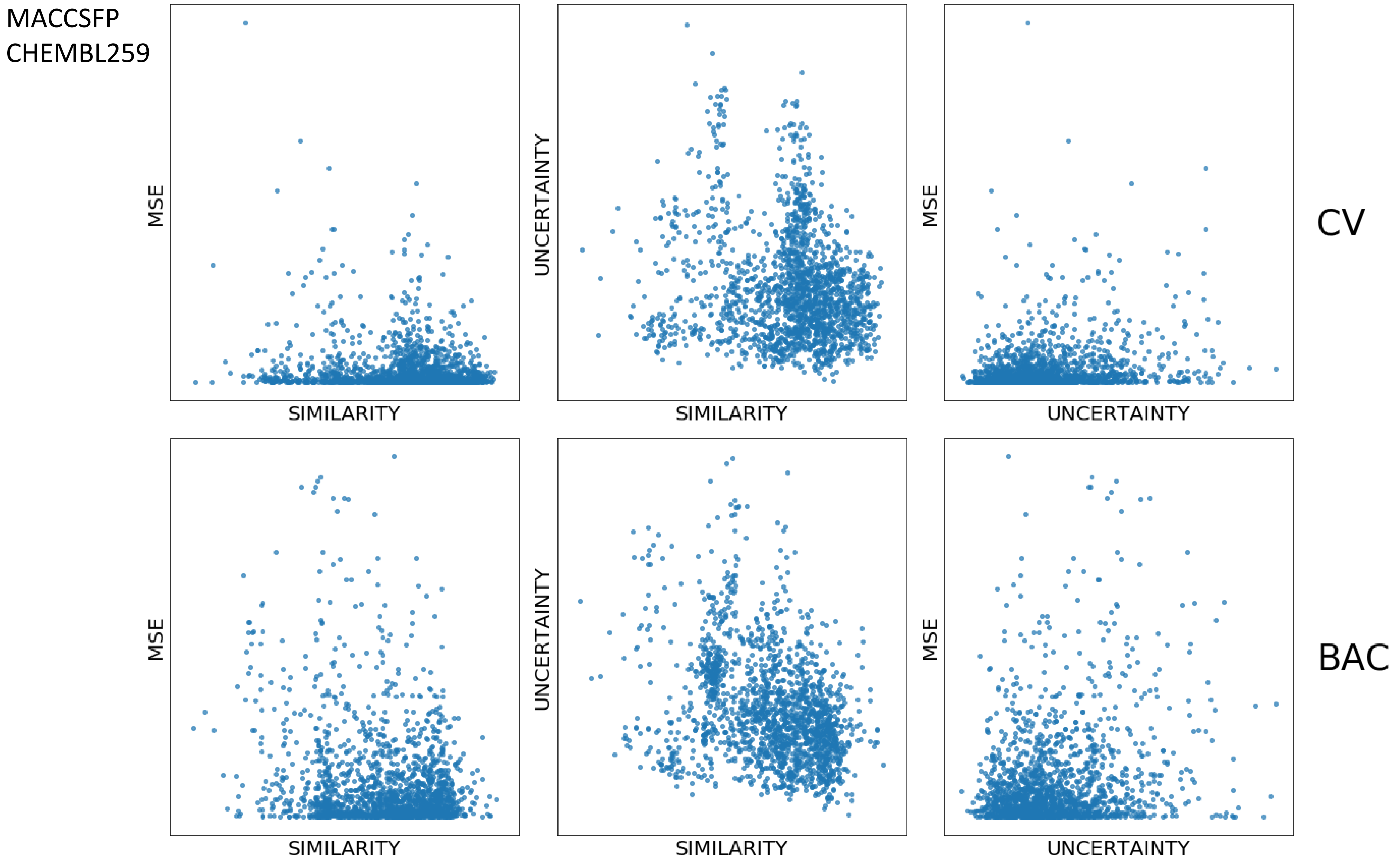
MACCSFP
CHEMBL259
MSE
SIMILARITY
UNCERTAINTY
SIMILARITY
MSE
UNCERTAINTY
CV
MSE
SIMILARITY
UNCERTAINTY
SIMILARITY
MSE
UNCERTAINTY
BAC

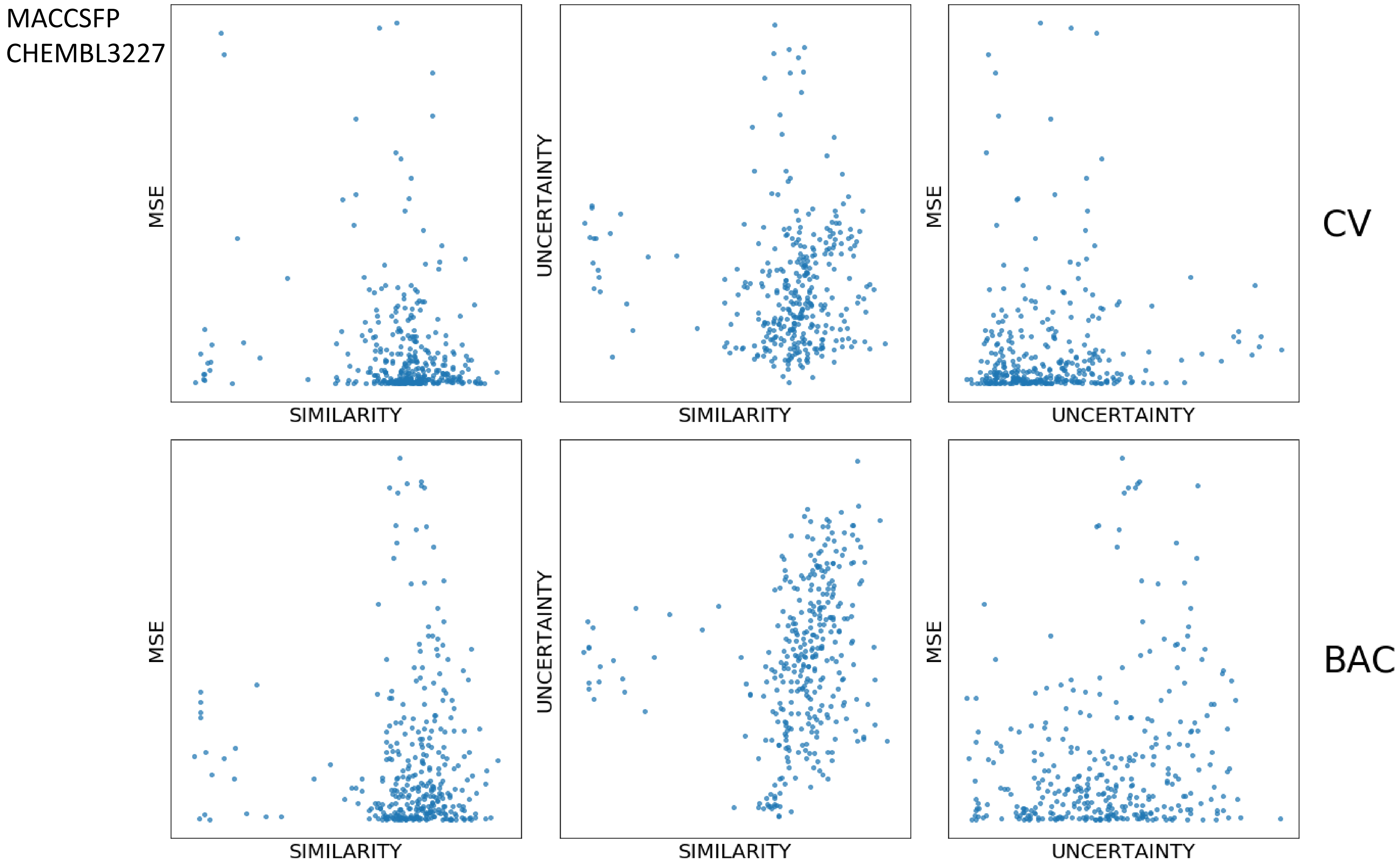
MACCSFP
CHEMBL3227
MSE
SIMILARITY
UNCERTAINTY
SIMILARITY
MSE
UNCERTAINTY
CV
MSE
SIMILARITY
UNCERTAINTY
SIMILARITY
MSE
UNCERTAINTY
BAC

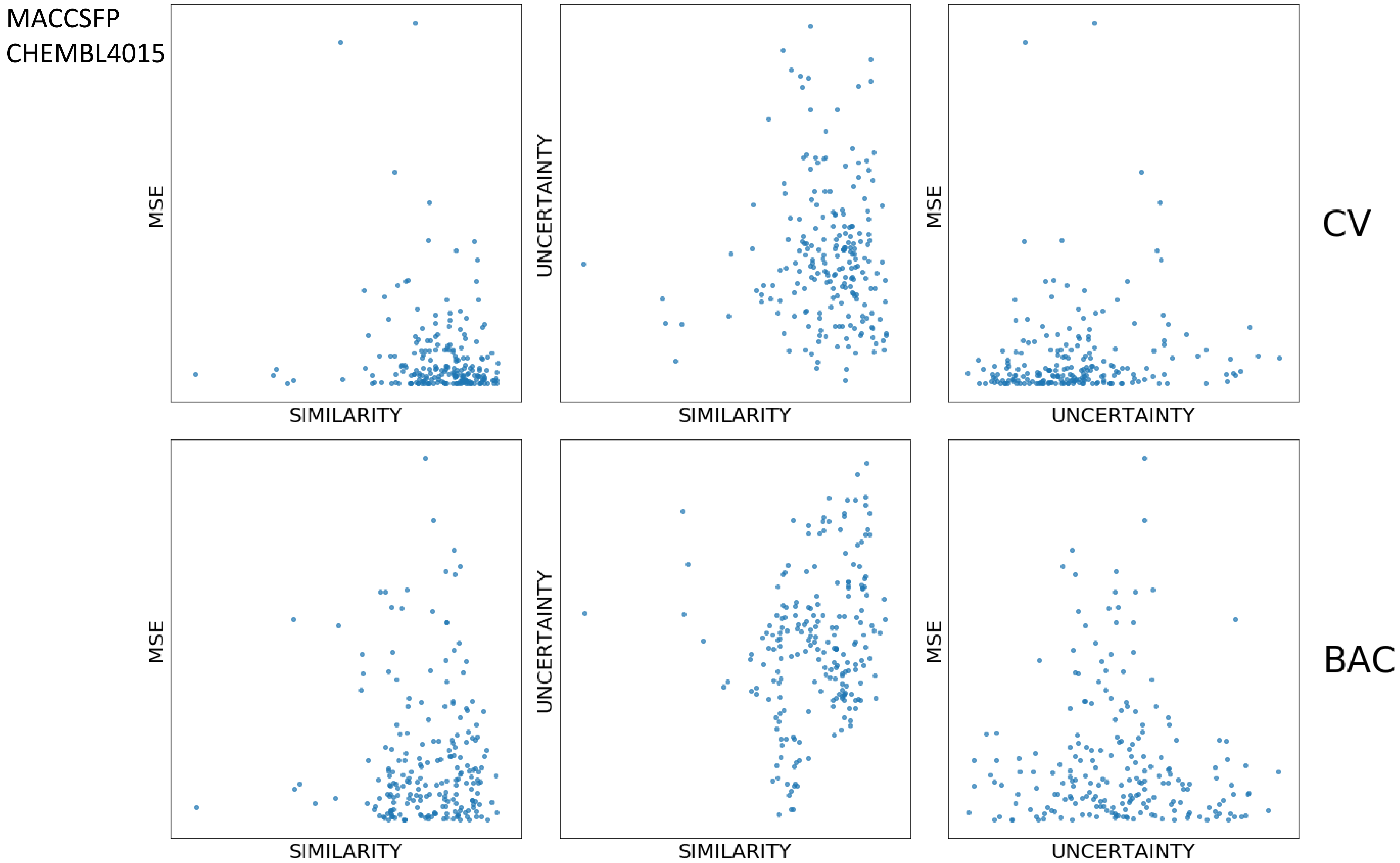
MACCSFP
CHEMBL4015
MSE
SIMILARITY
UNCERTAINTY
SIMILARITY
MSE
UNCERTAINTY
CV
MSE
SIMILARITY
UNCERTAINTY
SIMILARITY
MSE
UNCERTAINTY
BAC

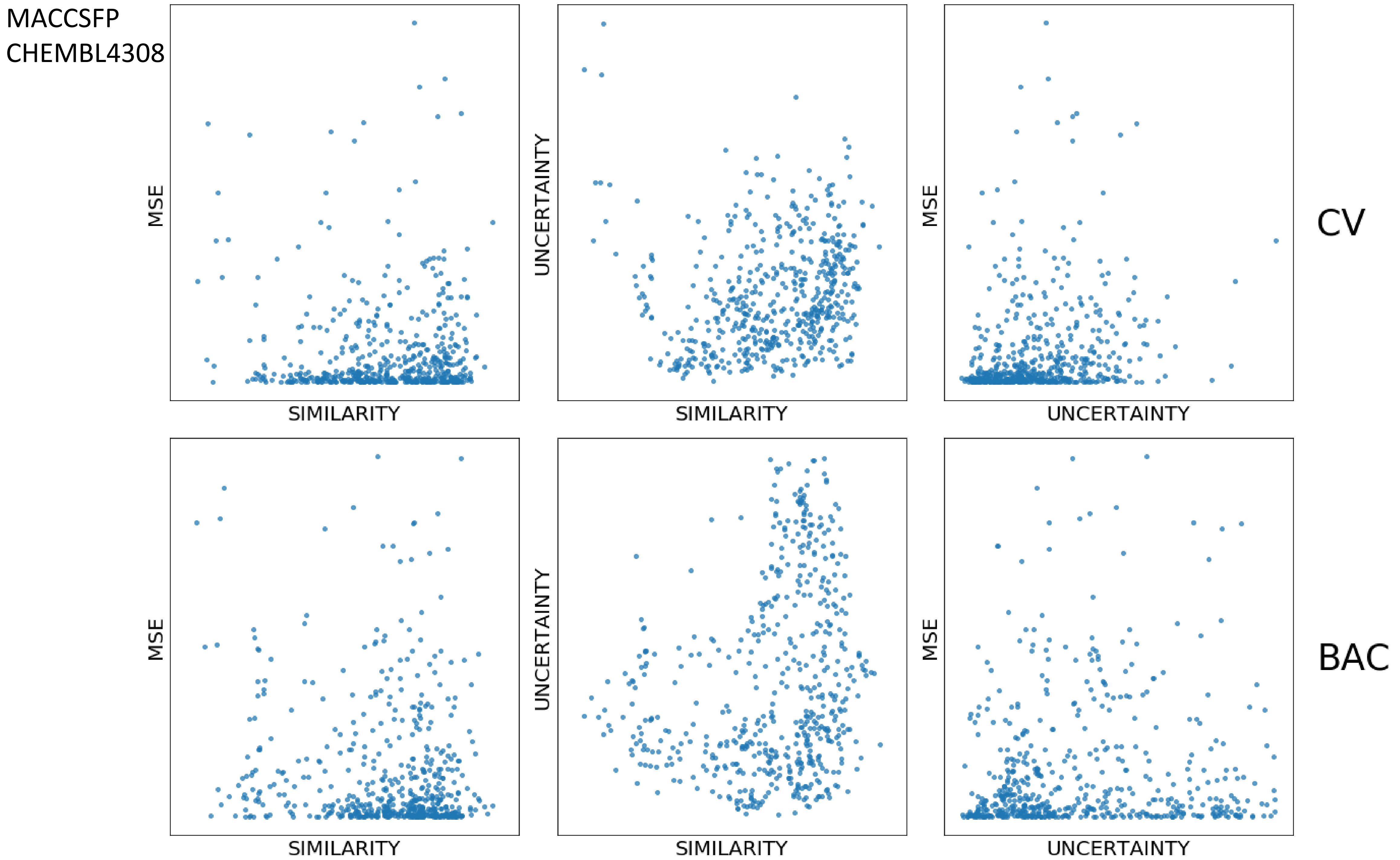
MACCSFP
CHEMBL4308
MSE
SIMILARITY
UNCERTAINTY
SIMILARITY
MSE
UNCERTAINTY
CV
MSE
SIMILARITY
UNCERTAINTY
SIMILARITY
MSE
UNCERTAINTY
BAC

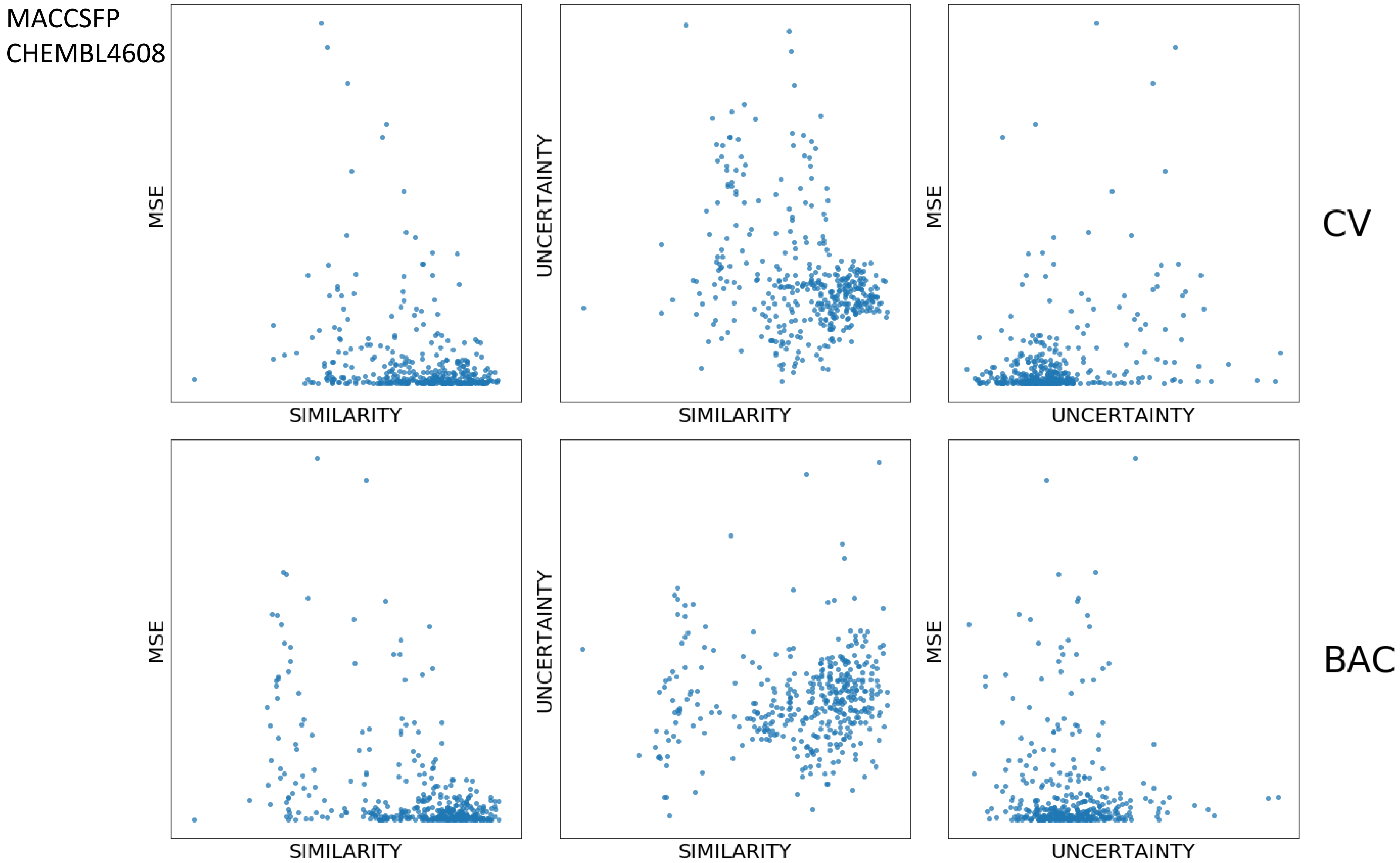
MACCSFP
CHEMBL4608
MSE
SIMILARITY
UNCERTAINTY
SIMILARITY
MSE
UNCERTAINTY
CV
MSE
SIMILARITY
UNCERTAINTY
SIMILARITY
MSE
UNCERTAINTY
BAC

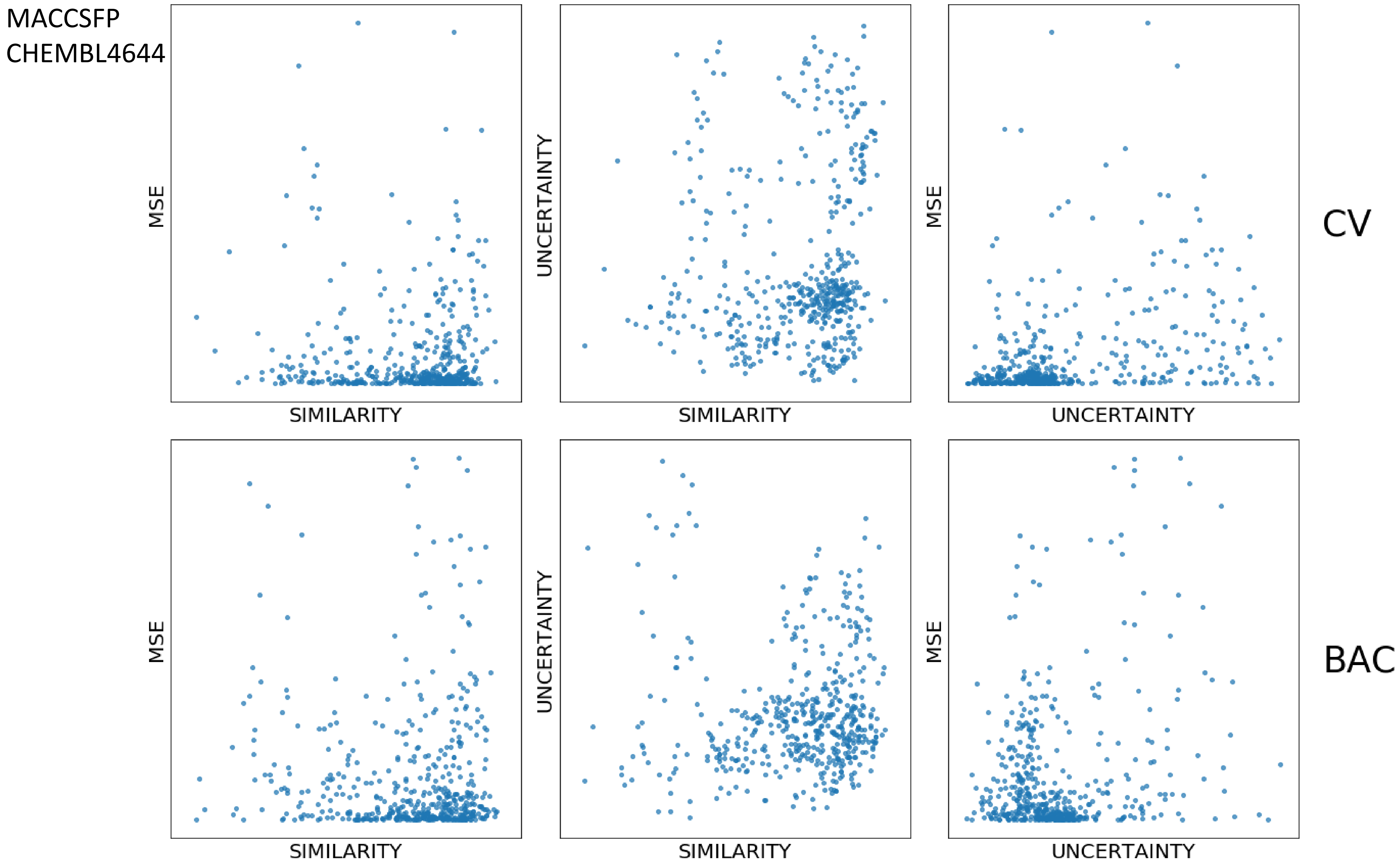
MACCSFP
CHEMBL4644
MSE
SIMILARITY
UNCERTAINTY
SIMILARITY
MSE
UNCERTAINTY
CV
MSE
SIMILARITY
UNCERTAINTY
SIMILARITY
MSE
UNCERTAINTY
BAC

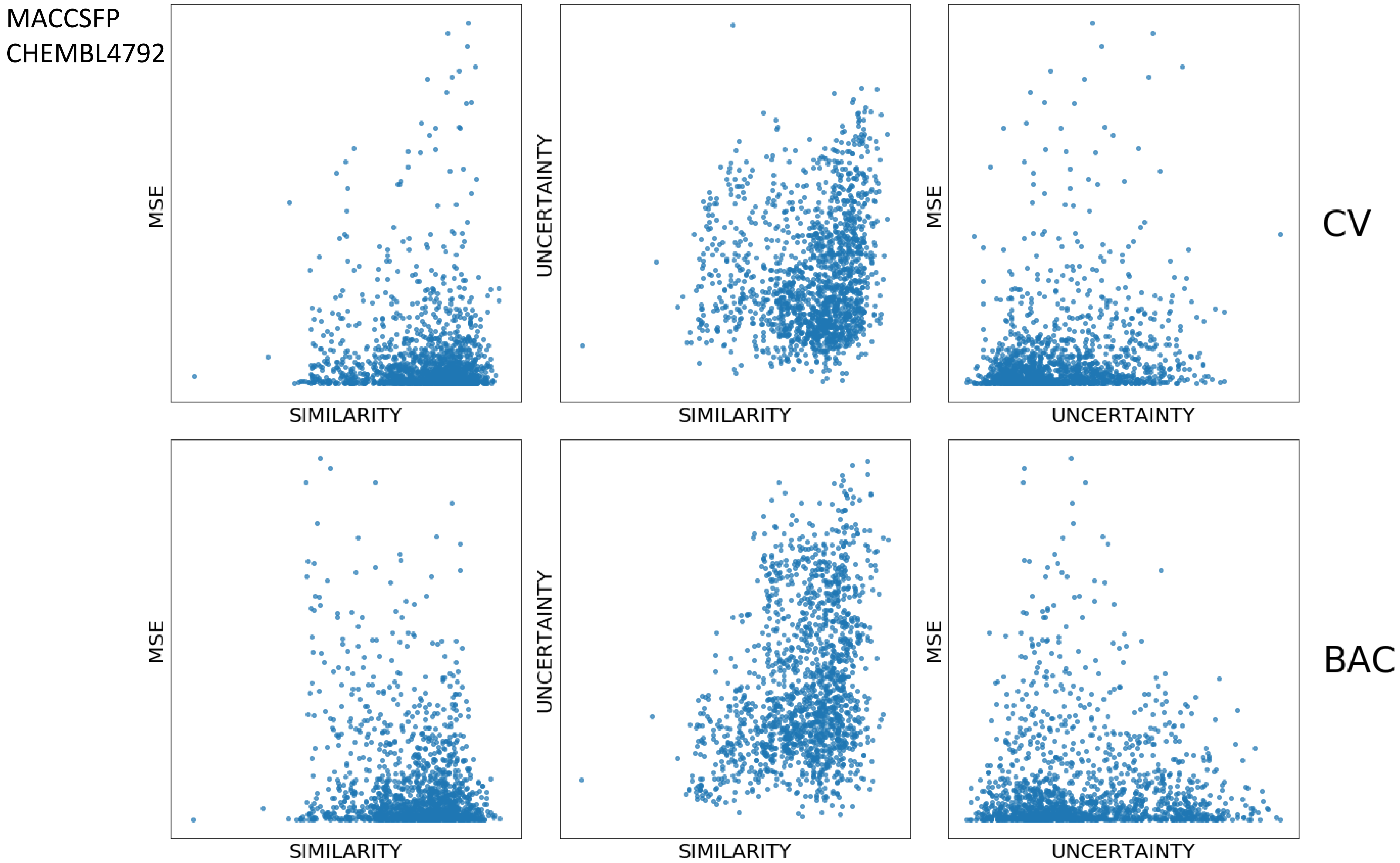
MACCSFP
CHEMBL4792
MSE
SIMILARITY
UNCERTAINTY
SIMILARITY
MSE
UNCERTAINTY
CV
MSE
SIMILARITY
UNCERTAINTY
SIMILARITY
MSE
UNCERTAINTY
BAC

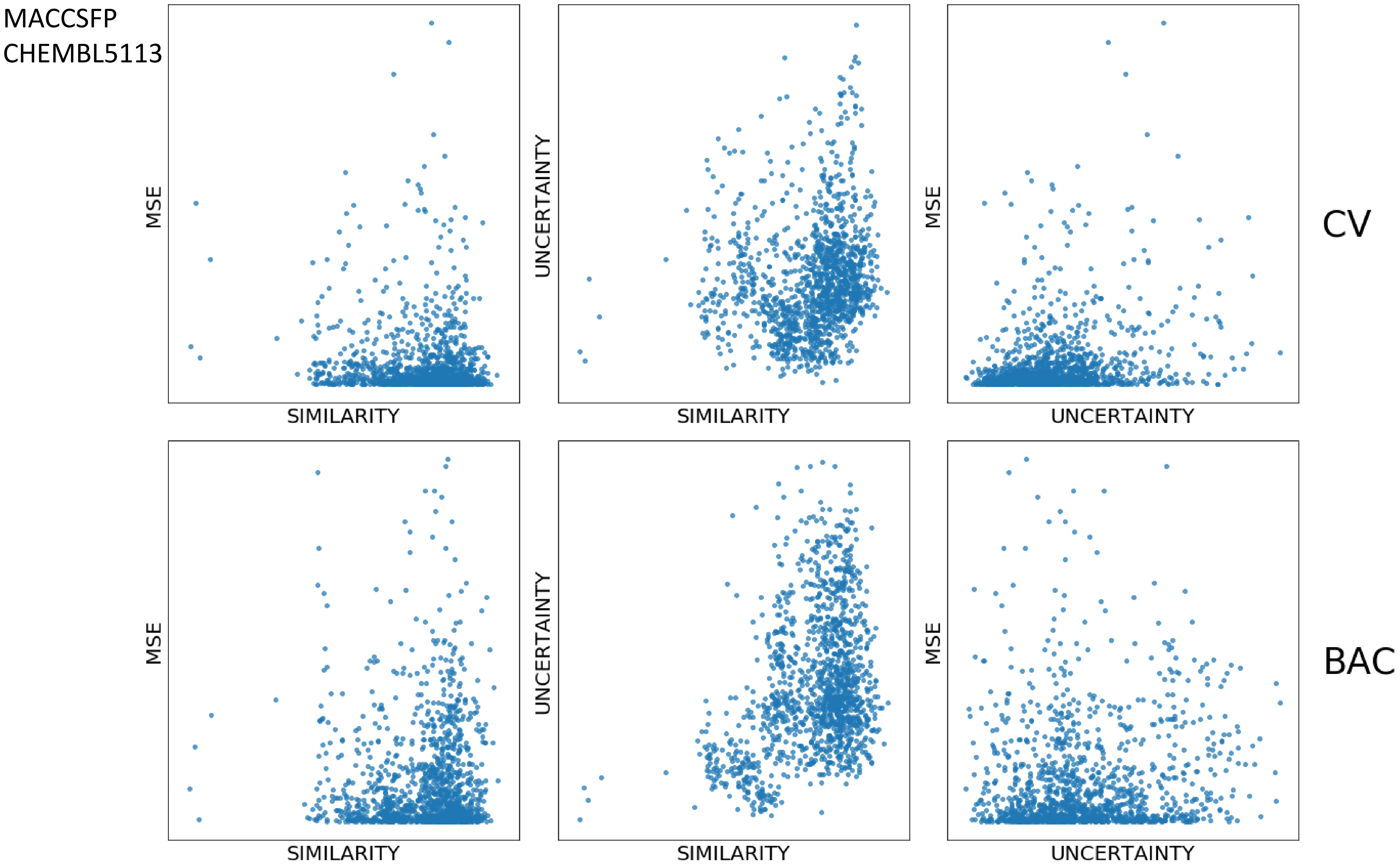

MACCSFP
CHEMBL5113
MSE
SIMILARITY
UNCERTAINTY
SIMILARITY
MSE
UNCERTAINTY
CV
MSE
SIMILARITY
UNCERTAINTY
SIMILARITY
MSE
UNCERTAINTY
BAC
