# Supplementary material for: How Sure Can We Be about ML Methods-Based Evaluation of Compound Activity: Incorporation of Information about Prediction Uncertainty Using Deep Learning Techniques
Source: Molecules. 2020 Mar 23;25(6):1452. doi: 10.3390/molecules25061452 (PMC7144469; doi:10.3390/molecules25061452)
Supplement: Supplementary file 1 [file molecules-25-01452-s001.zip › Supp_Info_for_submission/FileS4.pdf]

CHEMBL214 hashed1024-morgan-4

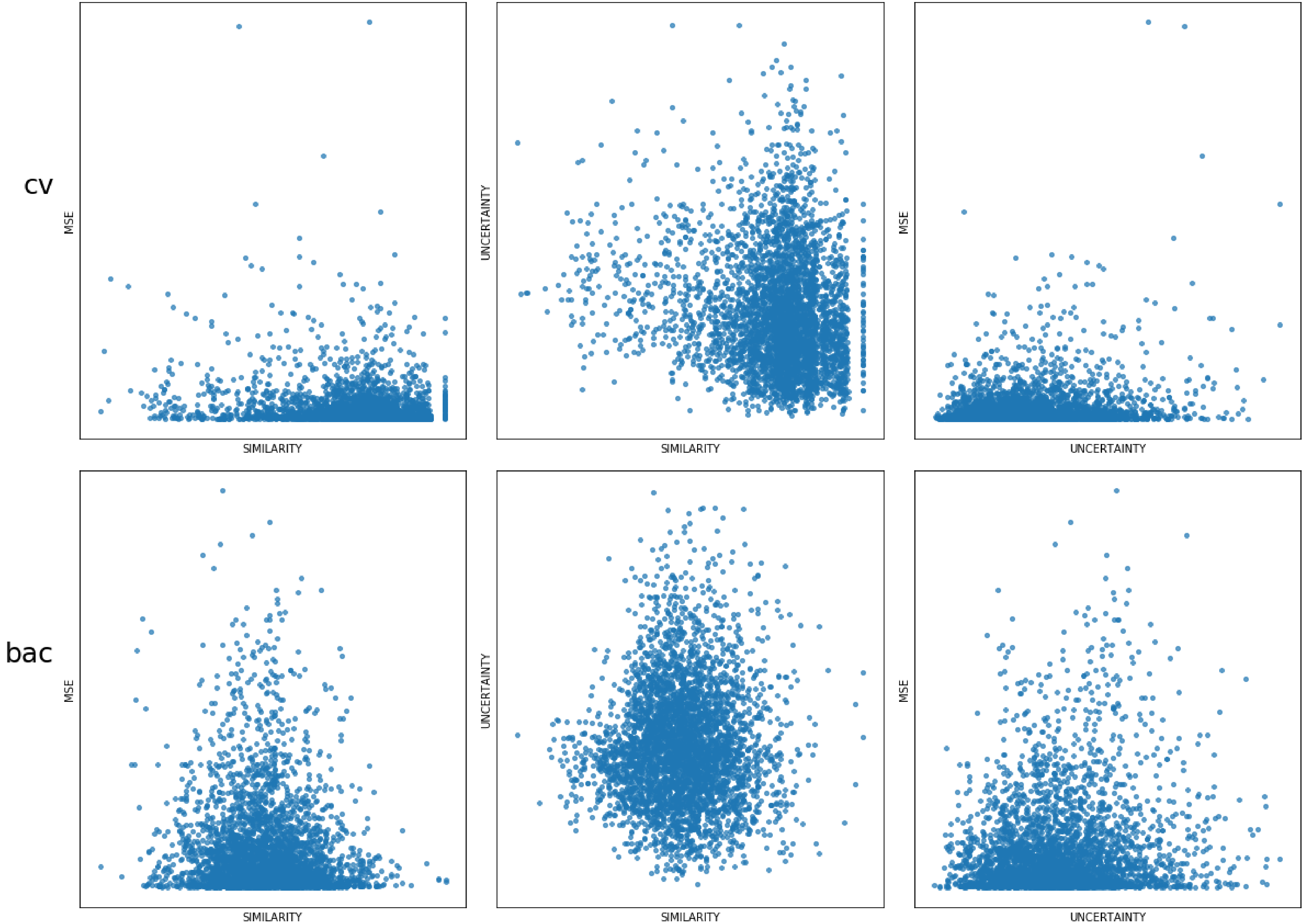

CHEMBL216 hashed1024-morgan-4

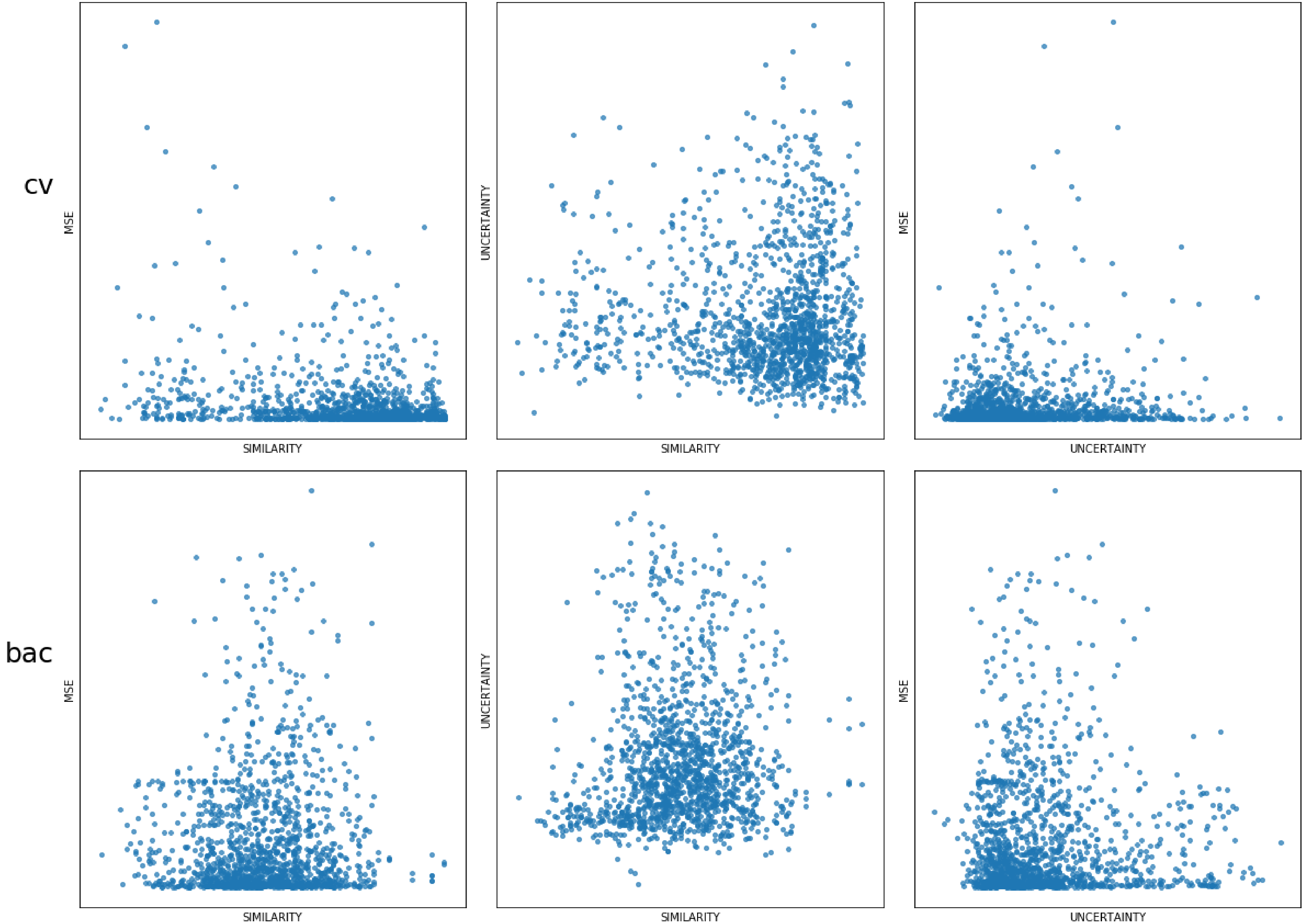

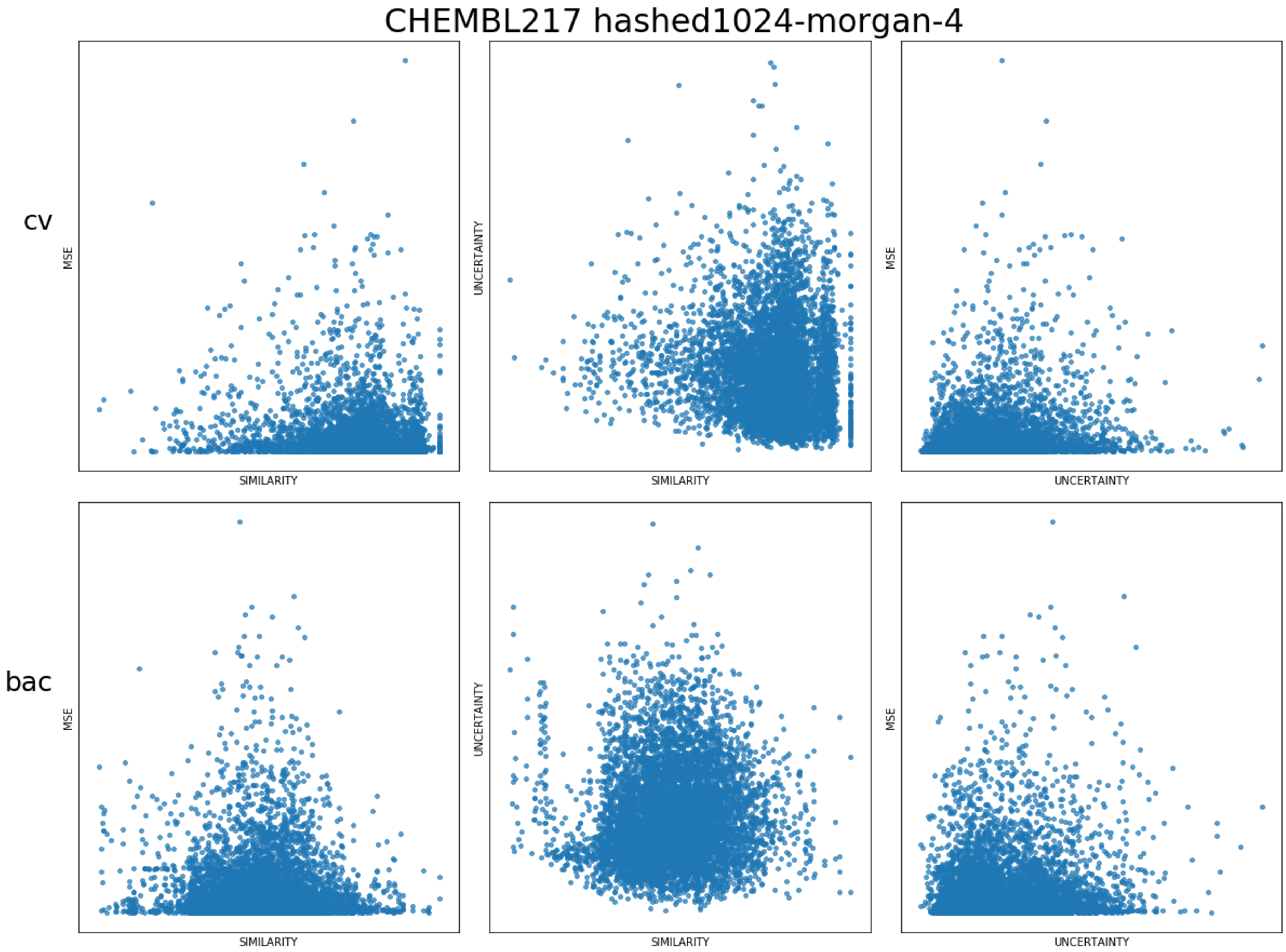
CHEMBL217 hashed1024-morgan-4
cv
bac
MSE
UNCERTAINTY
SIMILARITY

CHEMBL224 hashed1024-morgan-4

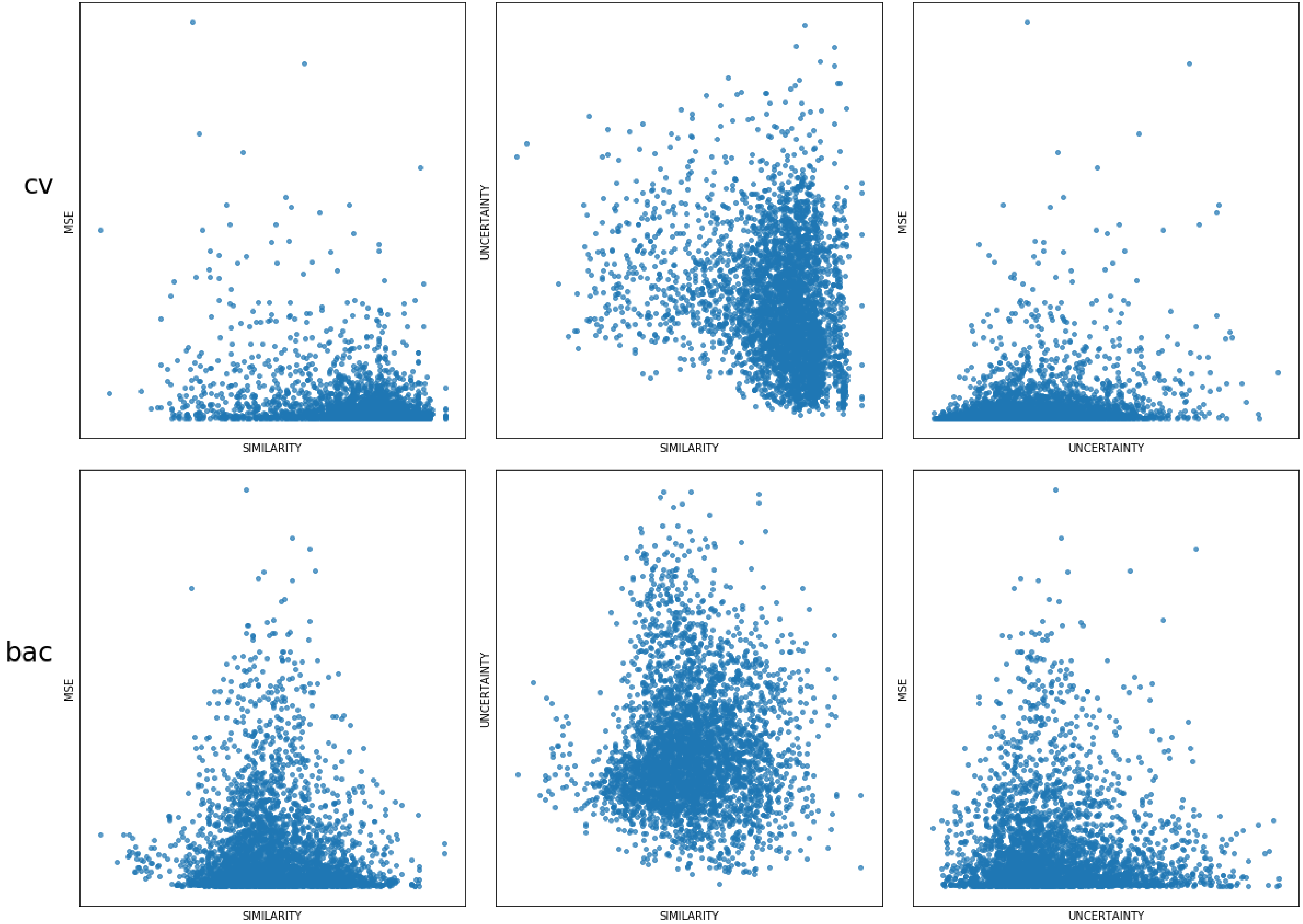

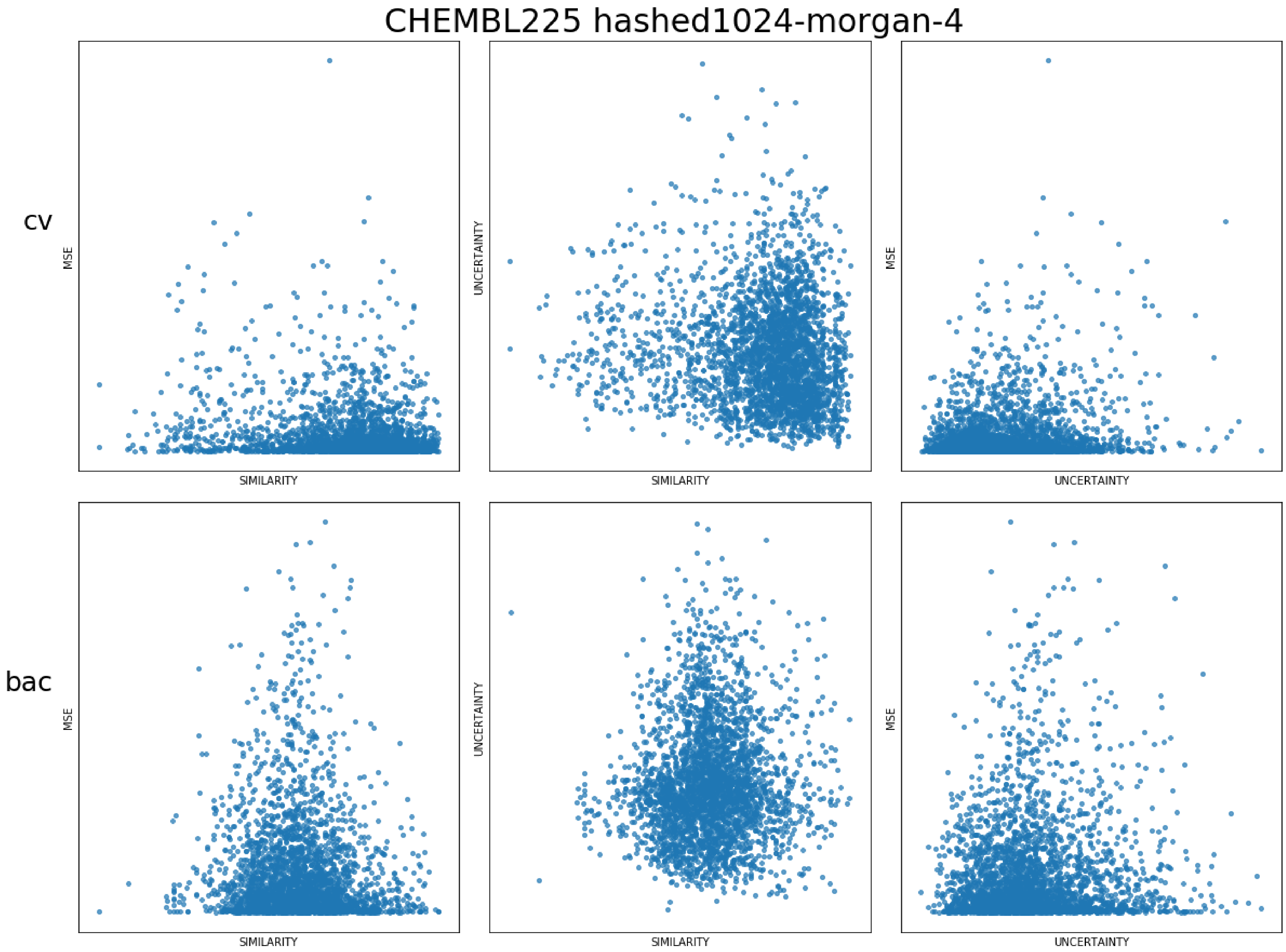
CHEMBL225 hashed1024-morgan-4
cv
bac
MSE
UNCERTAINTY
SIMILARITY

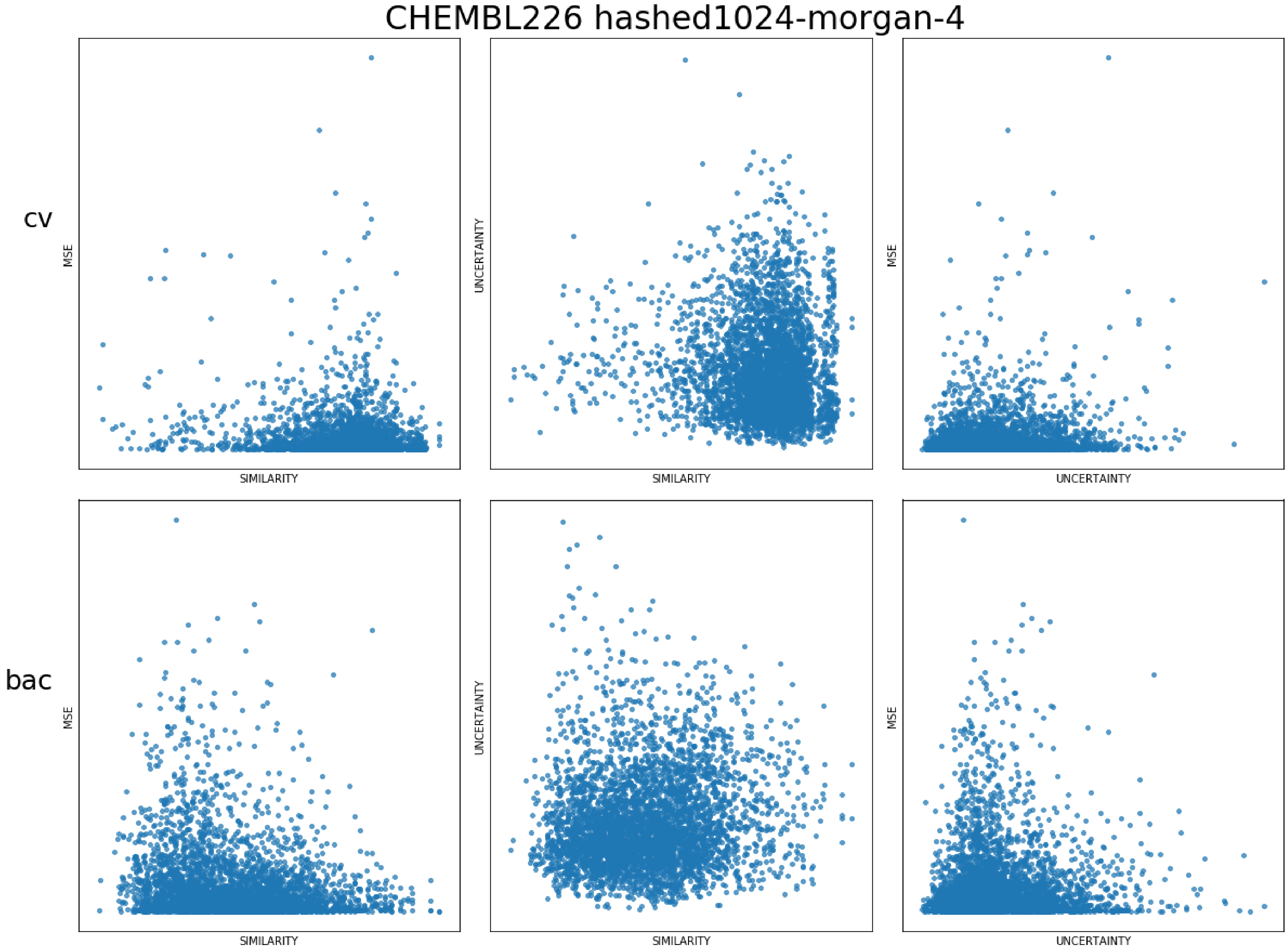
CHEMBL226 hashed1024-morgan-4
cv
bac
MSE
UNCERTAINTY
SIMILARITY

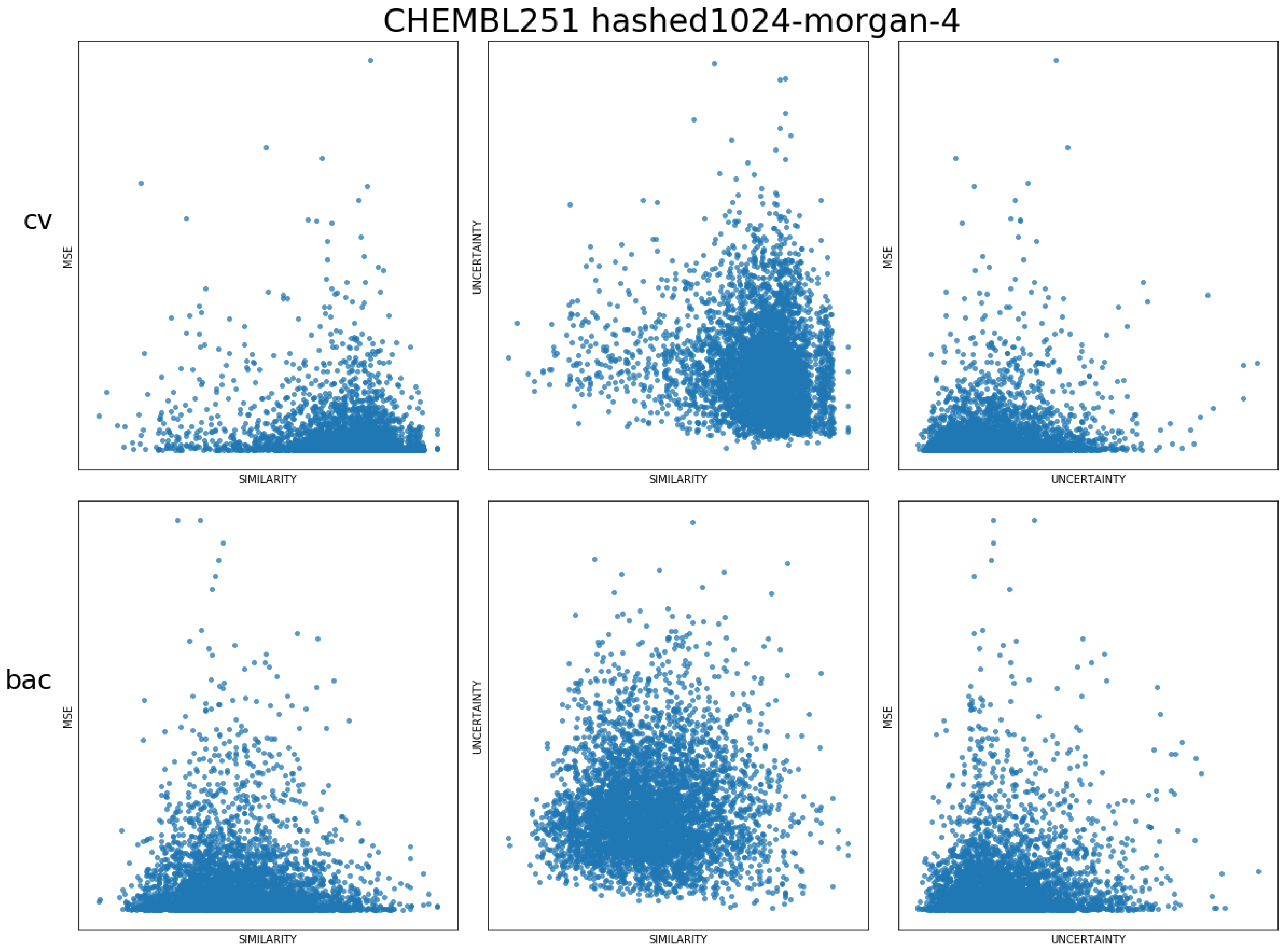
CHEMBL251 hashed1024-morgan-4
cv
bac
MSE
UNCERTAINTY
SIMILARITY

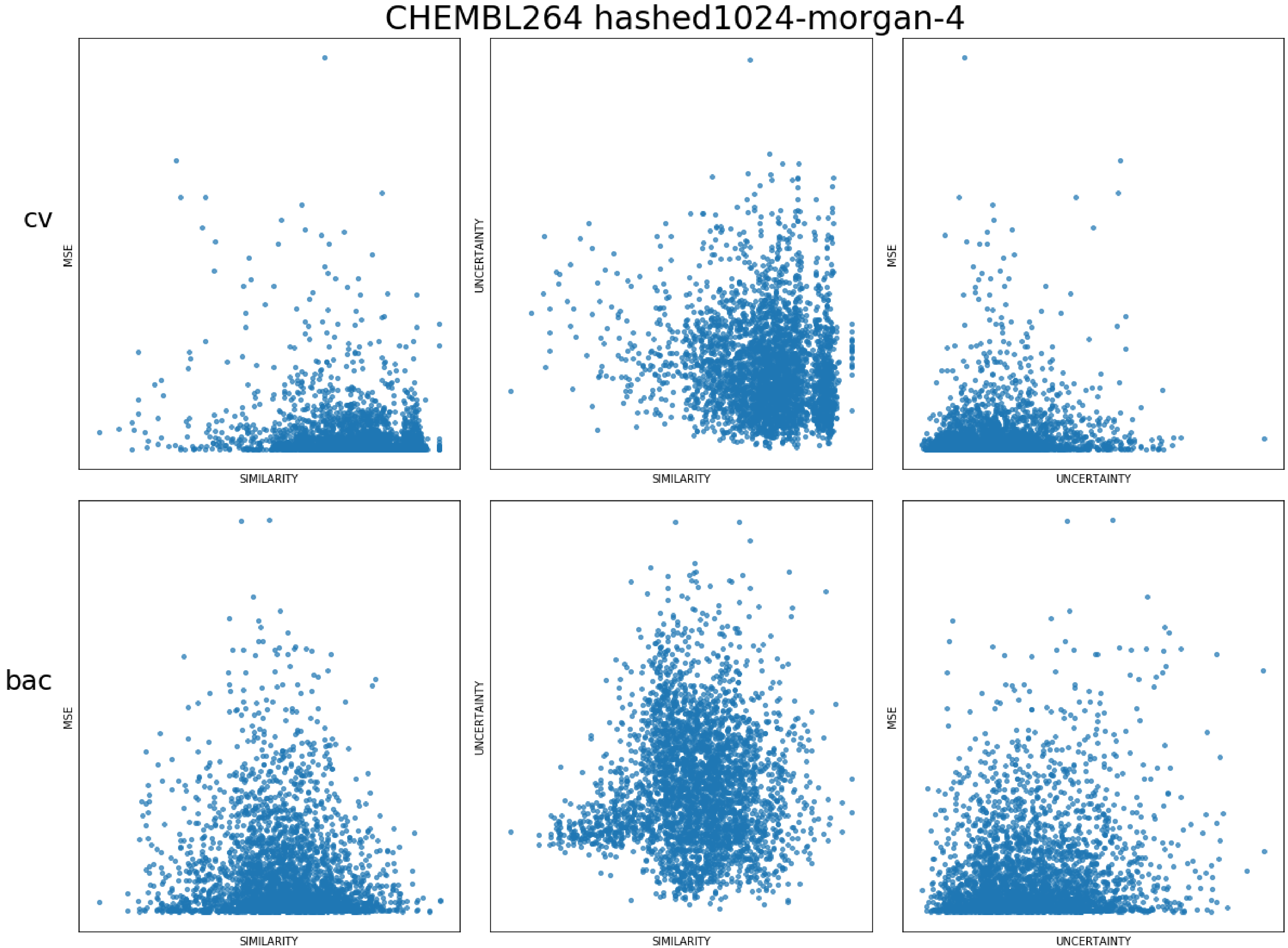
CHEMBL264 hashed1024-morgan-4
cv
bac
MSE
UNCERTAINTY
SIMILARITY

CHEMBL3155 hashed1024-morgan-4

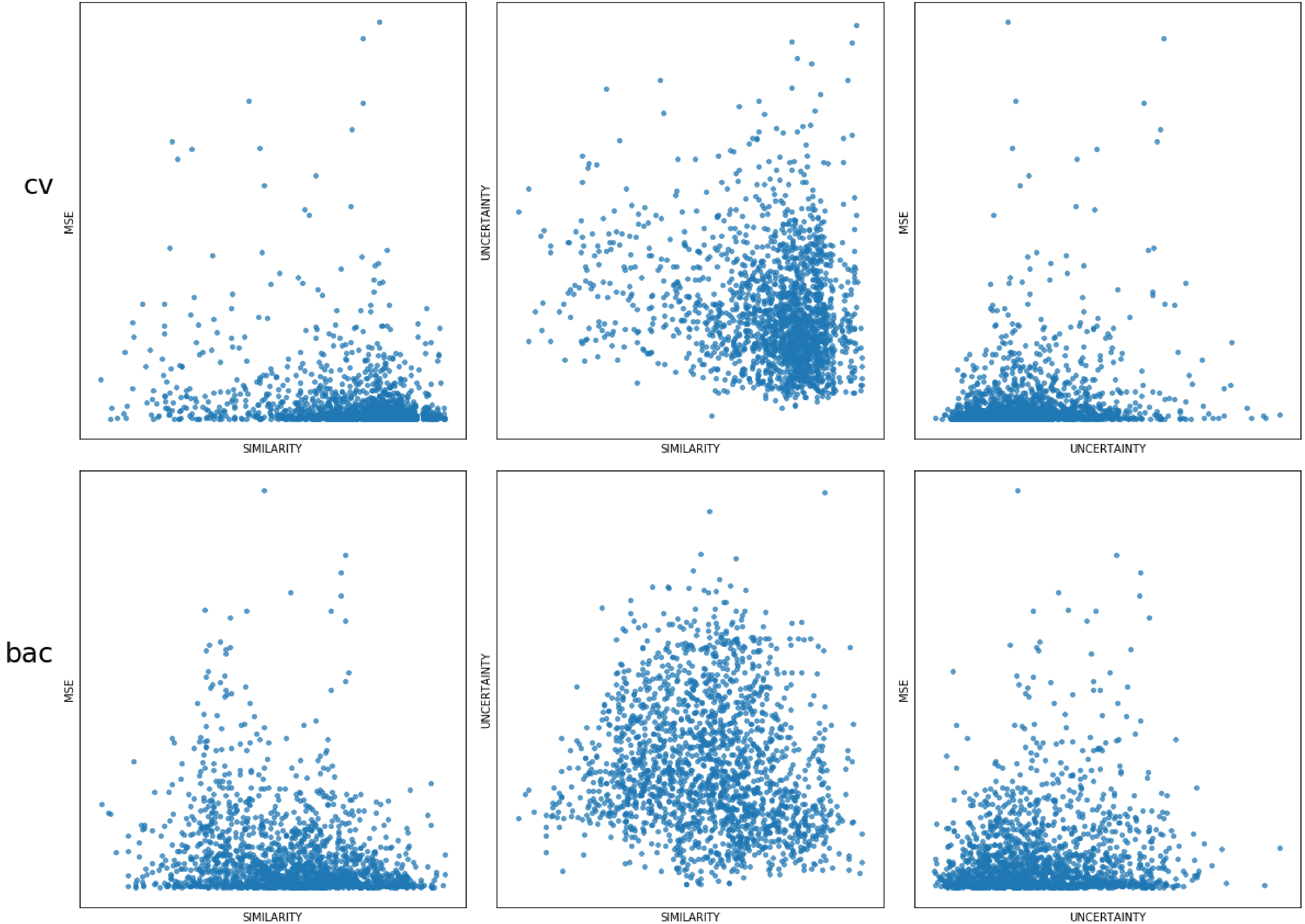

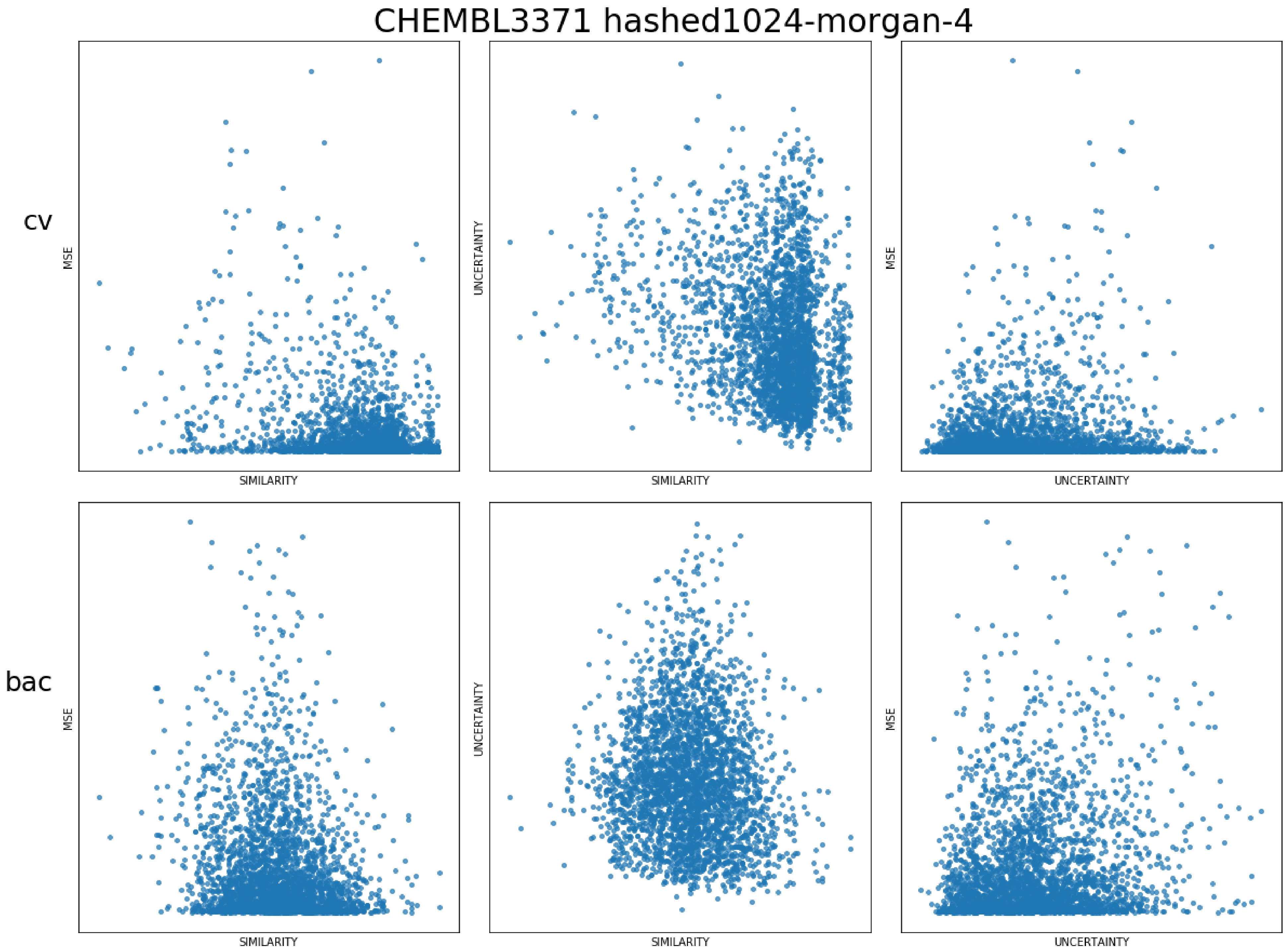
CHEMBL3371 hashed1024-morgan-4
cv
bac
MSE
UNCERTAINTY
SIMILARITY

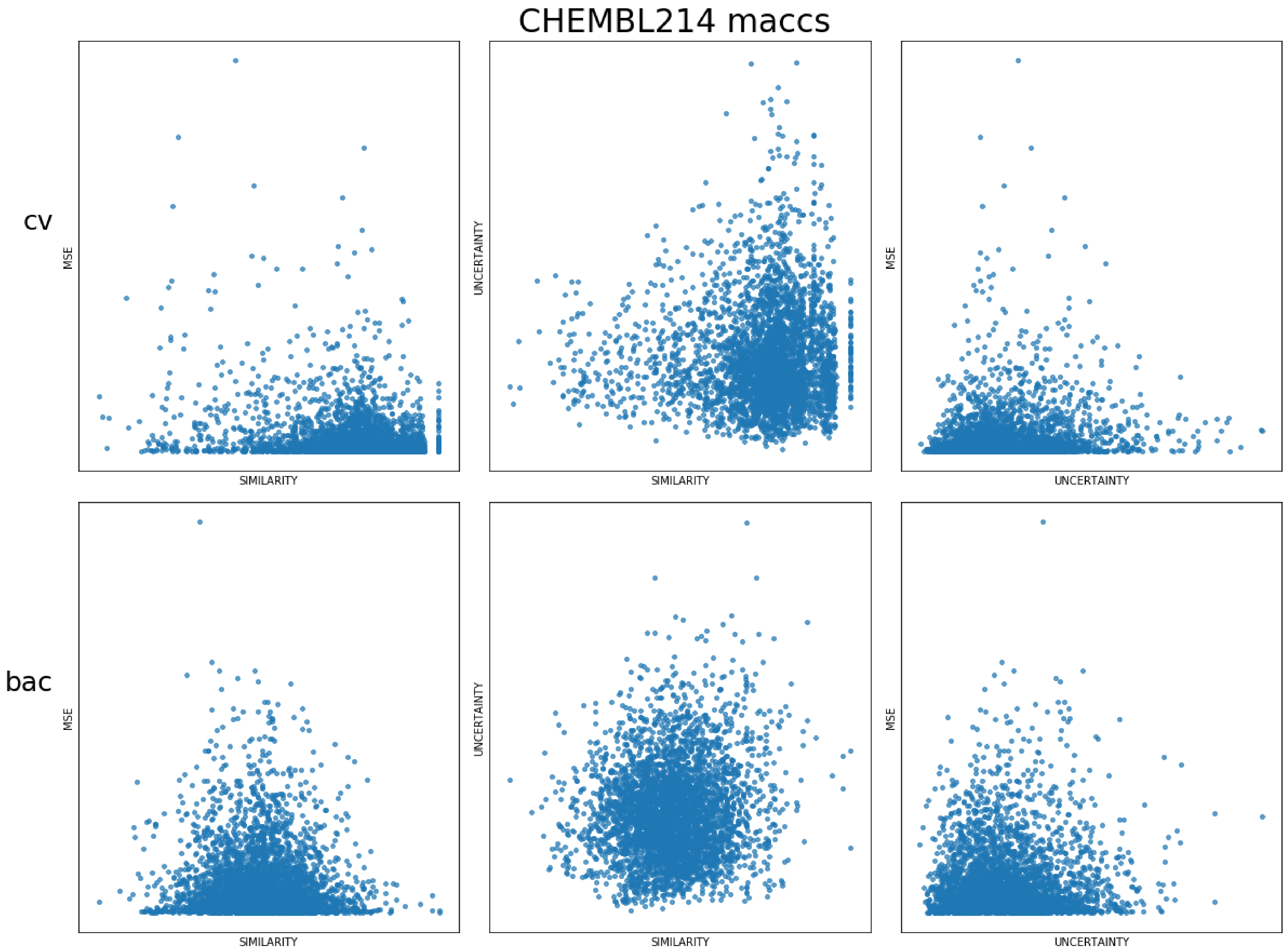
CHEMBL214 maccs
cv
bac
MSE
UNCERTAINTY
SIMILARITY

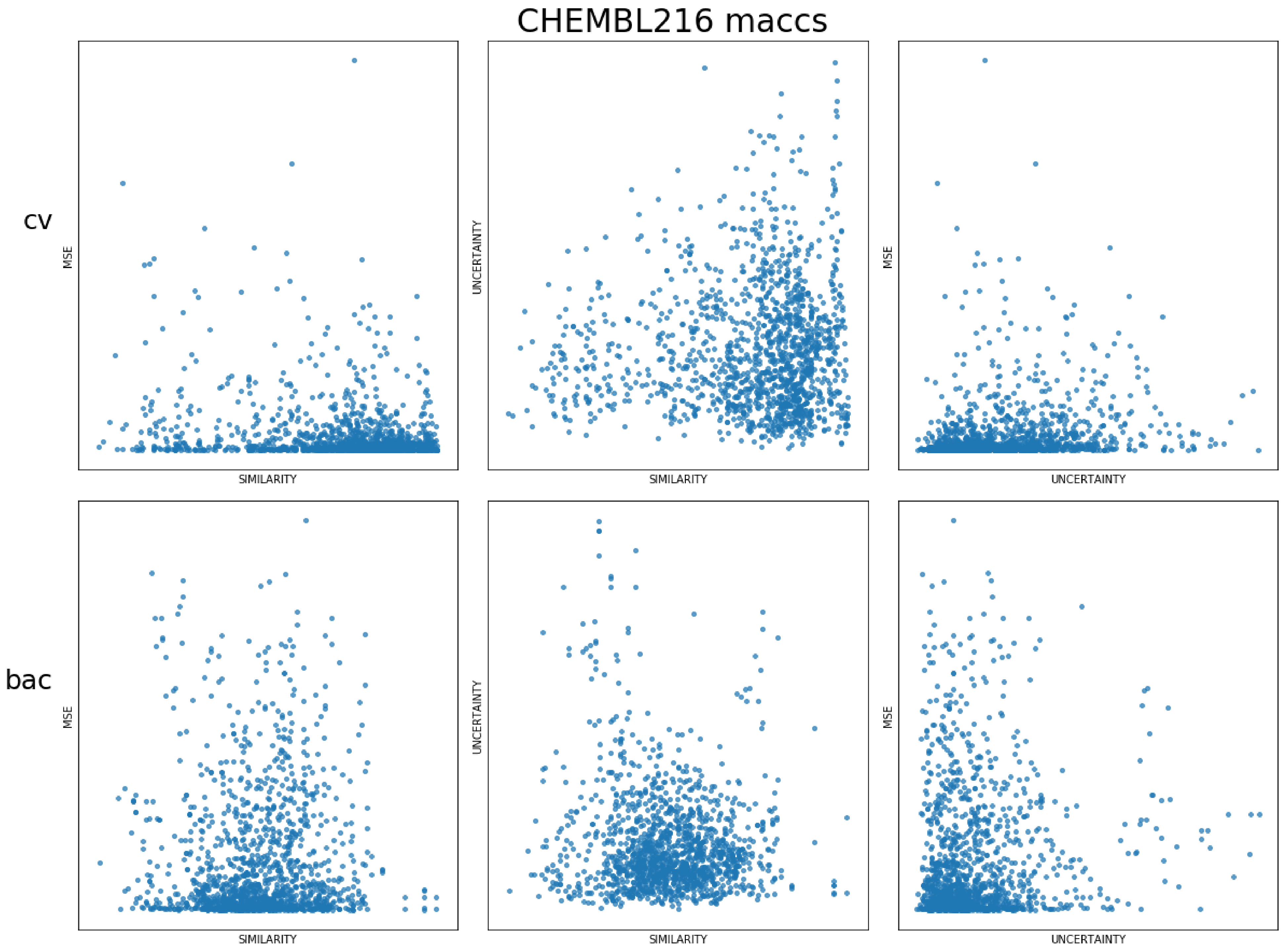
CHEMBL216 maccs
cv
bac
MSE
UNCERTAINTY
SIMILARITY

CHEMBL217 maccs

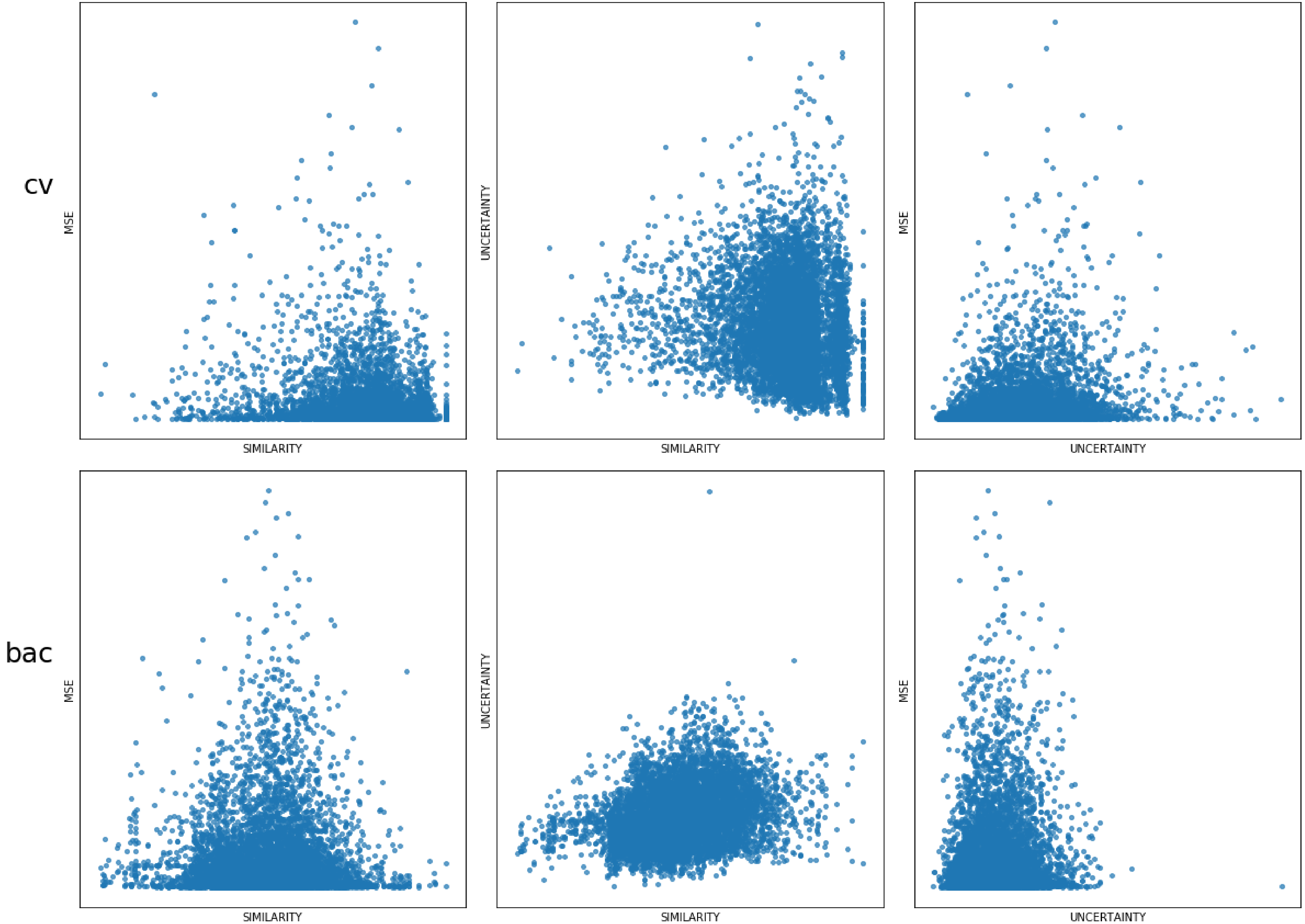

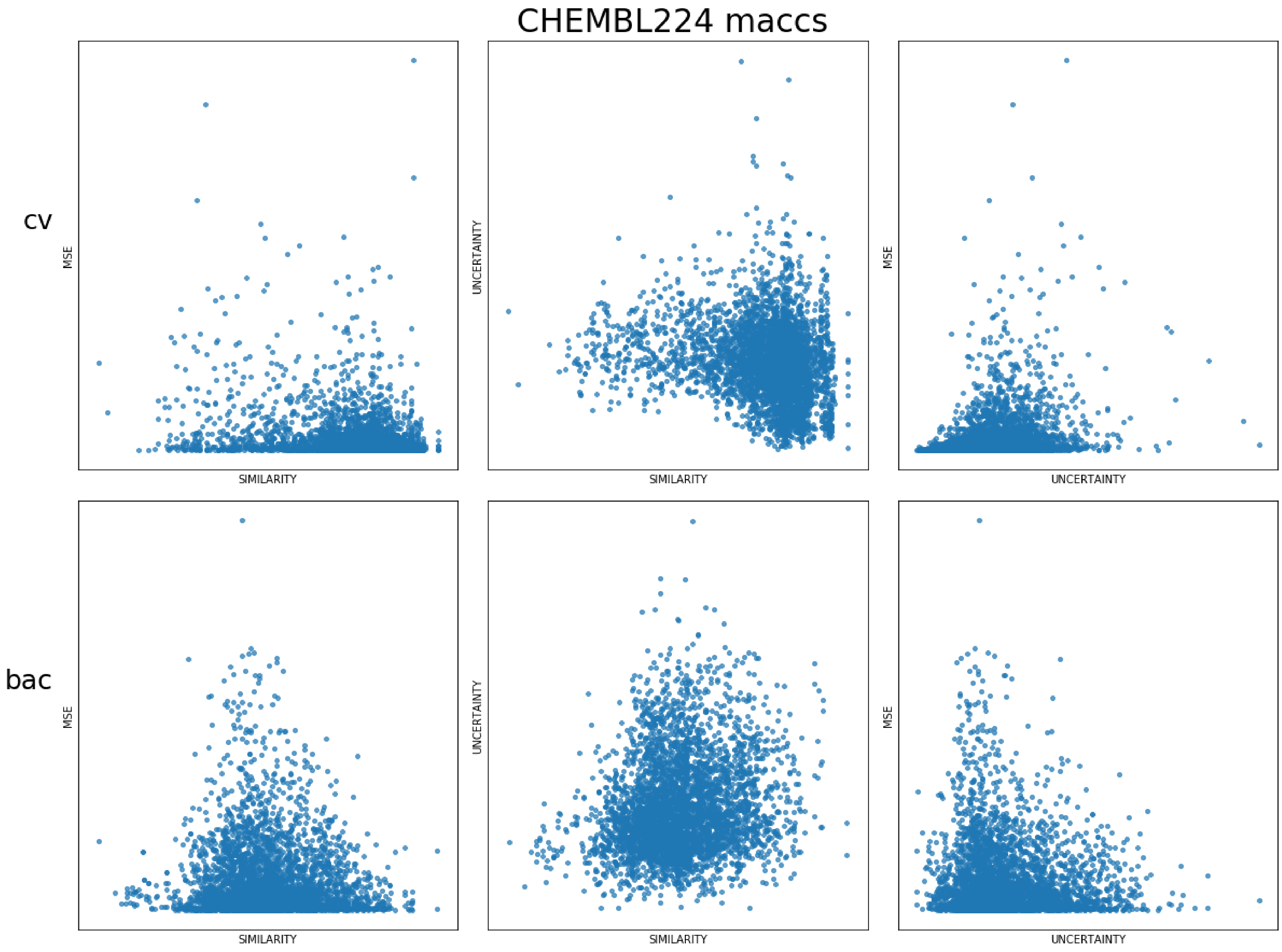
CHEMBL224 maccs
cv
bac
MSE
SIMILARITY
UNCERTAINTY
MSE
SIMILARITY
UNCERTAINTY
MSE
UNCERTAINTY

CHEMBL225 maccs

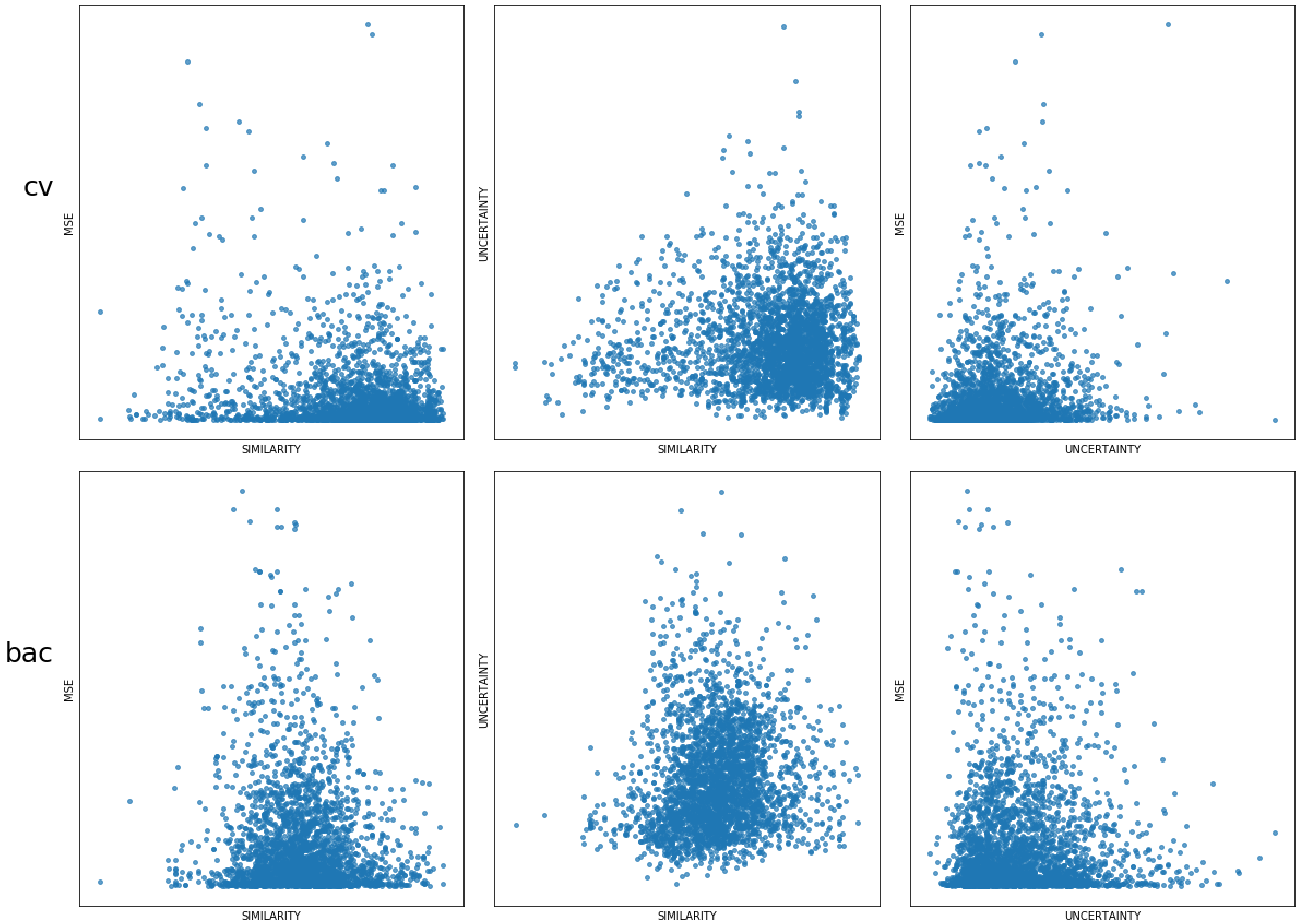

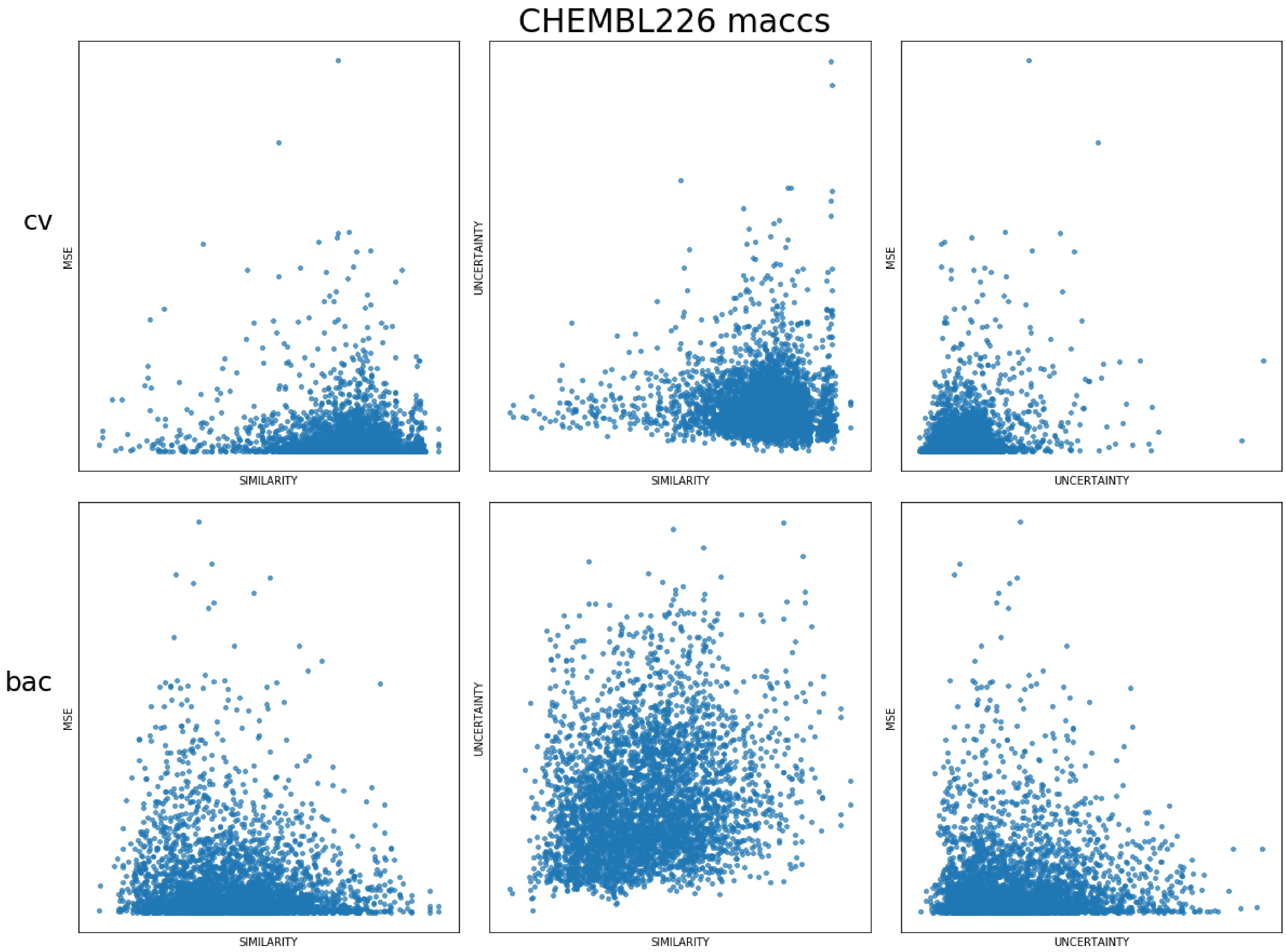
CHEMBL226 maccs
cv
bac
MSE
UNCERTAINTY
SIMILARITY

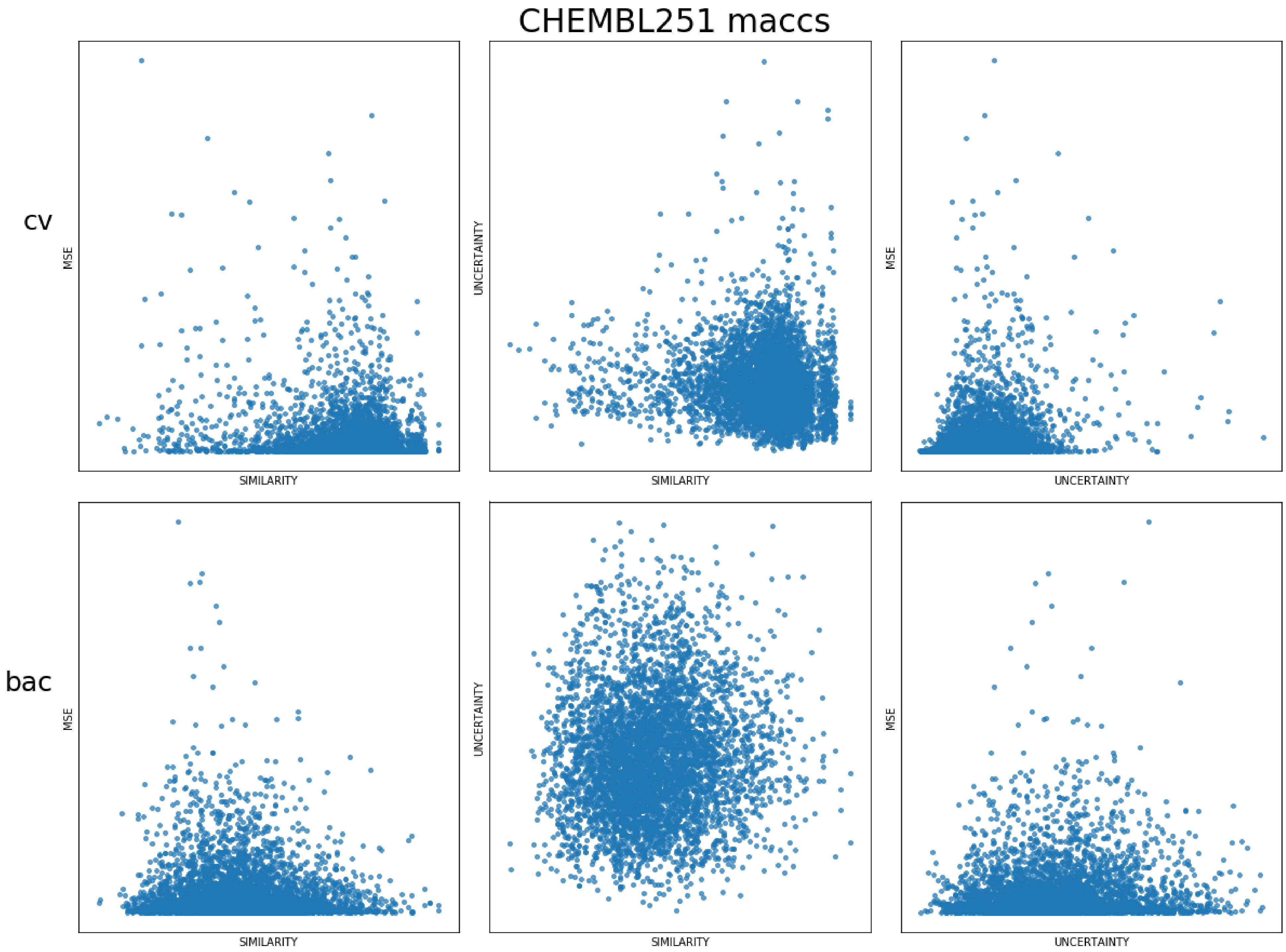
CHEMBL251 maccs
cv
bac
MSE
UNCERTAINTY
SIMILARITY

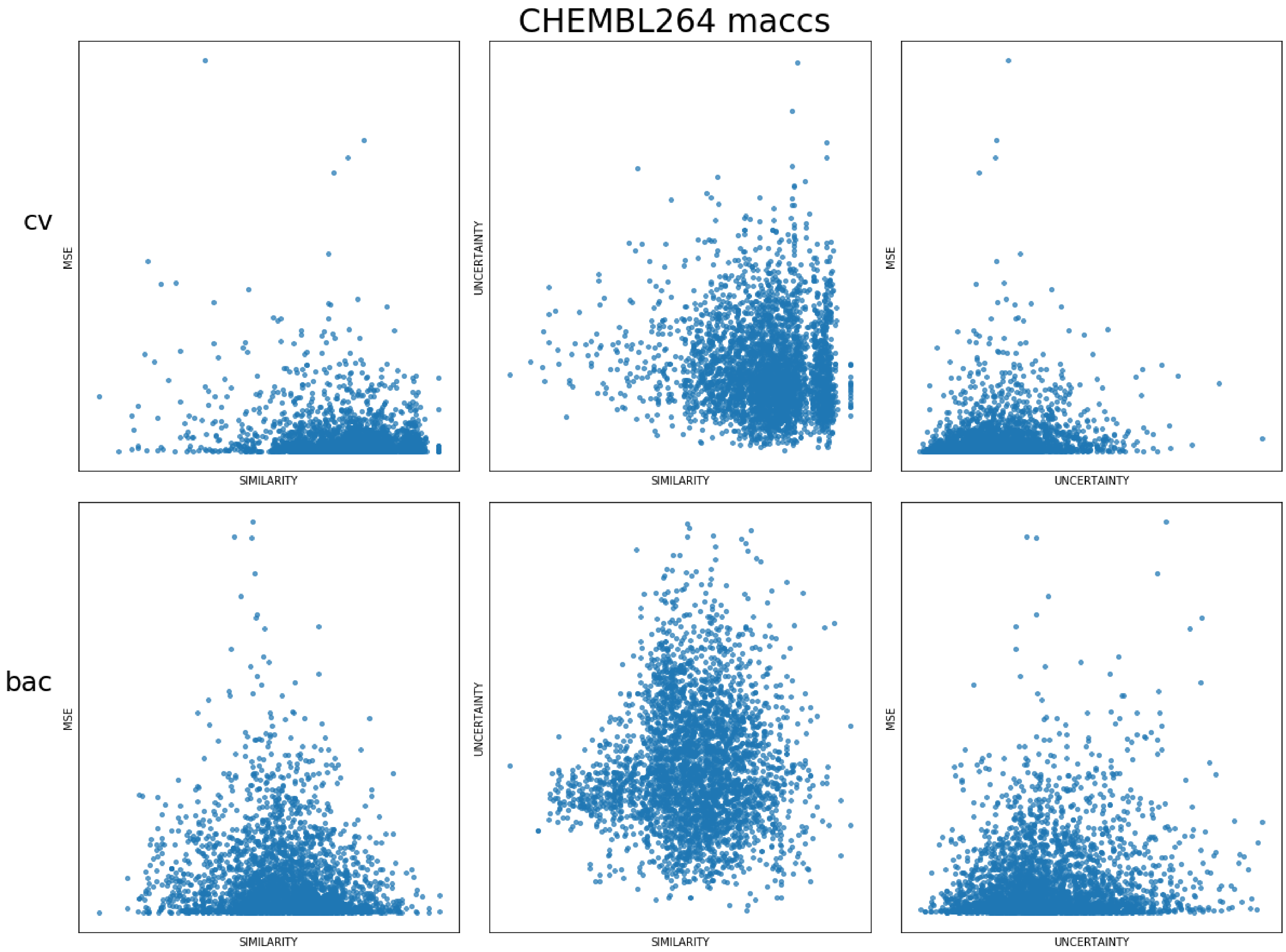
CHEMBL264 maccs
cv
bac
MSE
UNCERTAINTY
SIMILARITY

CHEMBL3155 maccs

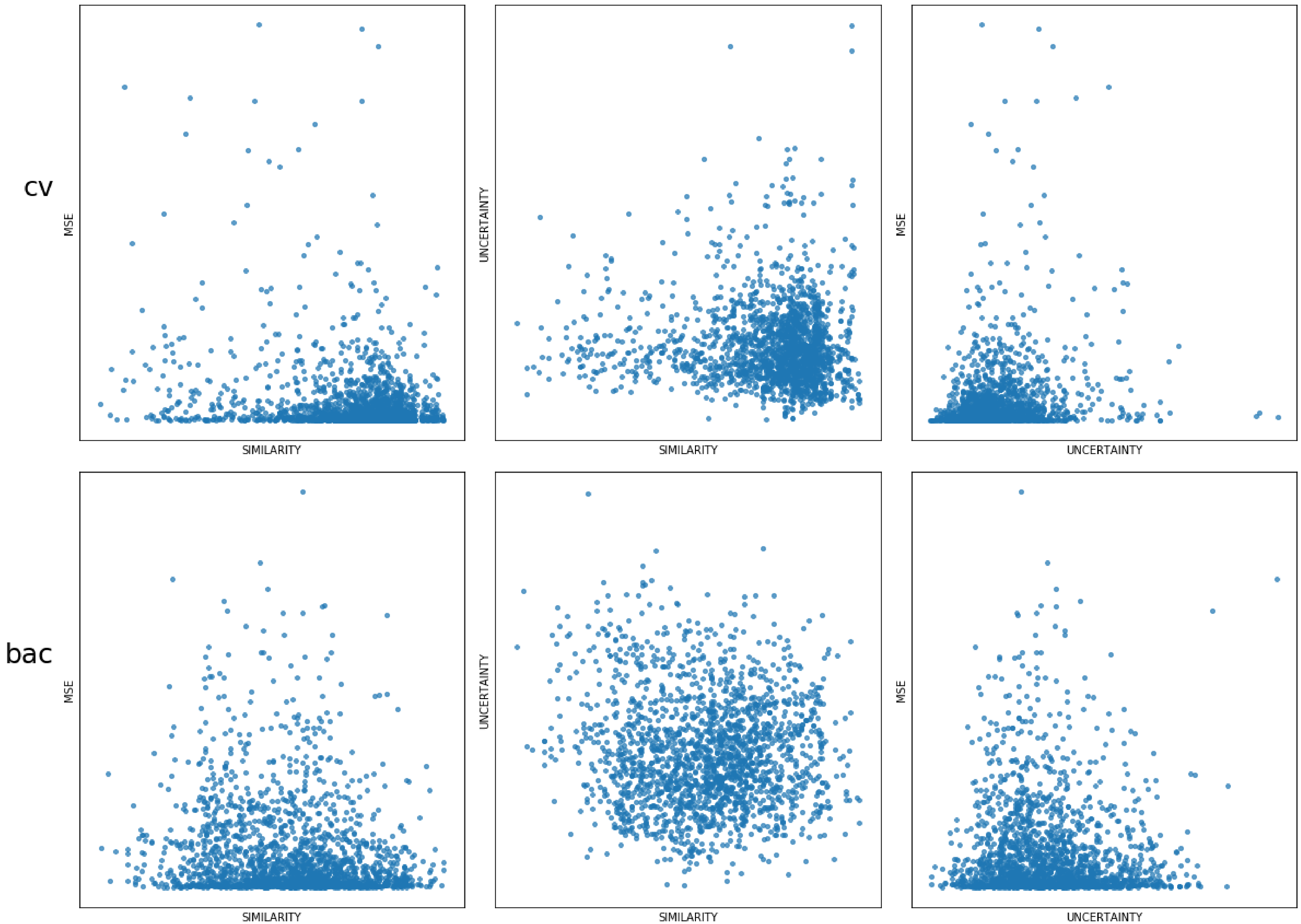

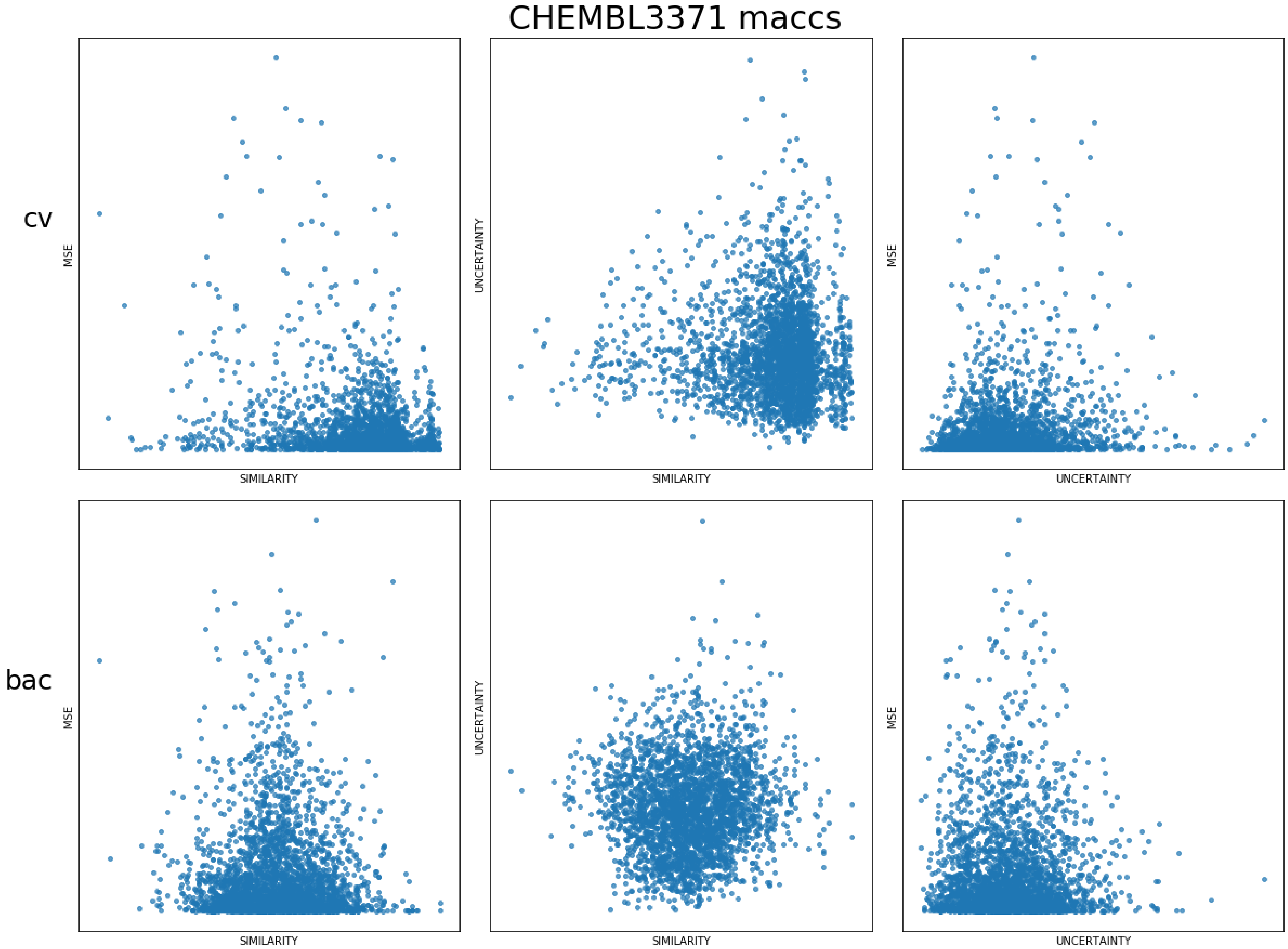
CHEMBL3371 maccs
cv
bac
MSE
UNCERTAINTY
SIMILARITY
