# Supplementary figures and images for: How Sure Can We Be about ML Methods-Based Evaluation of Compound Activity: Incorporation of Information about Prediction Uncertainty Using Deep Learning Techniques
Source: Molecules. 2020 Mar 23;25(6):1452. doi: 10.3390/molecules25061452 (PMC7144469; doi:10.3390/molecules25061452)

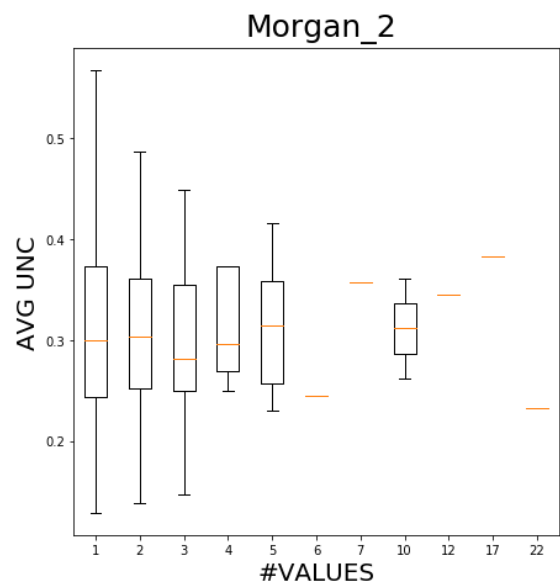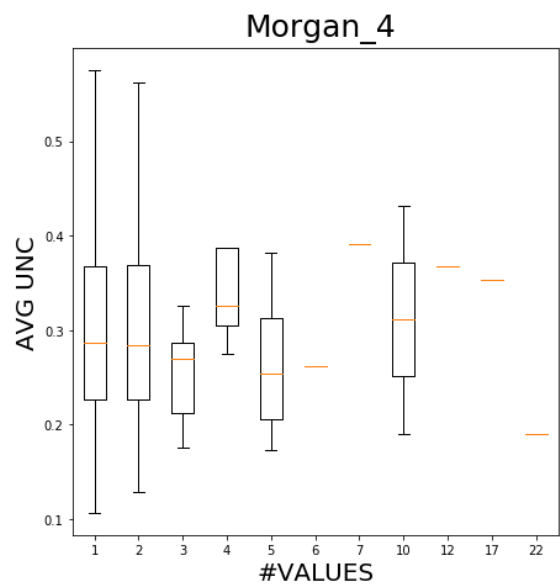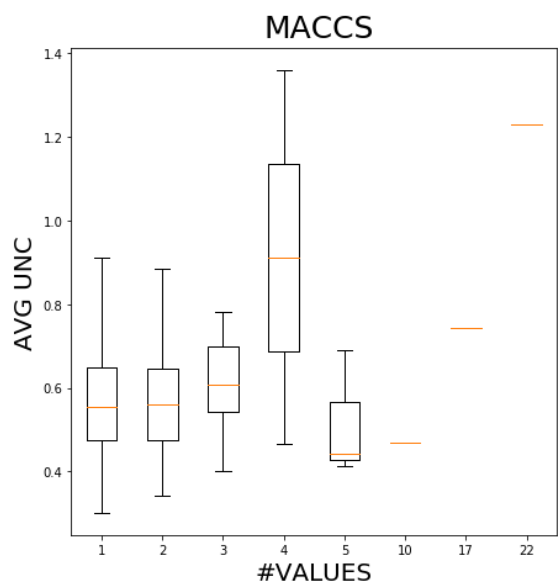

CV

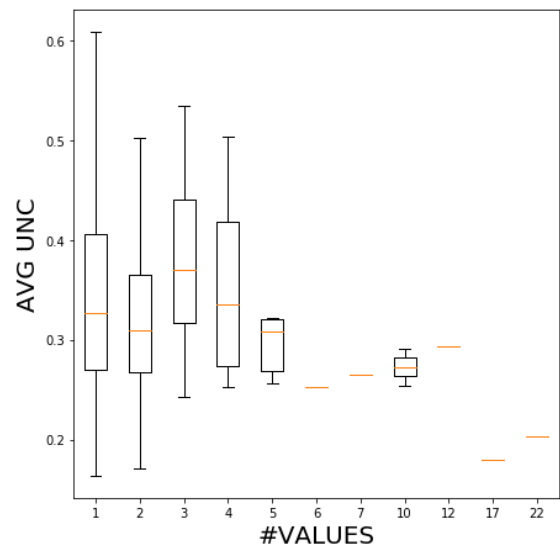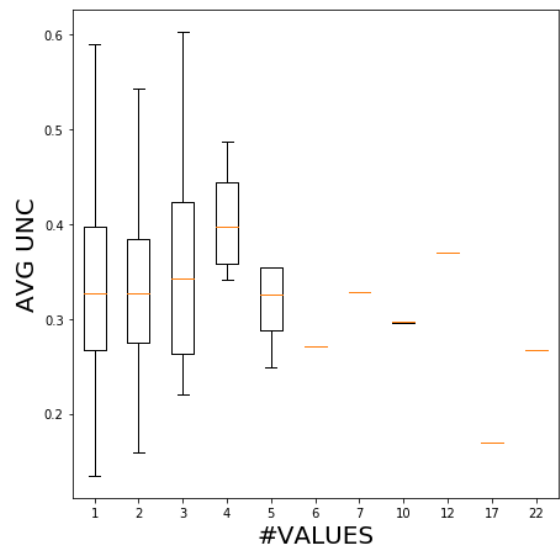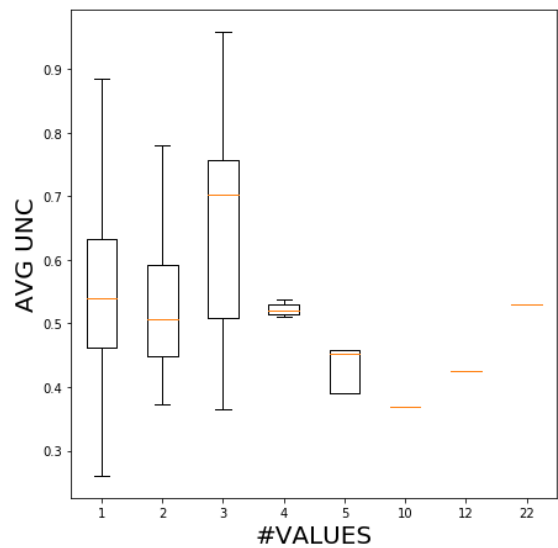

BAC

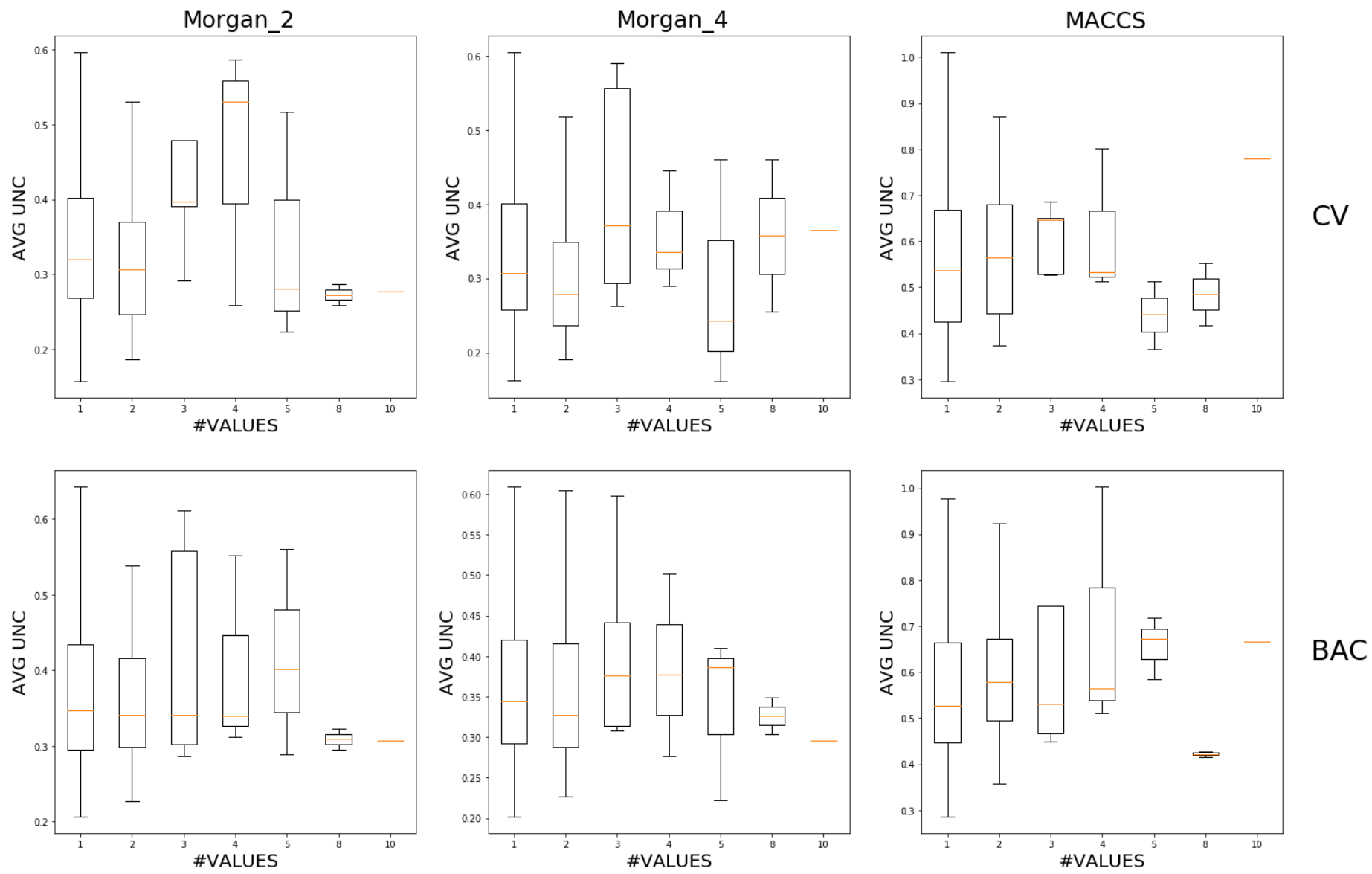

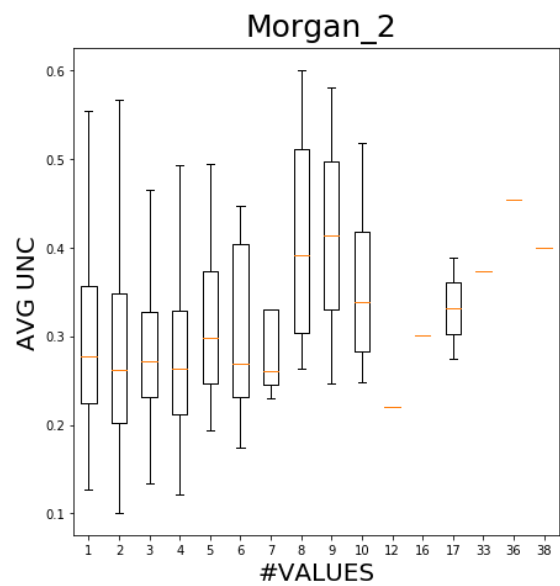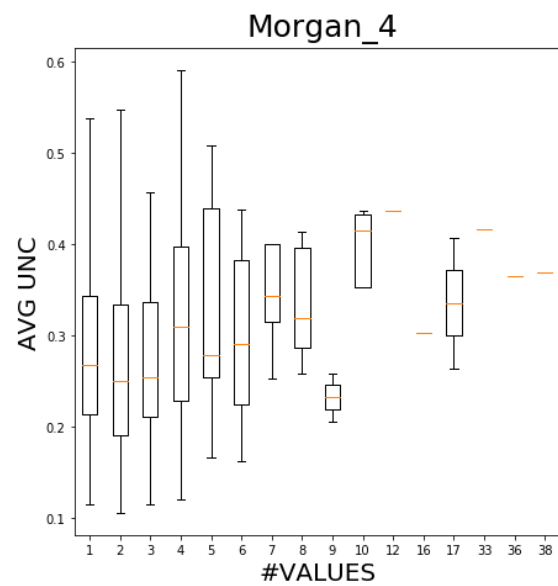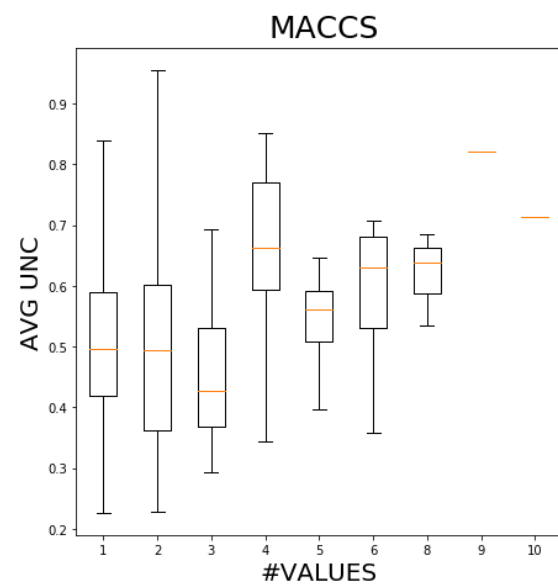

CV

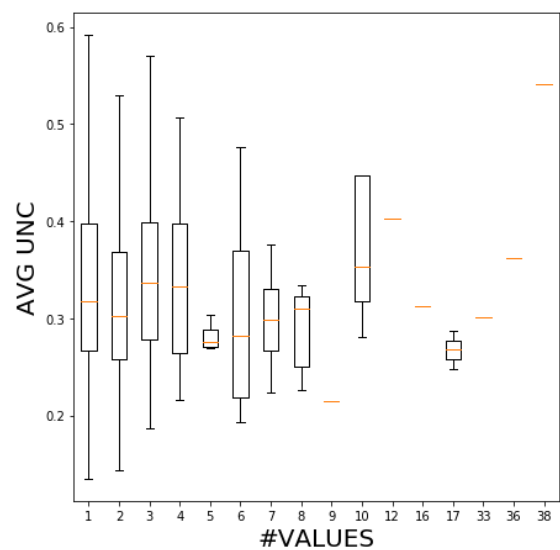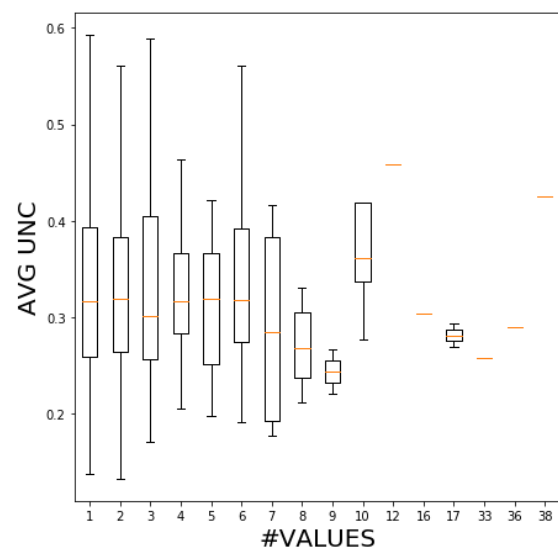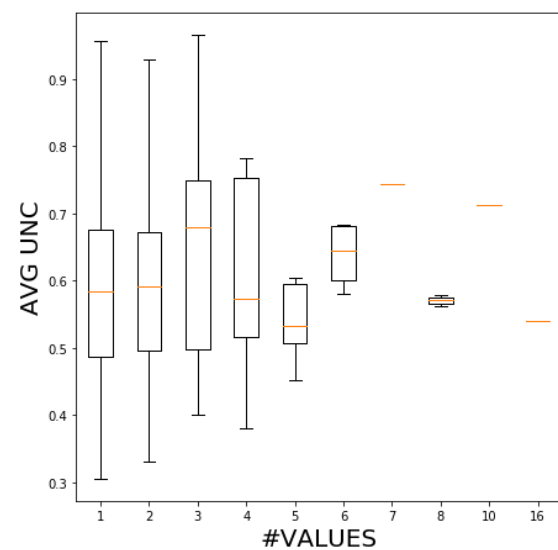

BAC

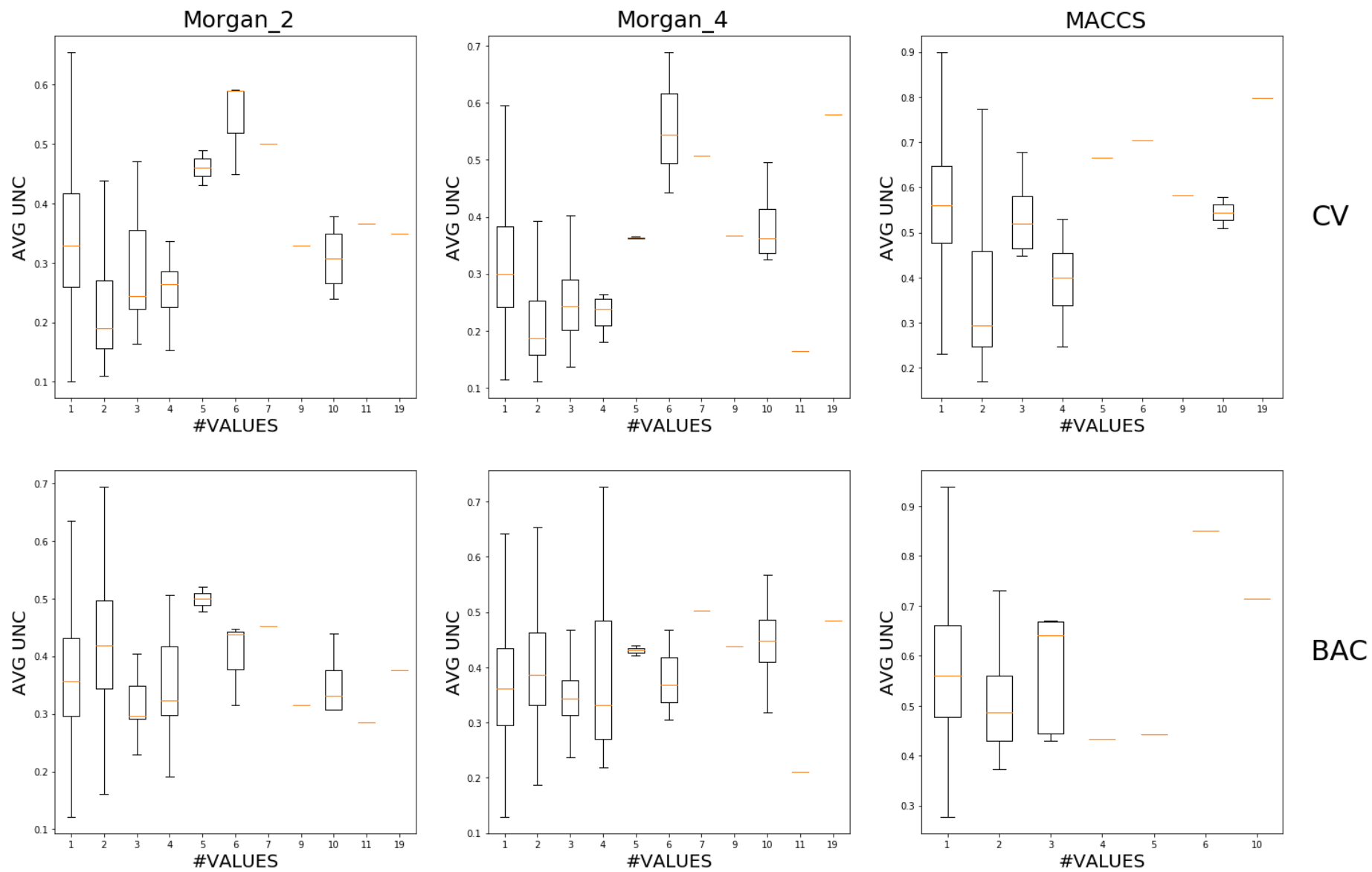

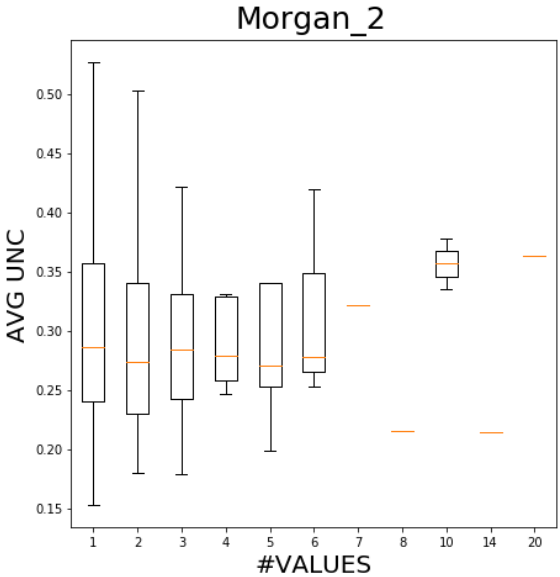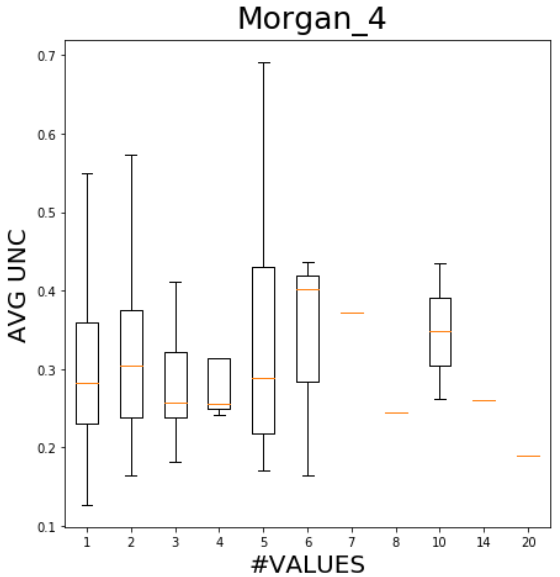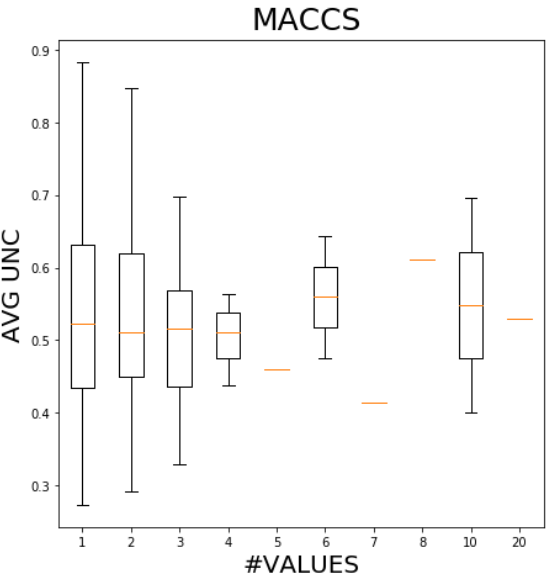

CV

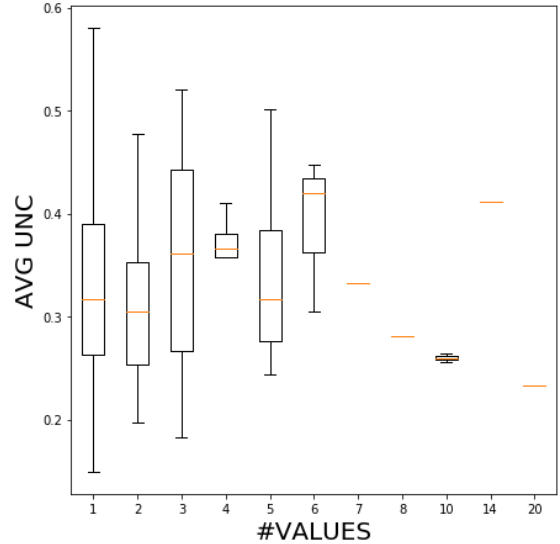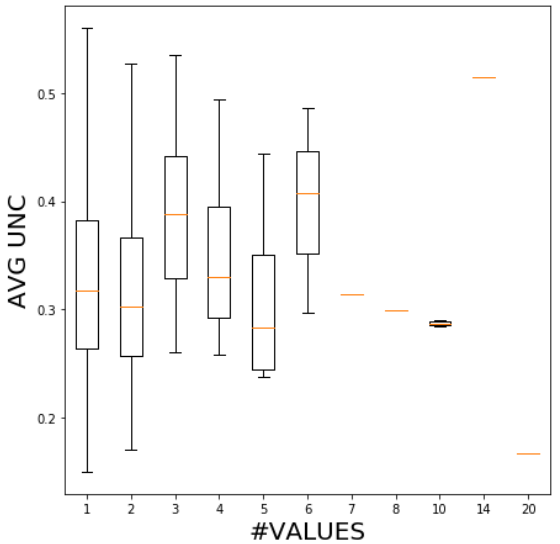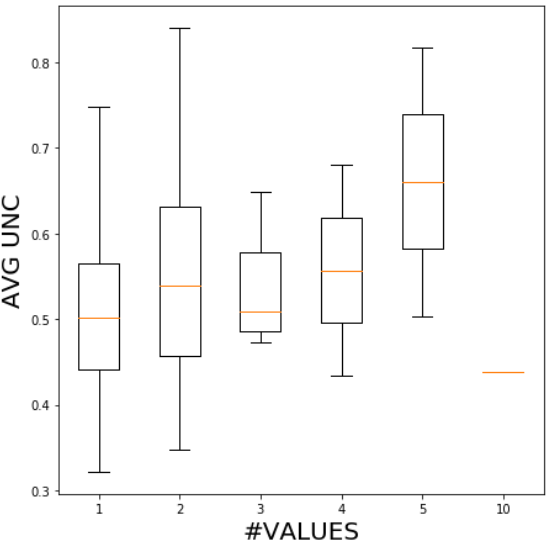

BAC

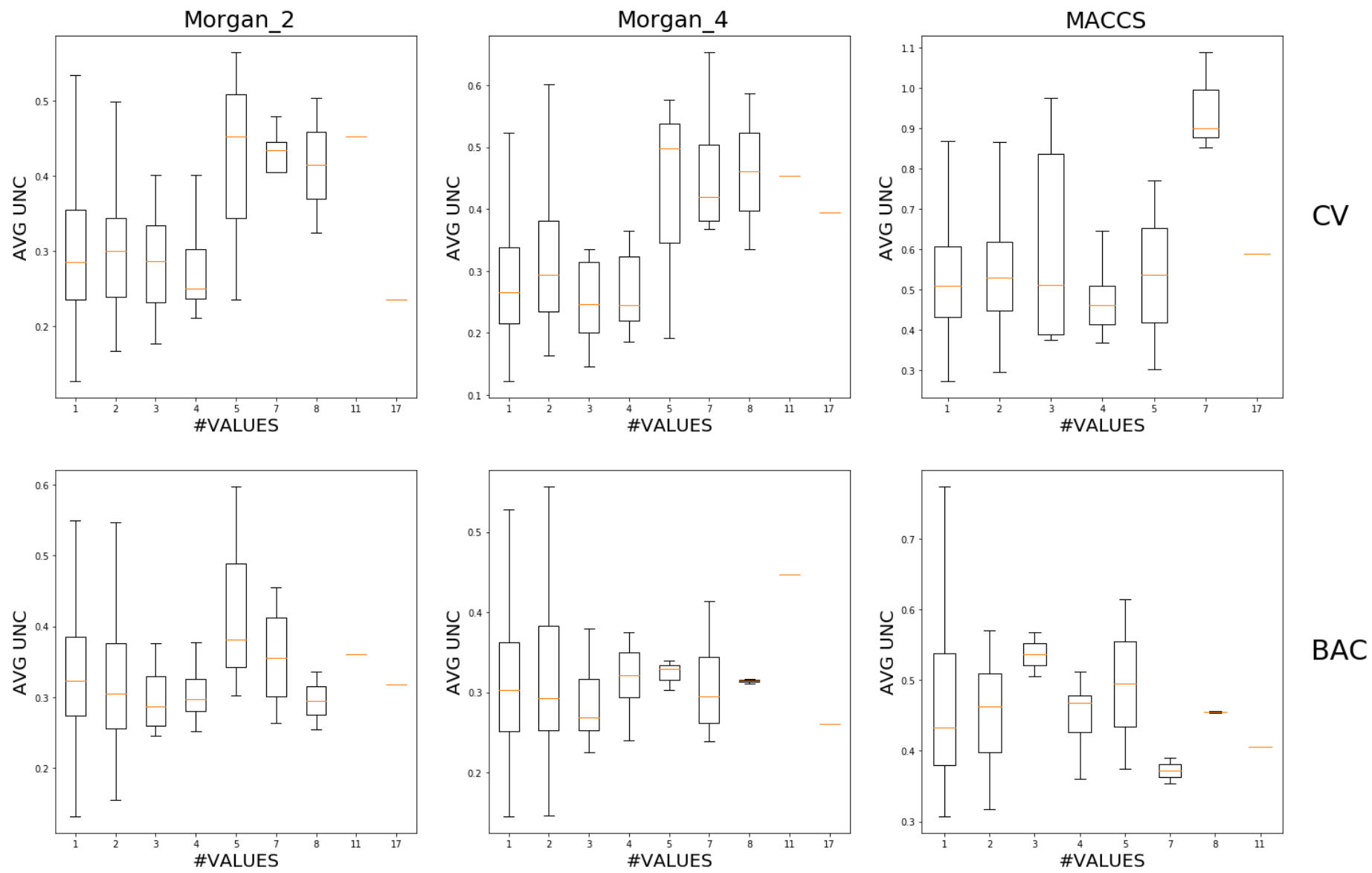

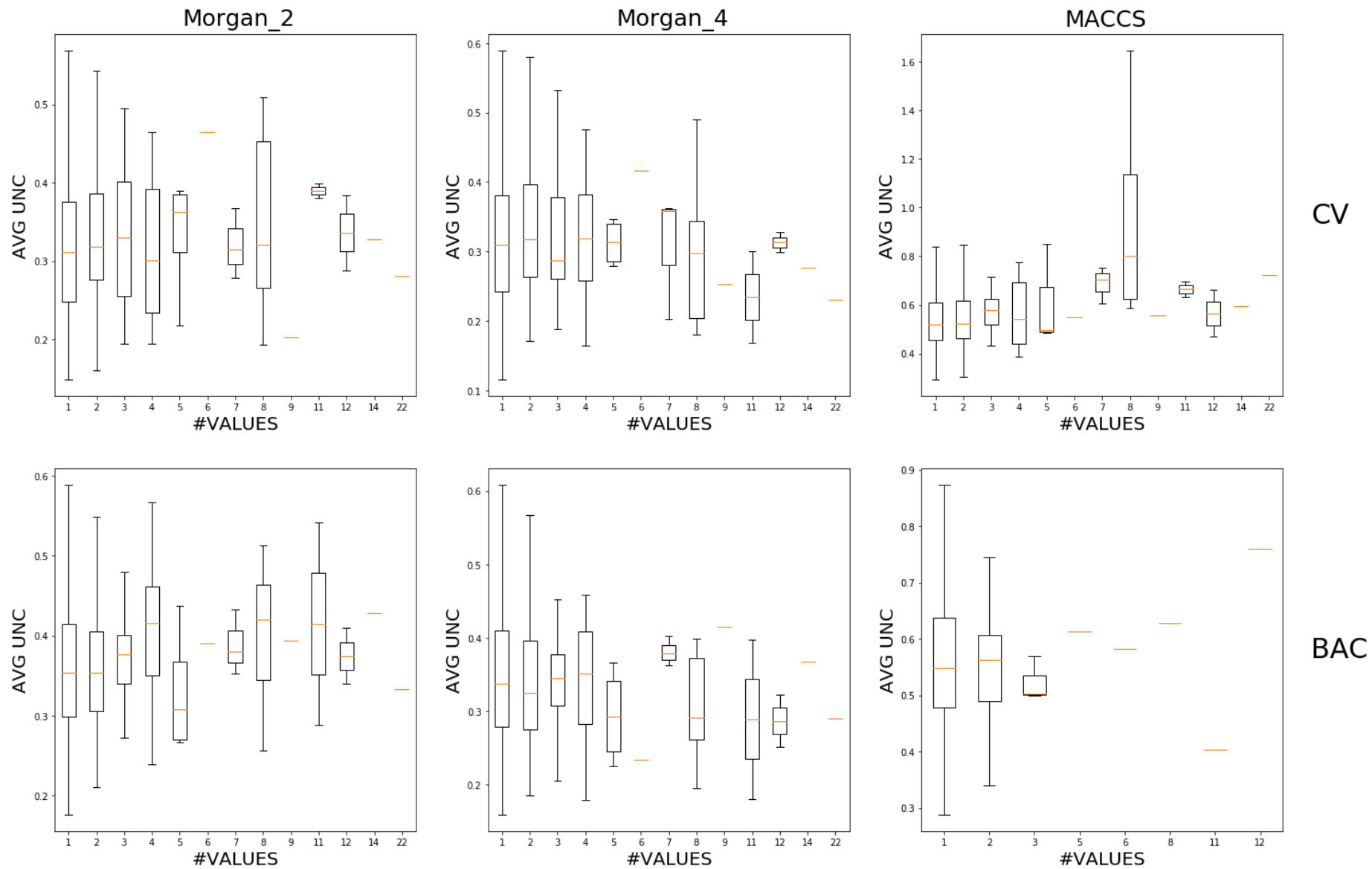

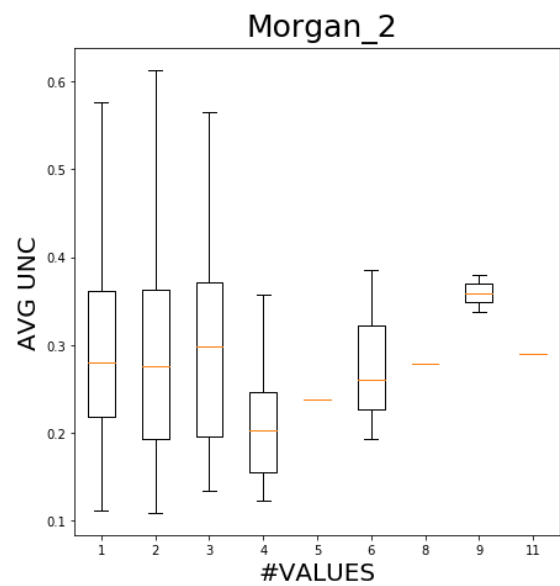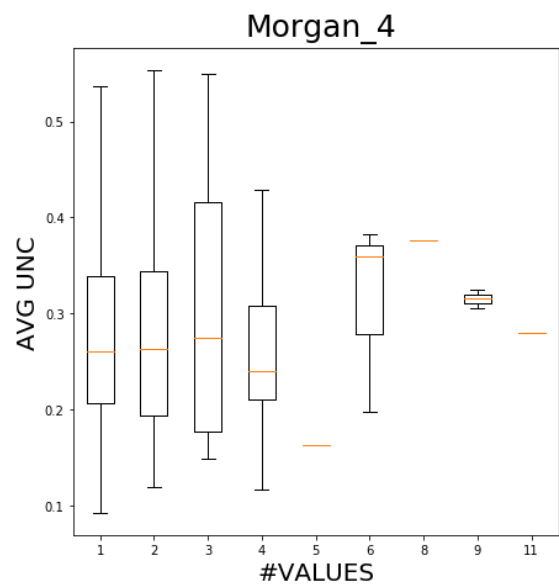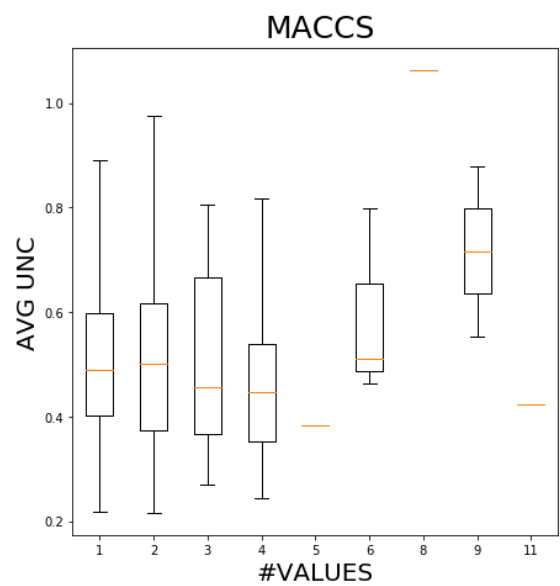

CV

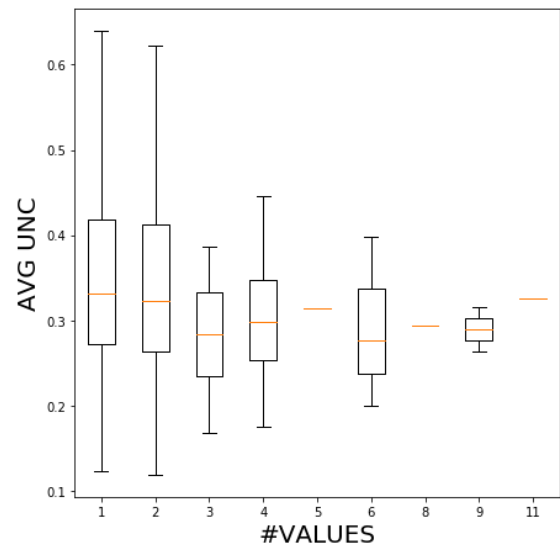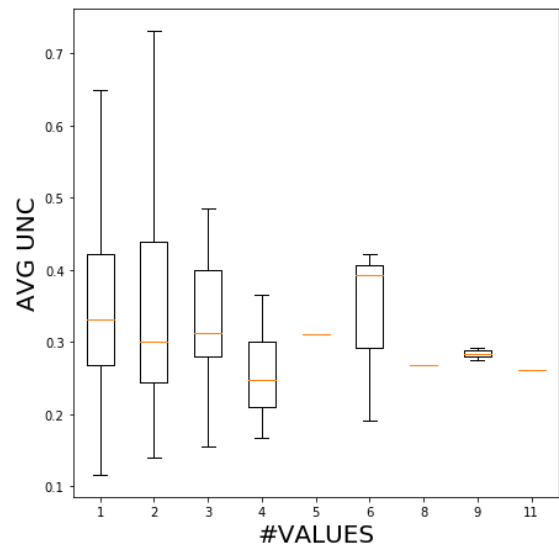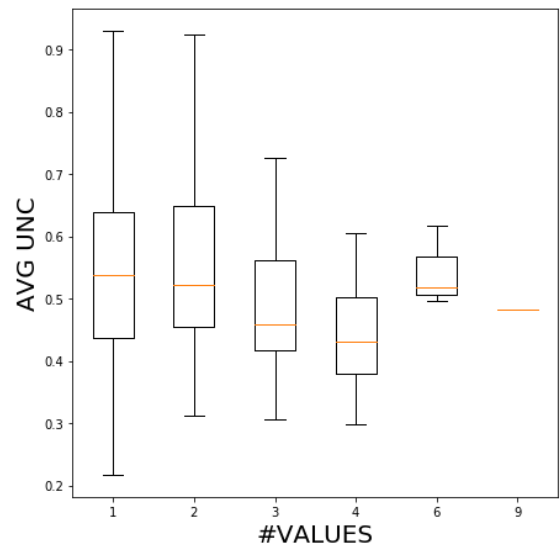

BAC

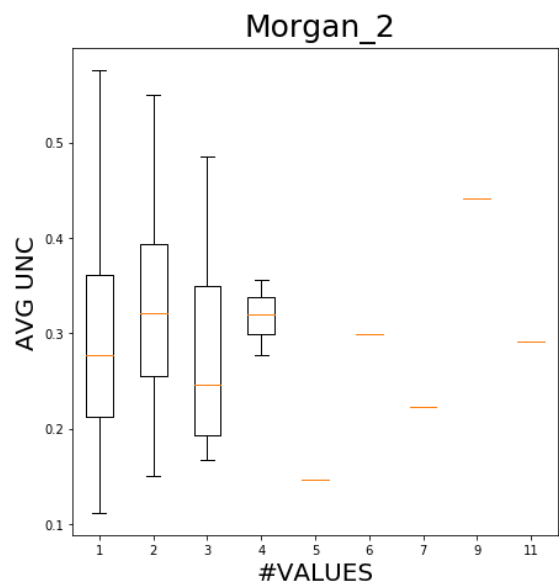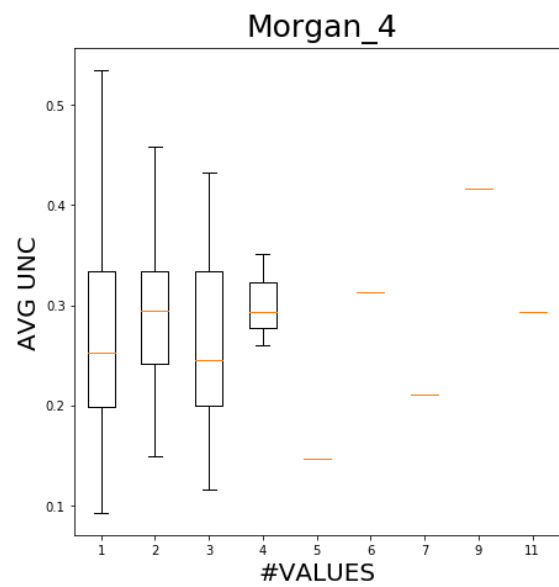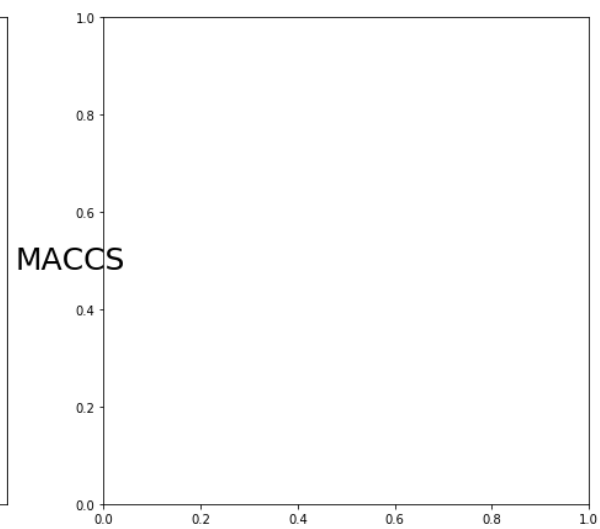

CV

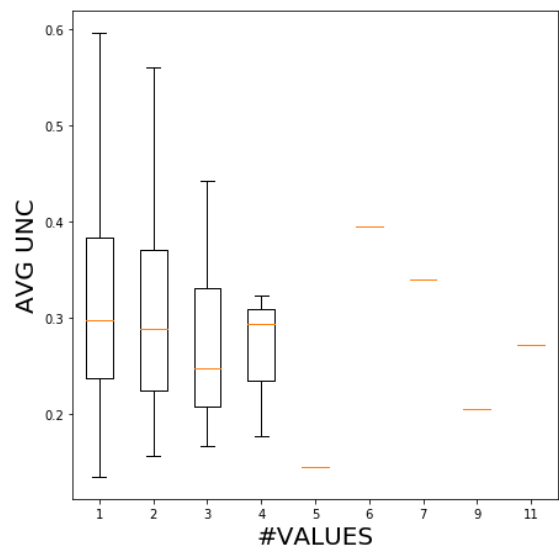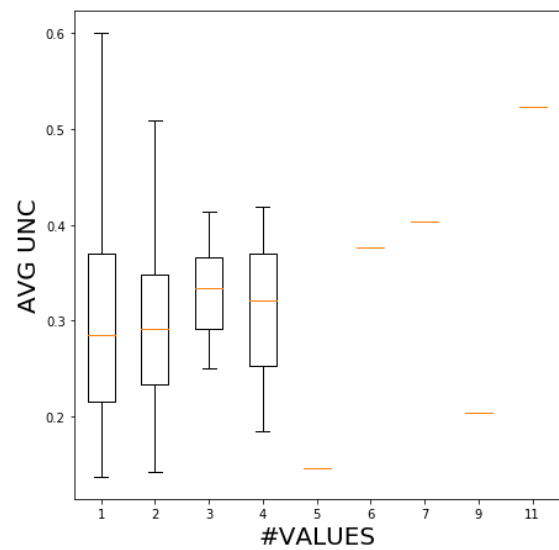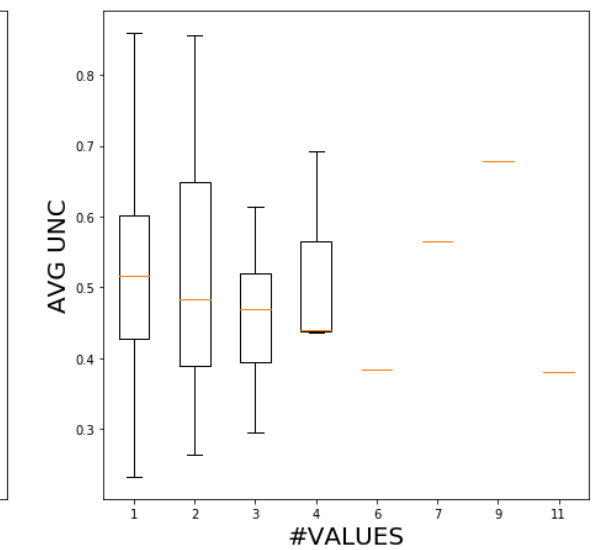

BAC

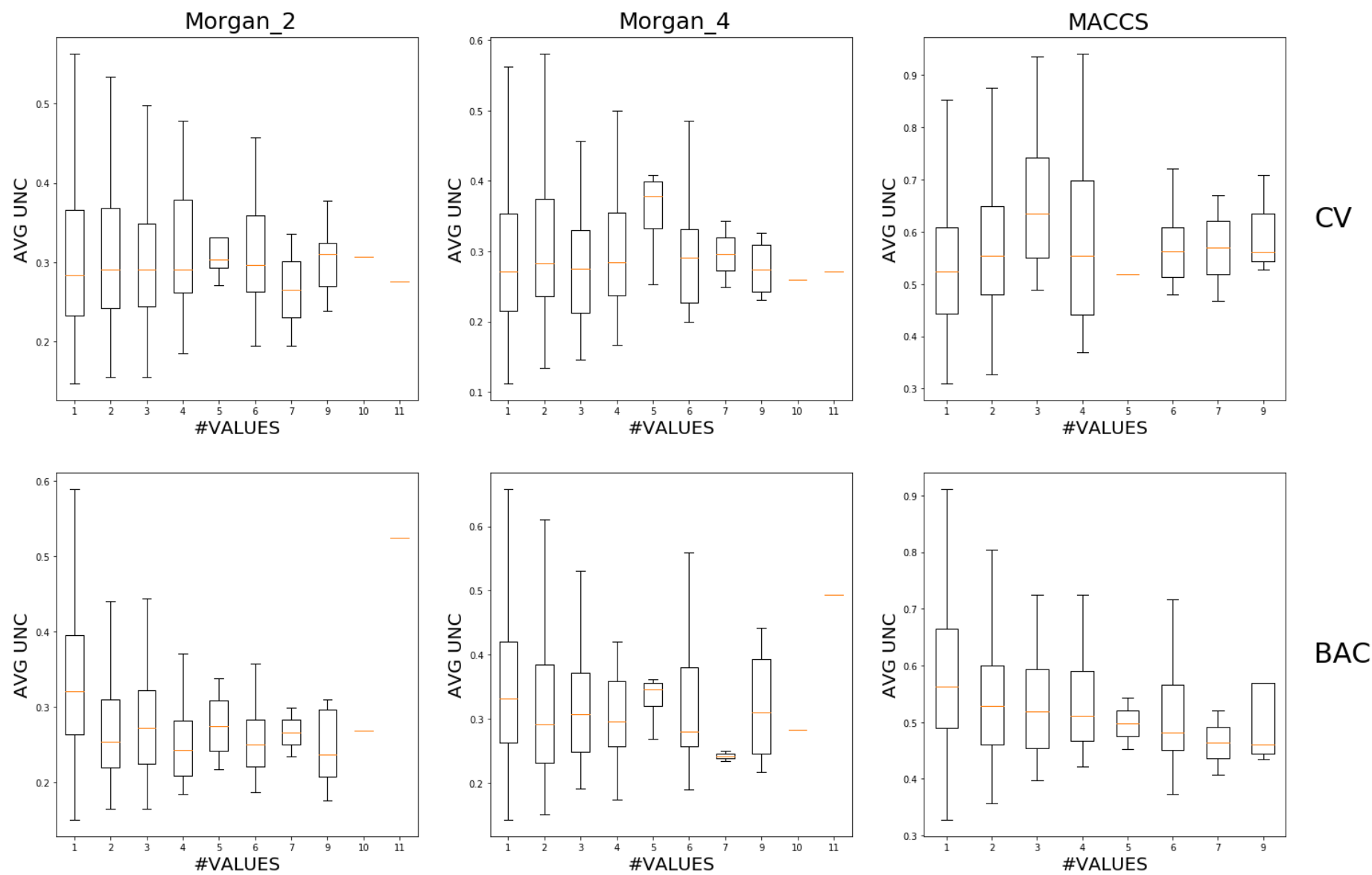

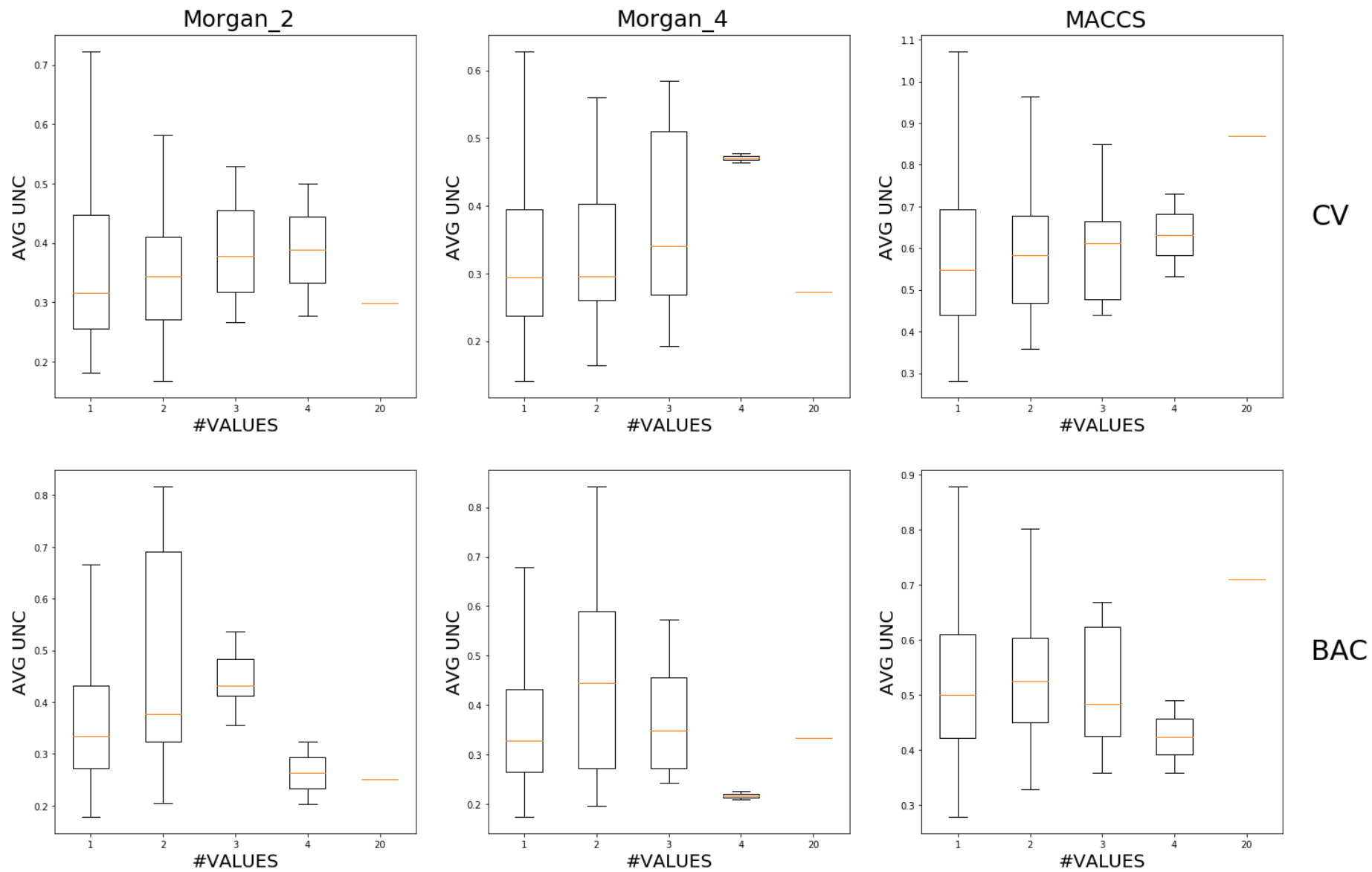

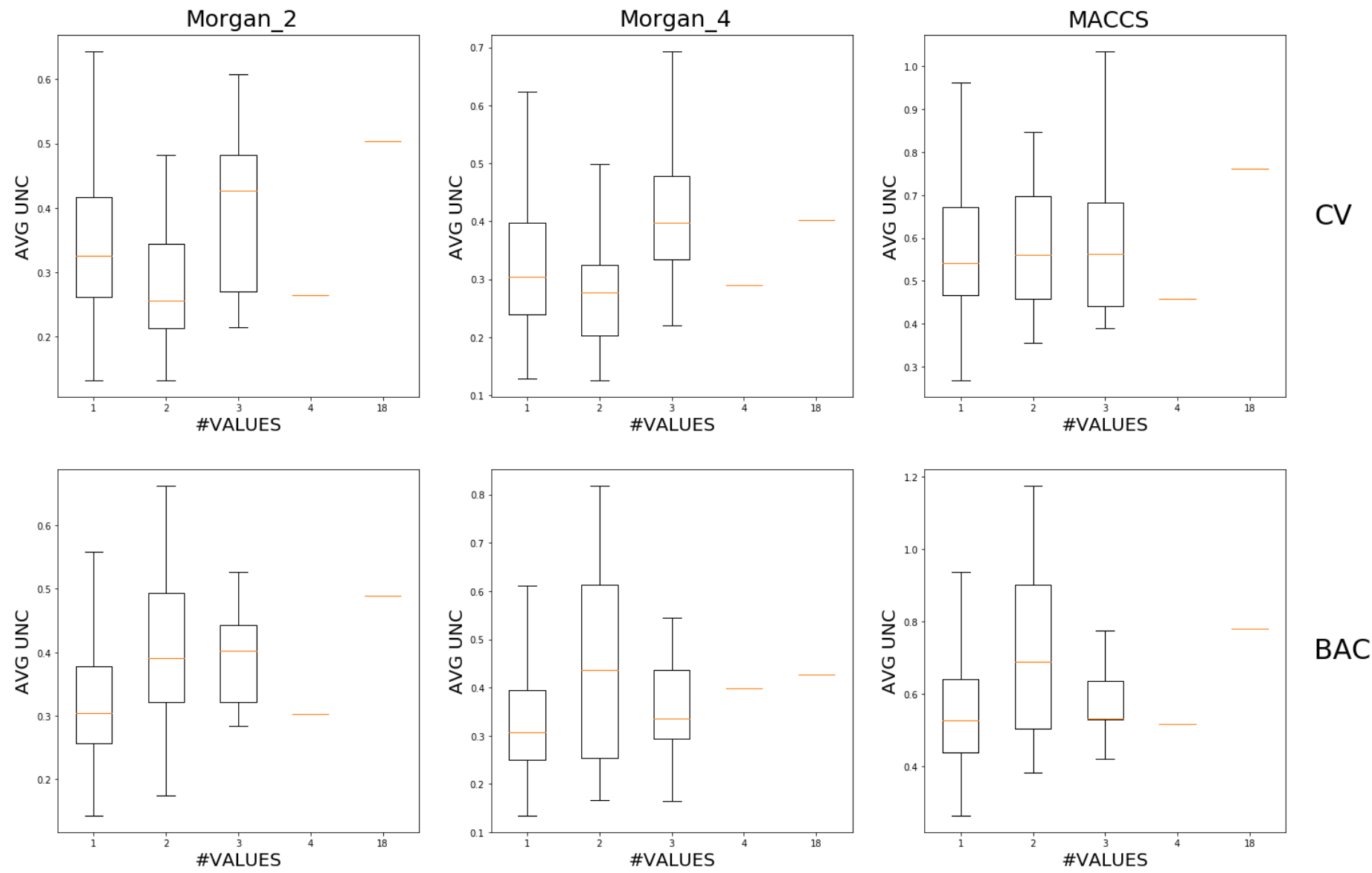

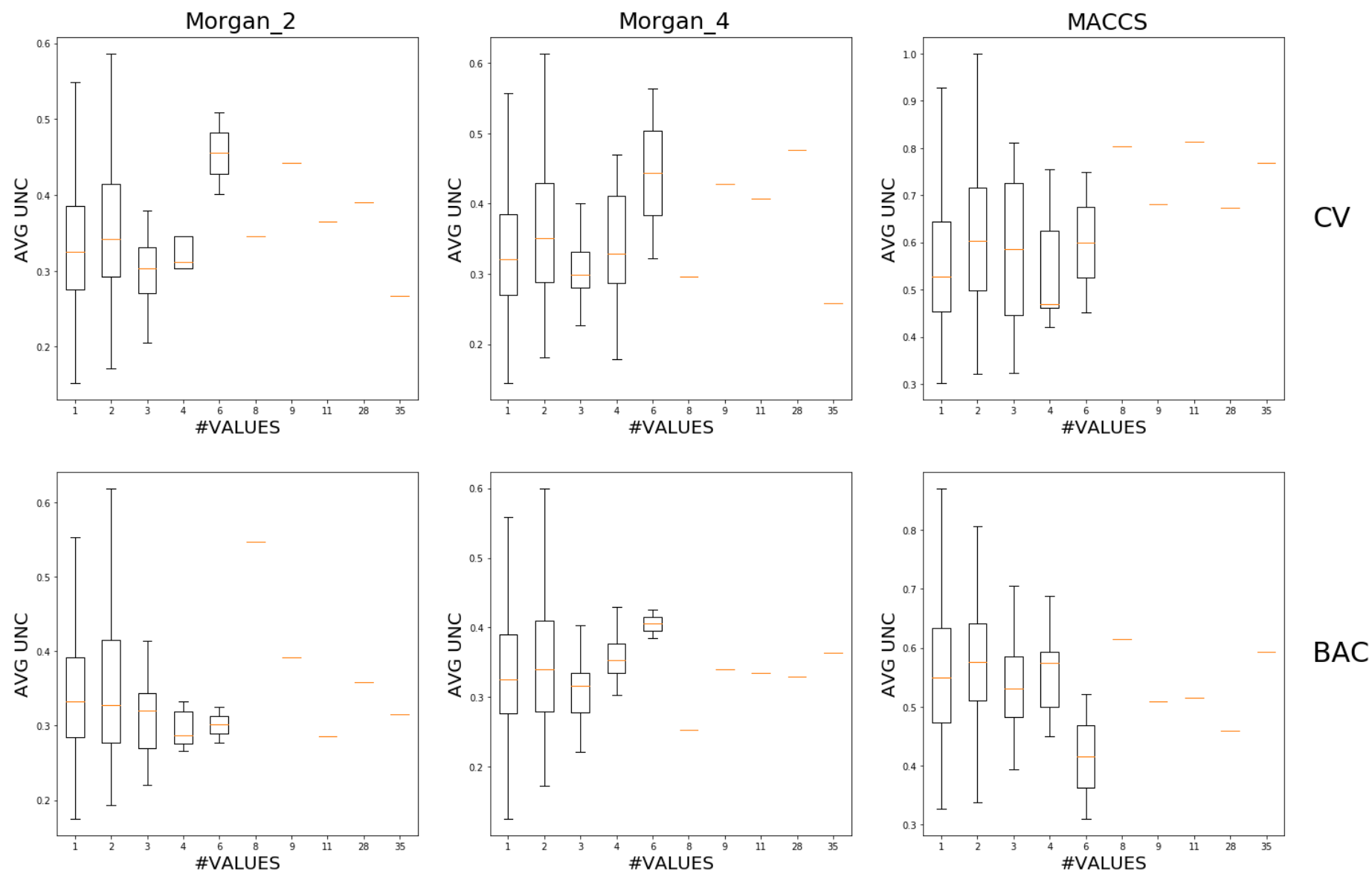

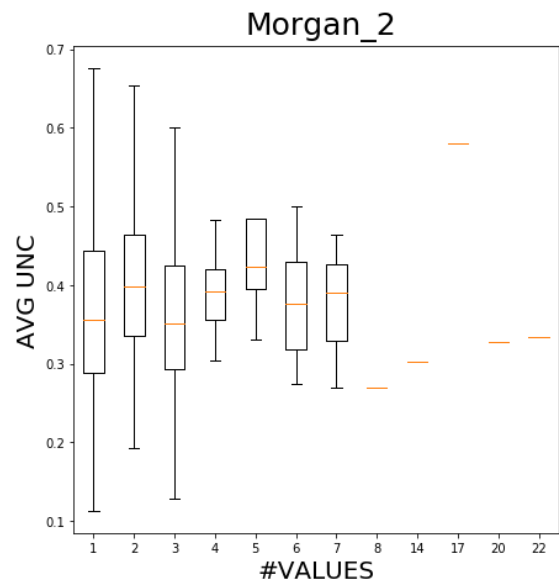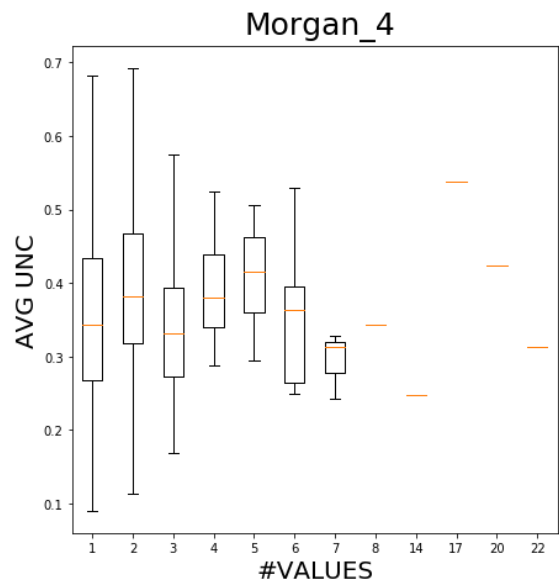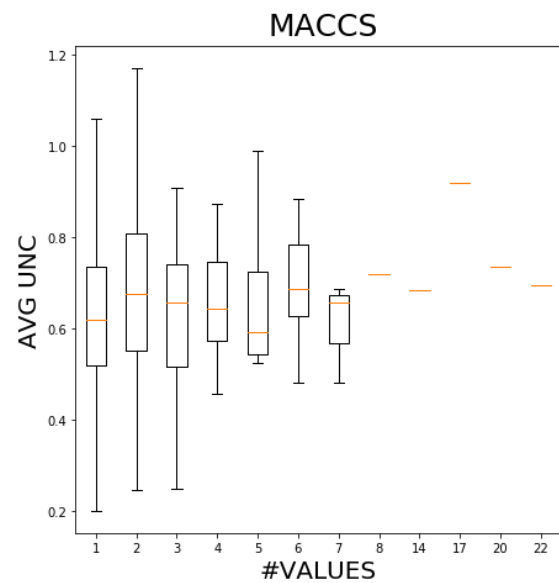

CV

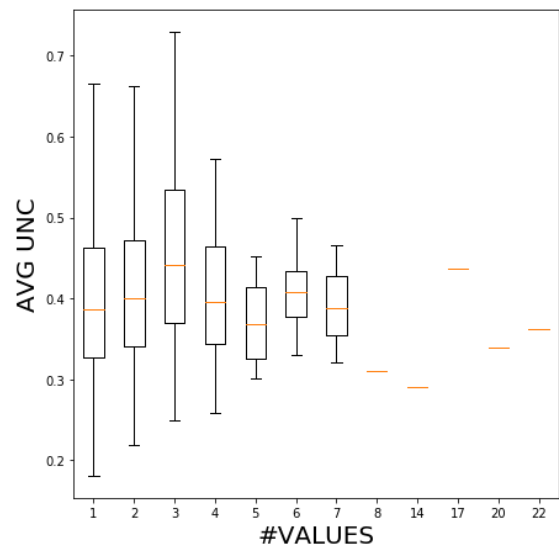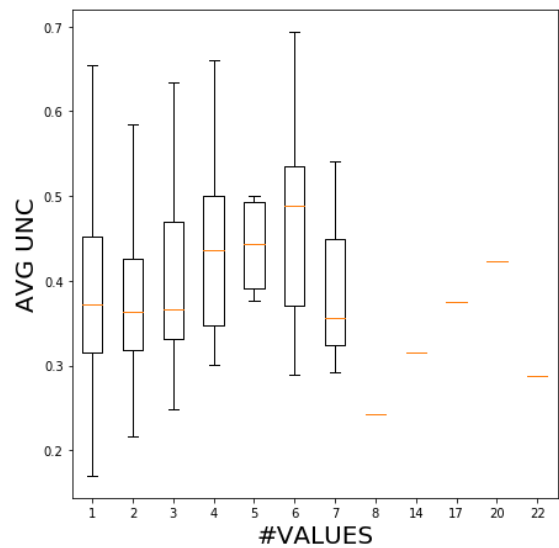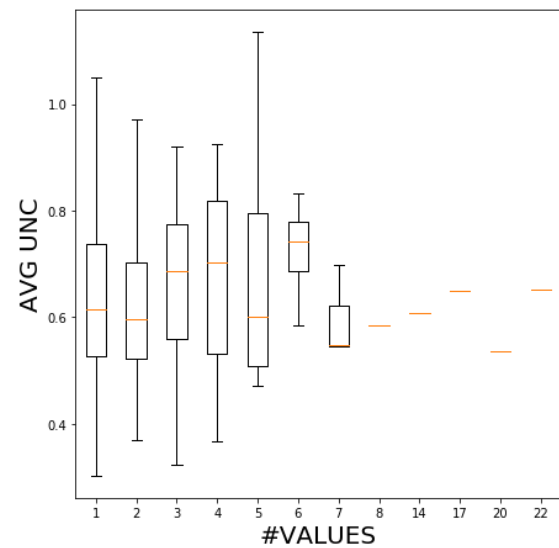

BAC

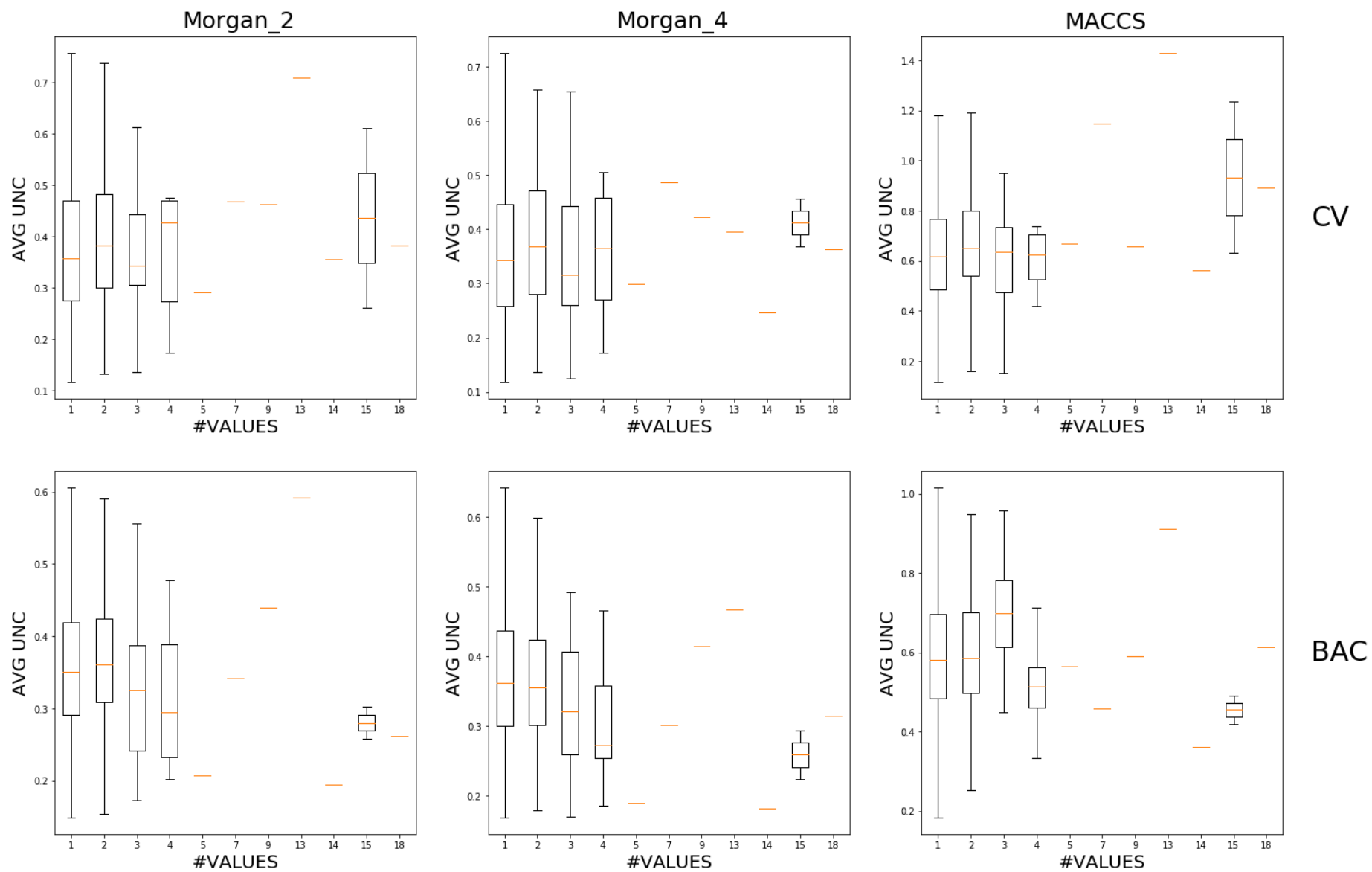

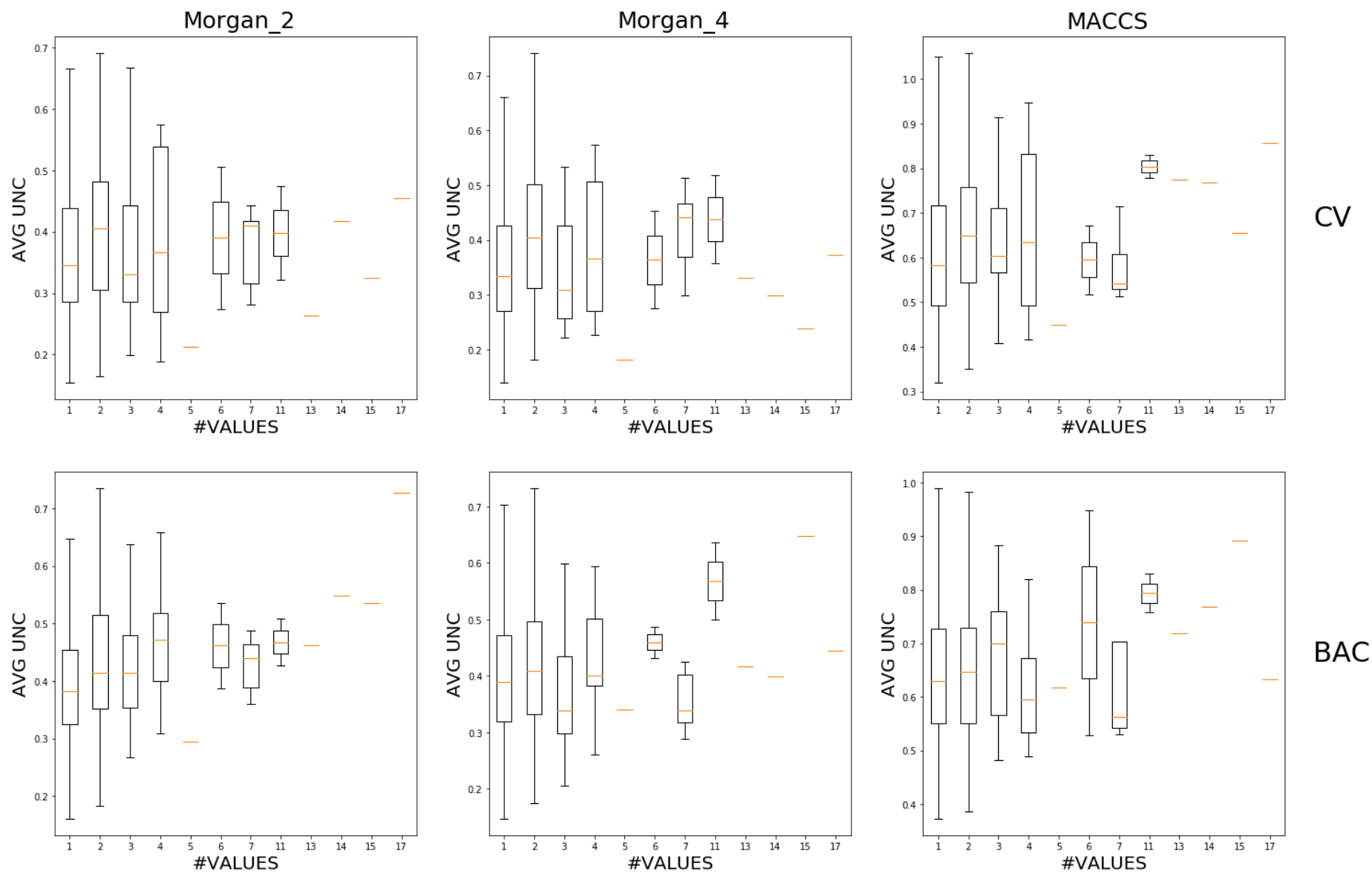

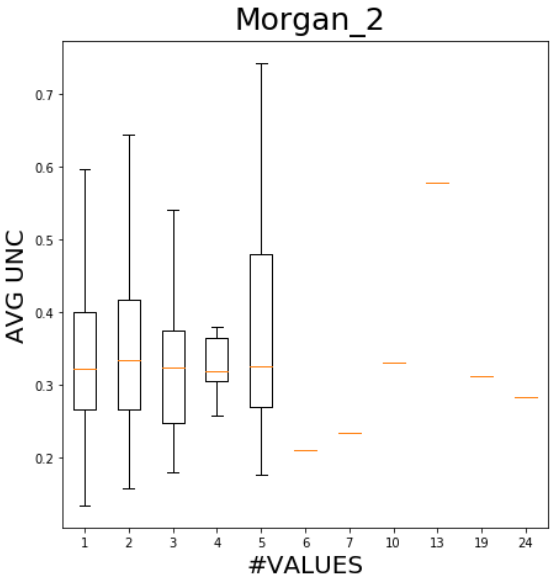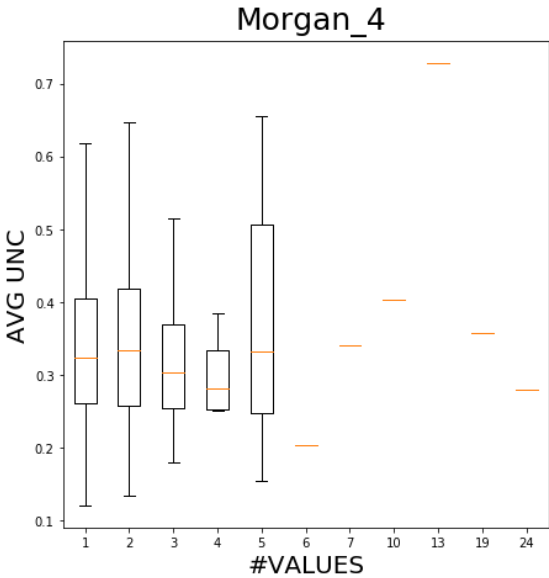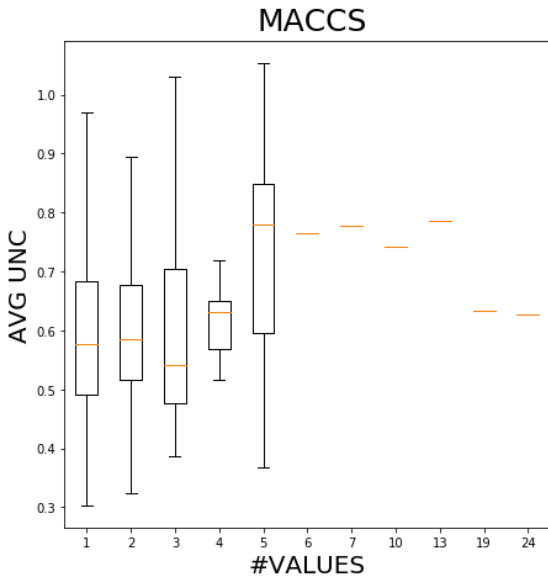

CV

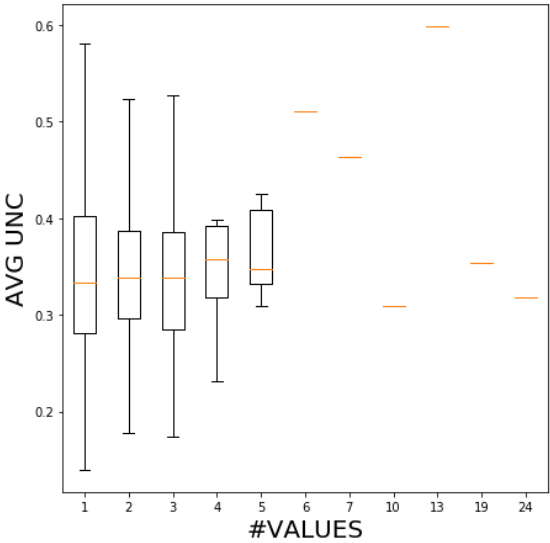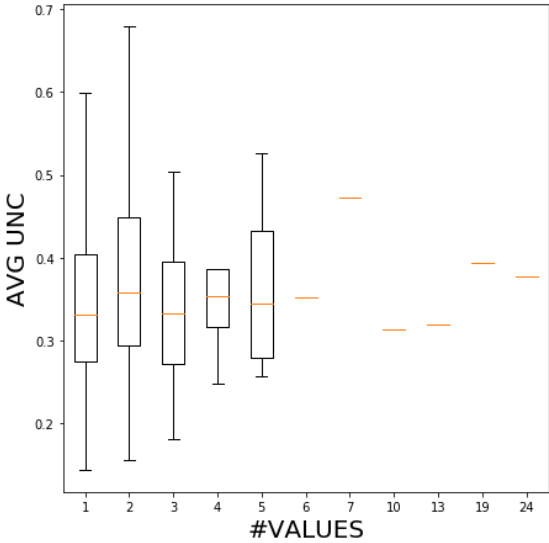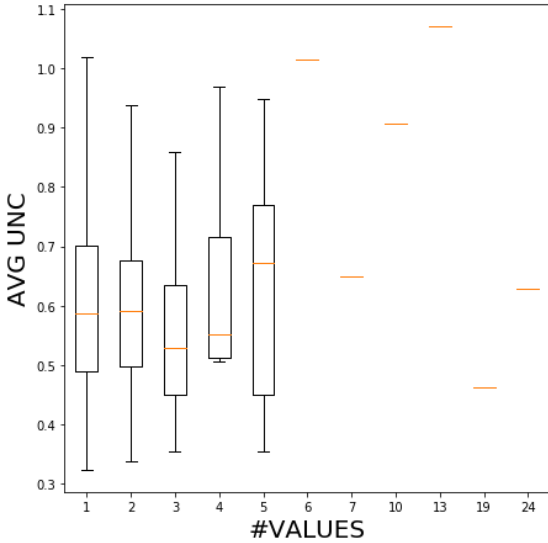

BAC

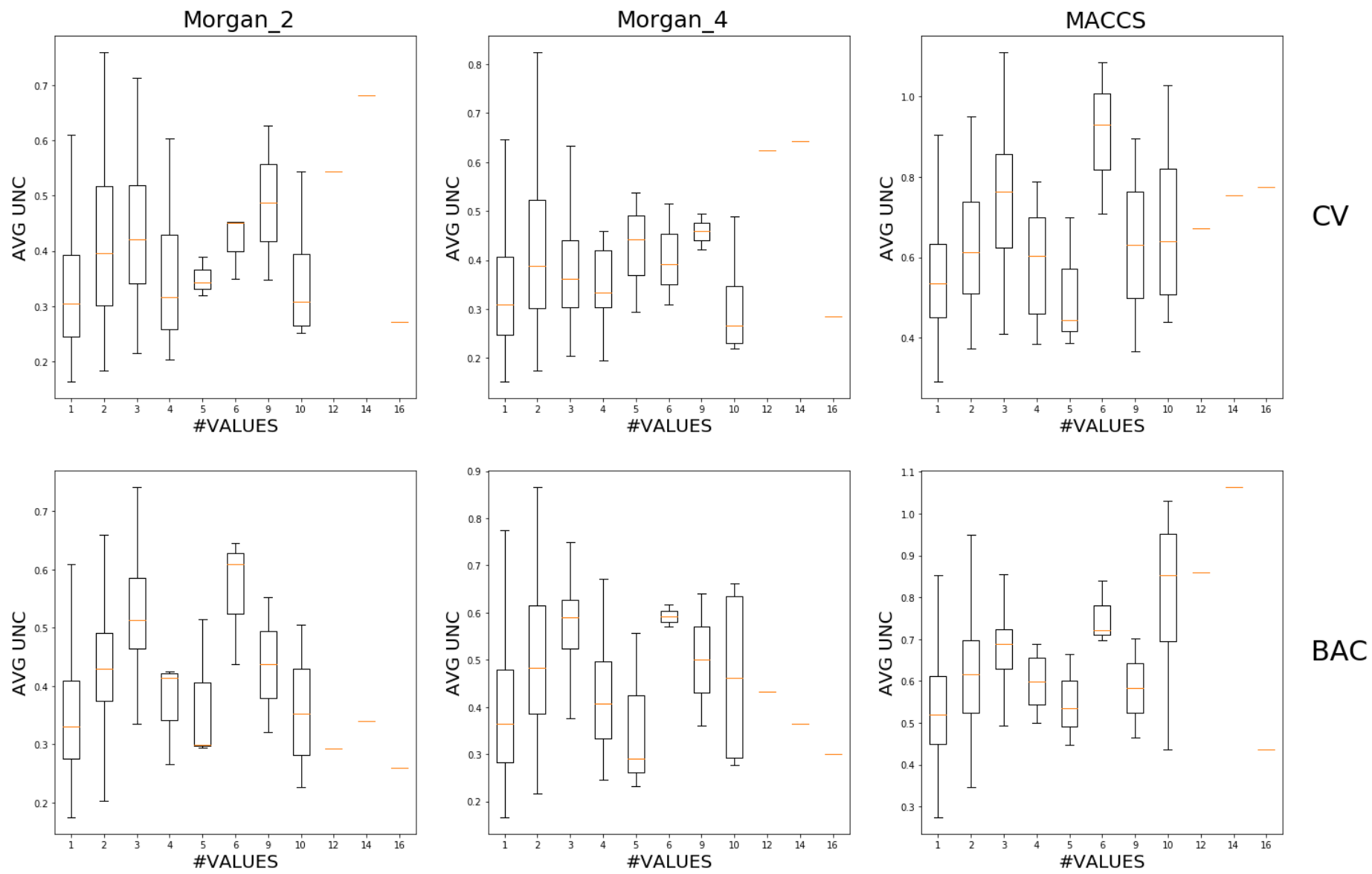

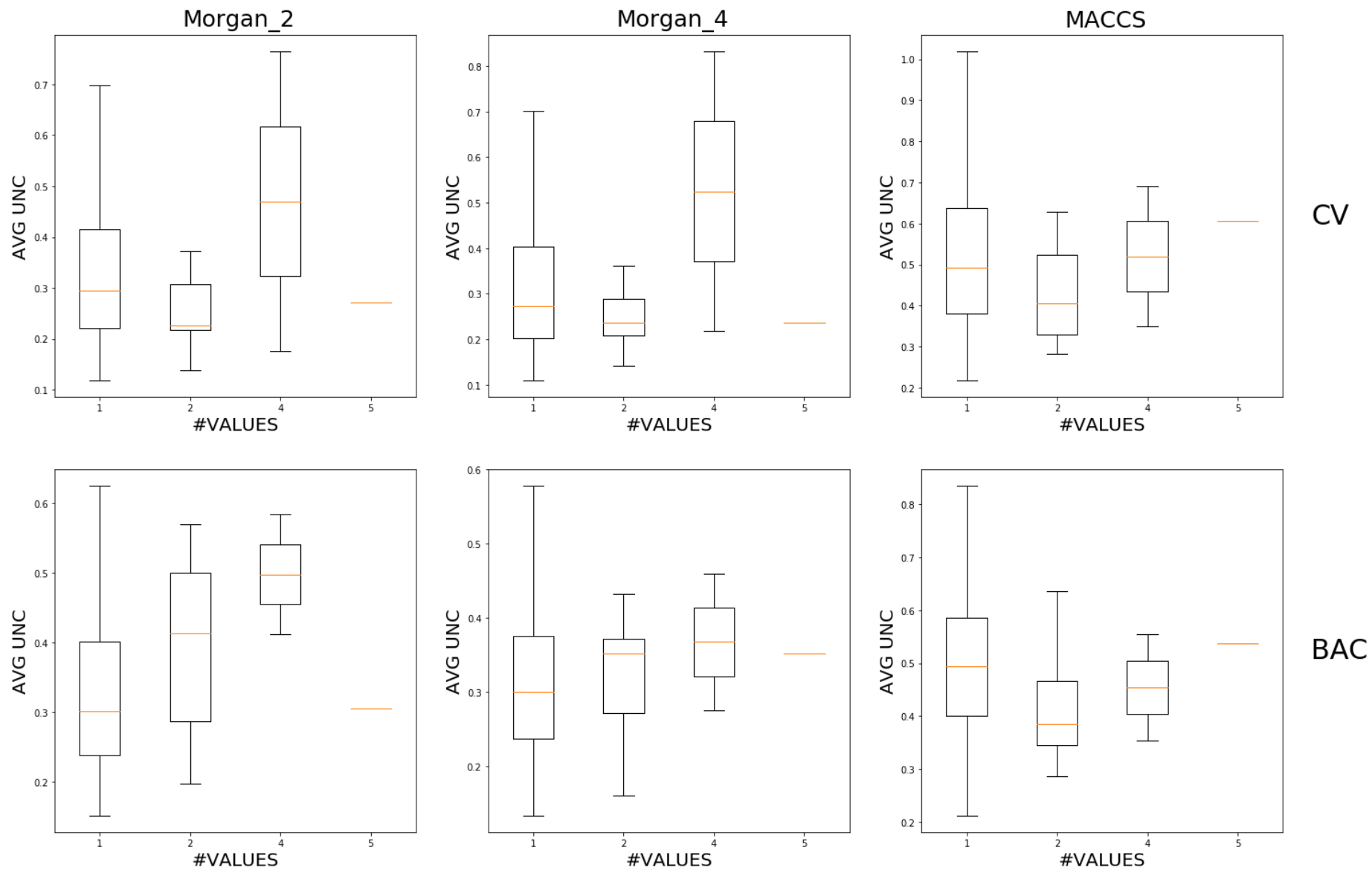

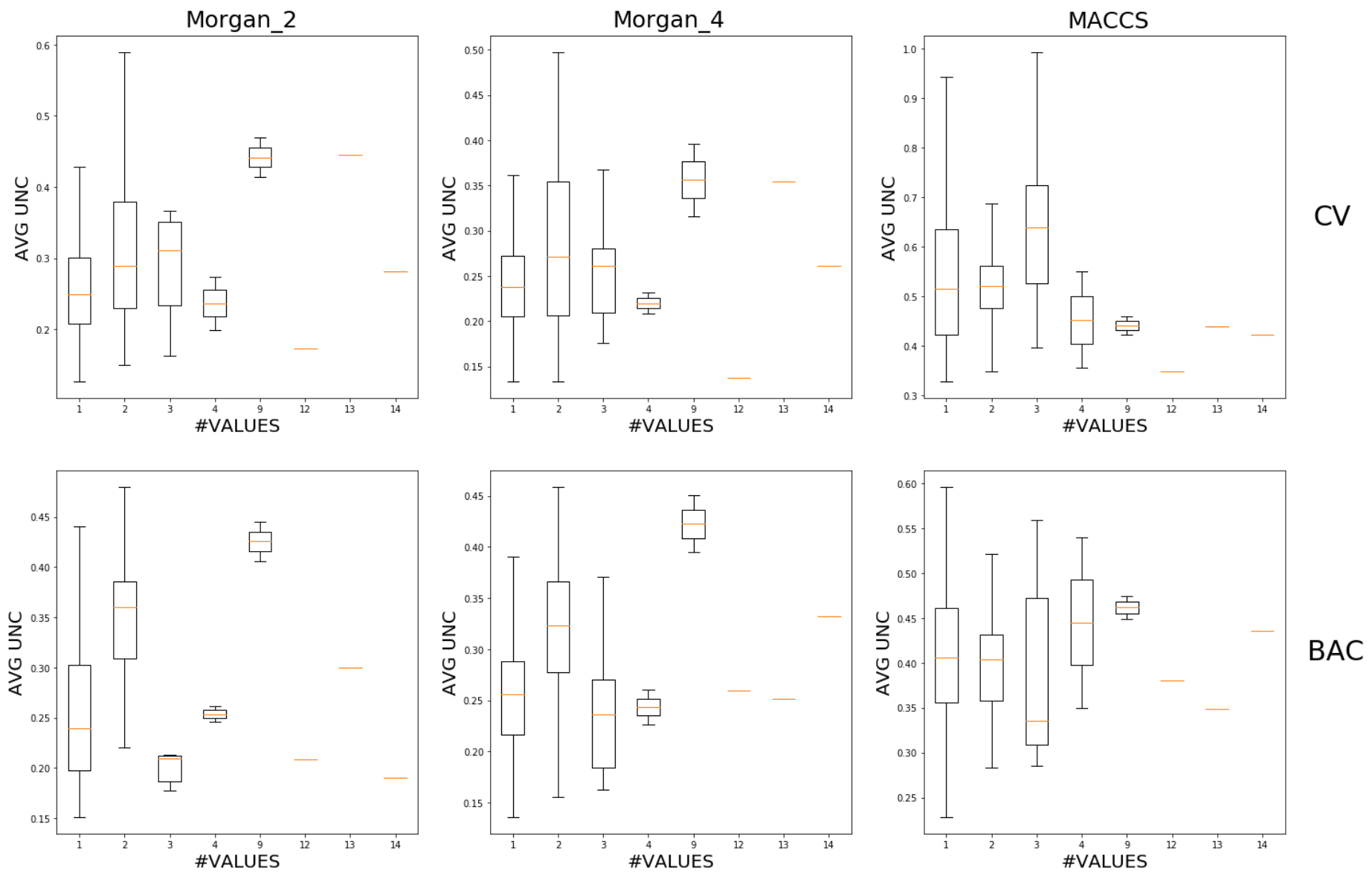

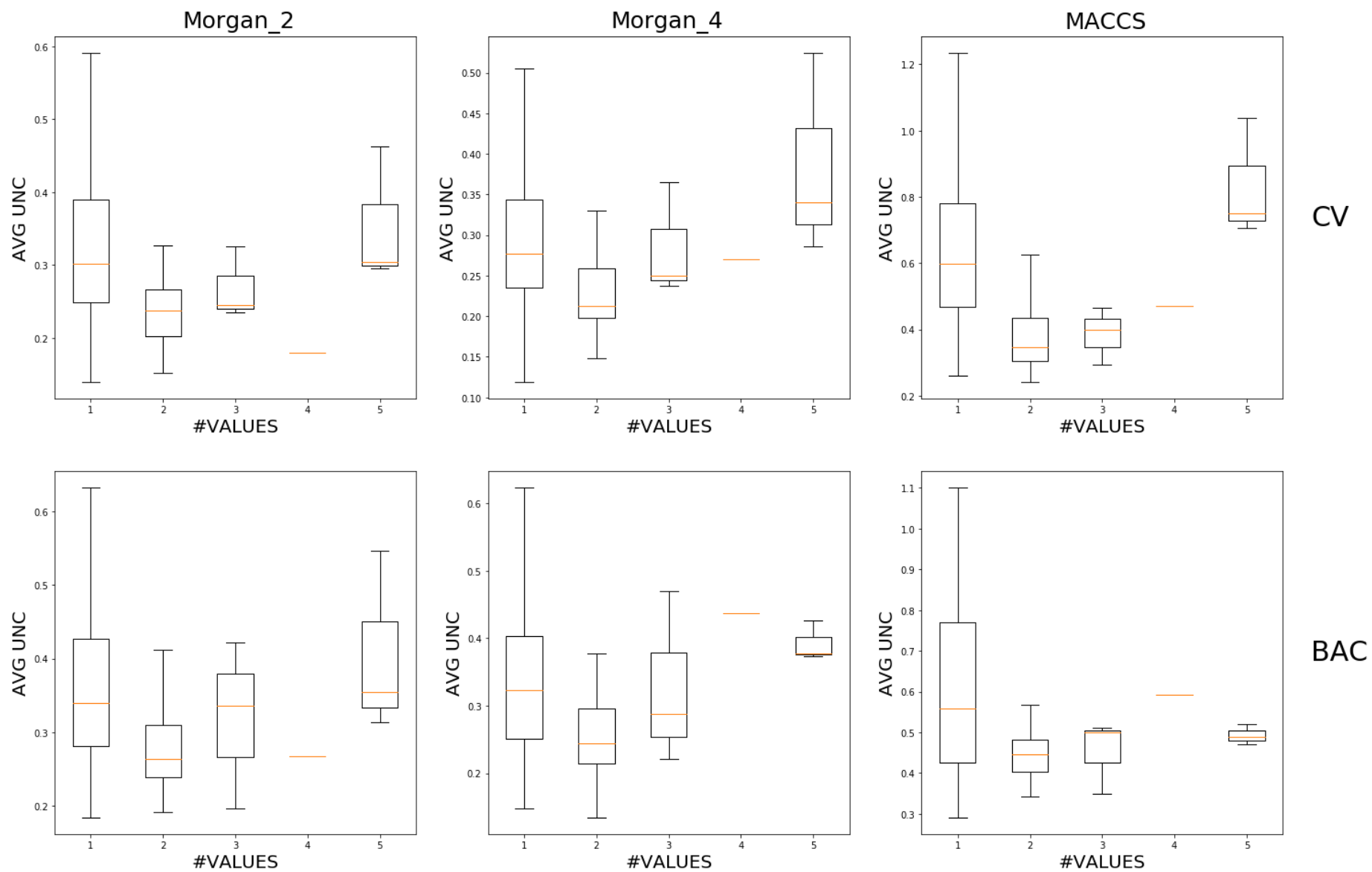

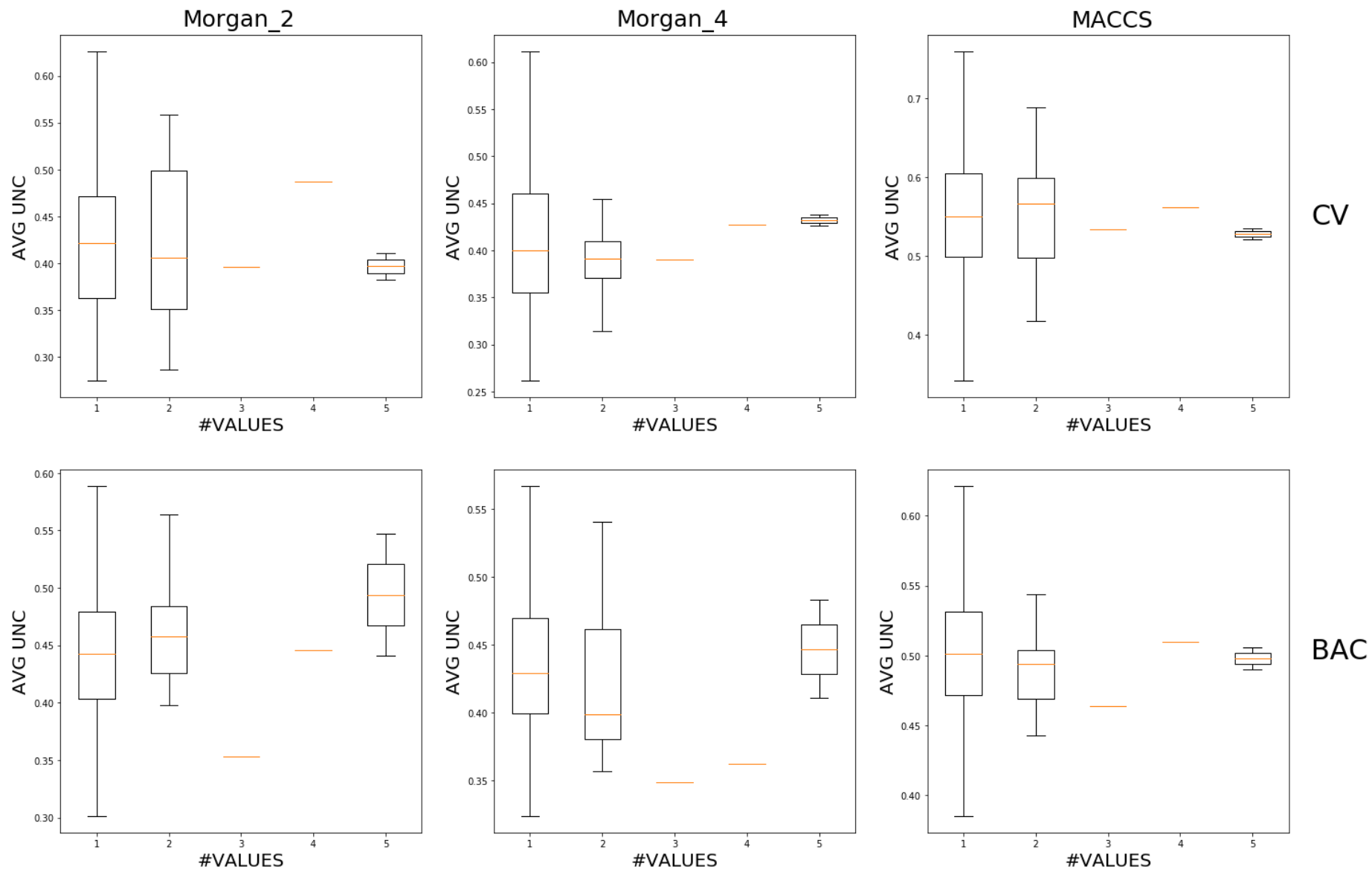

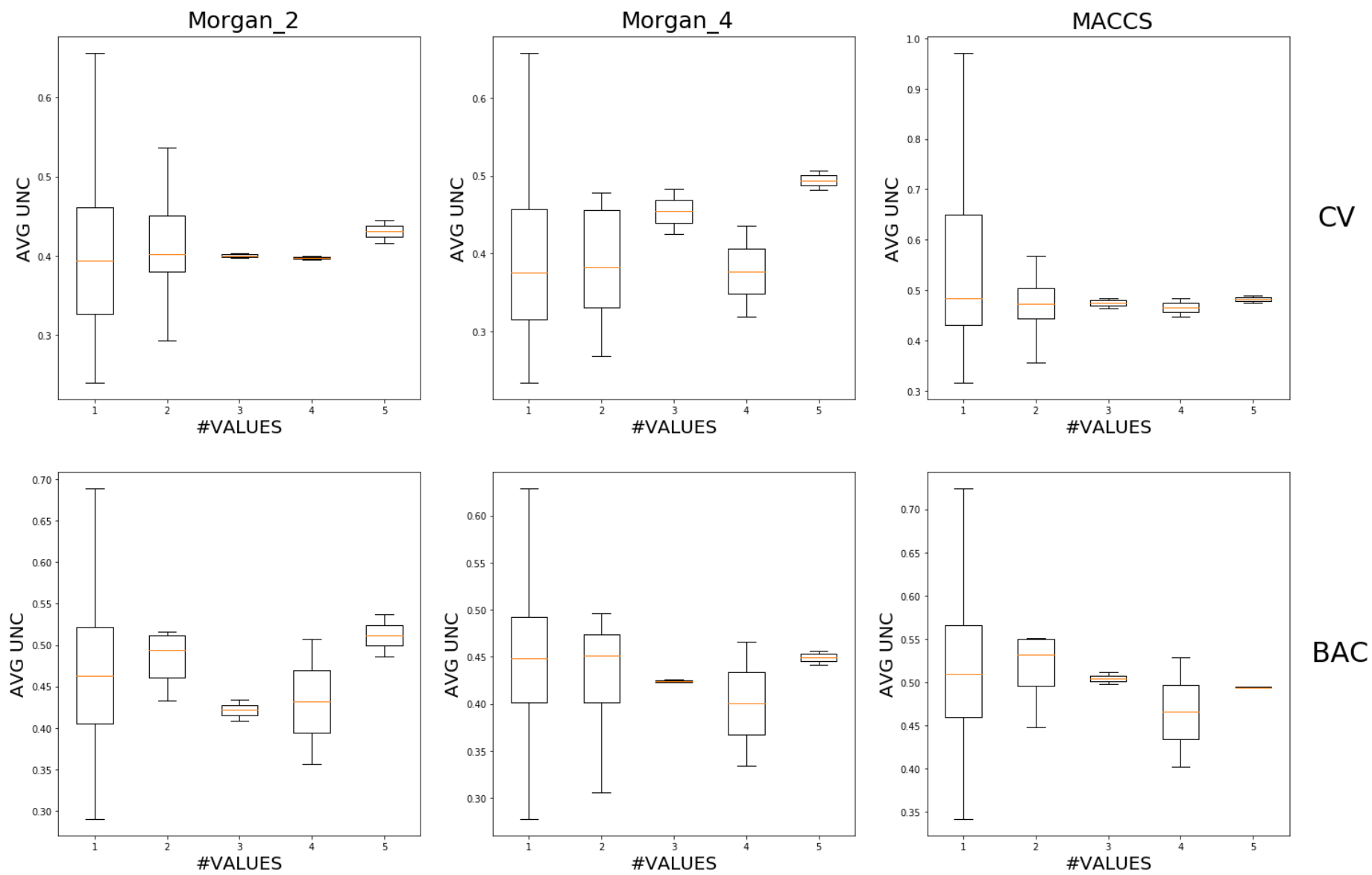

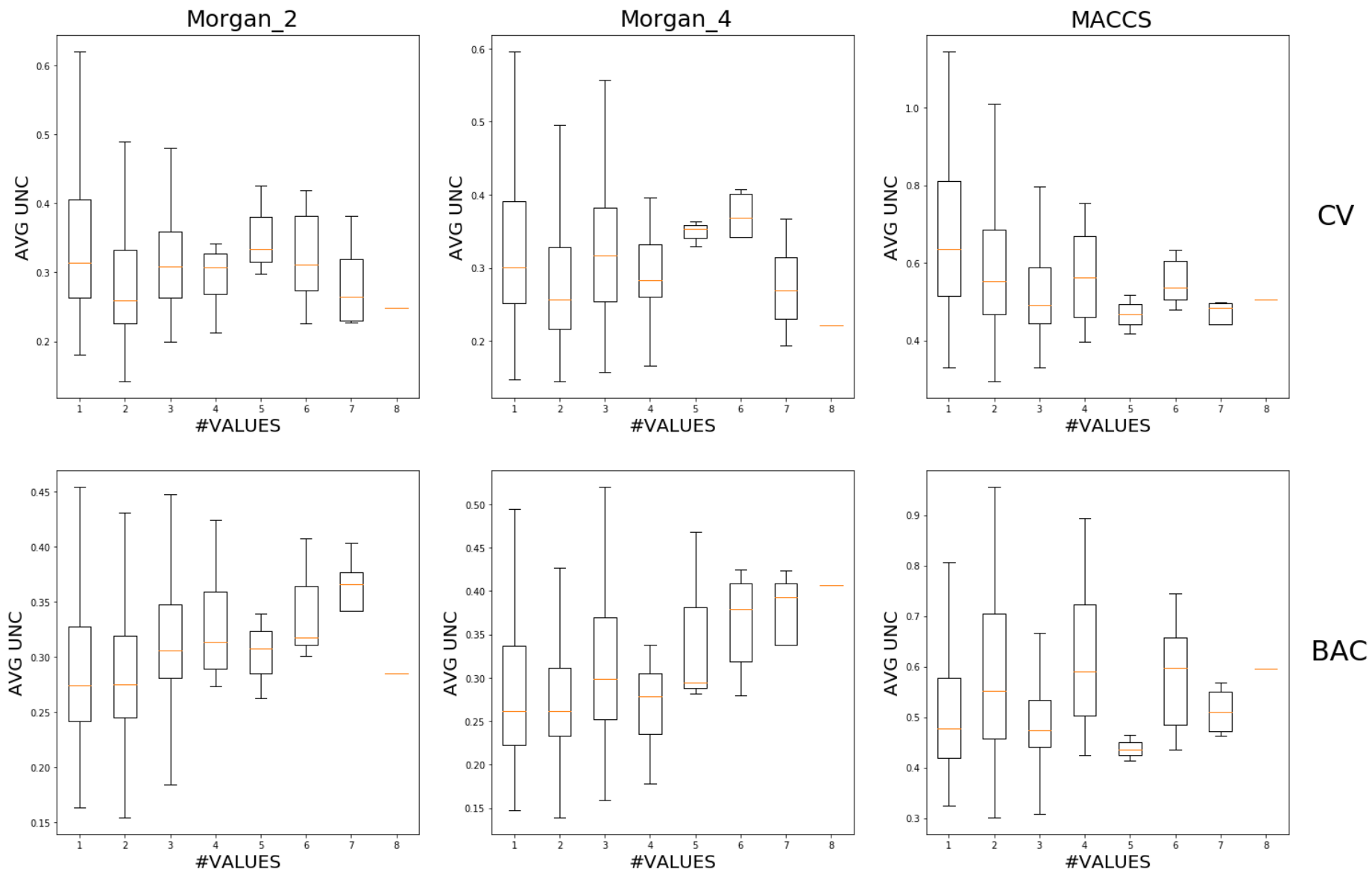

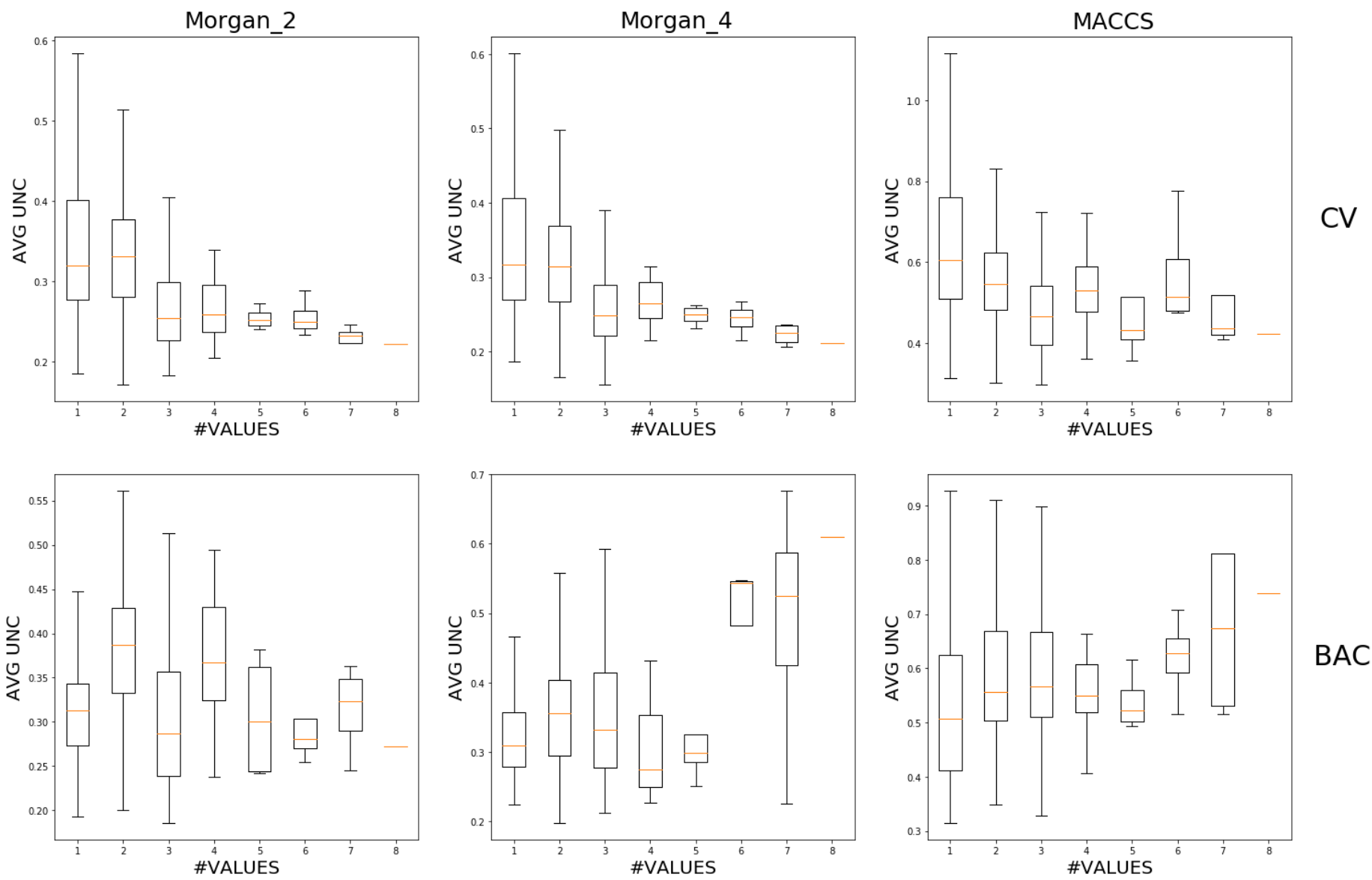

Supplement: Supplementary file 1 [file molecules-25-01452-s001.zip › Supp_Info_for_submission/FileS2.pdf]

CHEMBL214

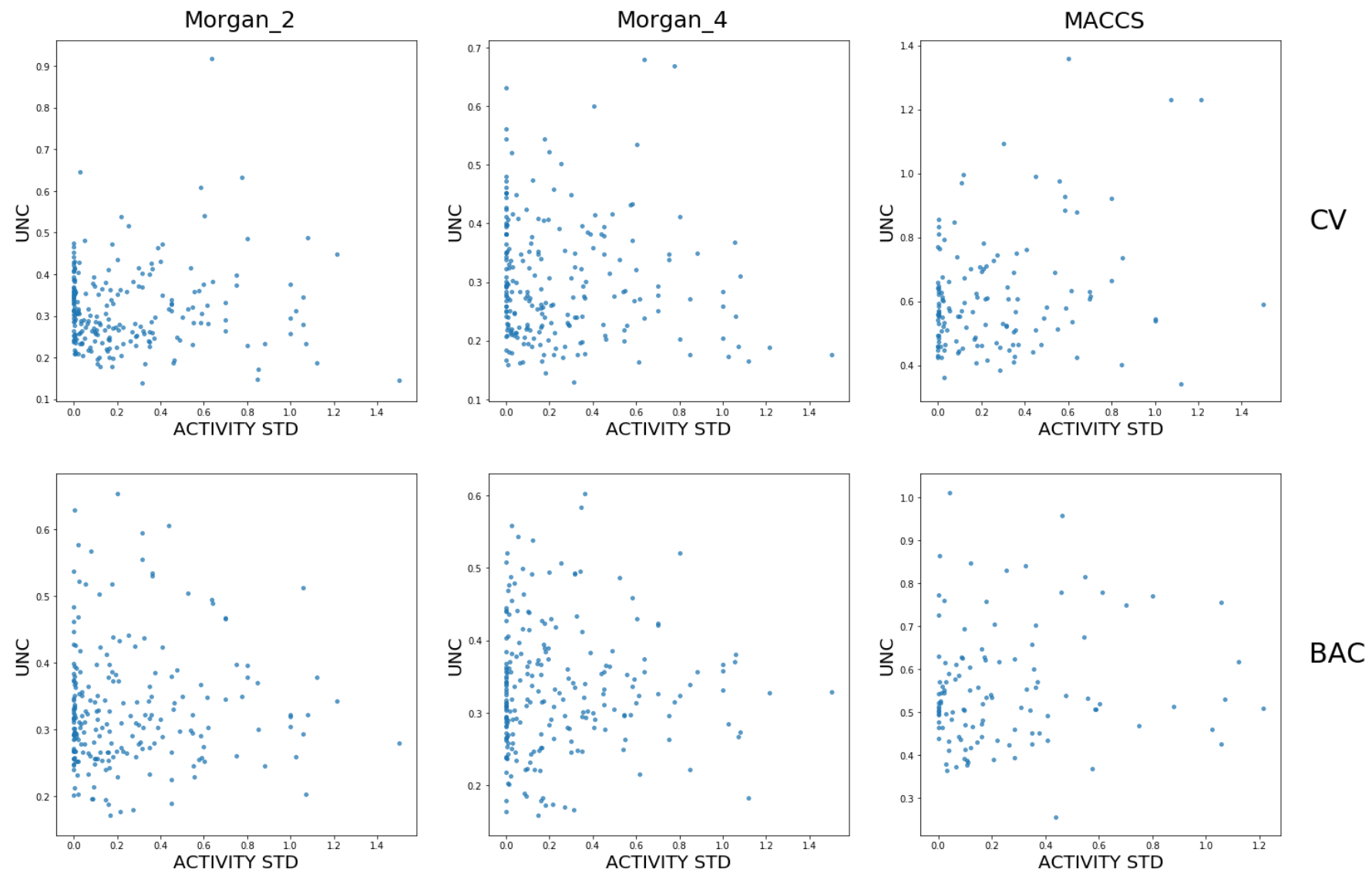

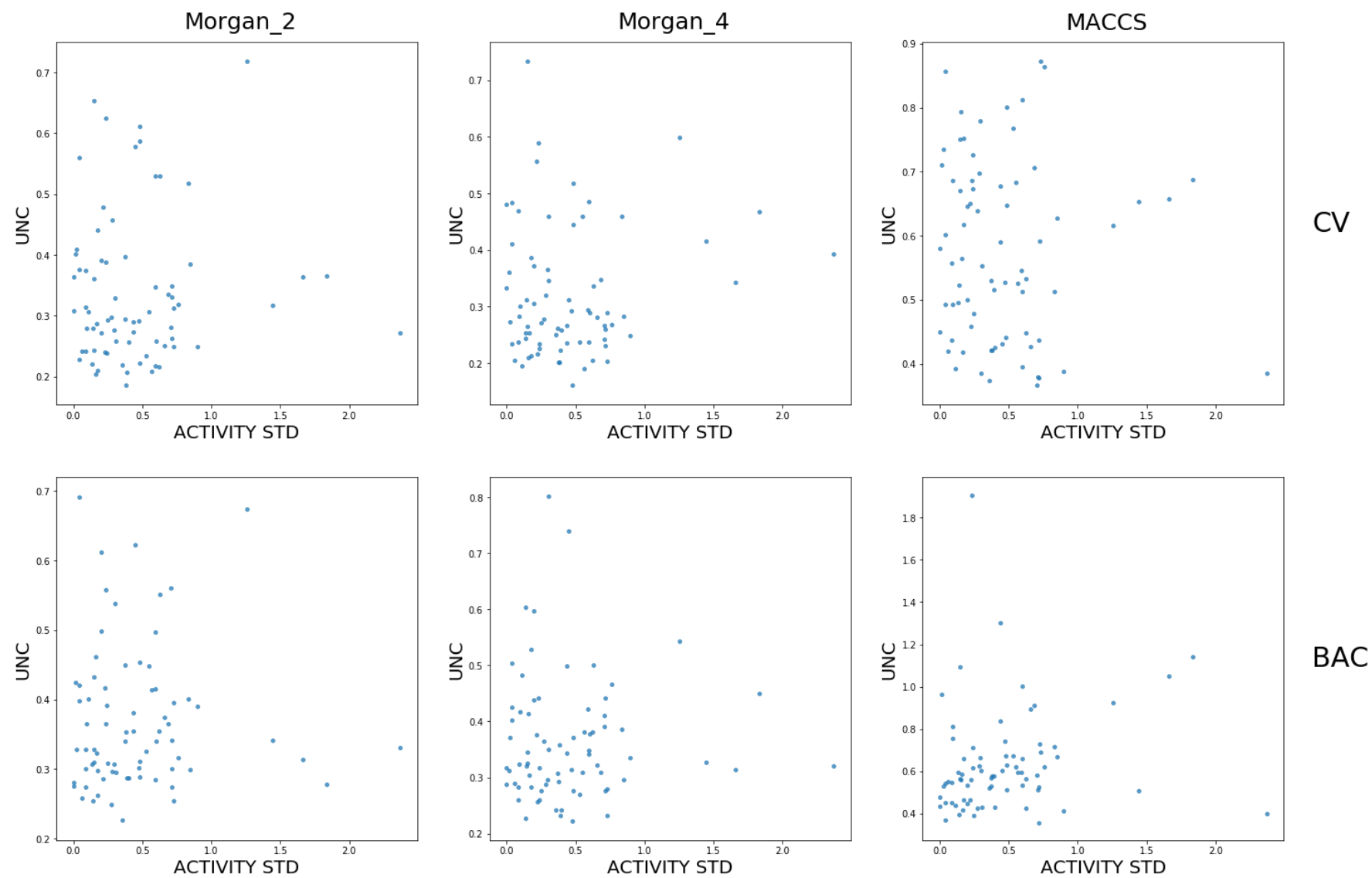

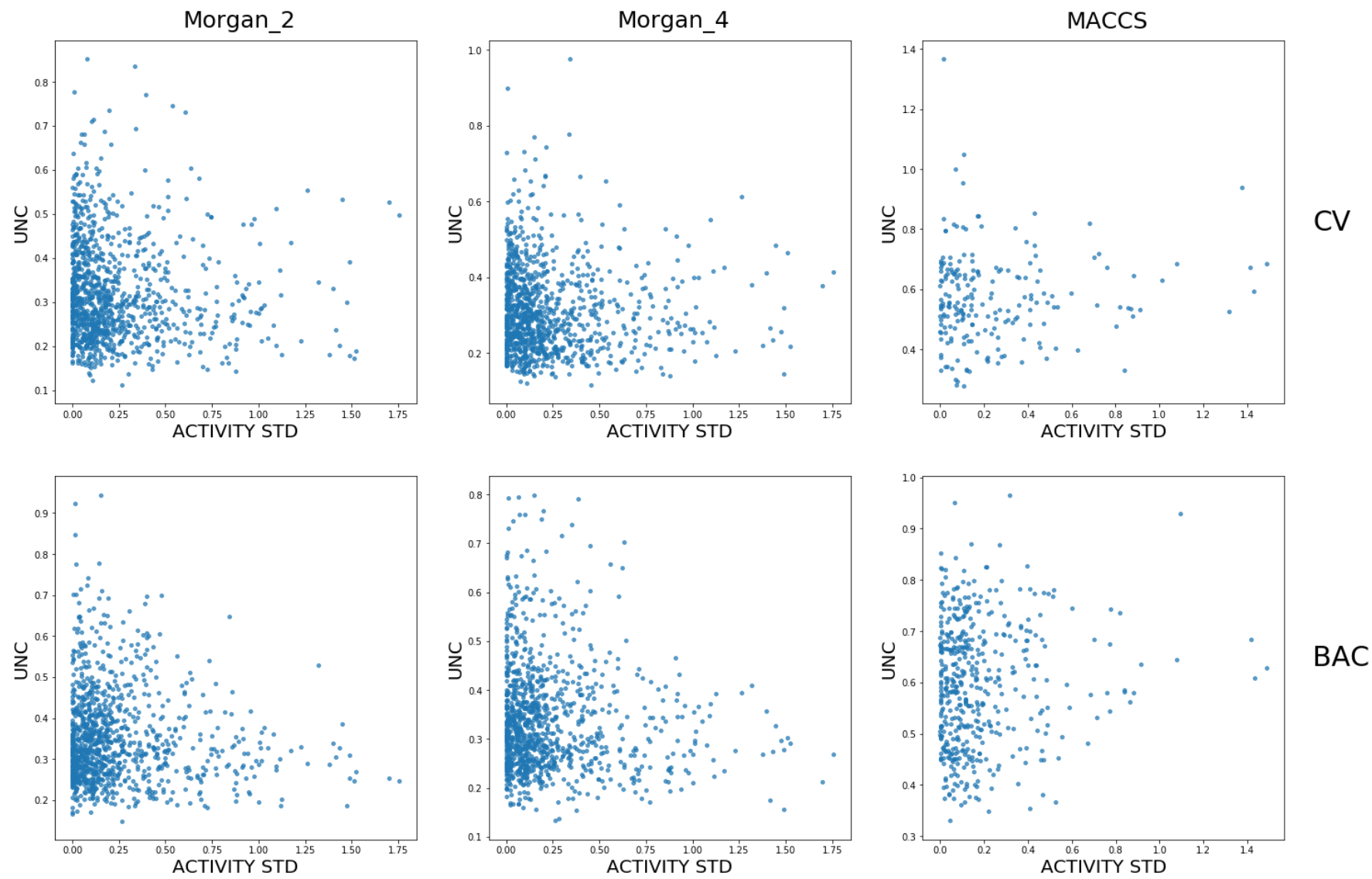

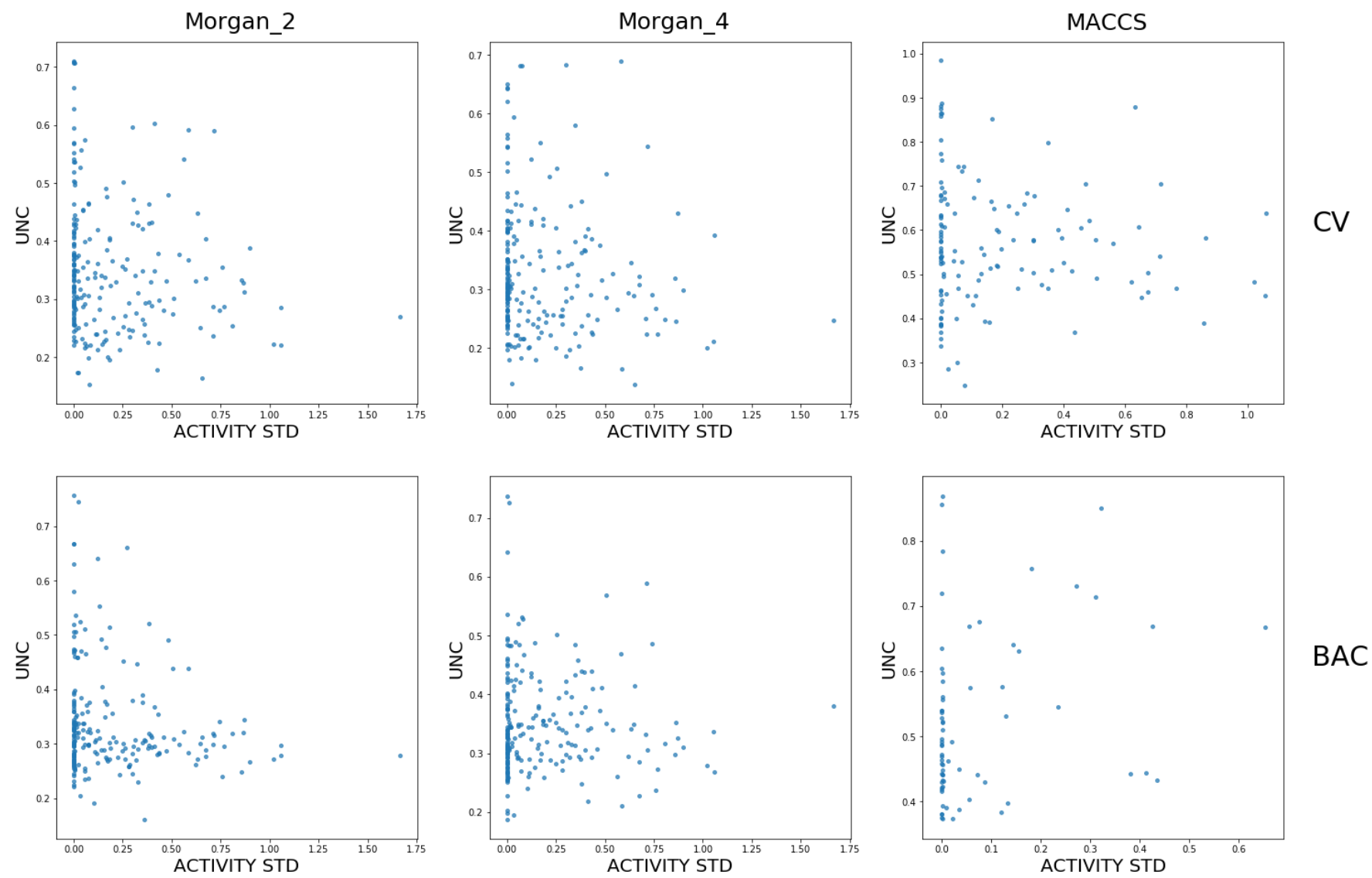

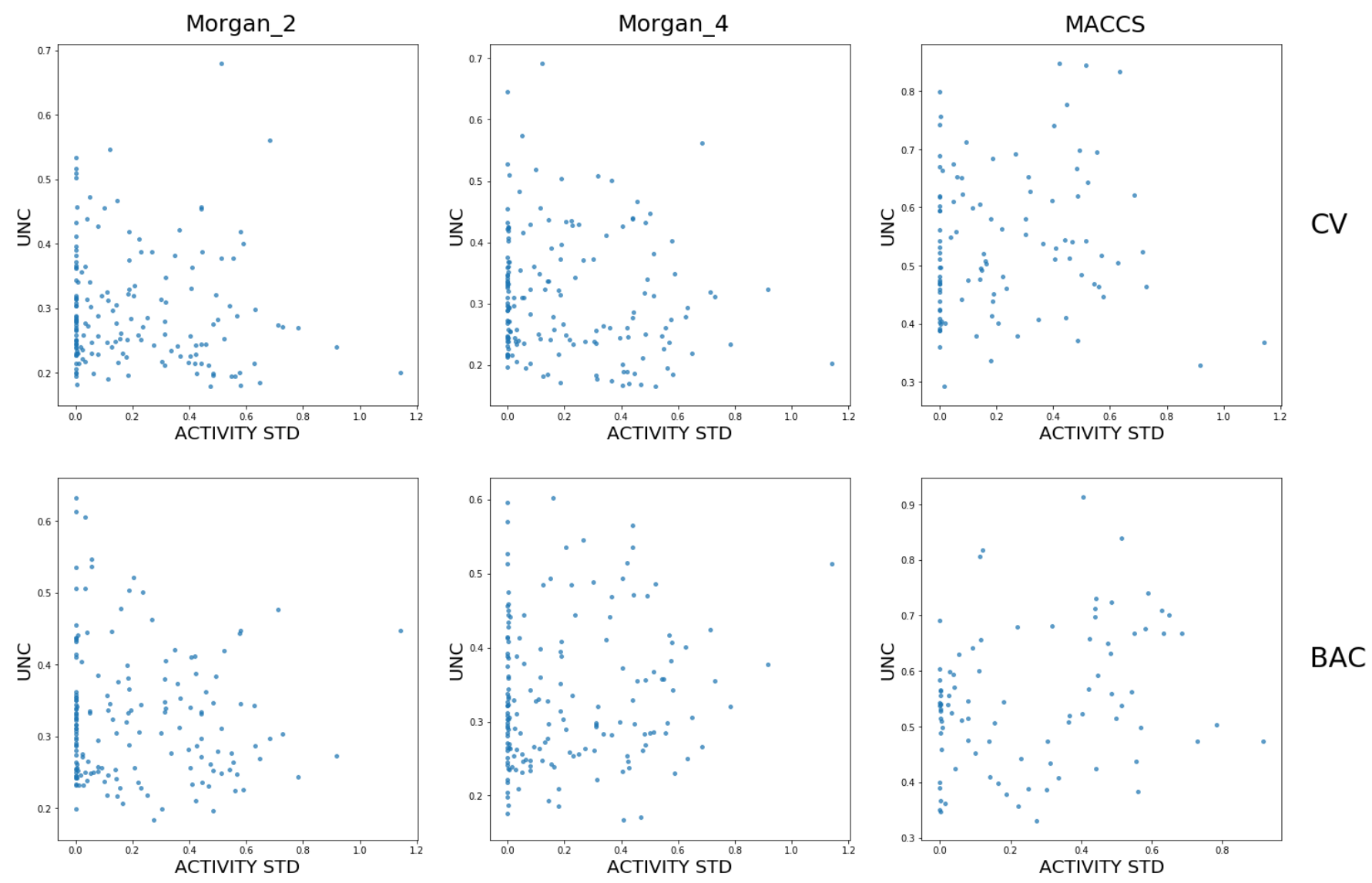

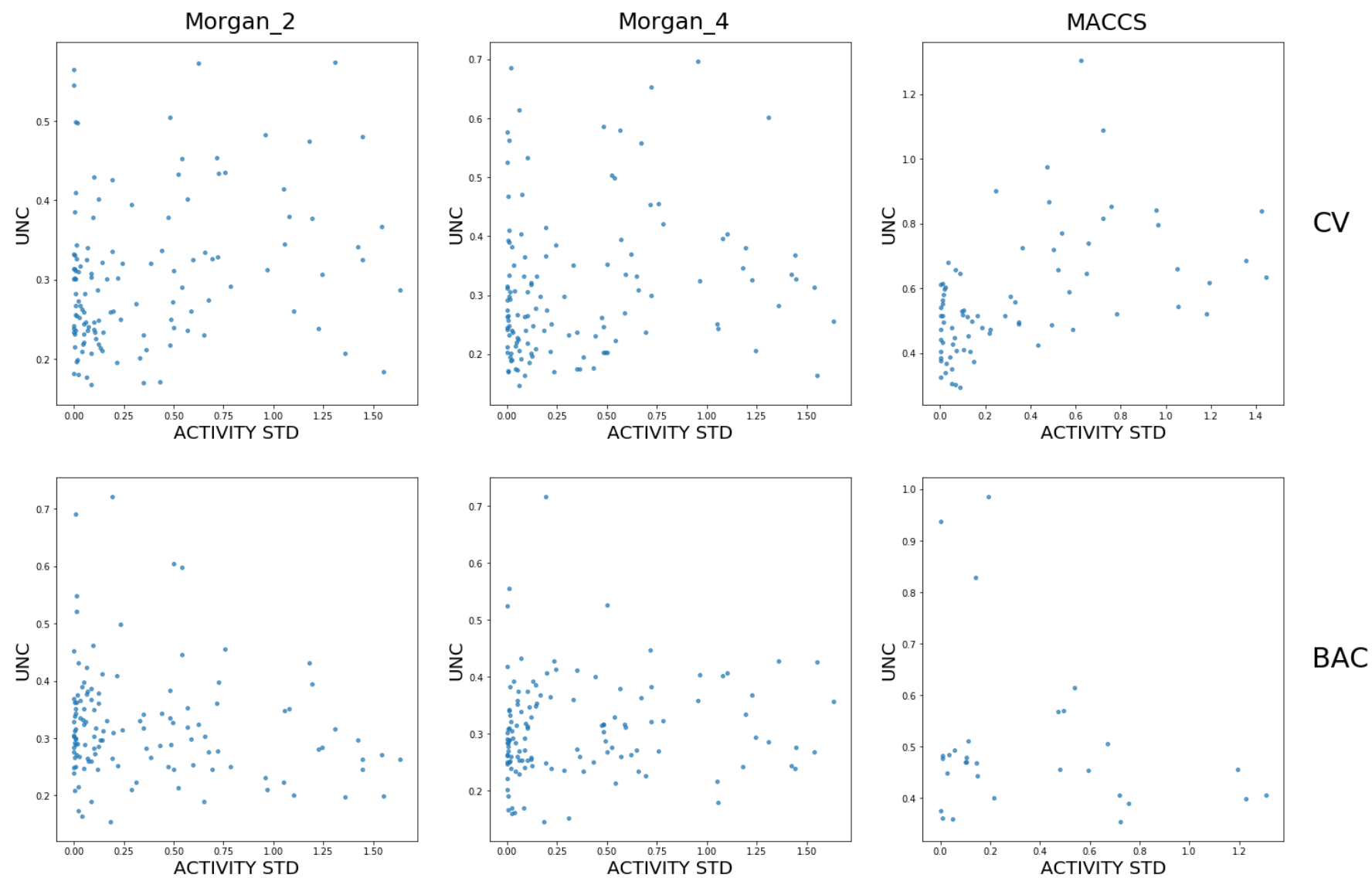

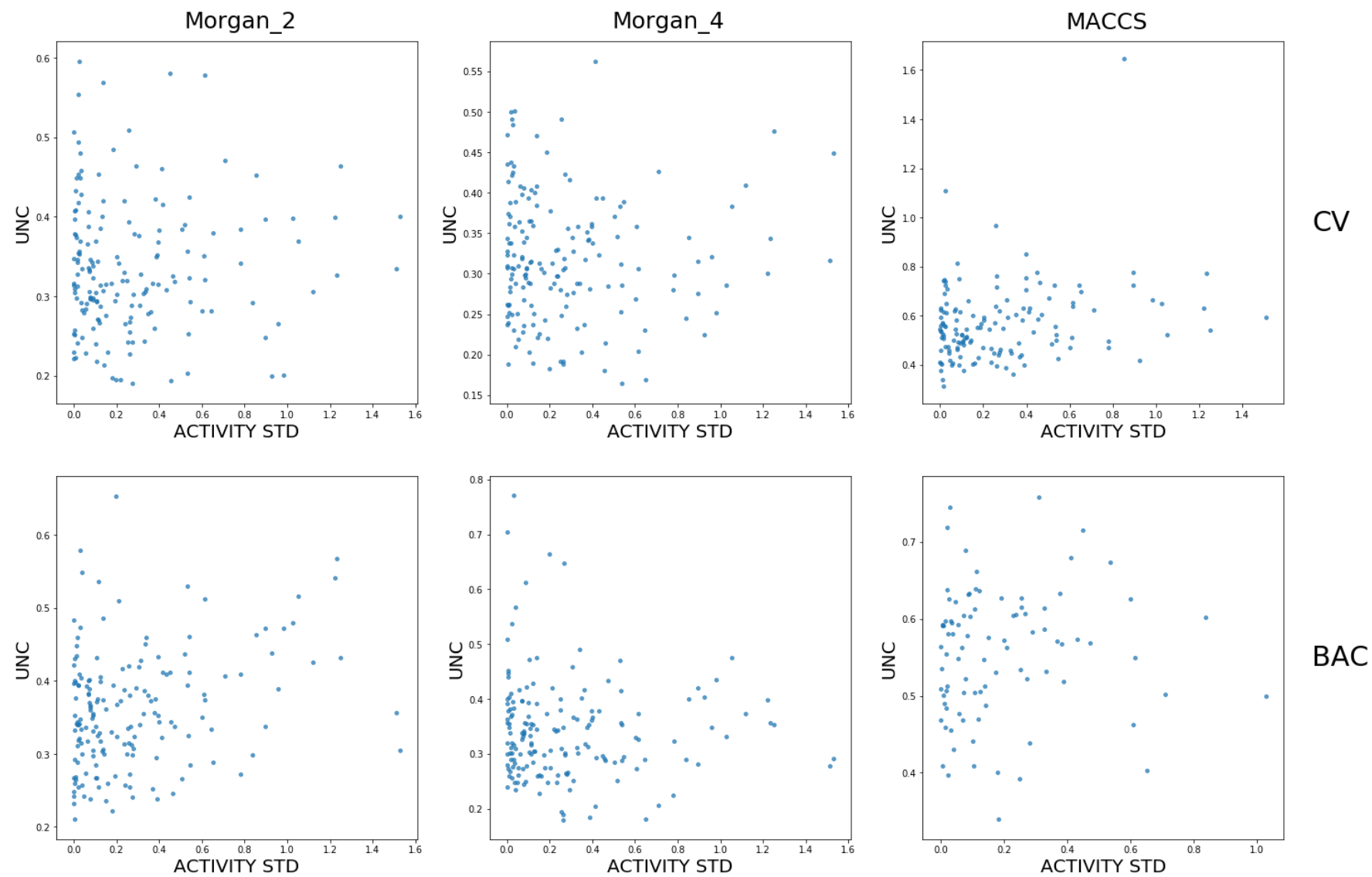

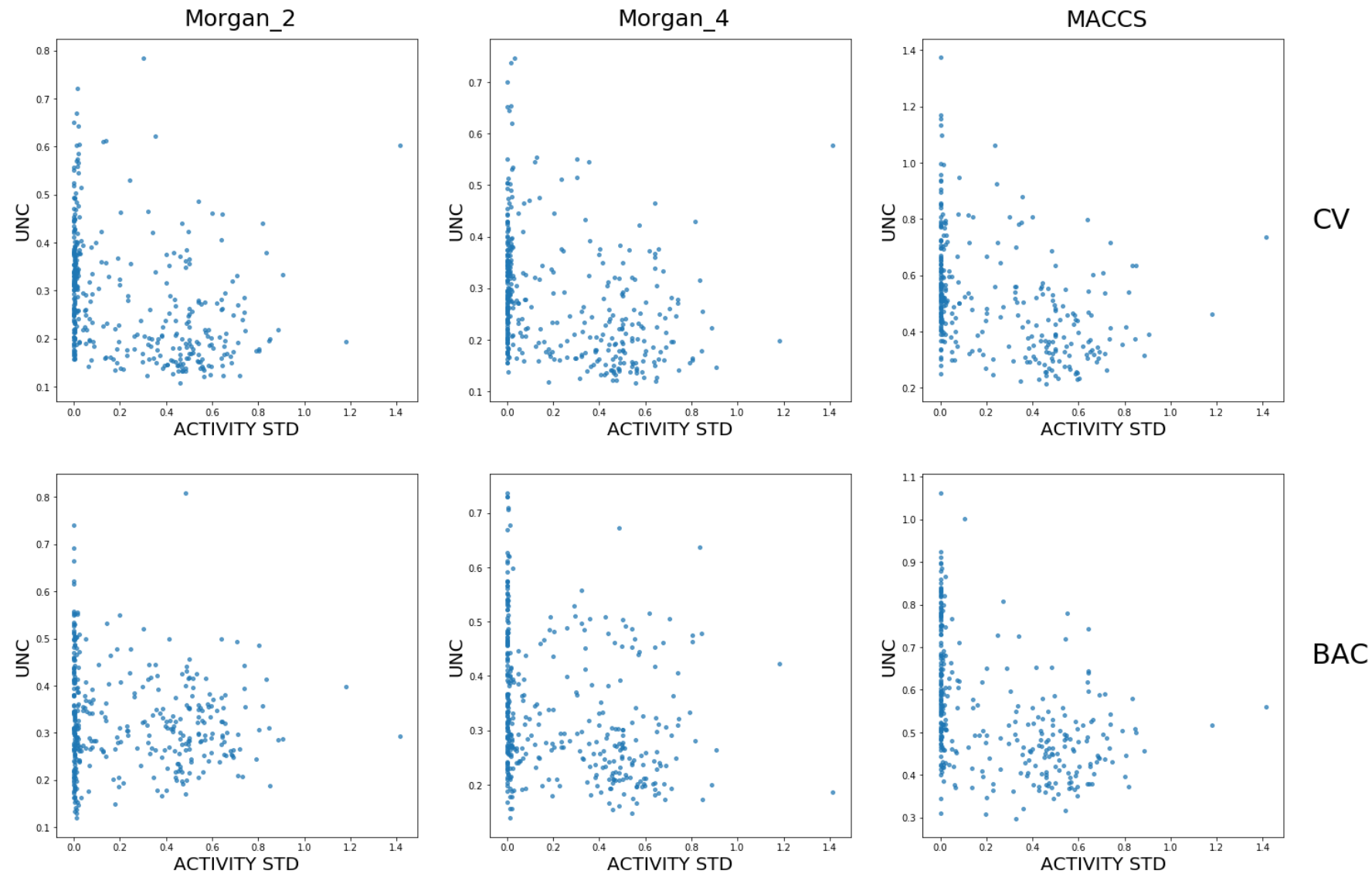

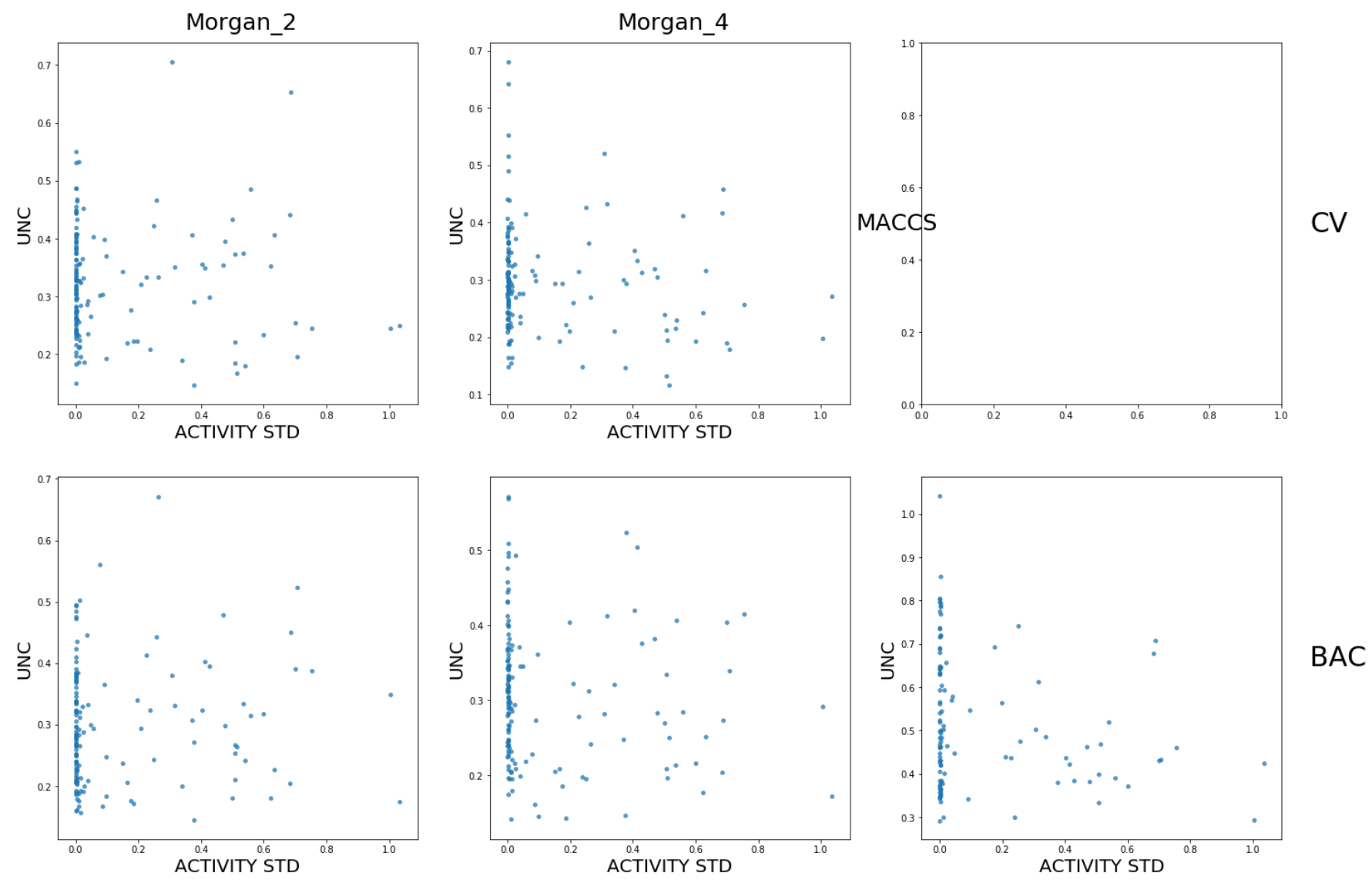

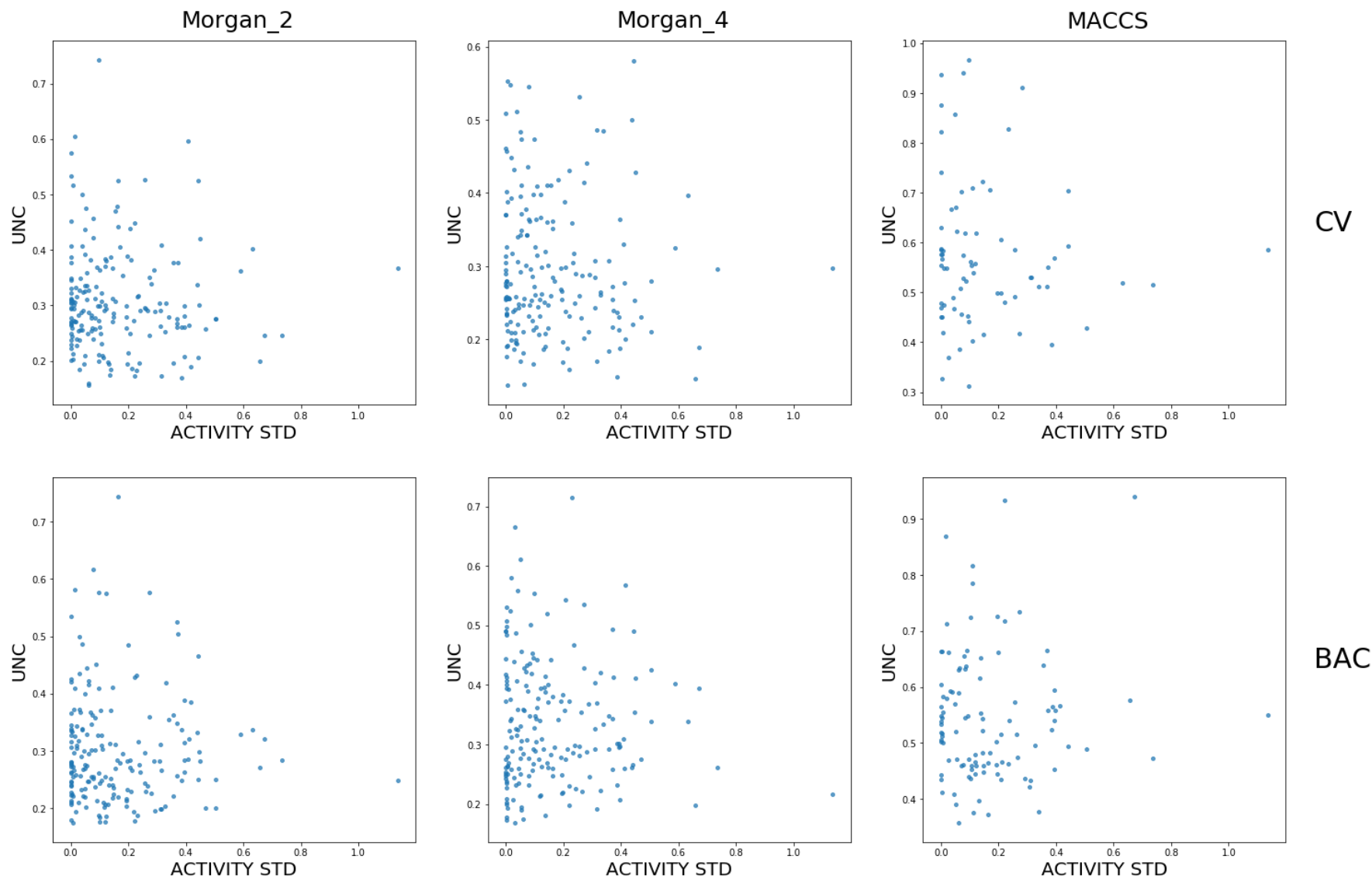

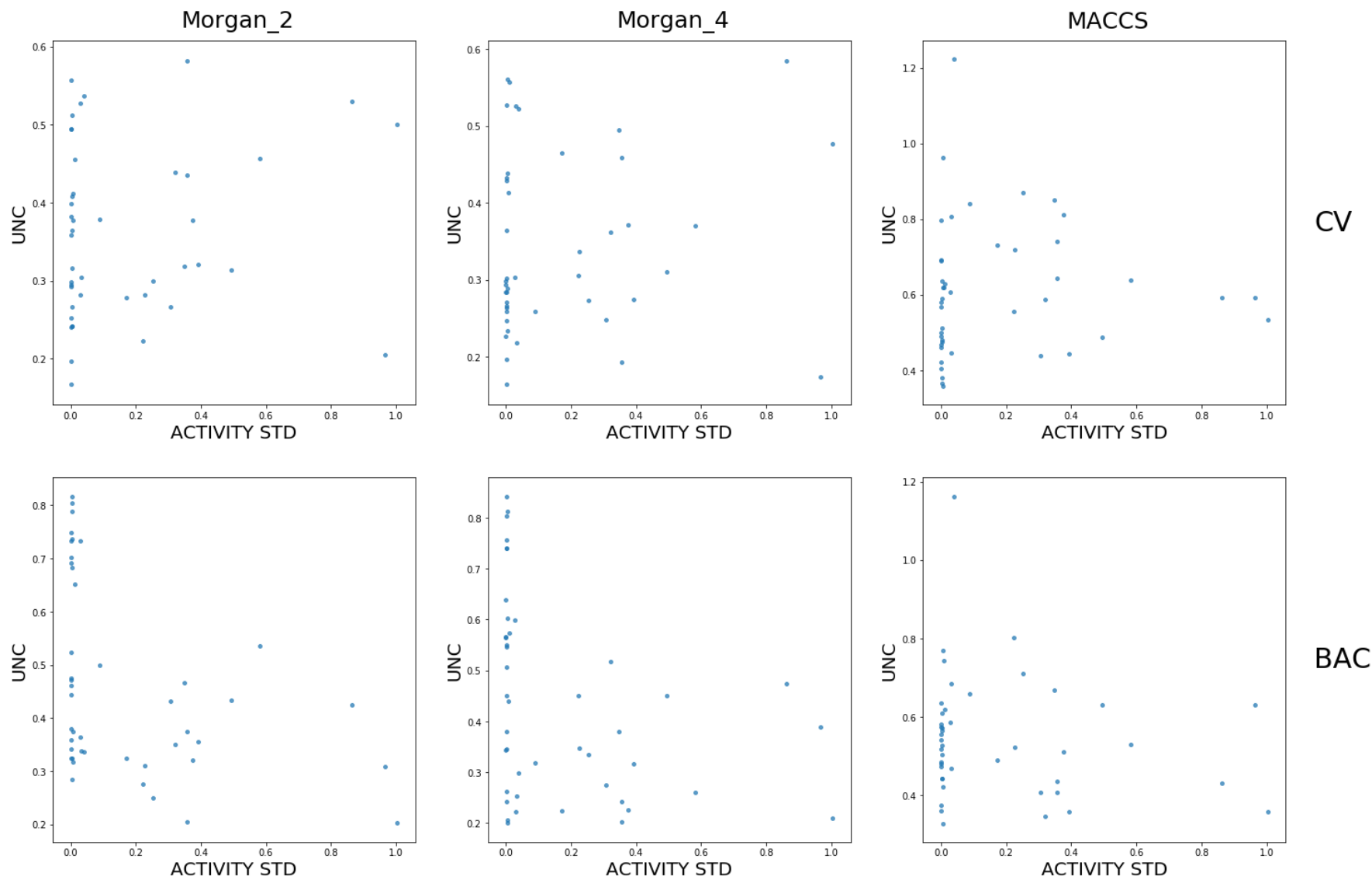

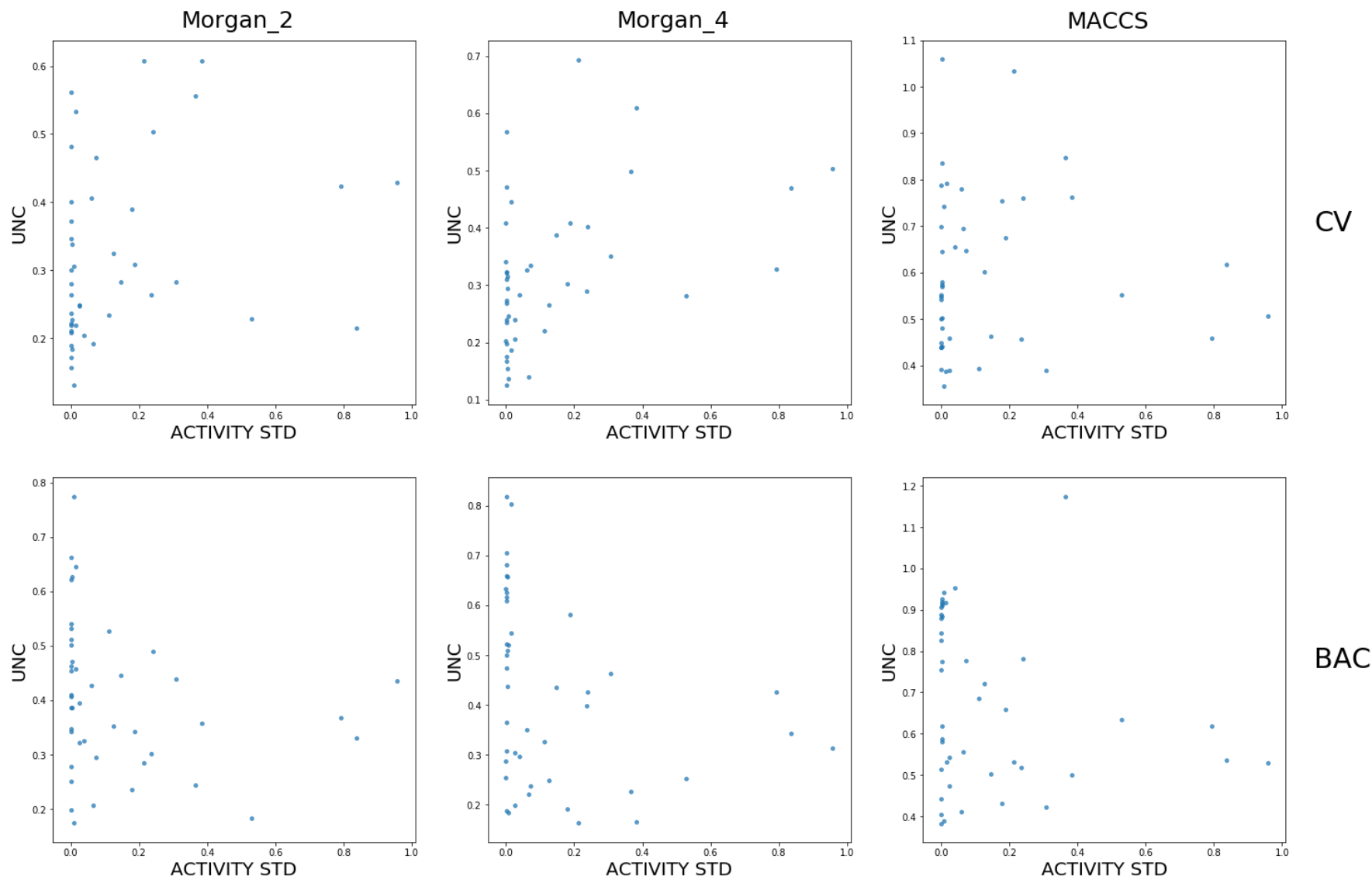

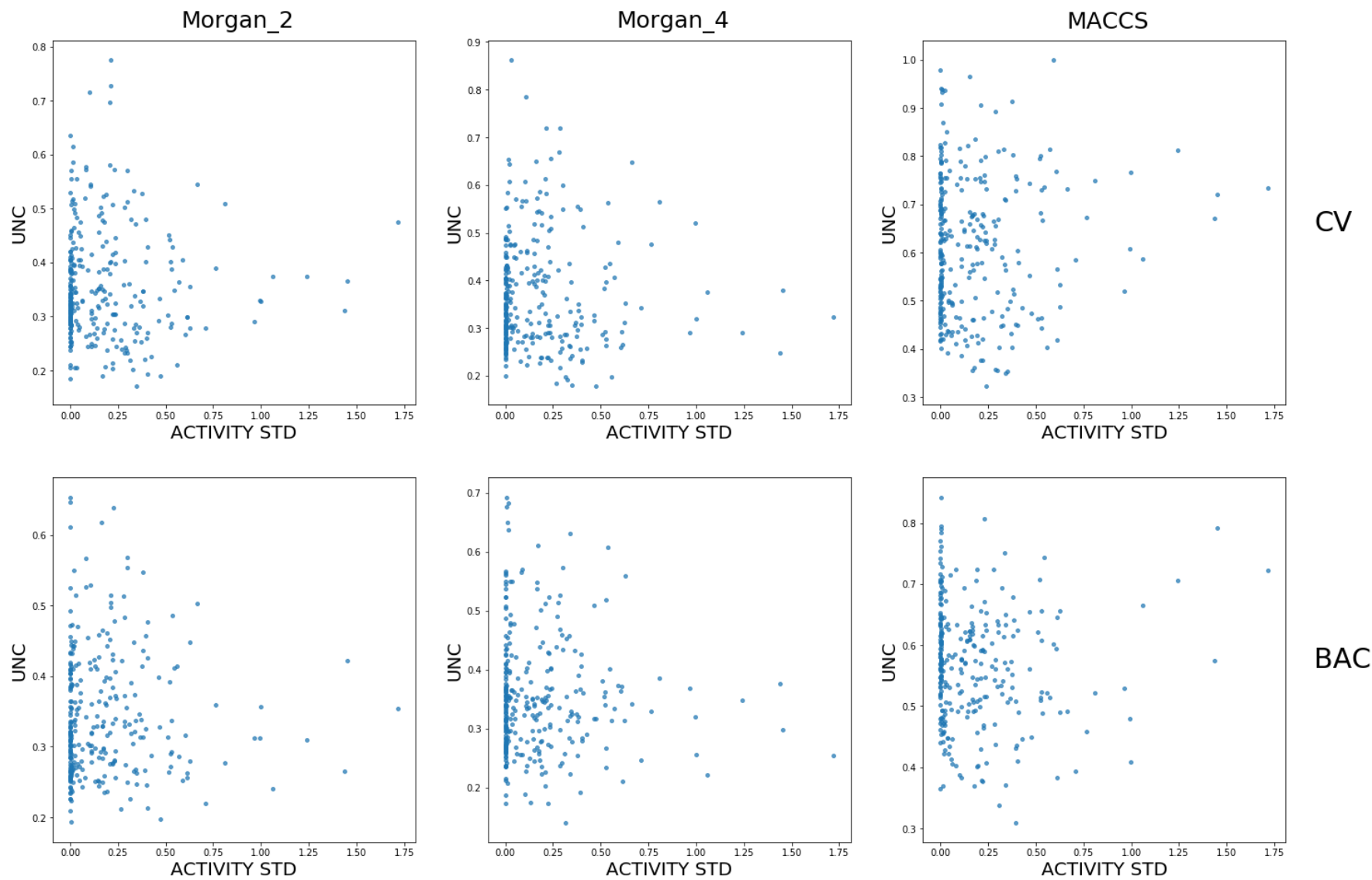

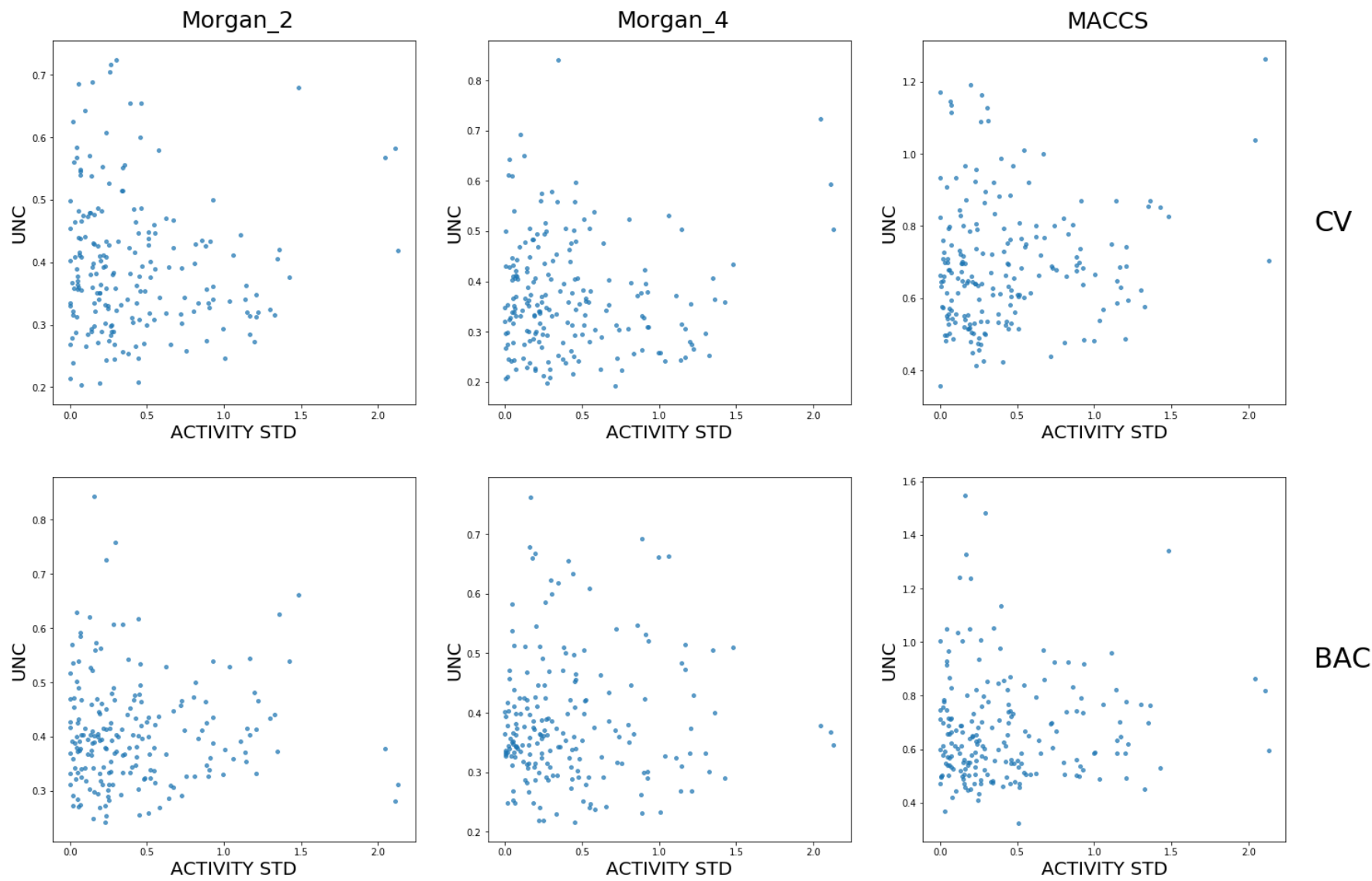

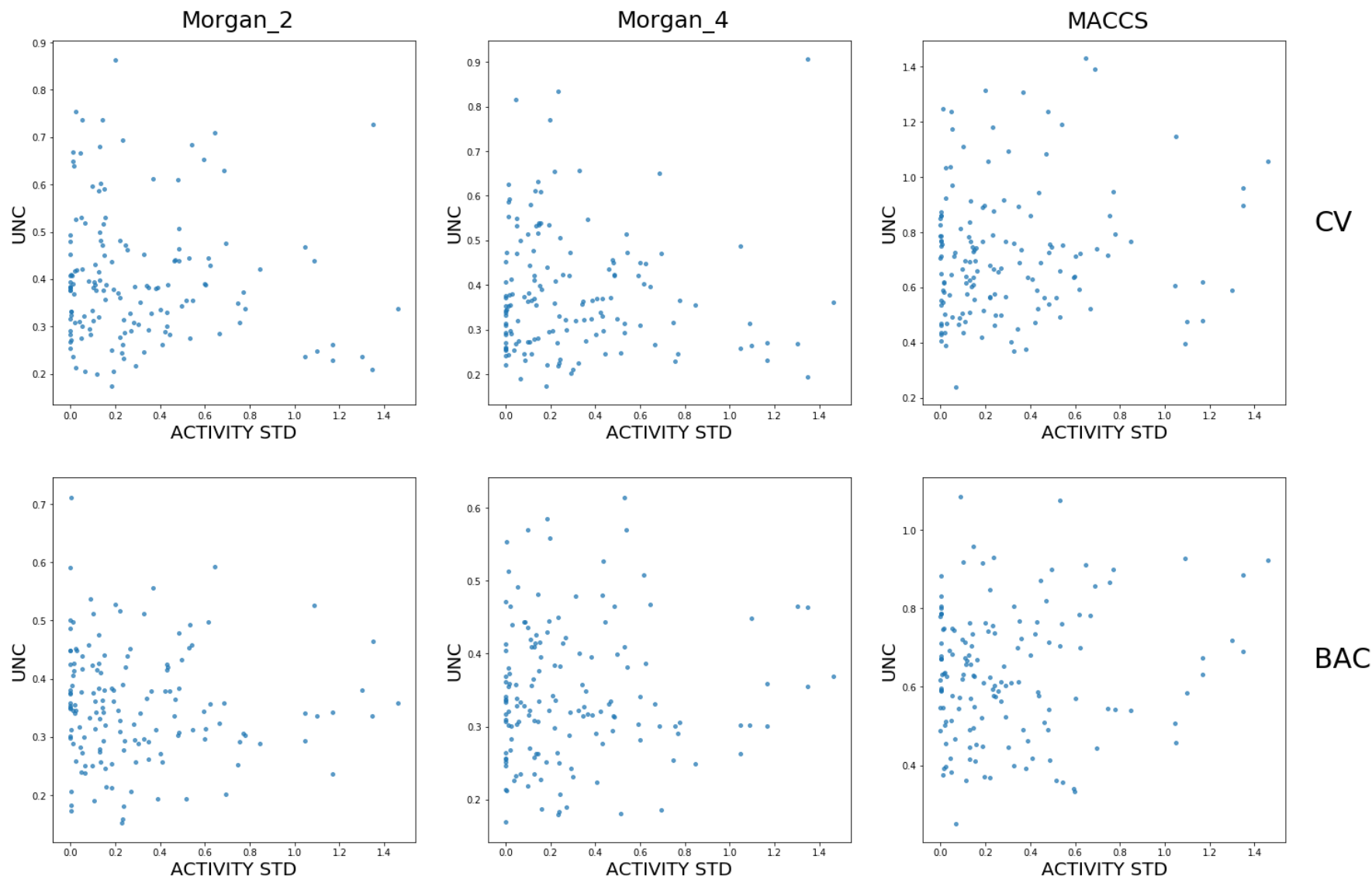

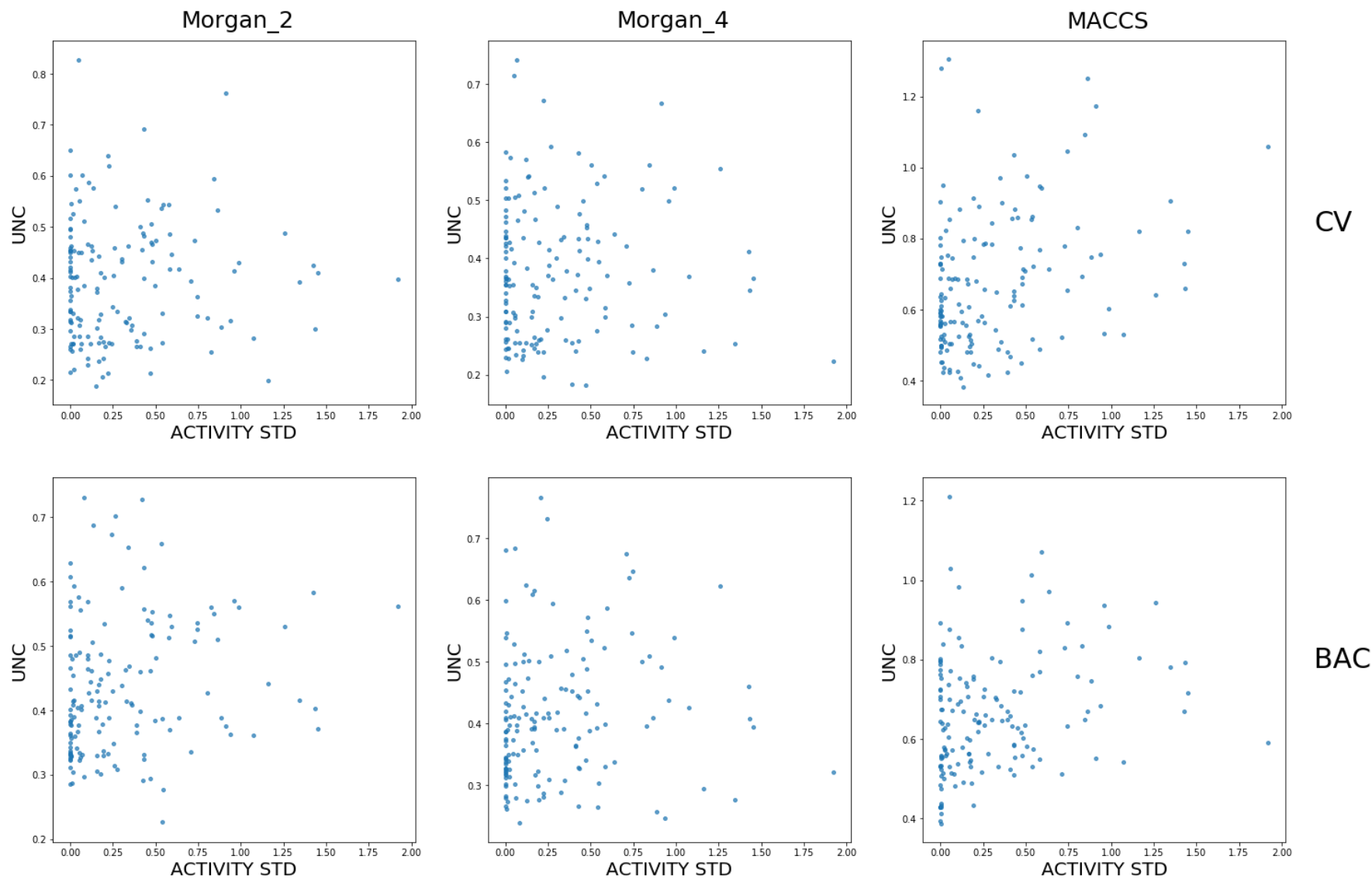

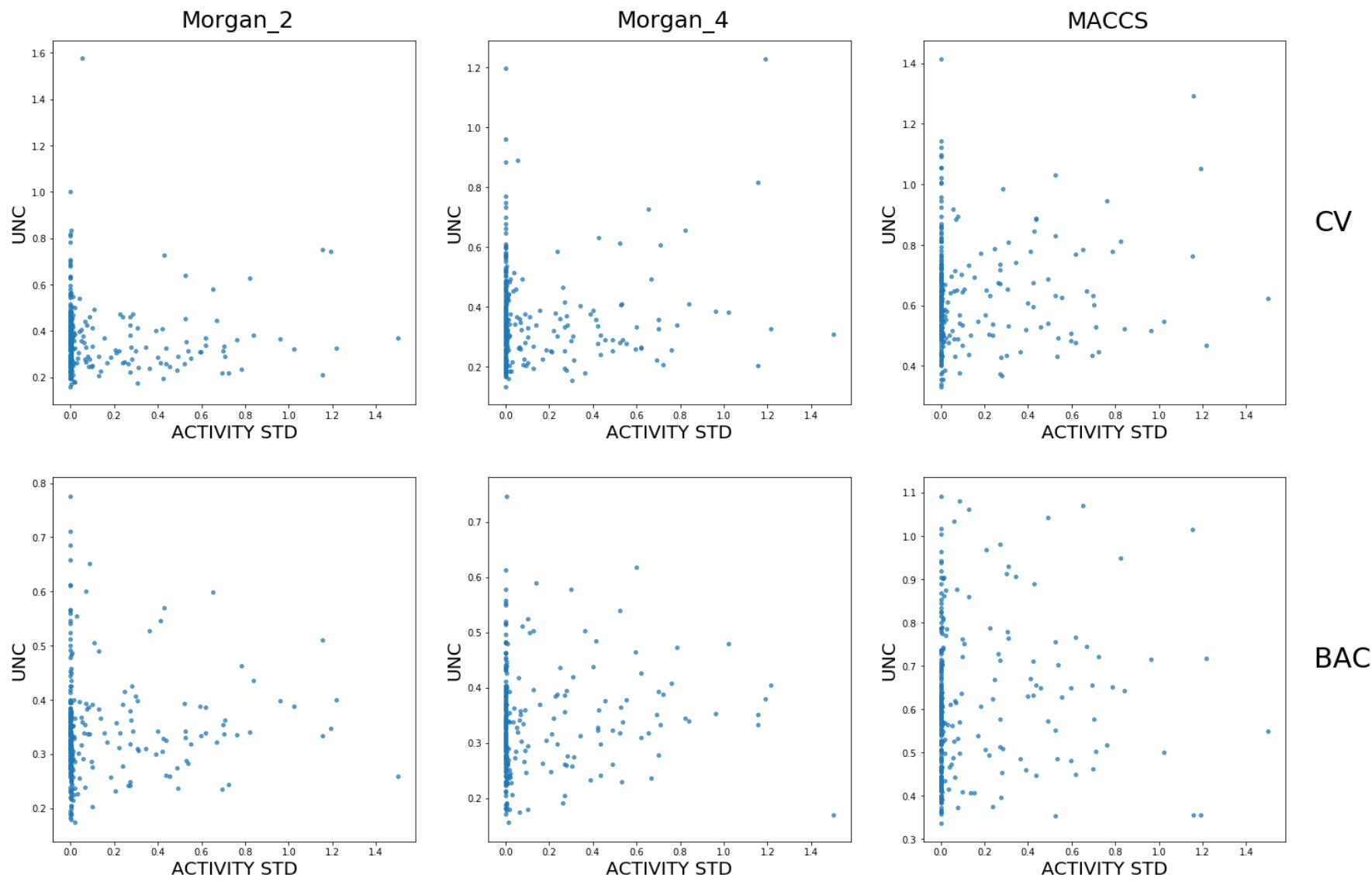

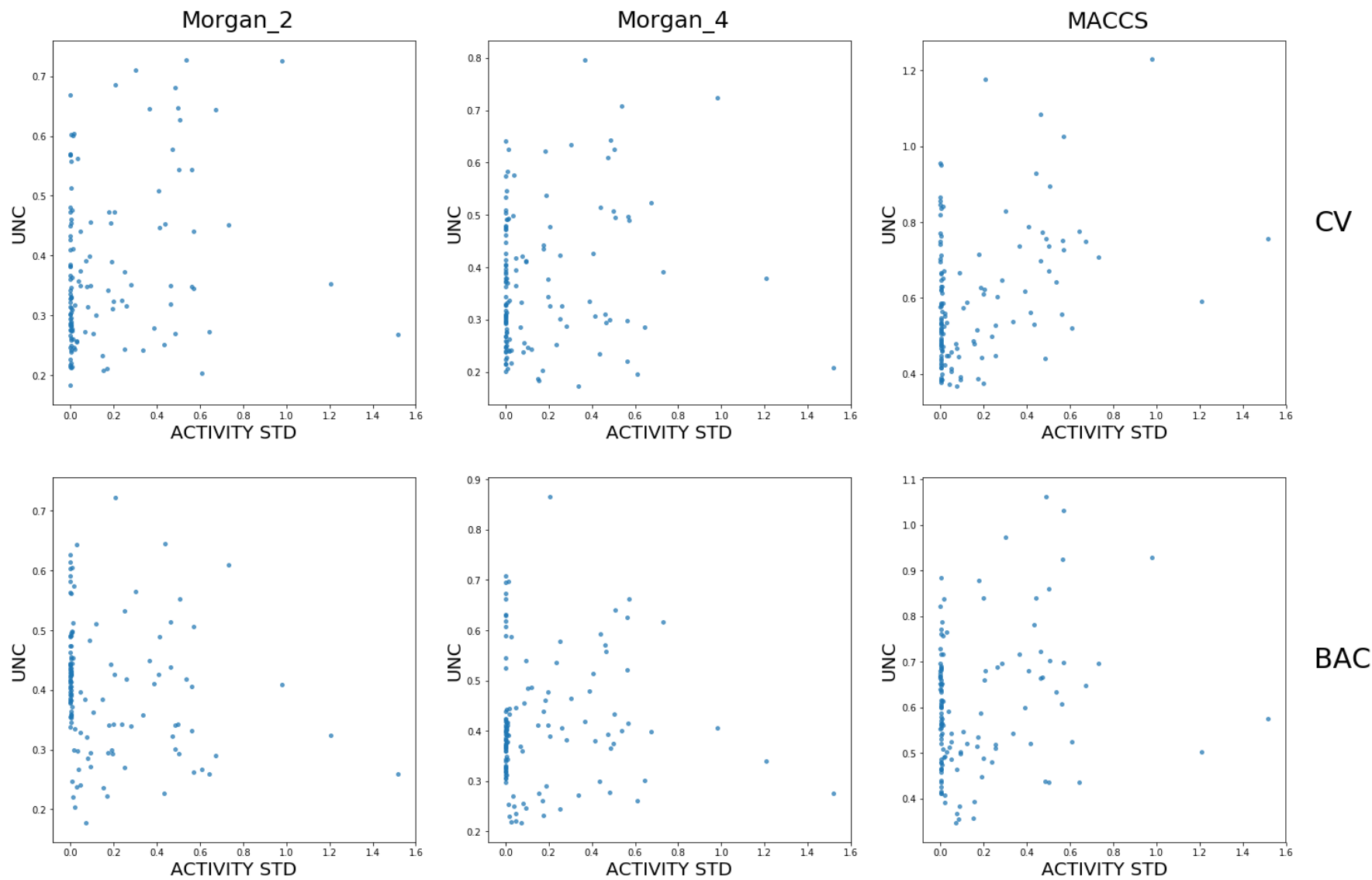

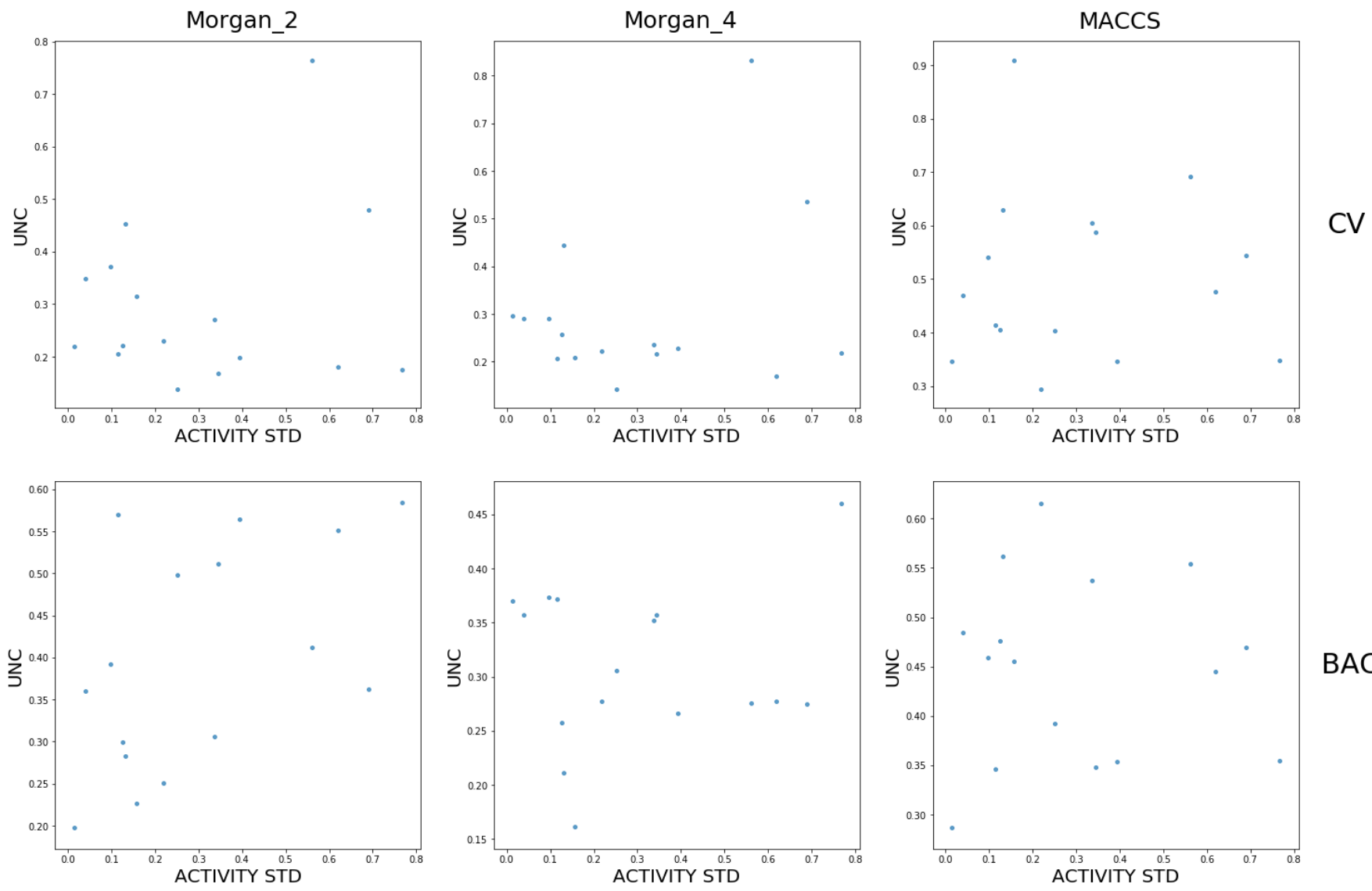

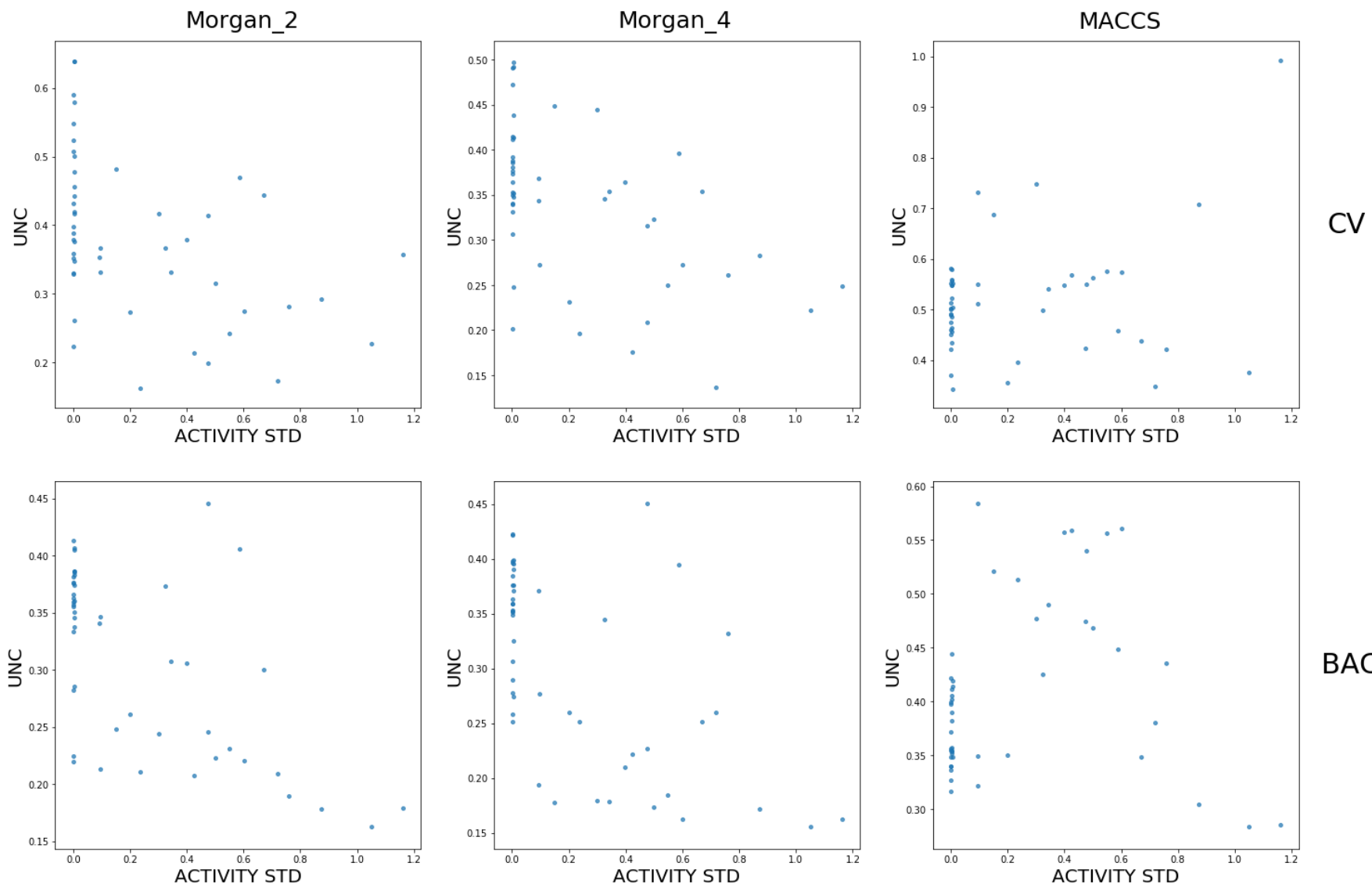

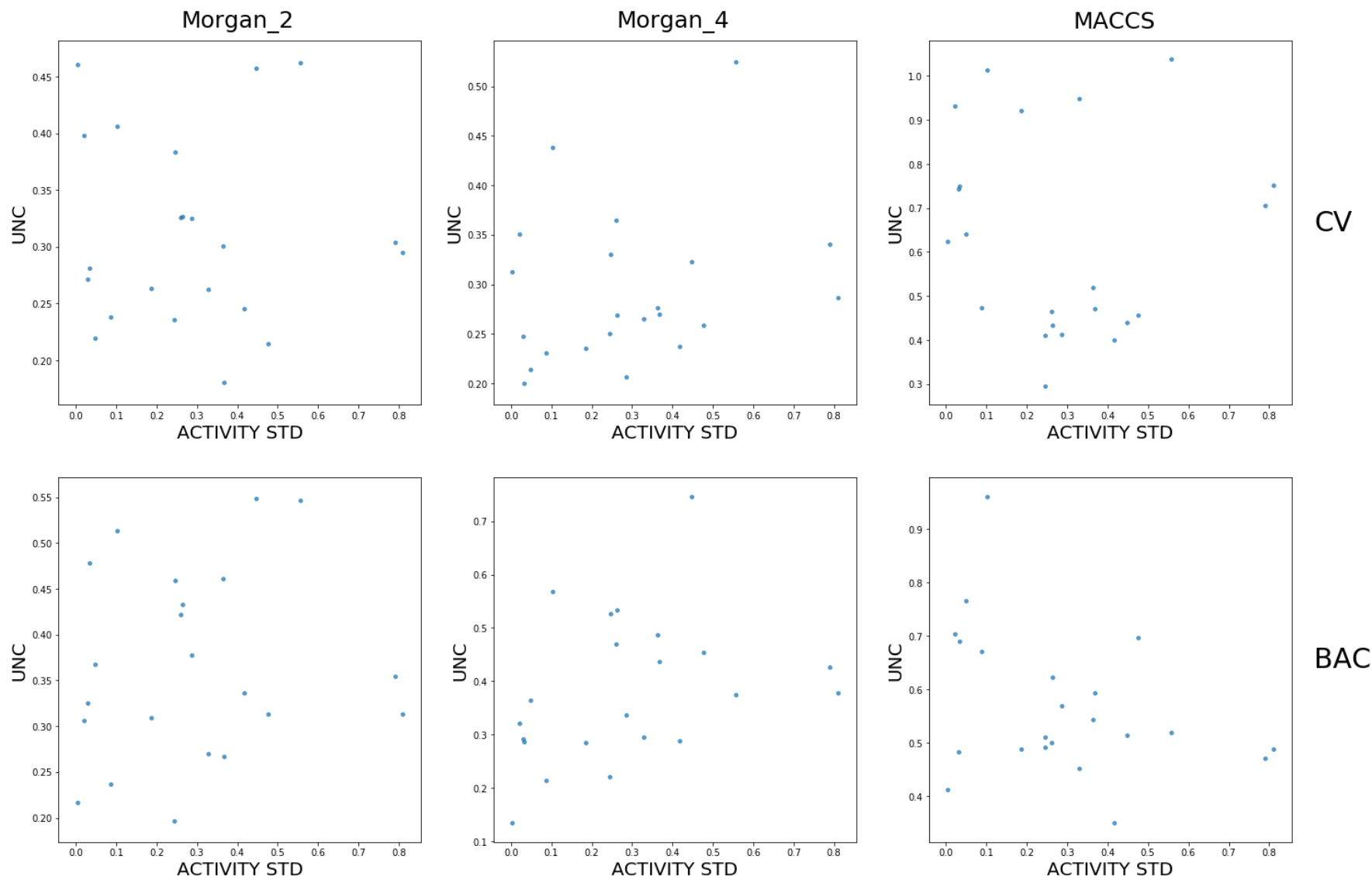

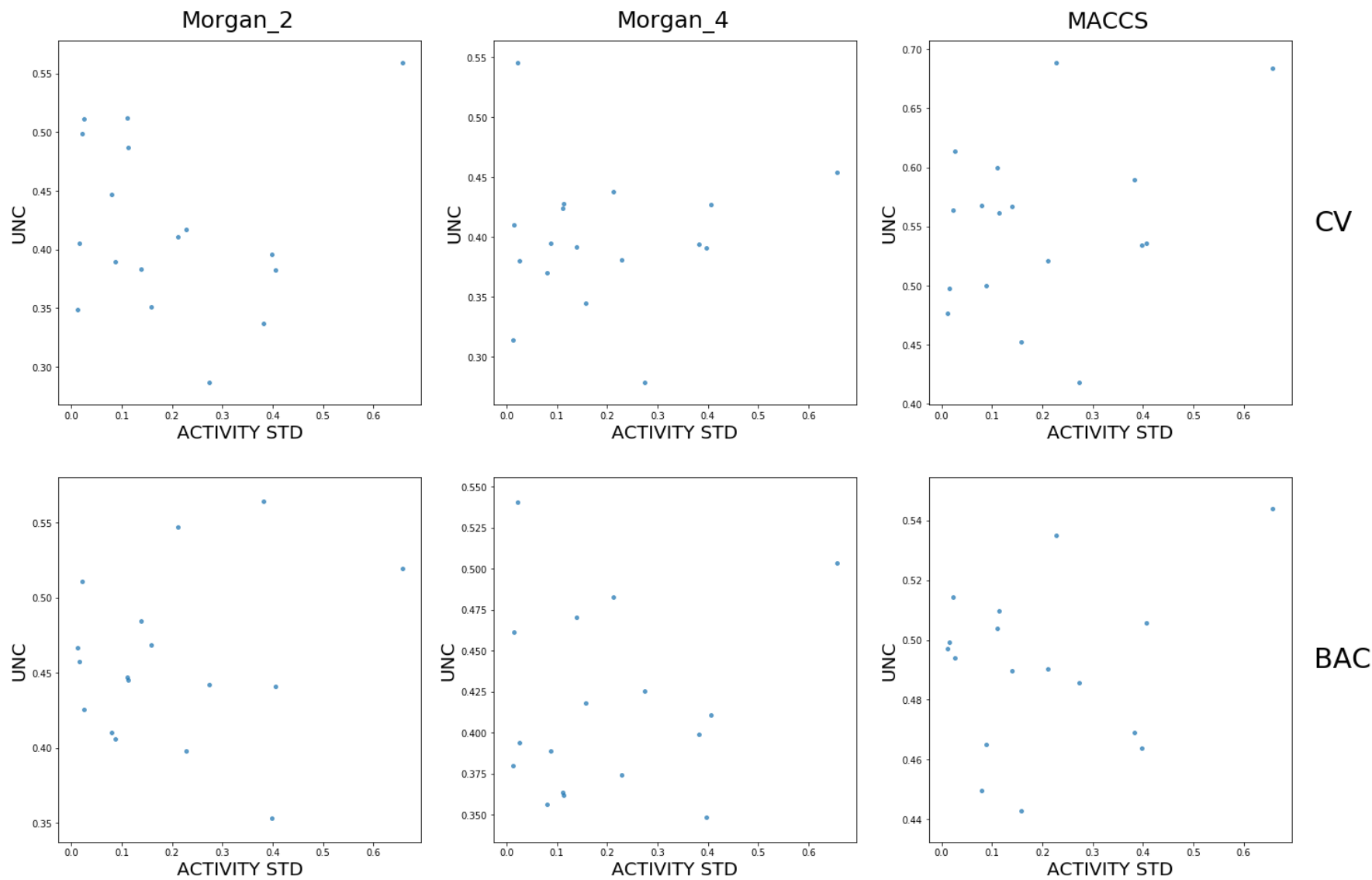

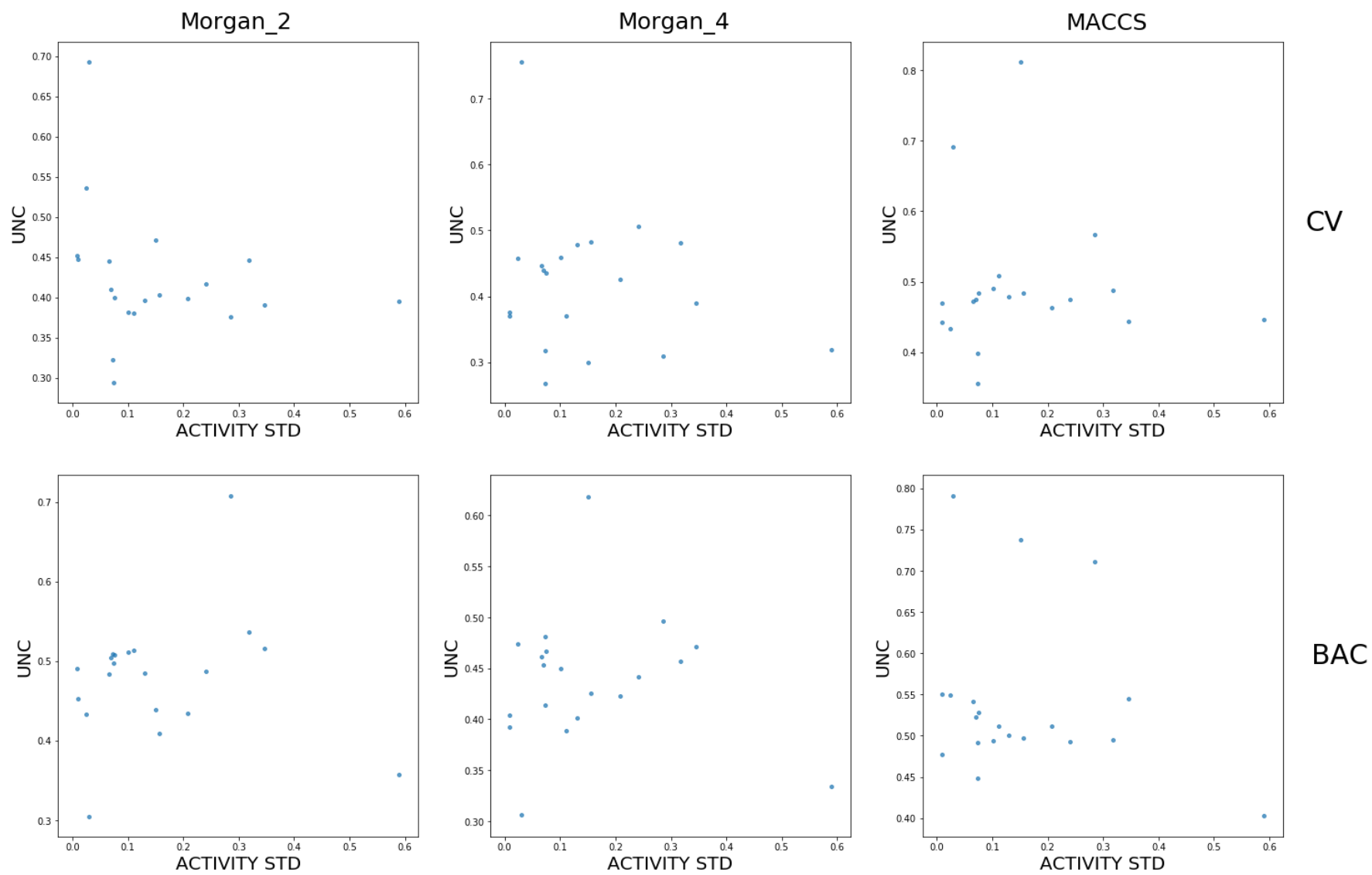

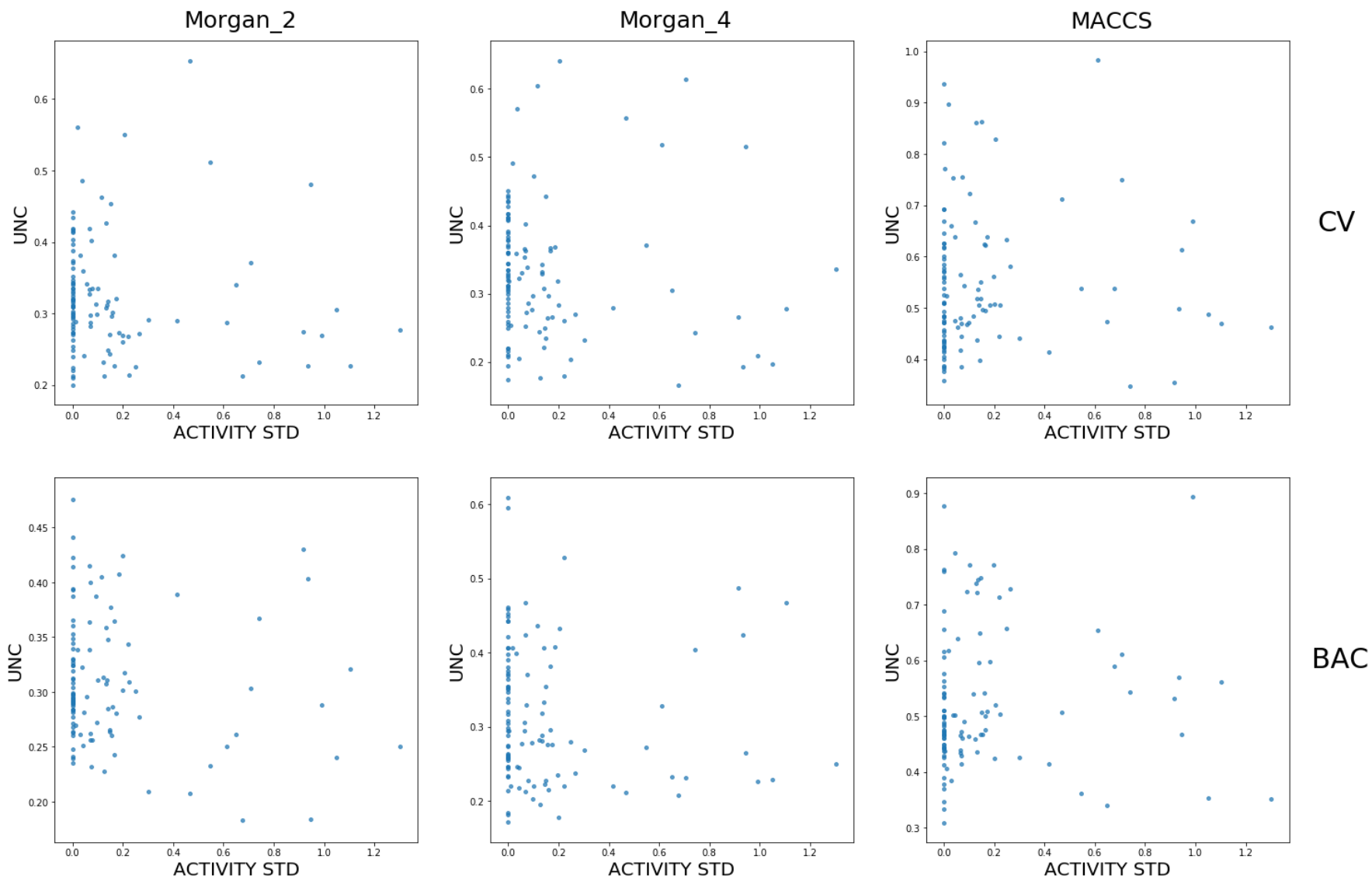

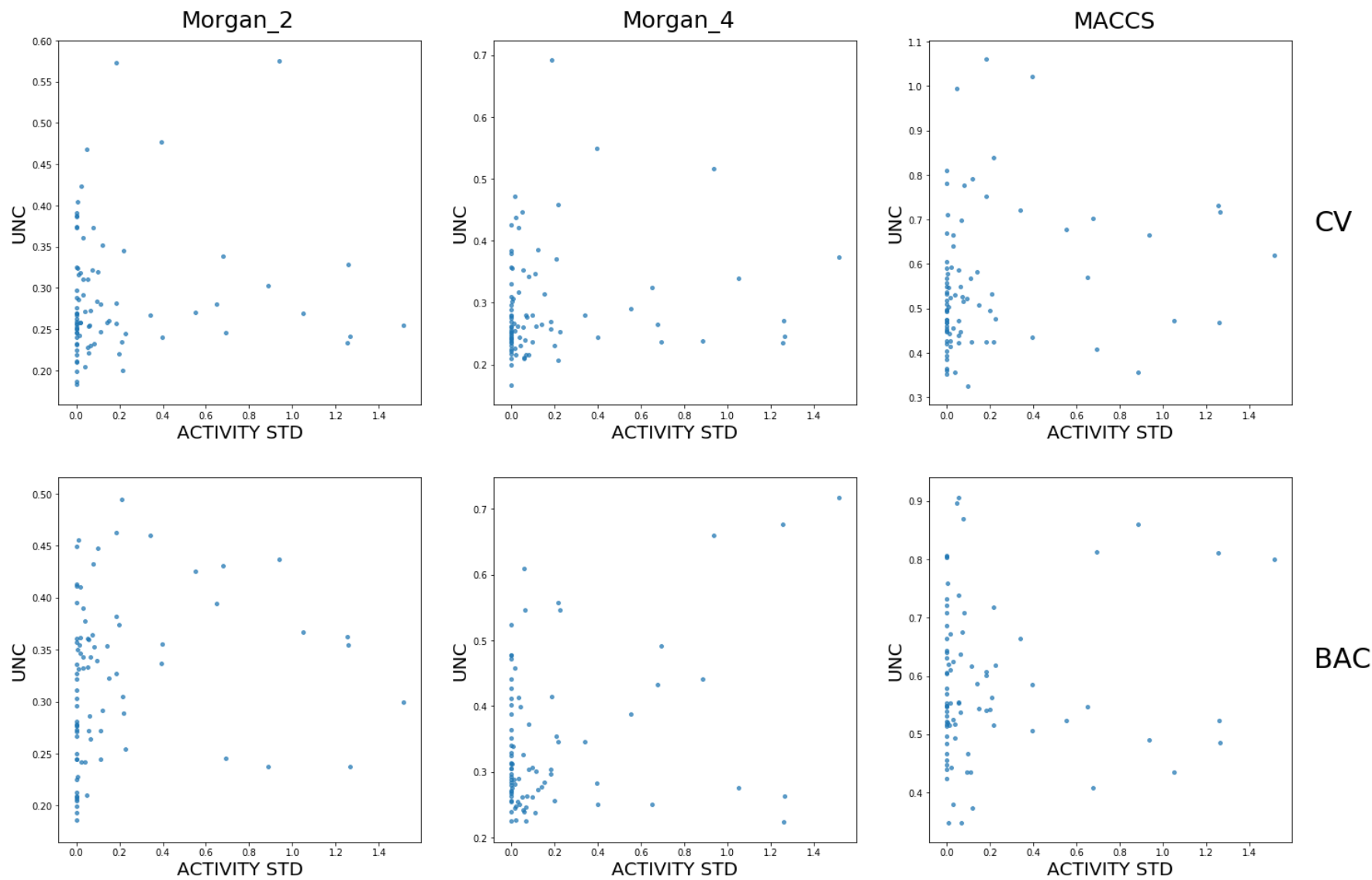

Supplement: Supplementary file 1 [file molecules-25-01452-s001.zip › Supp_Info_for_submission/FileS3.pdf]

## CHEMBL214

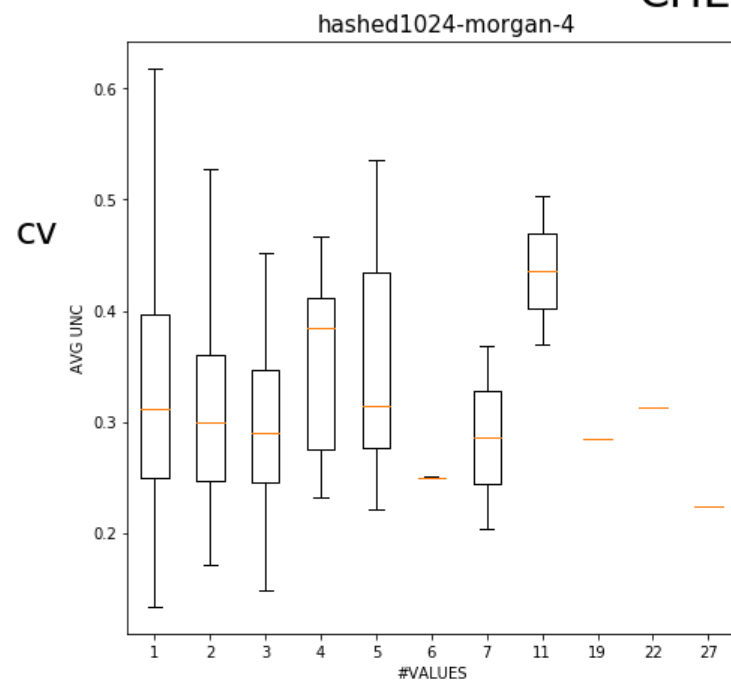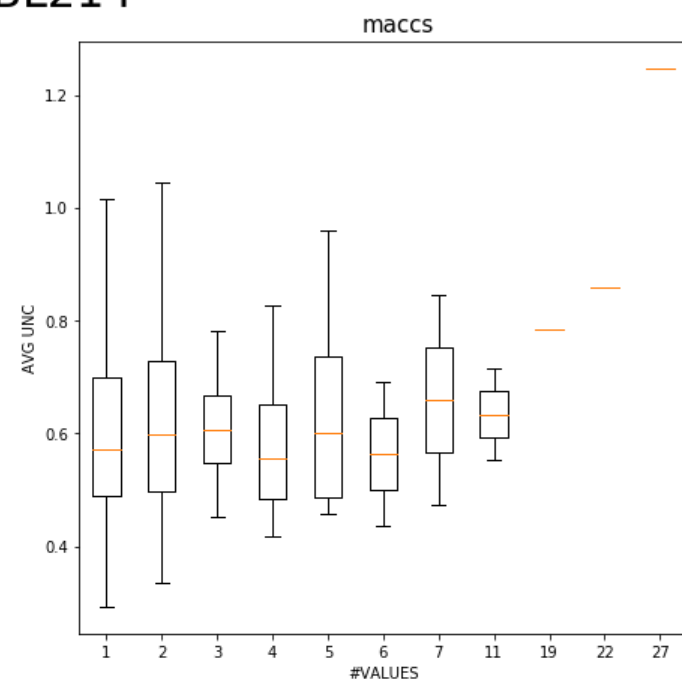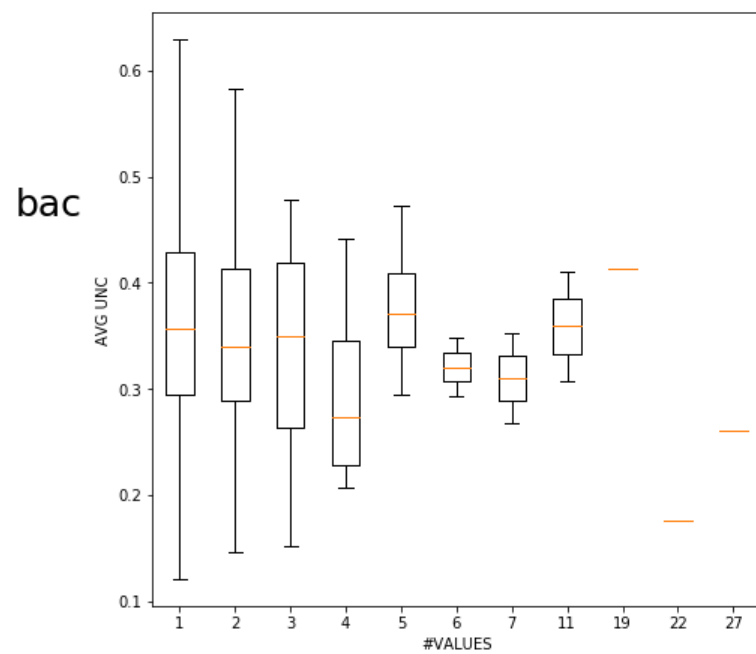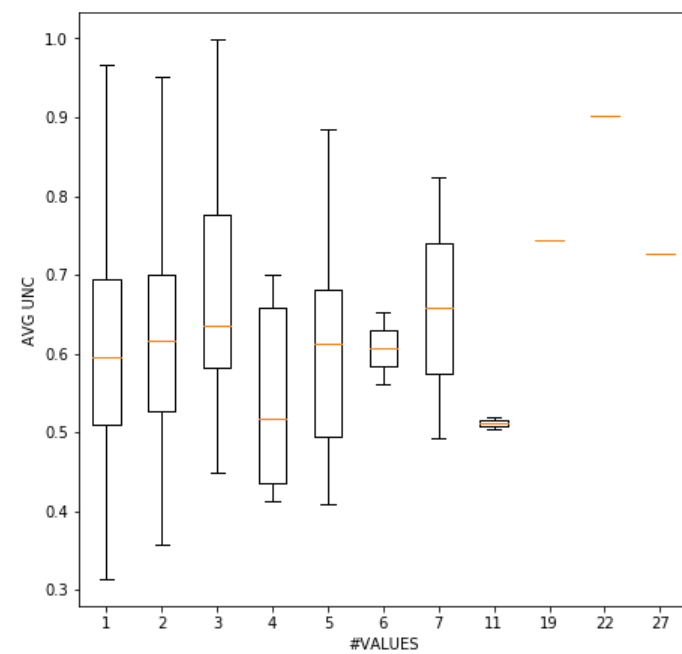

# CHEMBL216

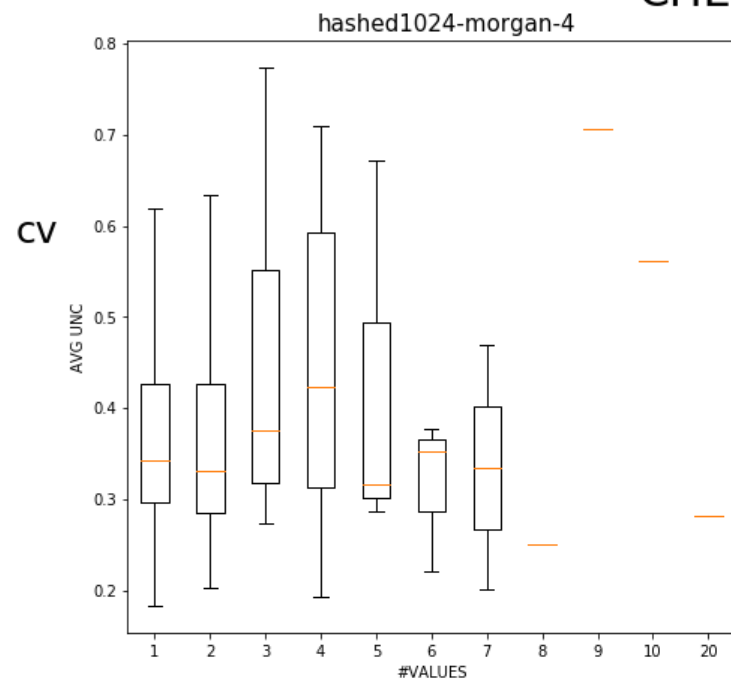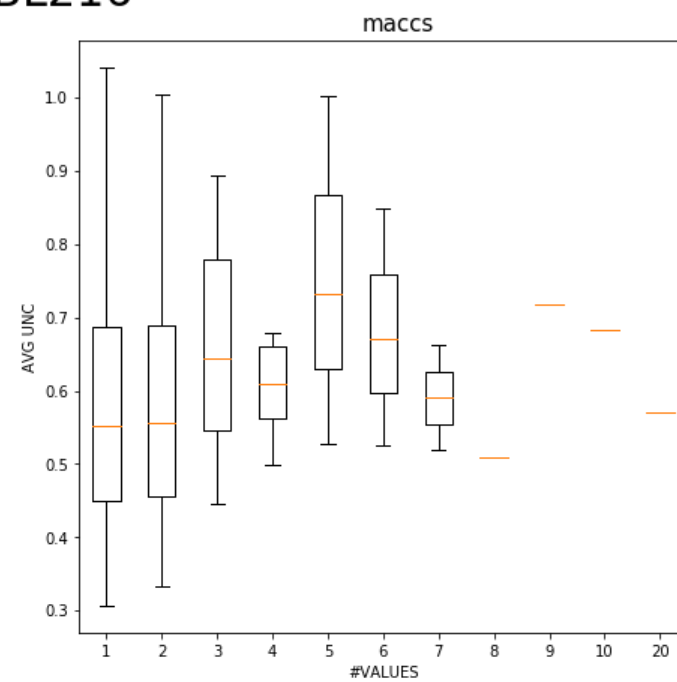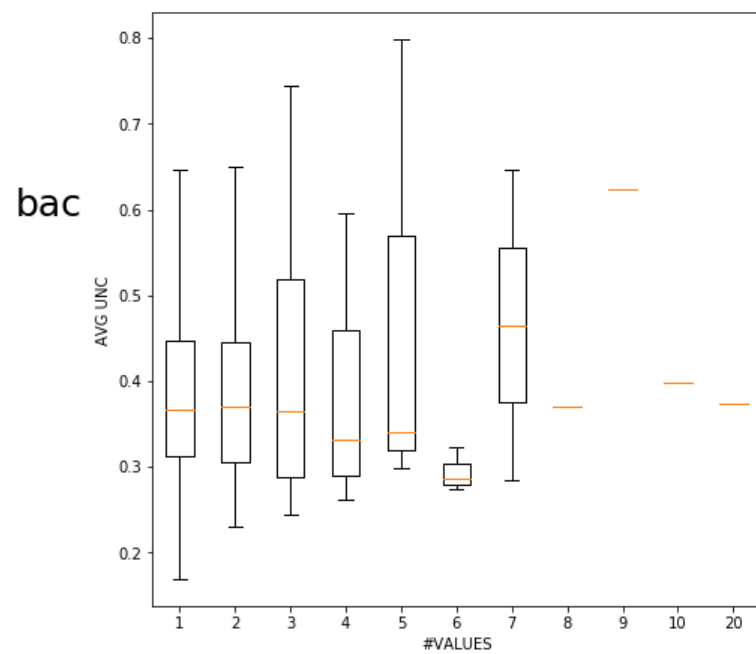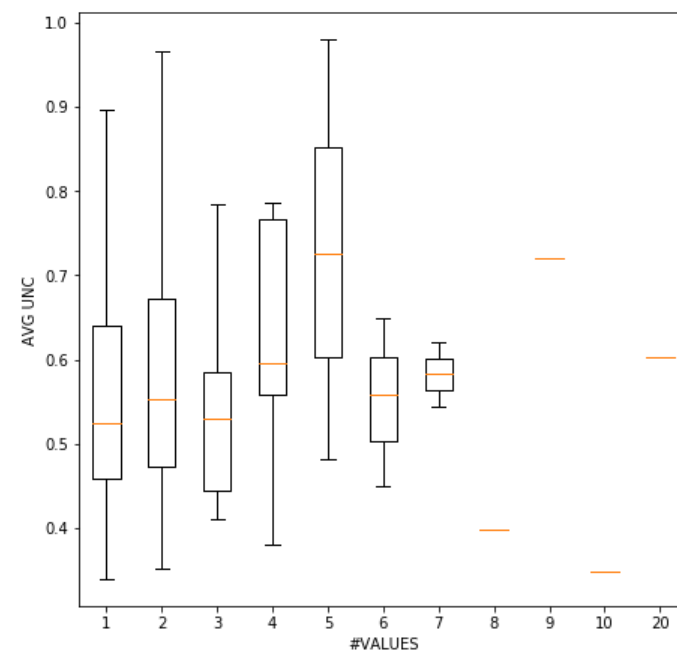

## CHEMBL217

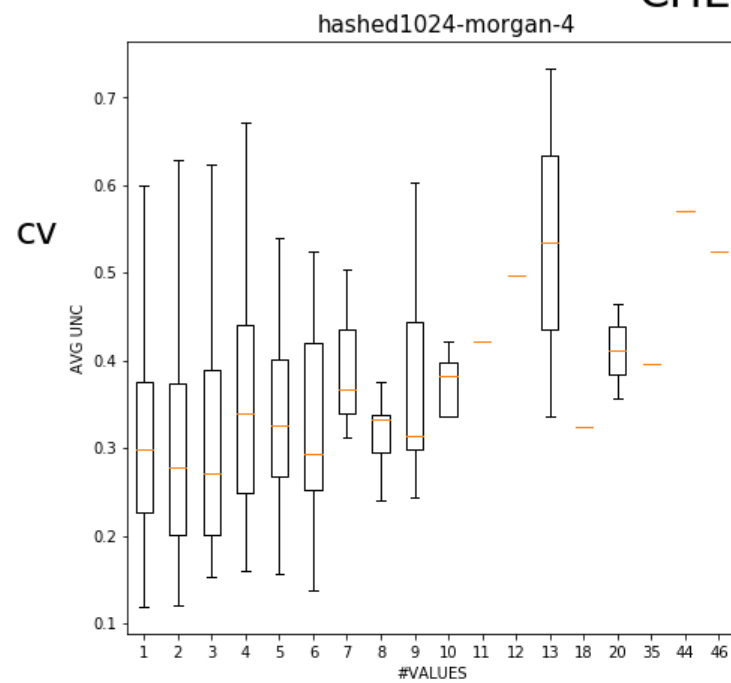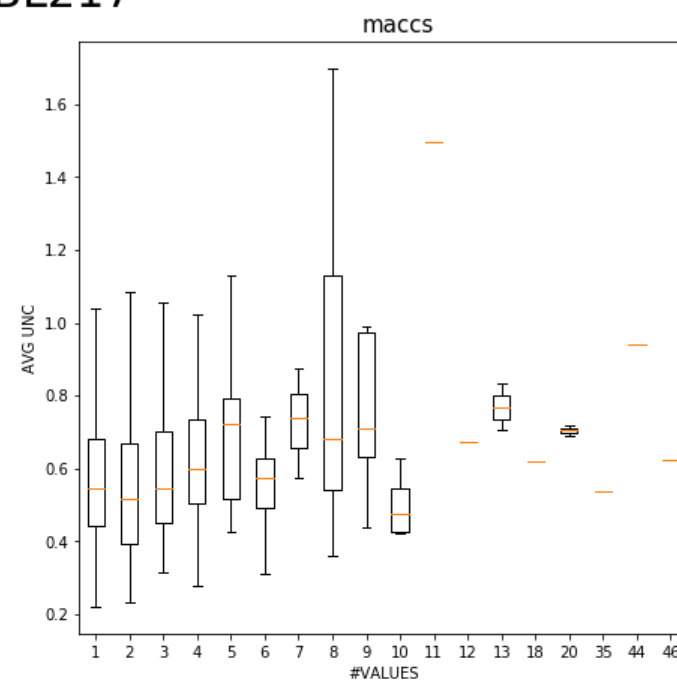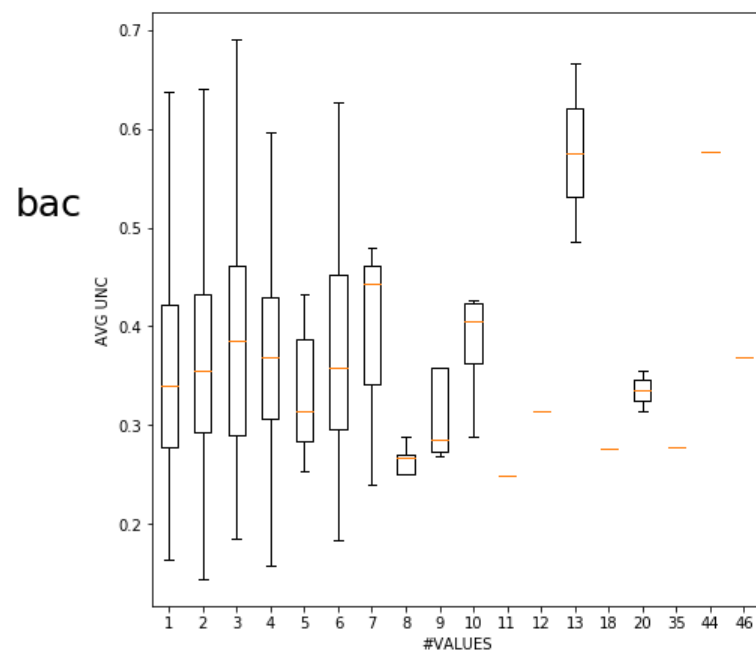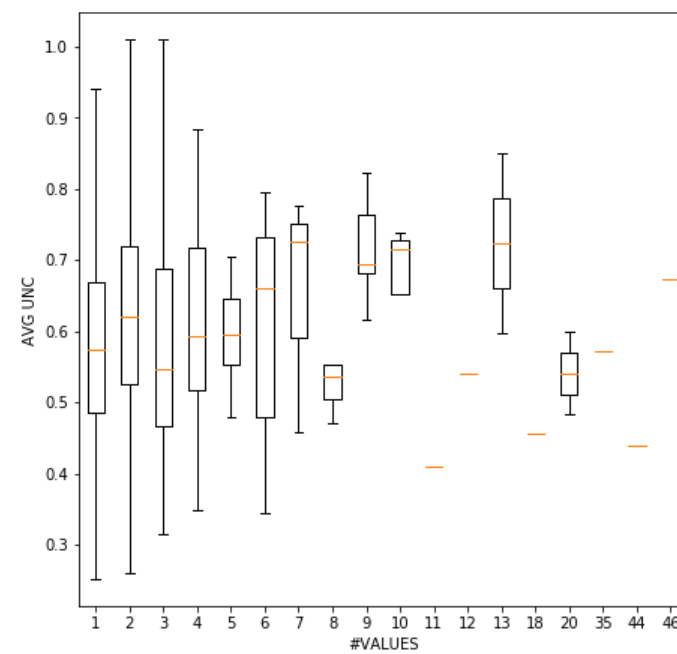

## CHEMBL224

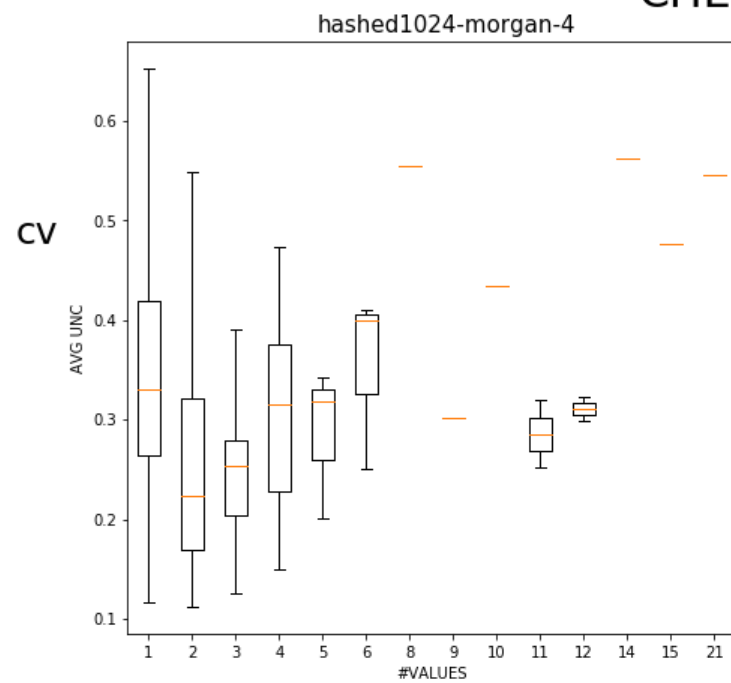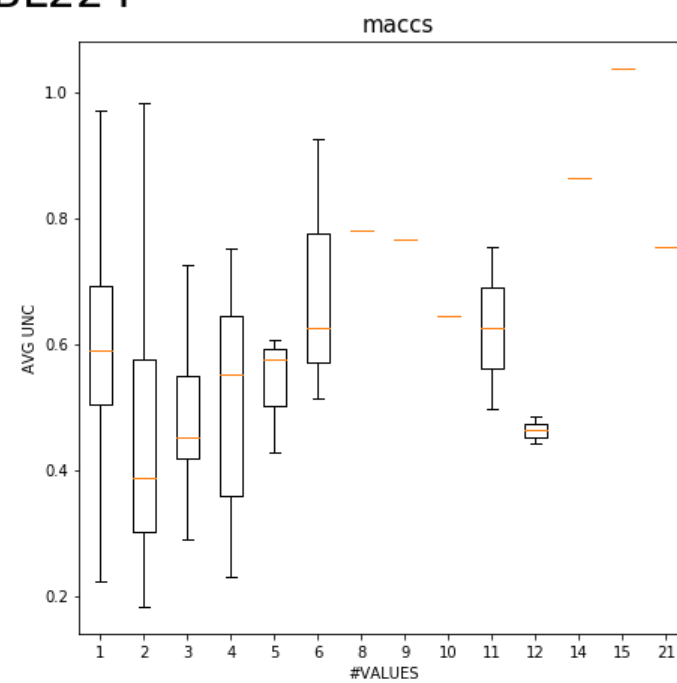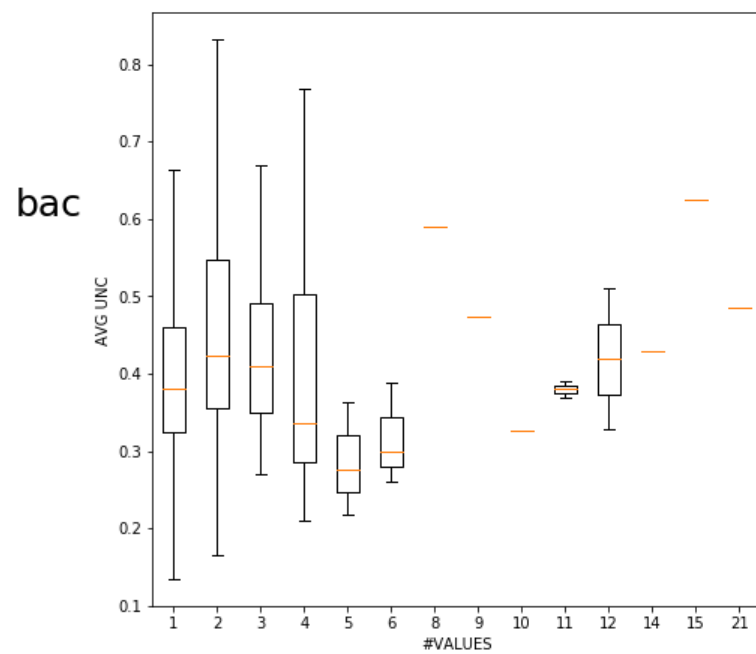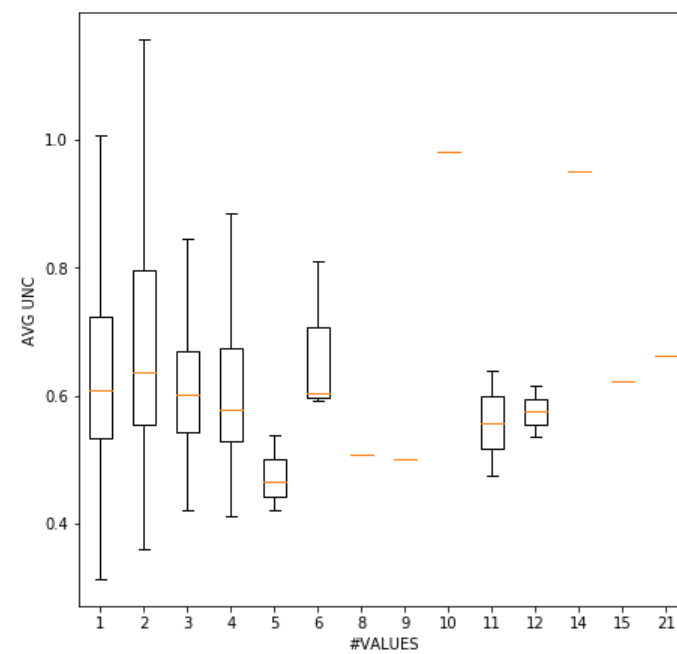

# CHEMBL225

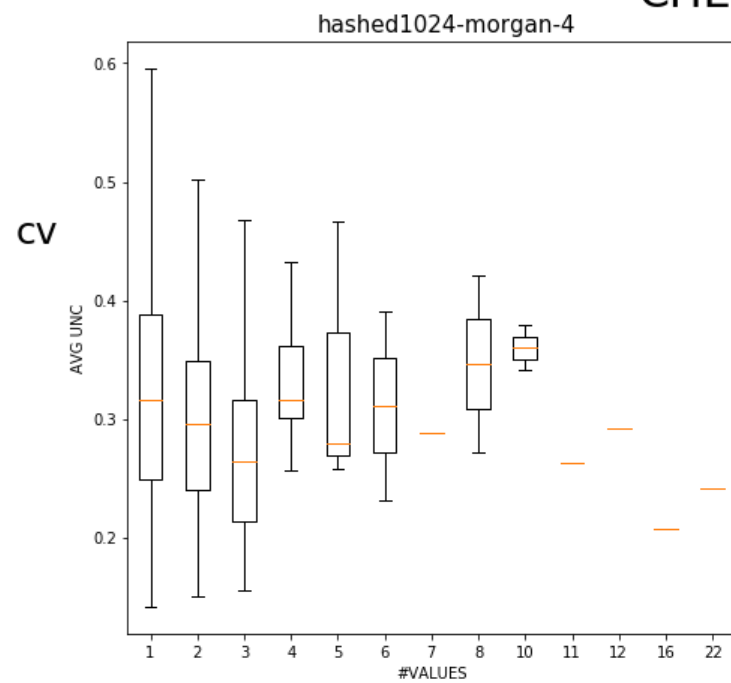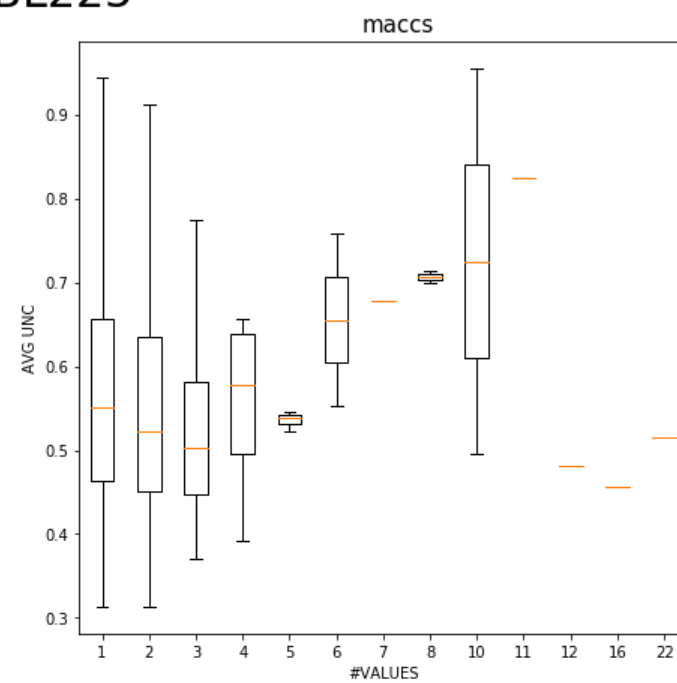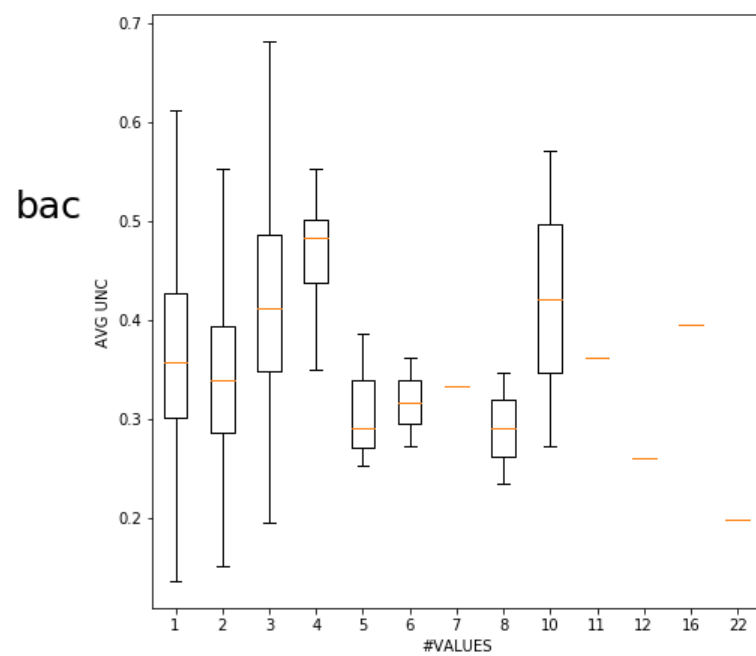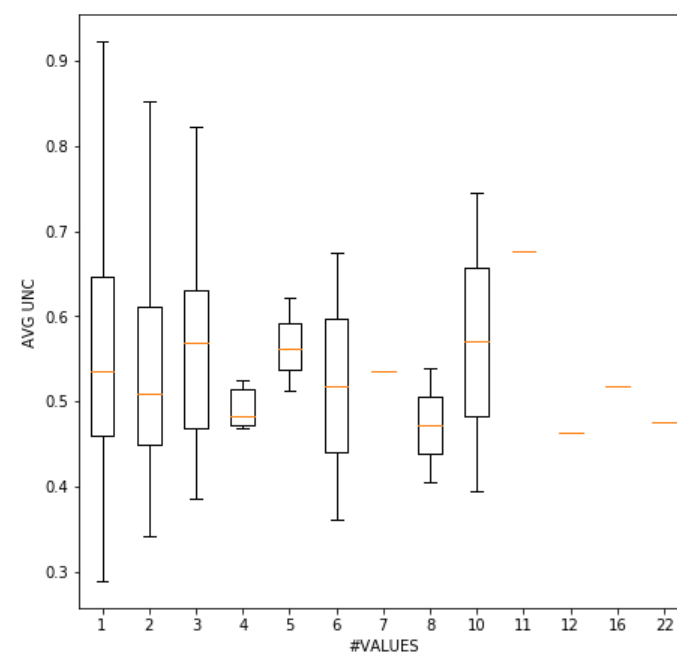

# CHEMBL226

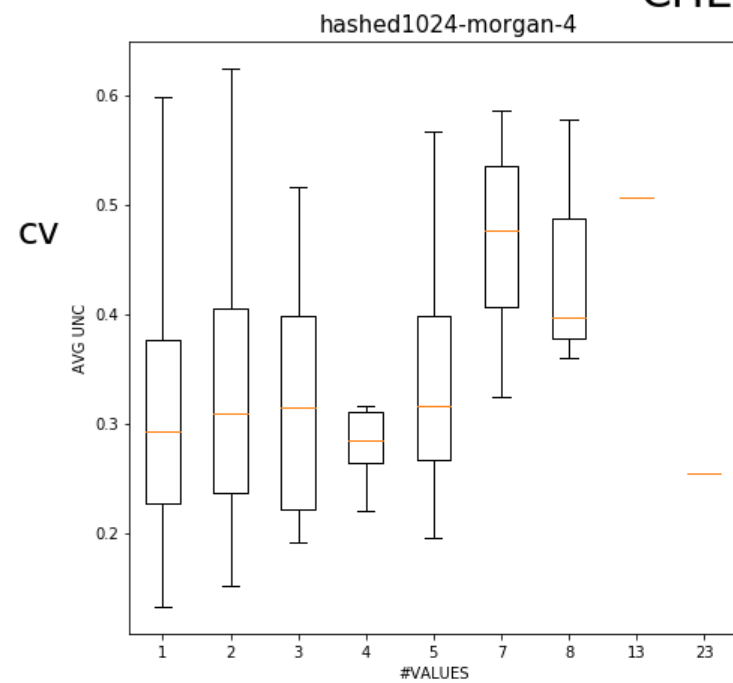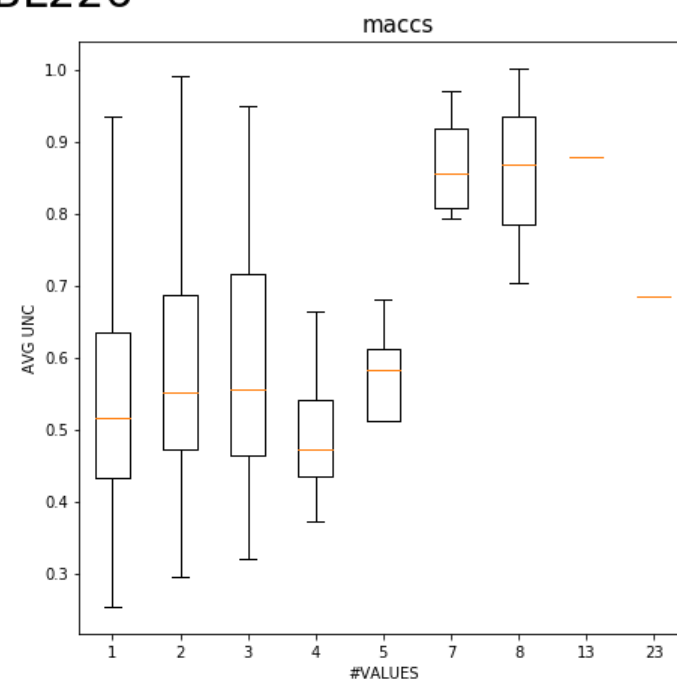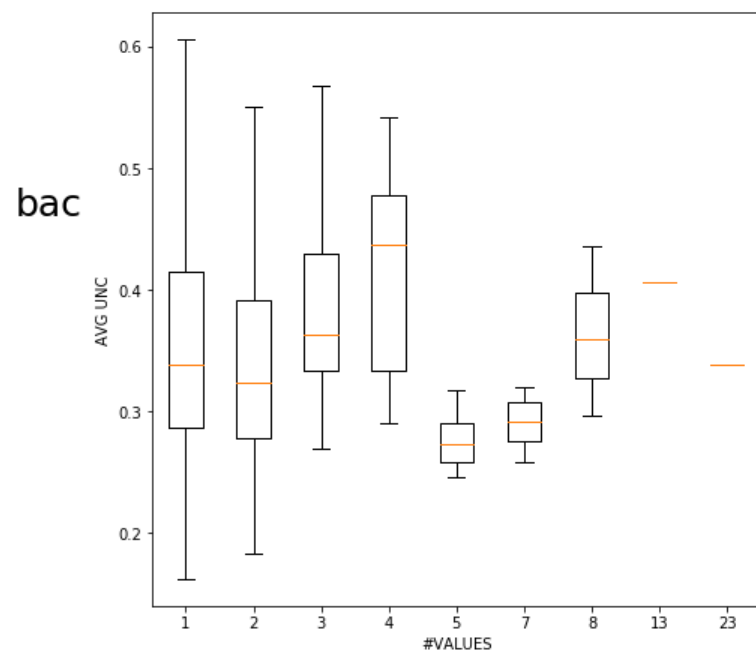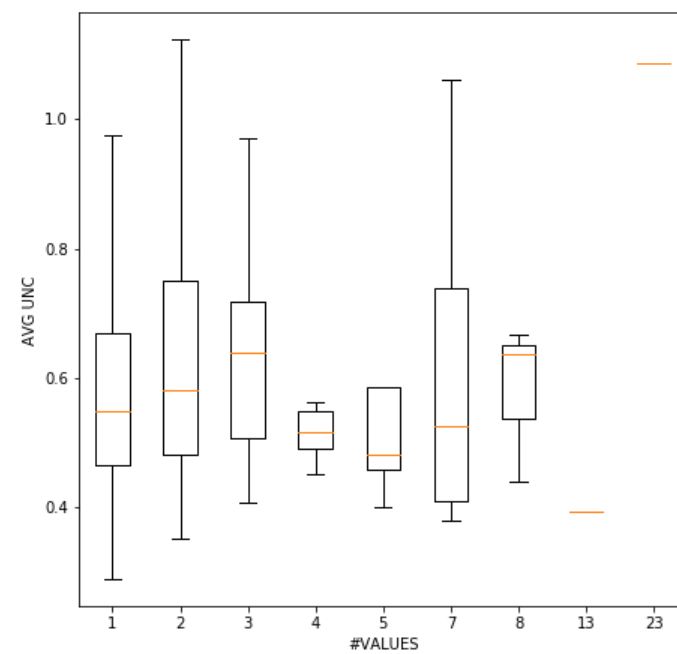

## CHEMBL251

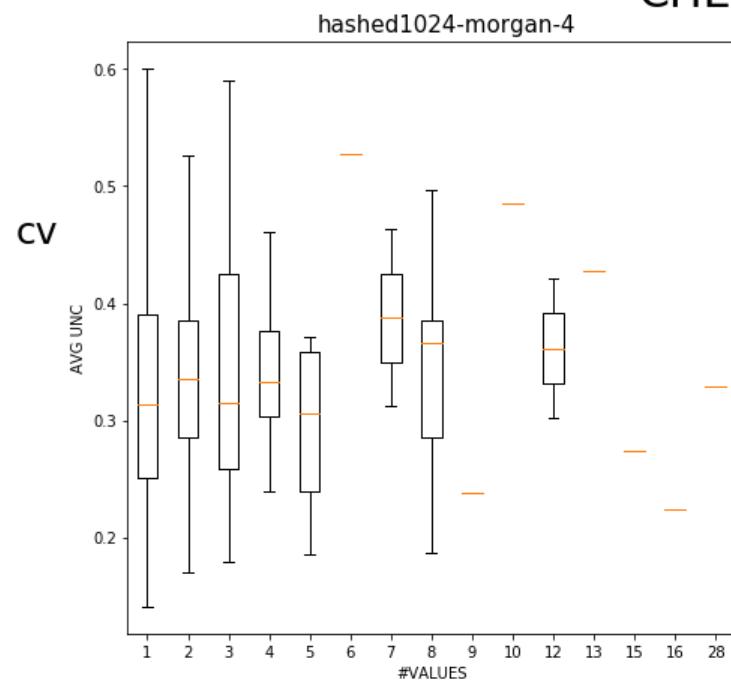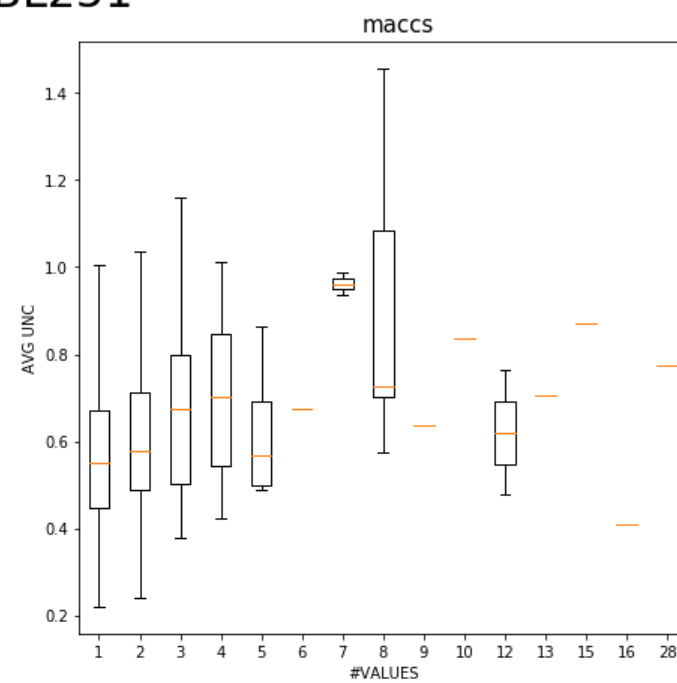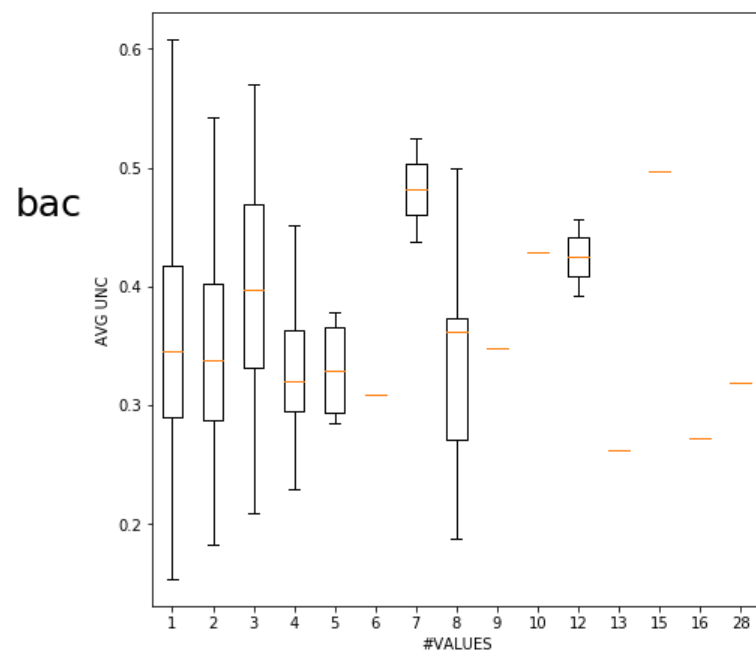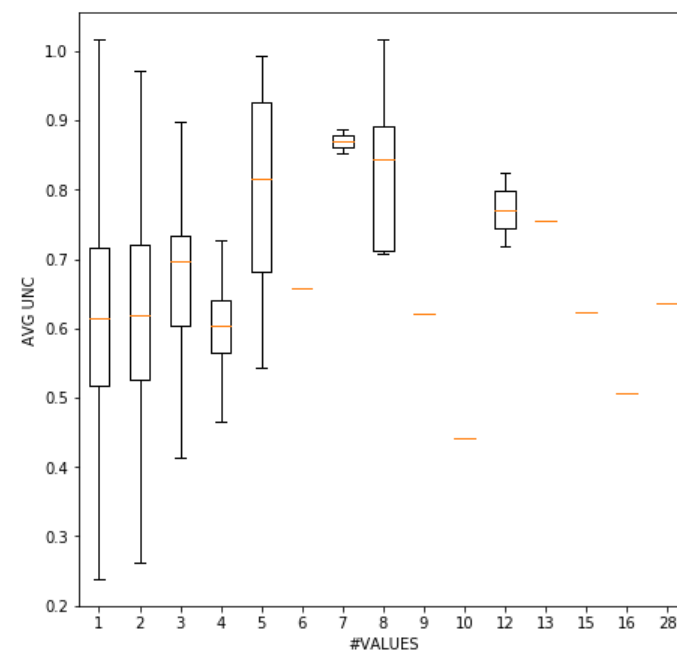

# CHEMBL264

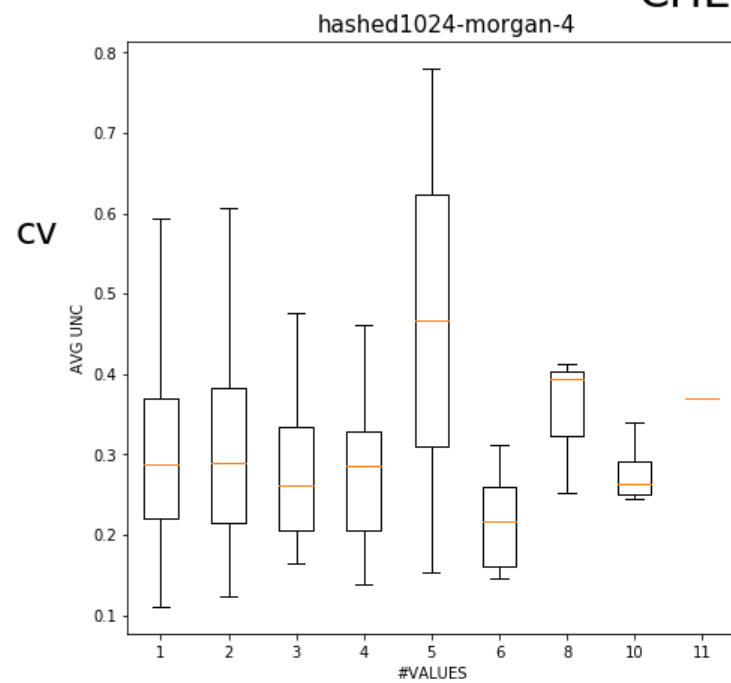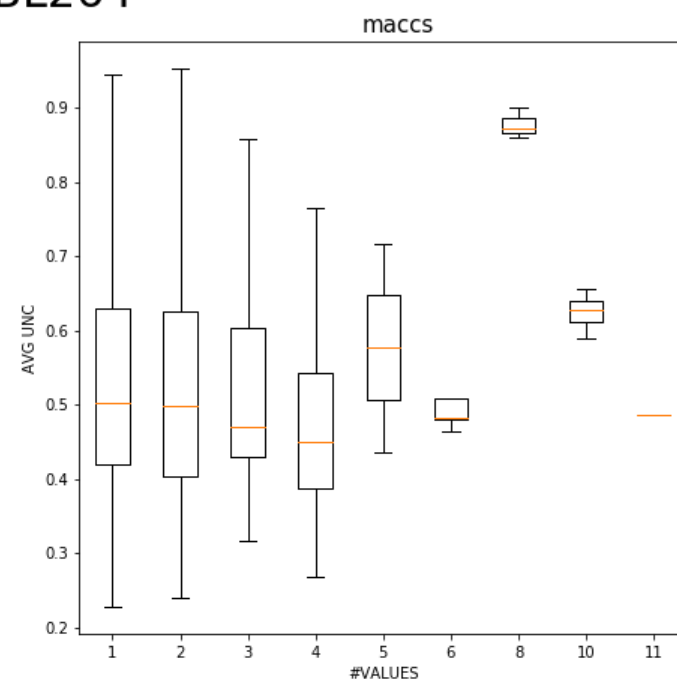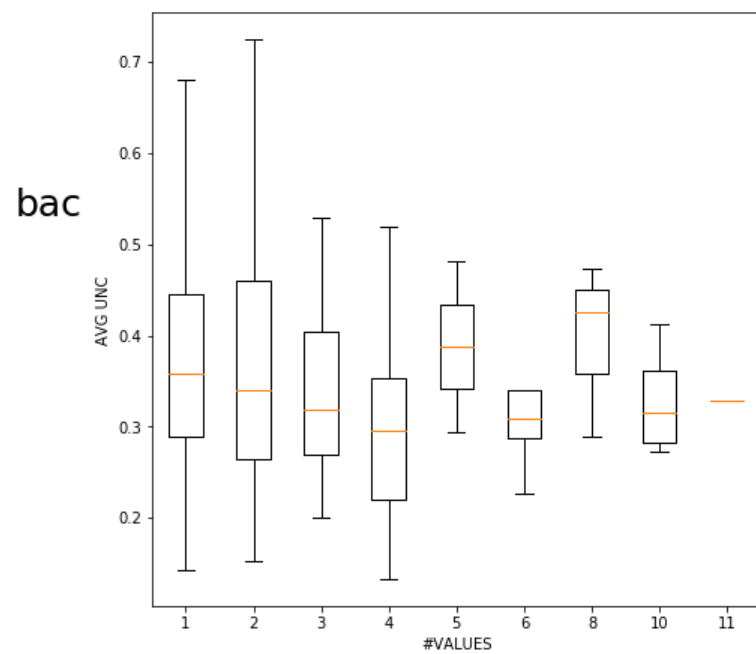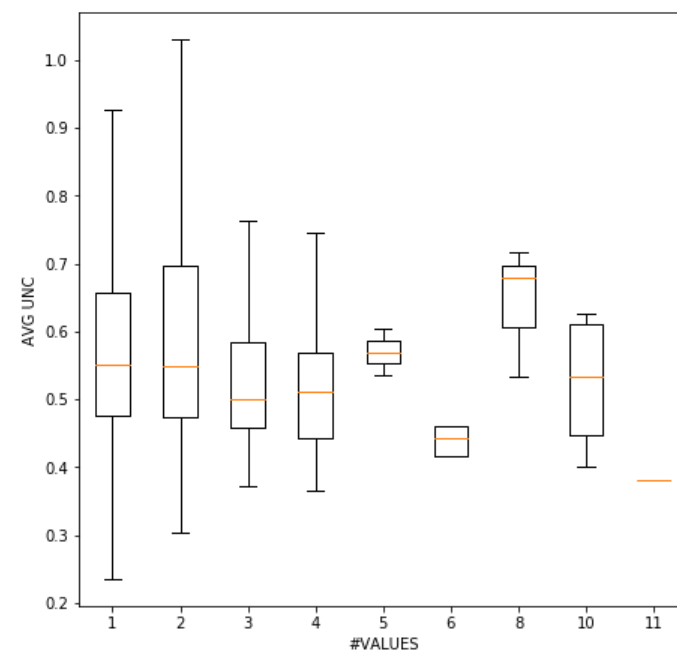

# CHEMBL3155

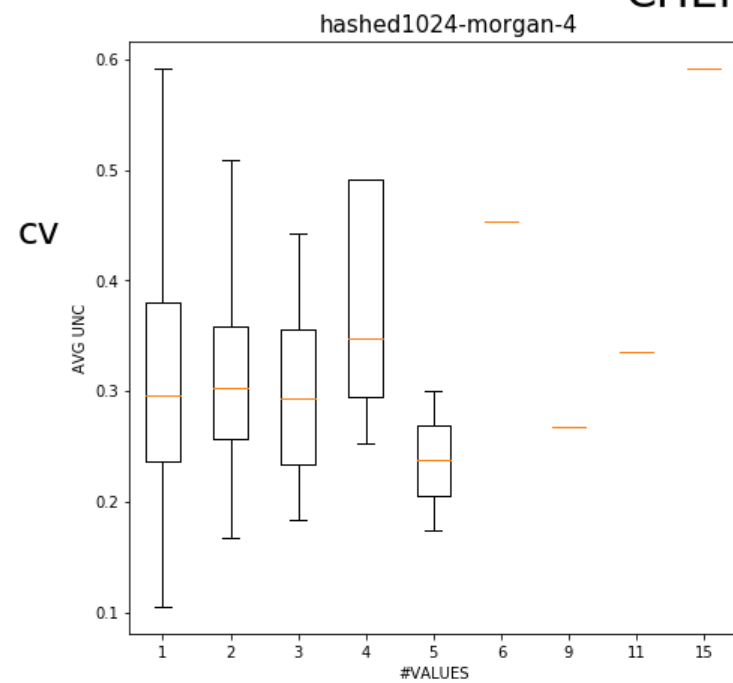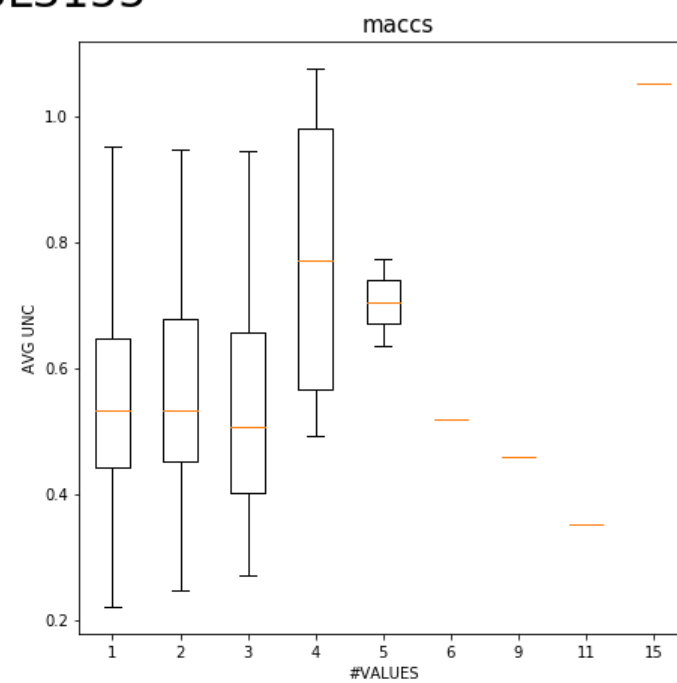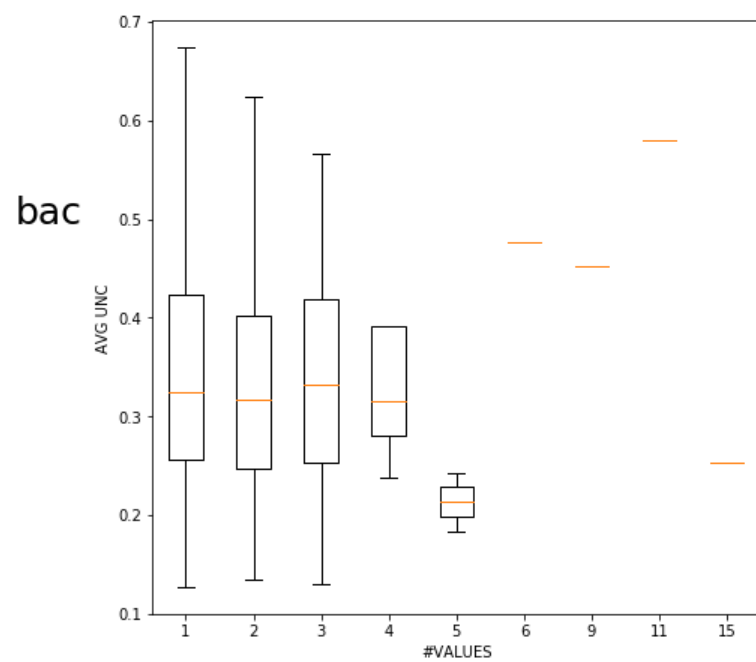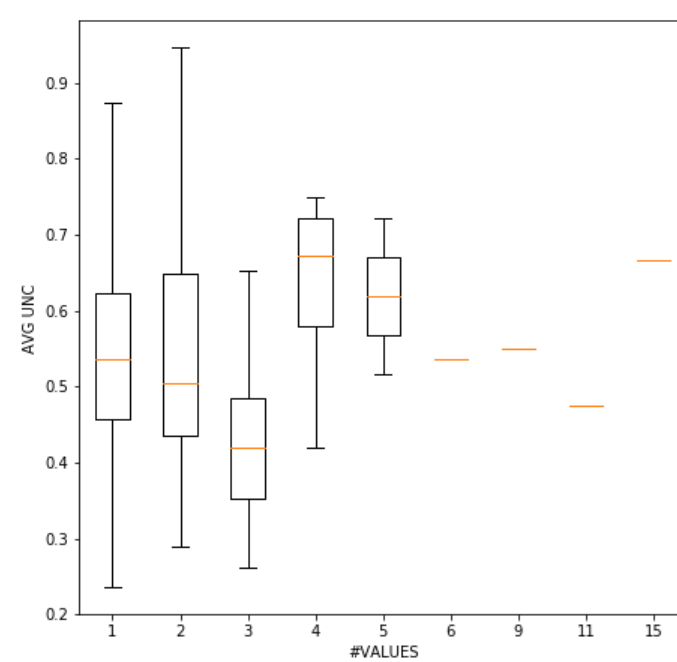

# CHEMBL3371

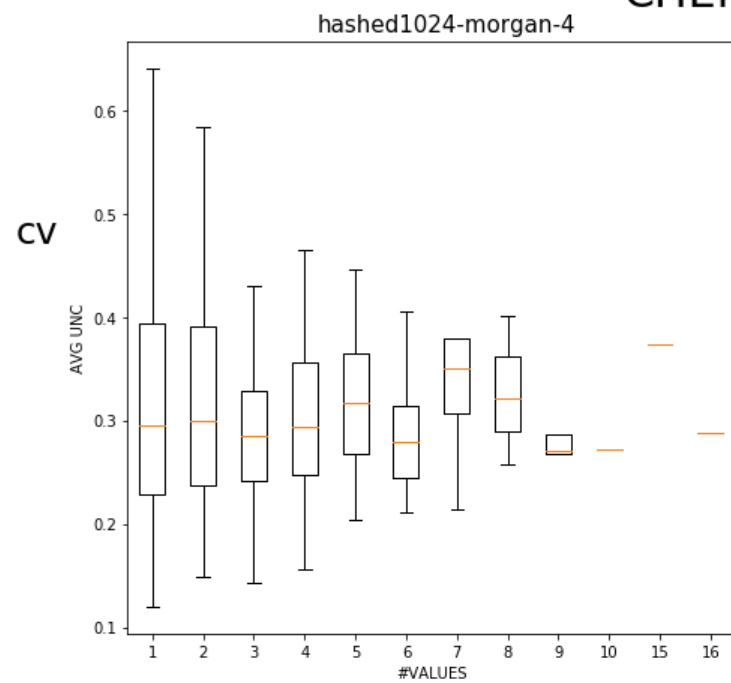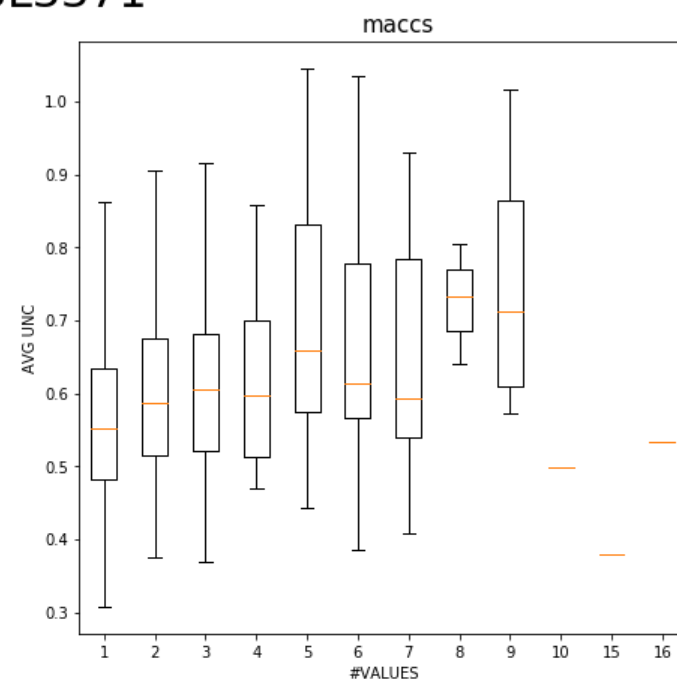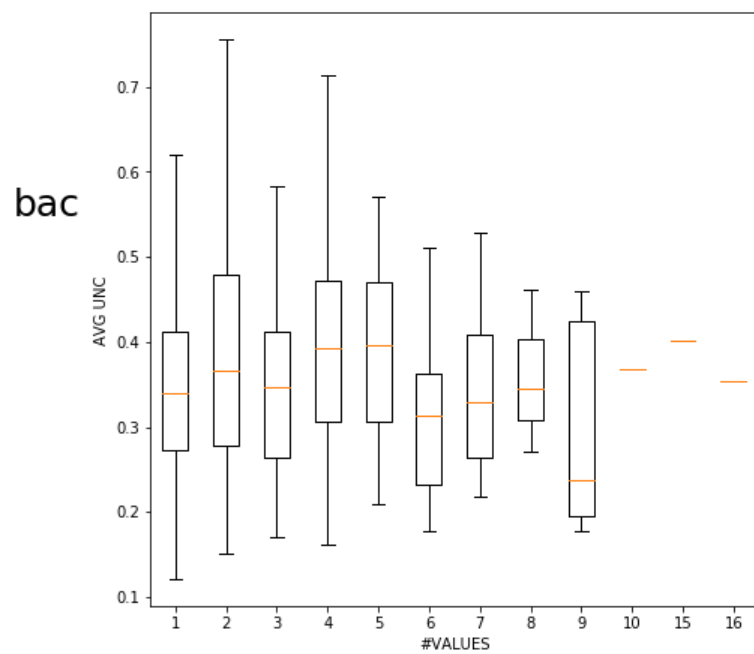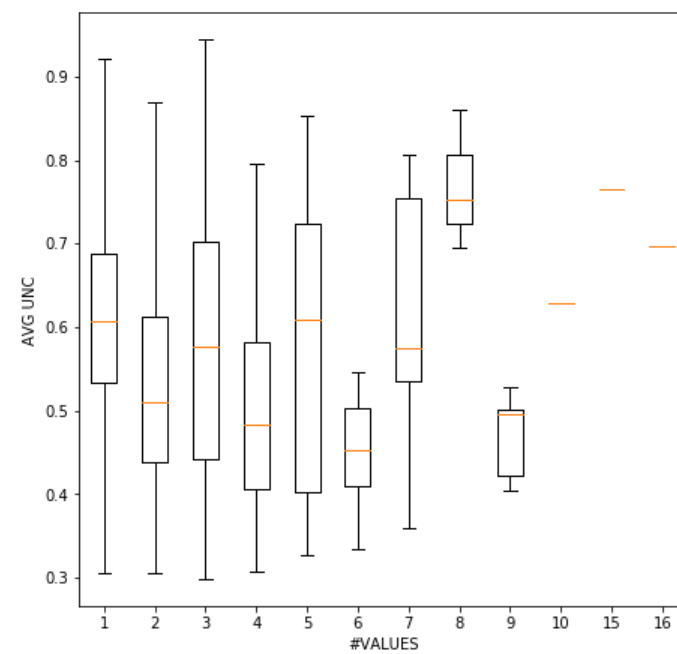

Supplement: Supplementary file 1 [file molecules-25-01452-s001.zip › Supp_Info_for_submission/FileS5.pdf]

# CHEMBL214

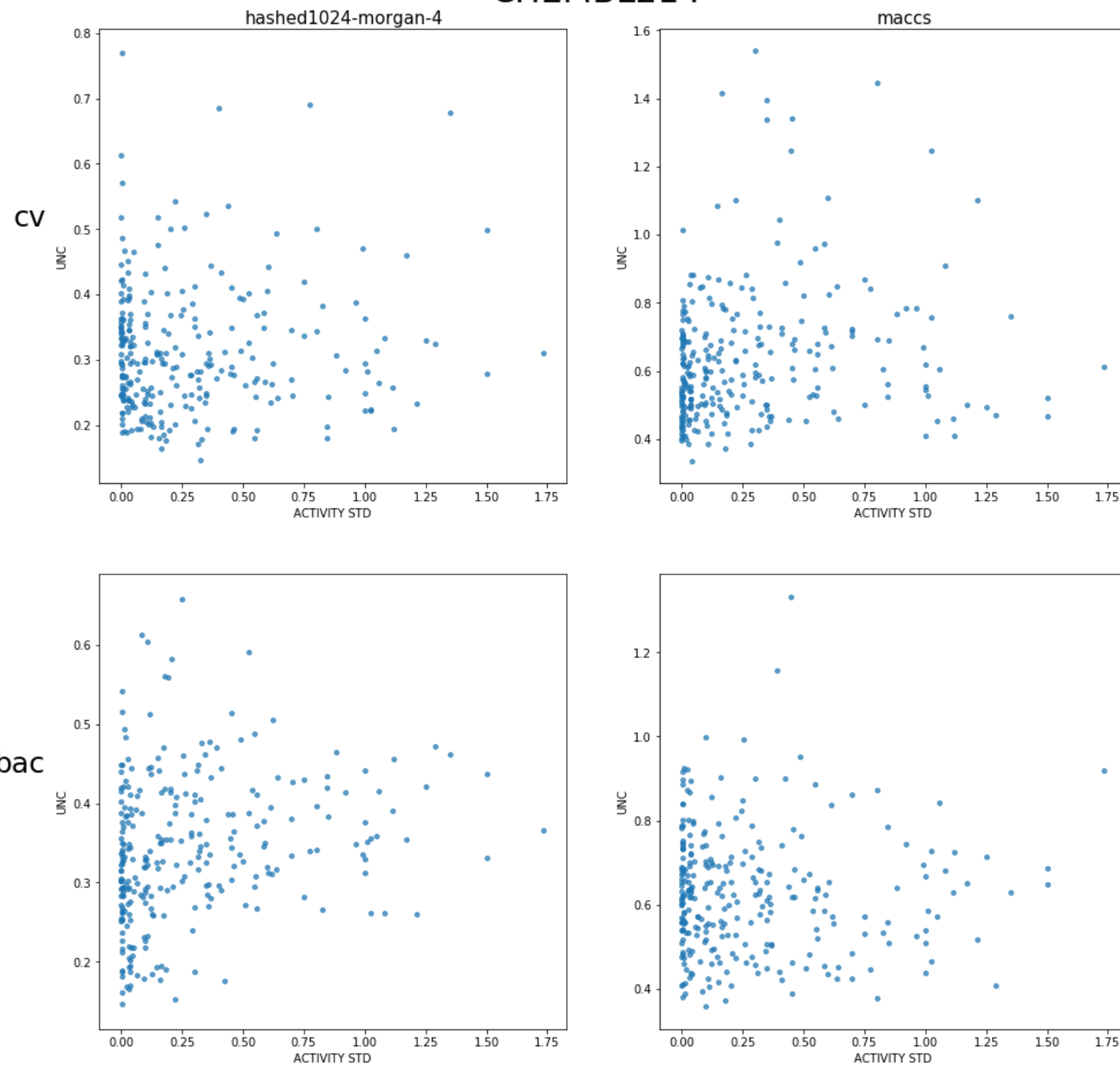

# CHEMBL216

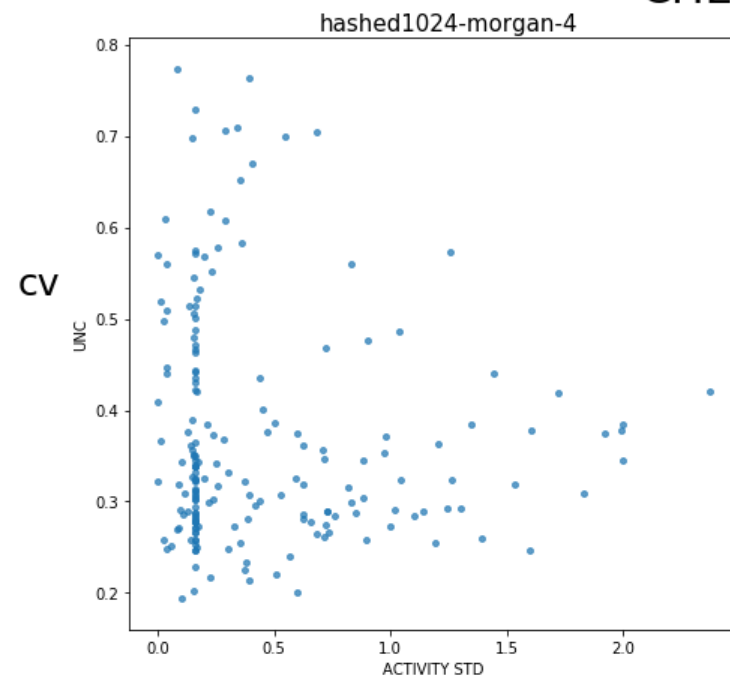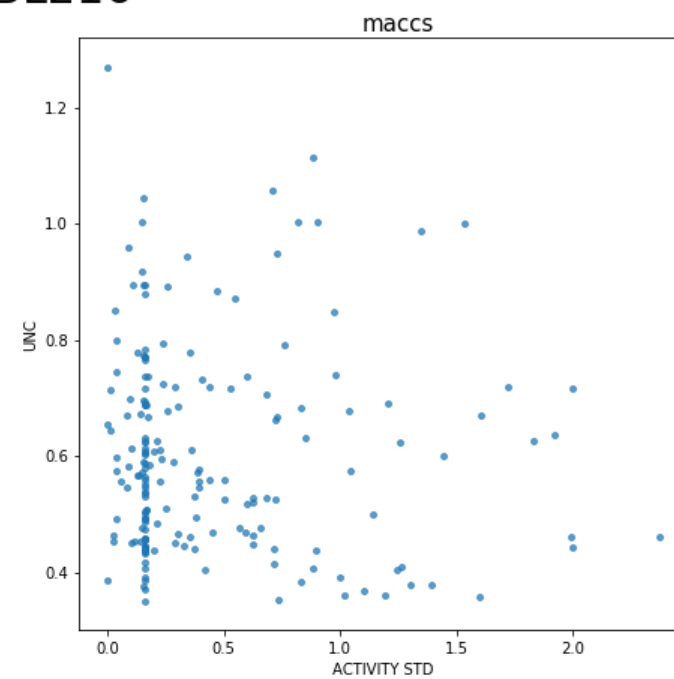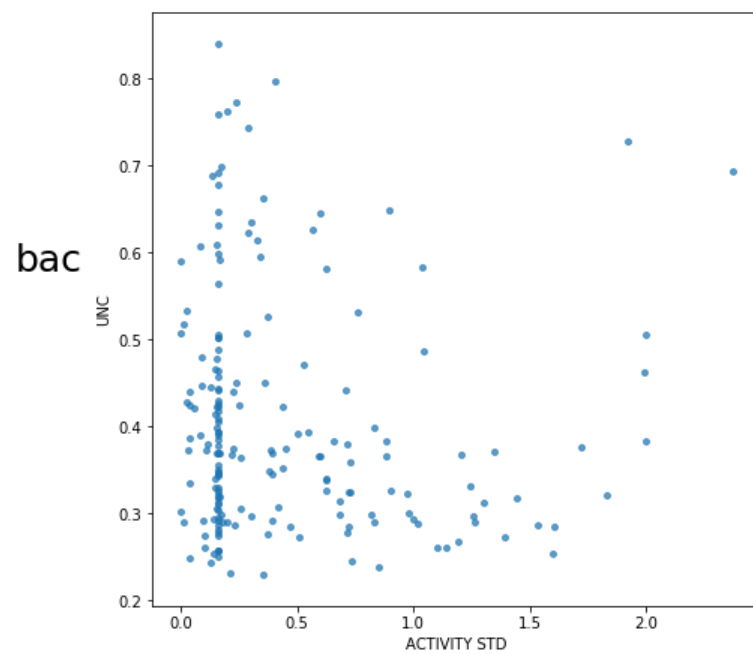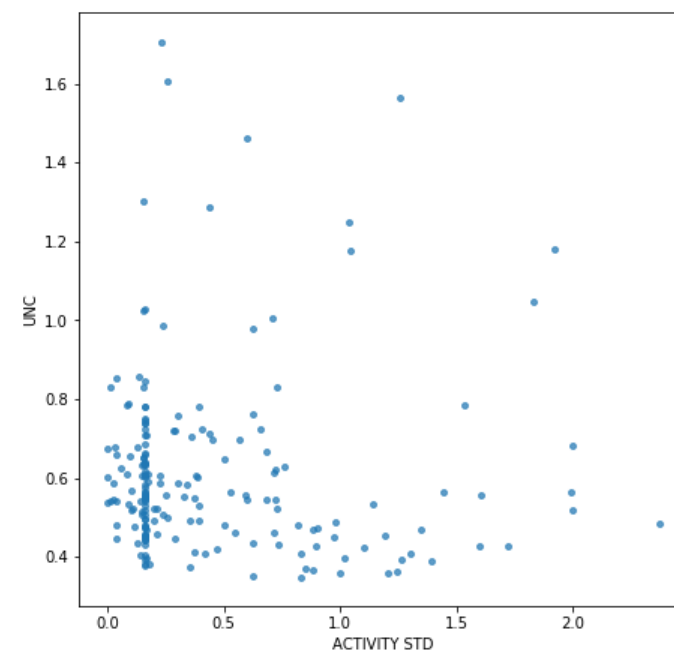

# CHEMBL217

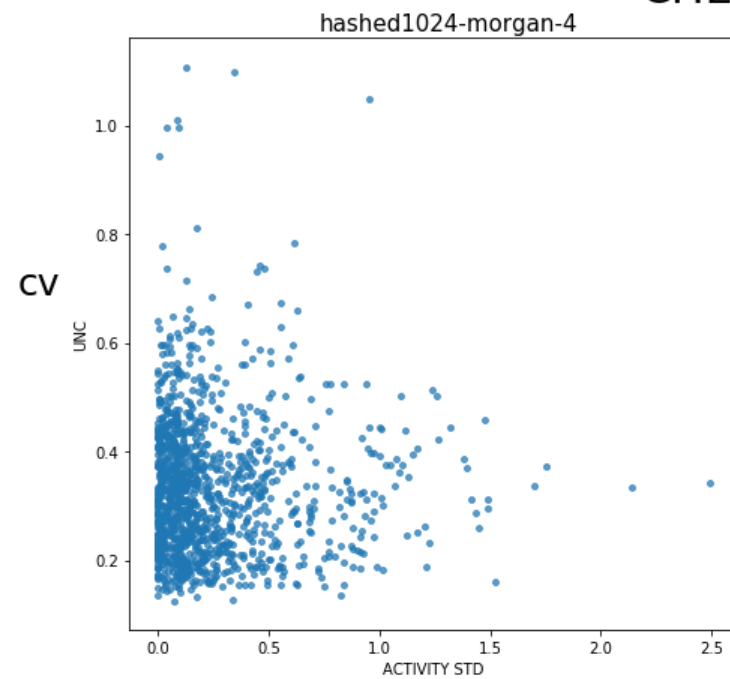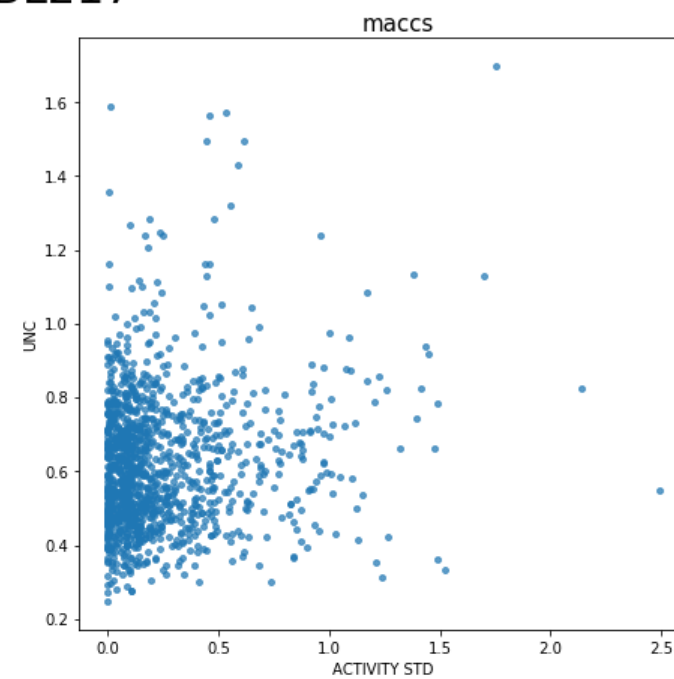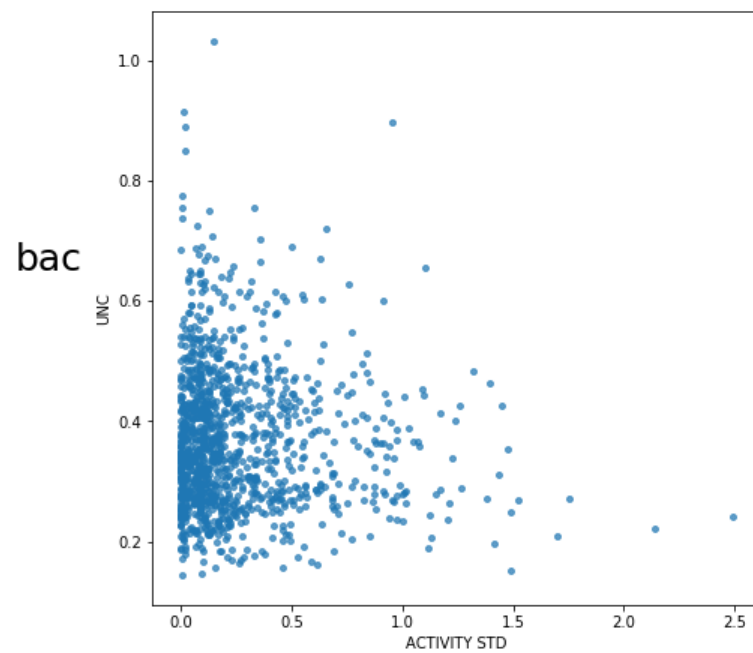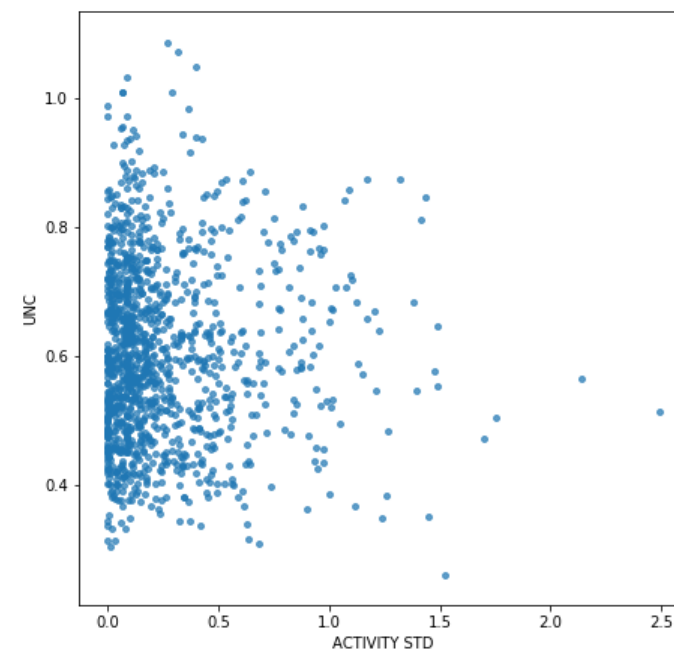

# CHEMBL224

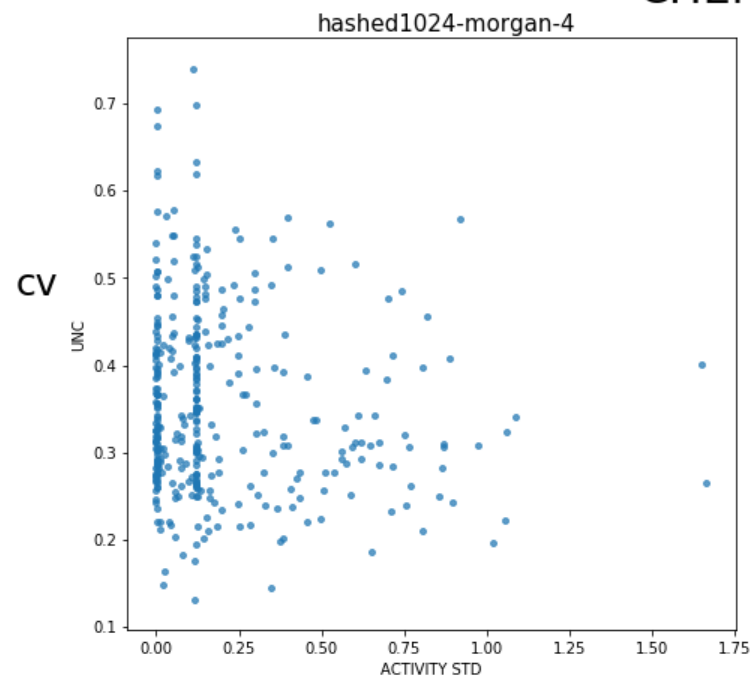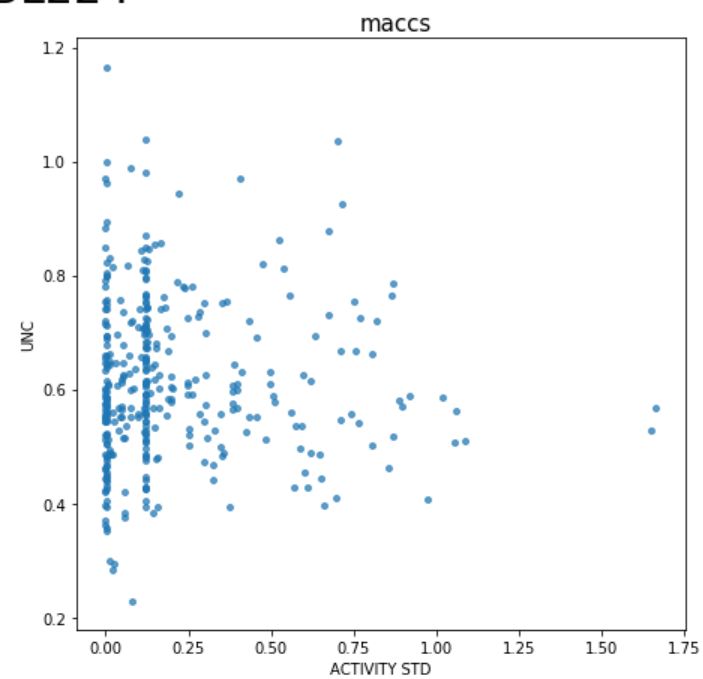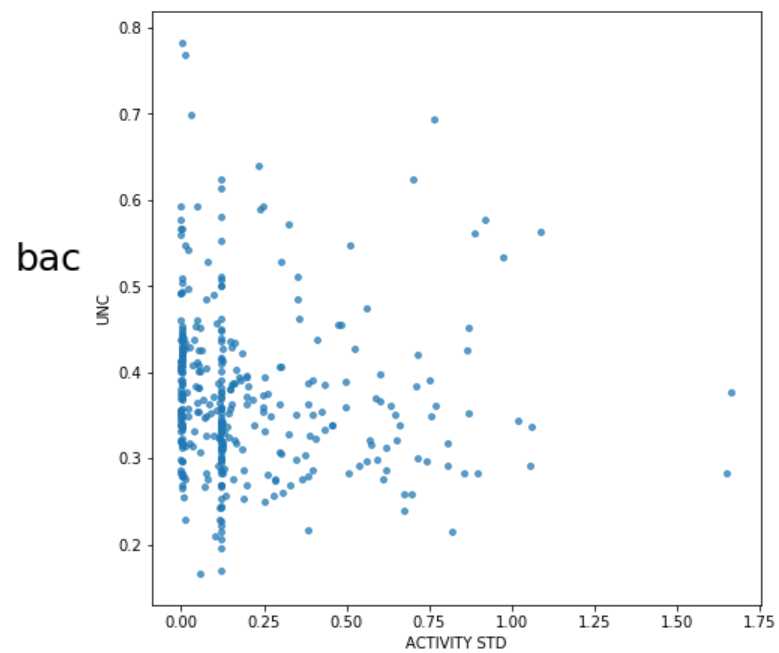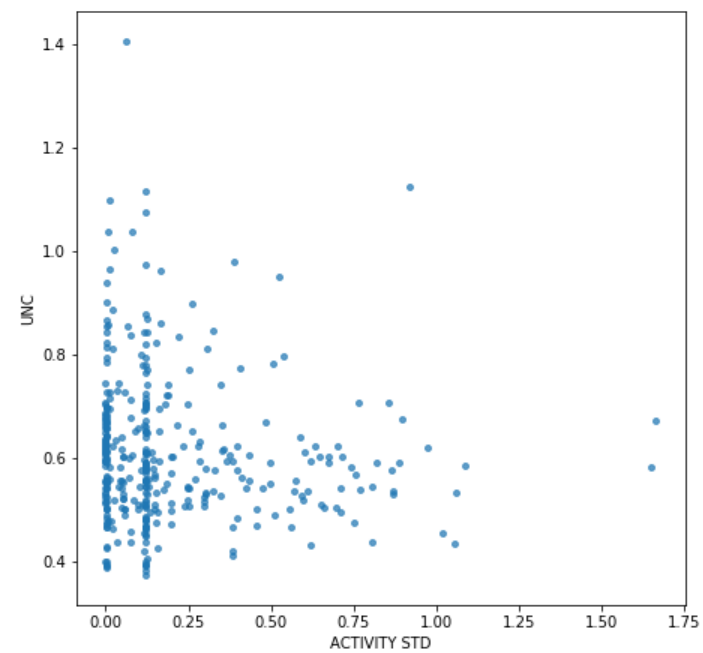

# CHEMBL225

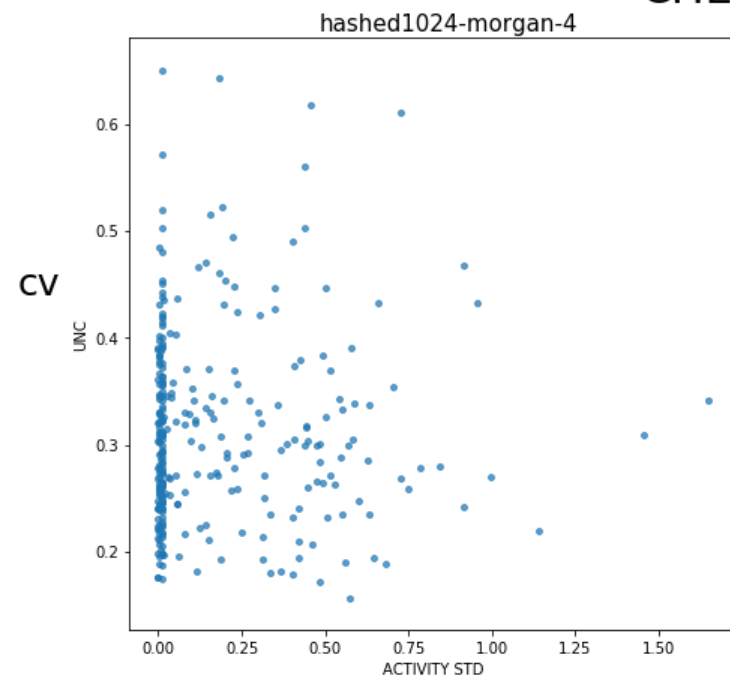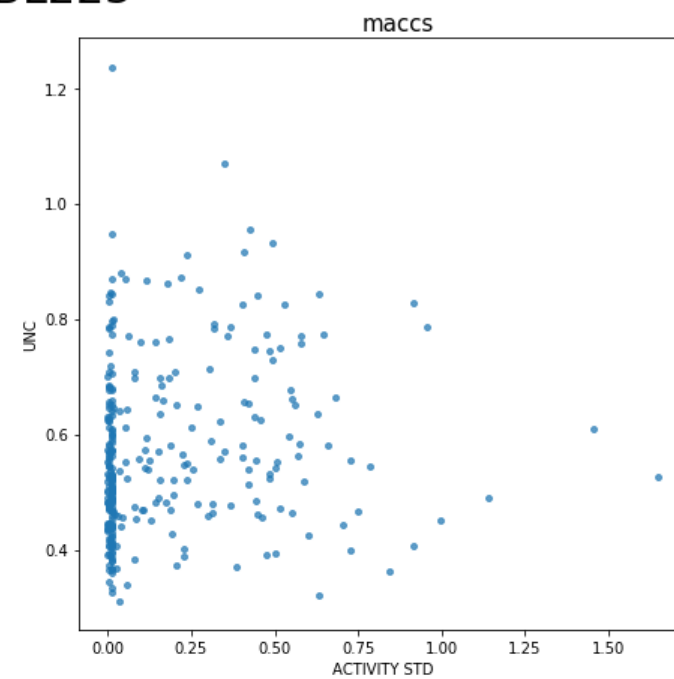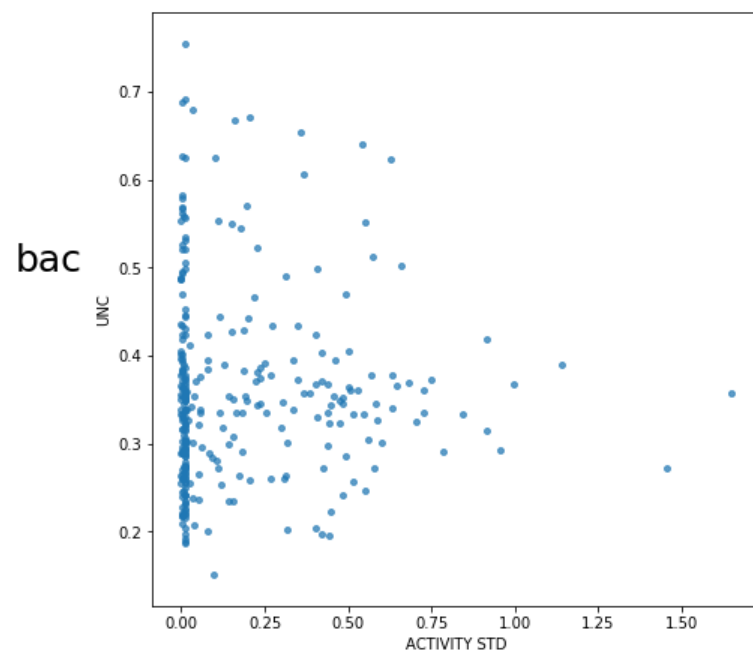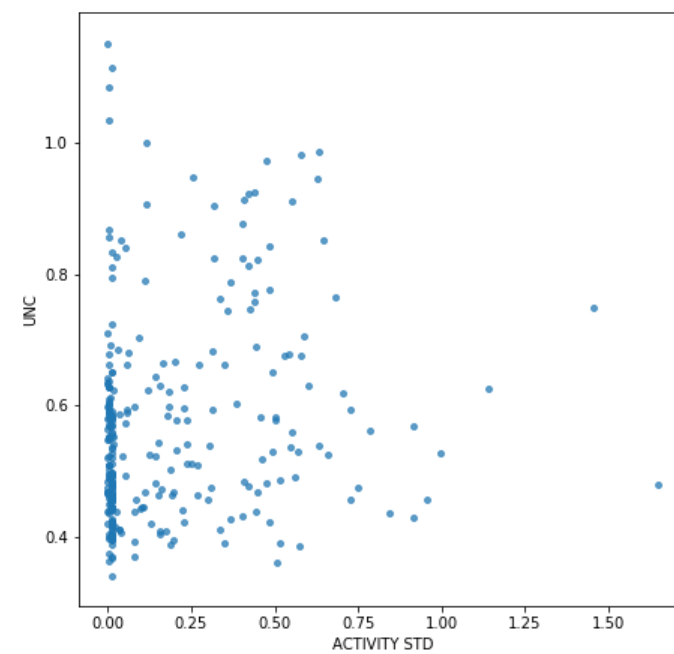

# CHEMBL226

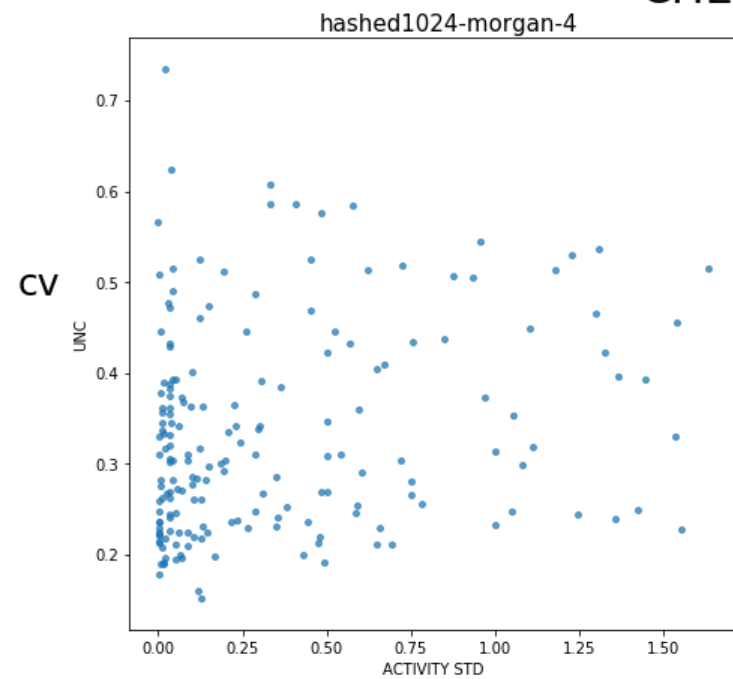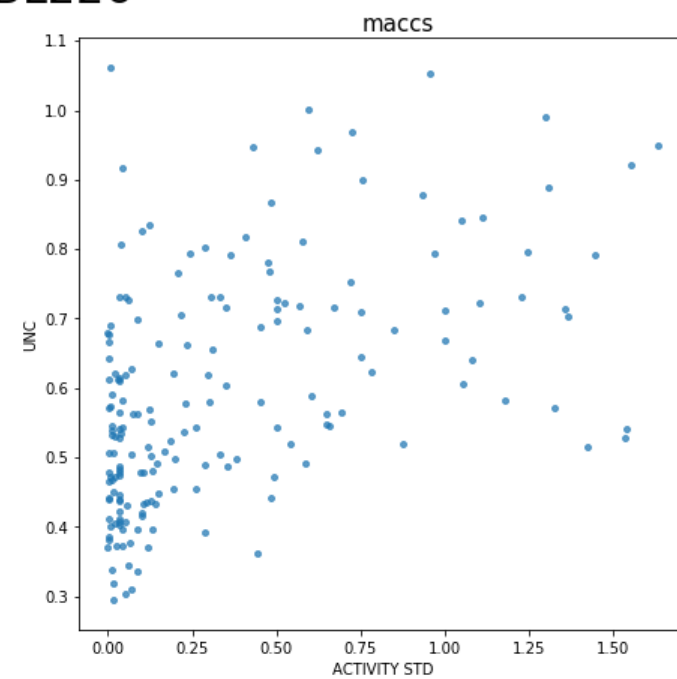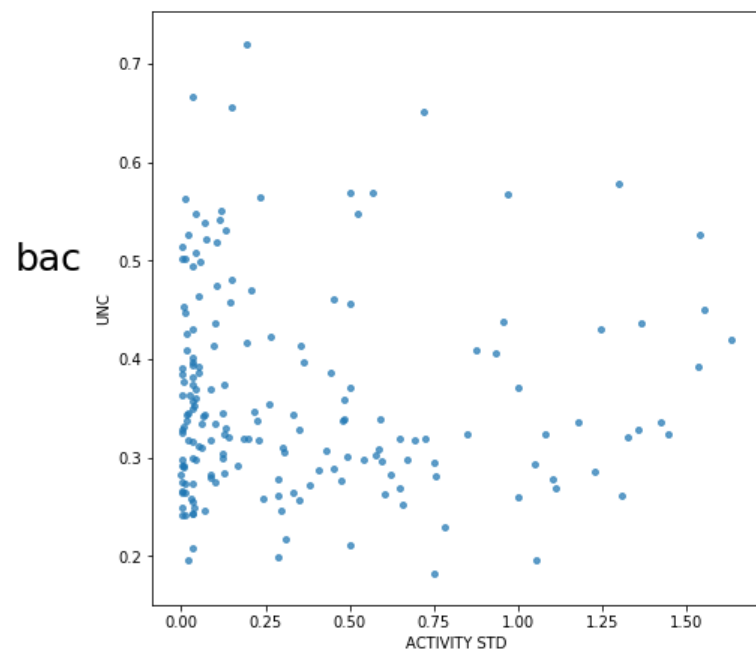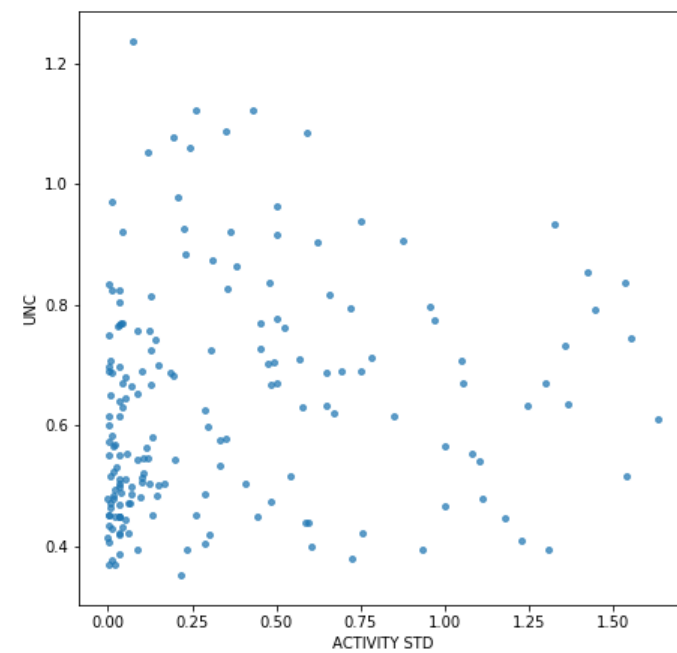

# CHEMBL251

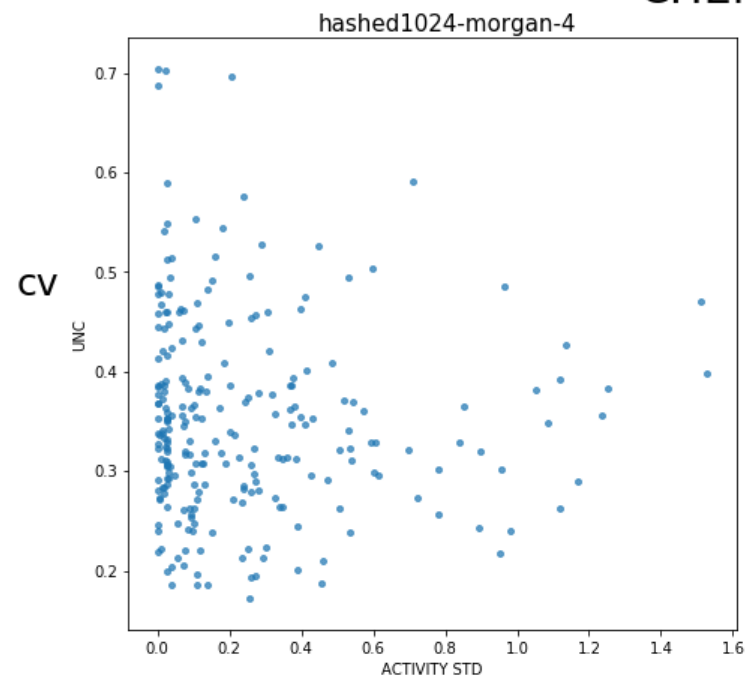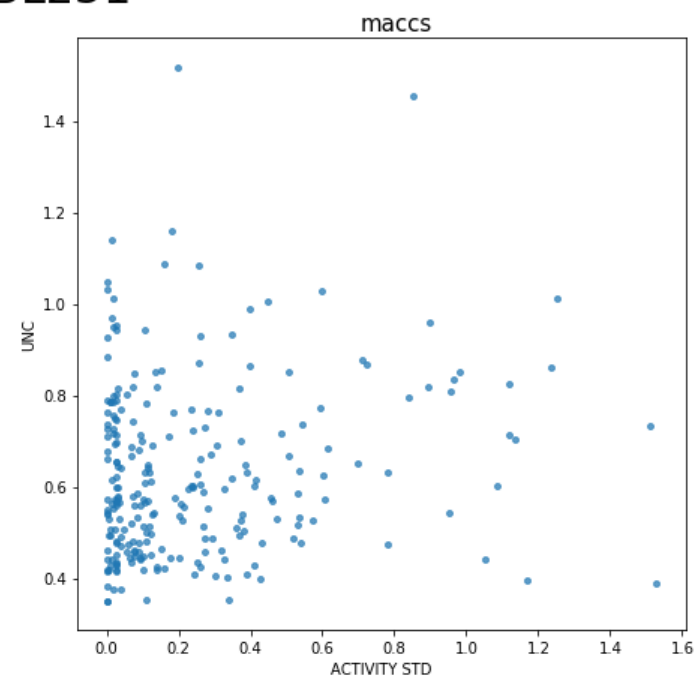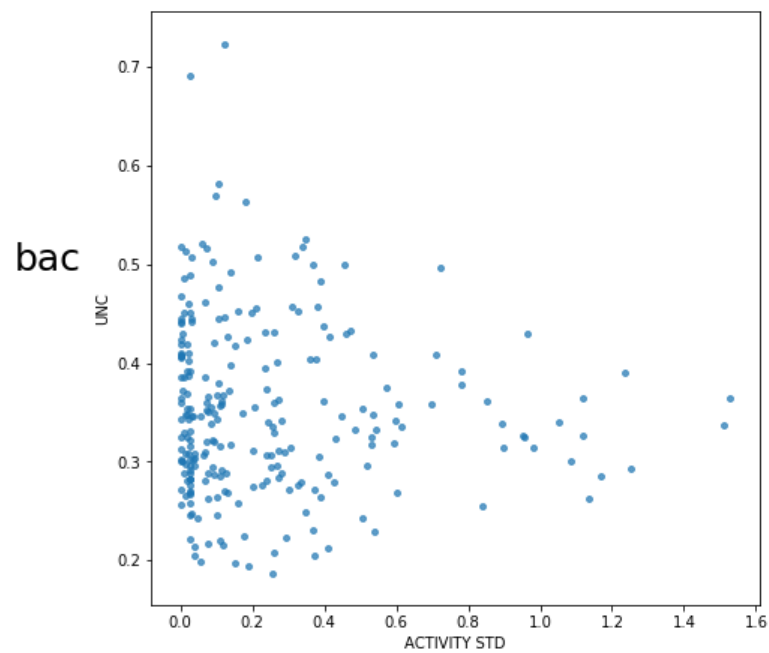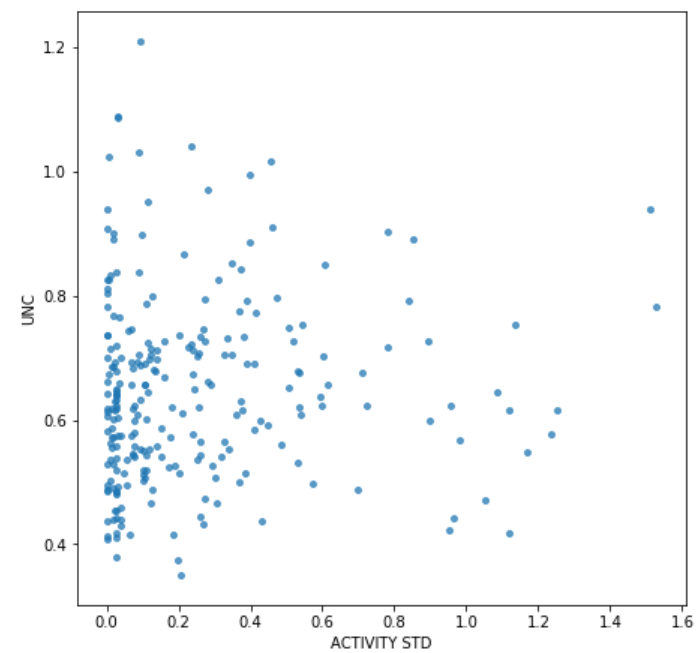

# CHEMBL264

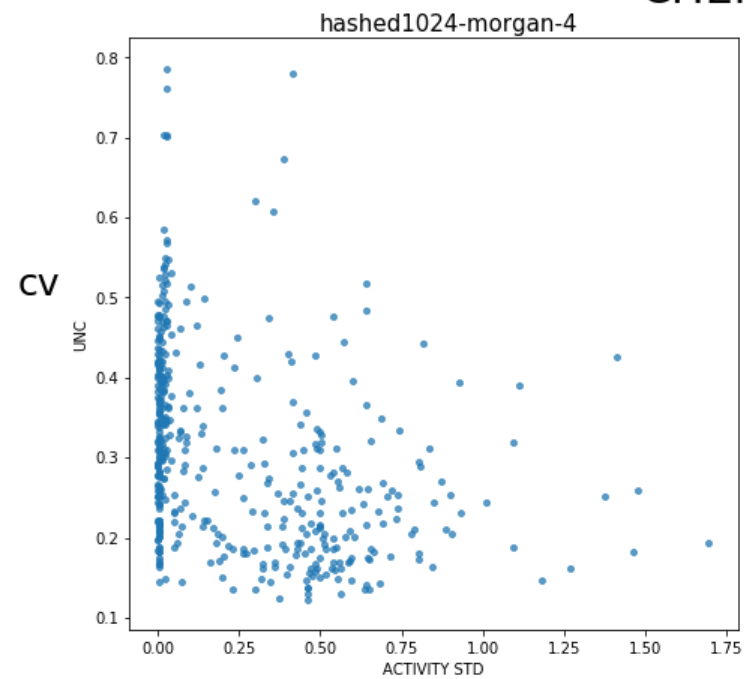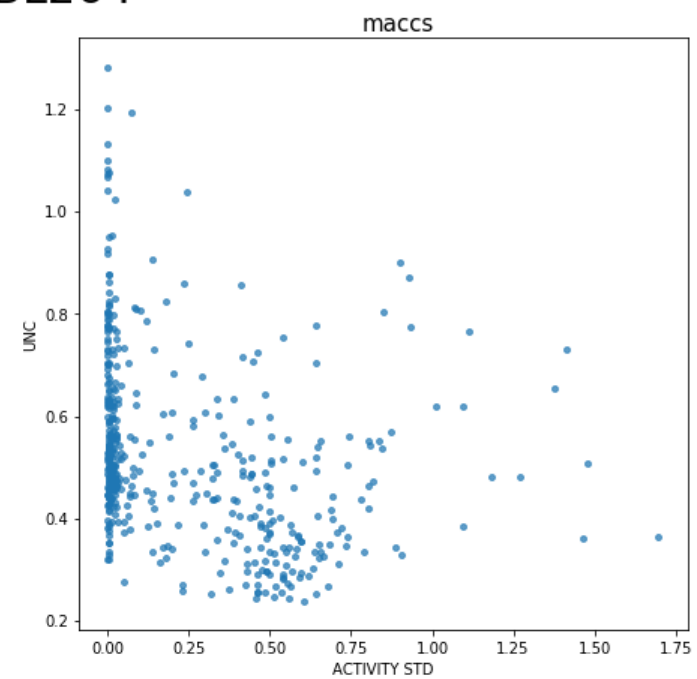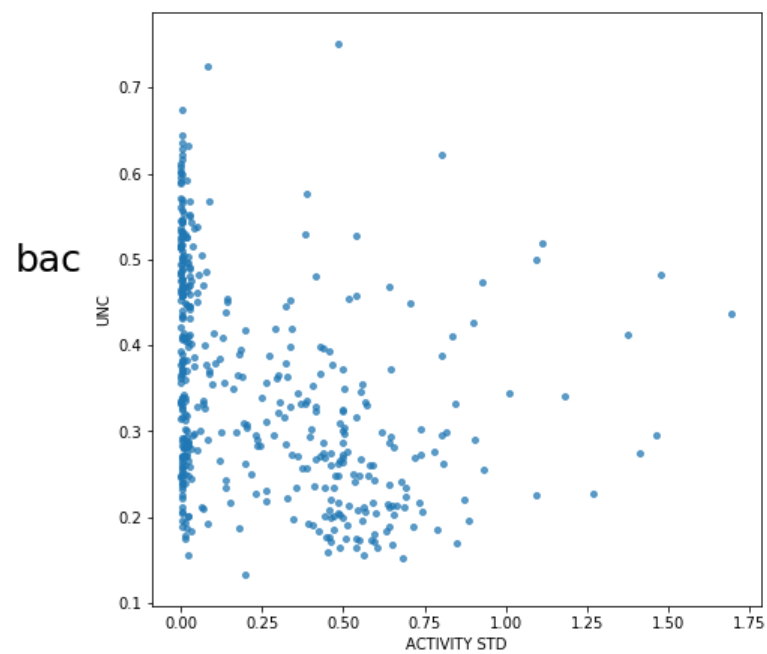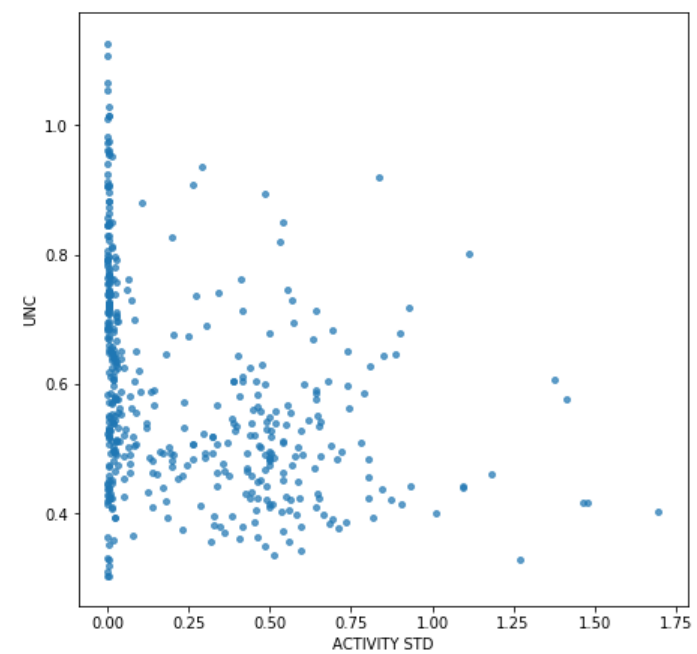

# CHEMBL3155

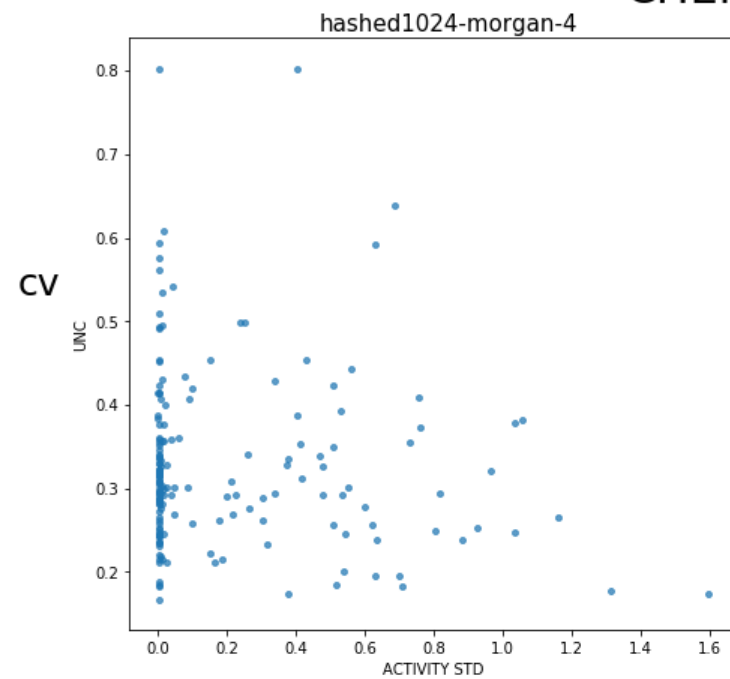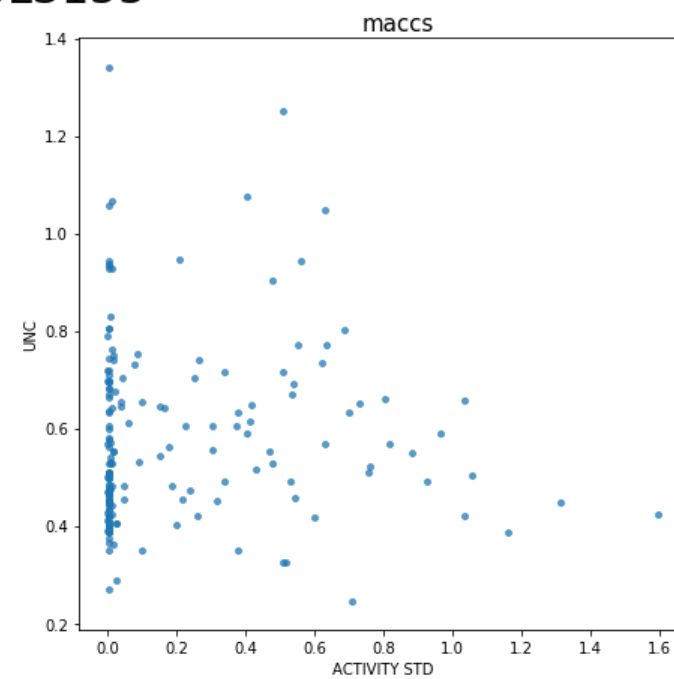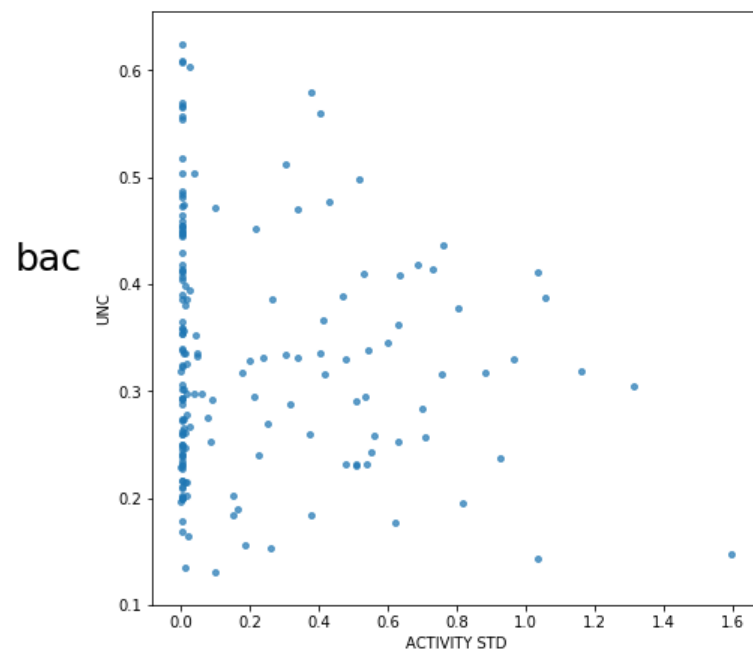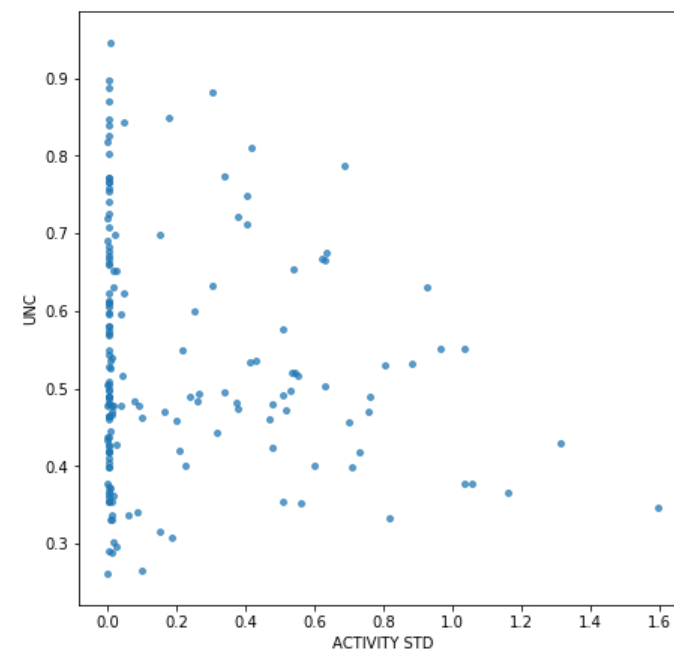

# CHEMBL3371

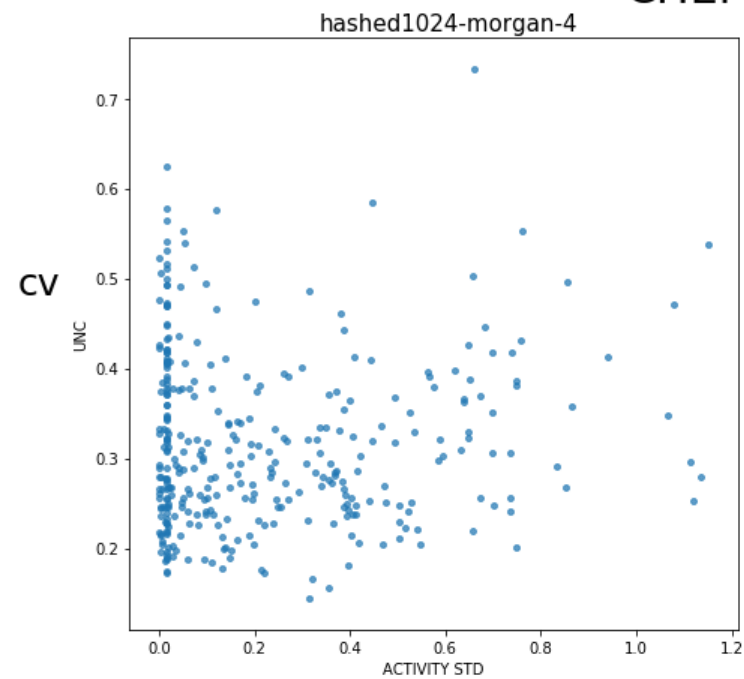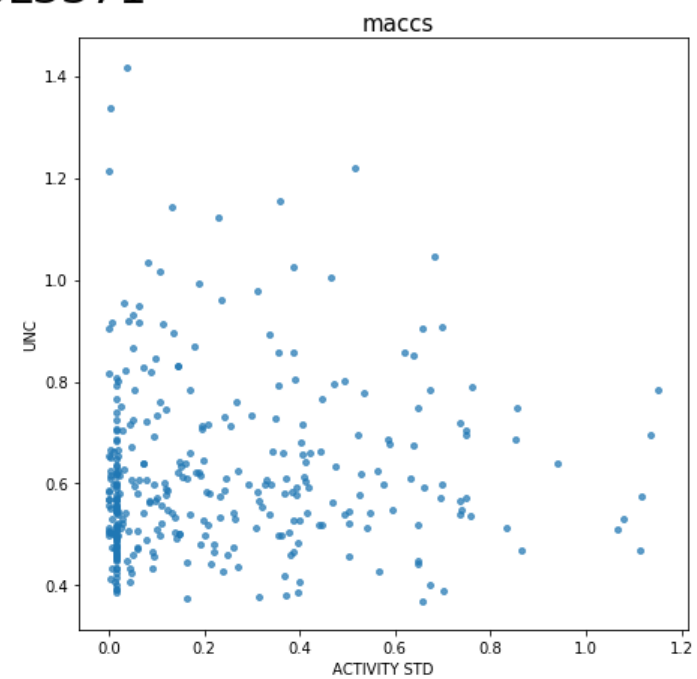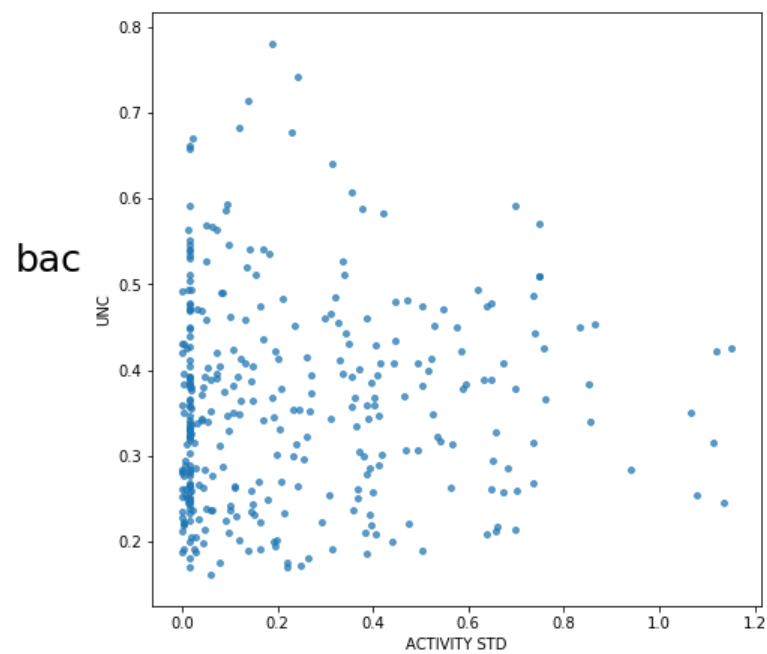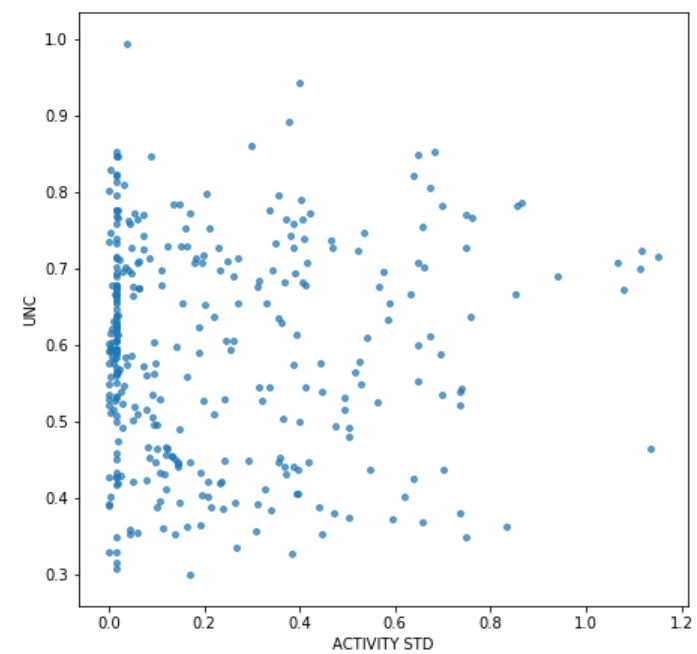

Supplement: Supplementary file 1 [file molecules-25-01452-s001.zip › Supp_Info_for_submission/FileS6.pdf]
